# Mechanistic investigation of aziridine aldehyde-driven peptide macrocyclization: the imidoanhydride pathway†
†Electronic supplementary information (ESI) available: Experimental procedures and characterization data. CCDC 1404272 and 1404273. For ESI and crystallographic data in CIF or other electronic format see DOI: 10.1039/c5sc01958c


**DOI:** 10.1039/c5sc01958c

**Published:** 2015-07-07

**Authors:** Serge Zaretsky, Jennifer L. Hickey, Joanne Tan, Dmitry Pichugin, Megan A. St. Denis, Spencer Ler, Benjamin K. W. Chung, Conor C. G. Scully, Andrei K. Yudin

**Affiliations:** a Davenport Research Laboratories , Department of Chemistry , University of Toronto , 80 St. George Street , Toronto , Ontario M5S 3H6 , Canada . Email: ayudin@chem.utoronto.ca; b Encycle Therapeutics Inc. , 101 College Street, Suite 314 , Toronto , Ontario M5G 1L7 , Canada; c Center for Structural Investigations of Complex Organic Molecules and Polymers , Department of Chemistry , University of Toronto , 80 St. George Street , Toronto , Ontario M5S 3H6 , Canada

## Abstract

Aziridine aldehyde dimers, peptides, and isocyanides participate in a multicomponent reaction to yield peptide macrocycles. We have investigated the selectivity and kinetics of this process and performed a detailed analysis of its chemoselectivity. While the reactants encompass all of the elements of the traditional Ugi four-component condensation, there is a significant deviation from the previously proposed mechanism. Our results provide evidence for an imidoanhydride pathway in peptide macrocyclization and lend justification for the diastereoselectivity and high effective molarity observed in the reaction.

## Abstract 2

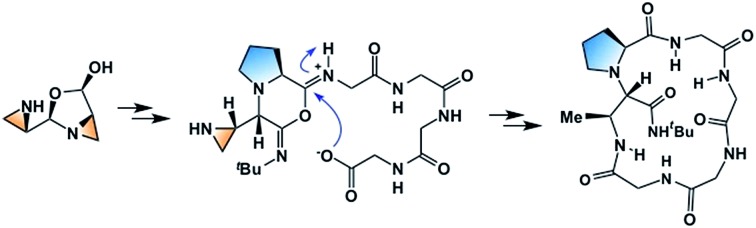
Aziridine aldehydes participate in a multicomponent reaction with α-amino amides and isocyanides to generate reactive imidoanhydride intermediates.

## Introduction

The vast majority of chemical processes rely on polar reactions between nucleophilic (Nu) and electrophilic (E) functional groups.^1^–^4^ Interactions between HOMO and LUMO orbitals of the Nu- and E-containing reagents result in a wide variety of reactive intermediates.^5^–^8^ One of the long-standing interests of our research program has been to evaluate underexplored reactive intermediates and to seek their application in the synthesis of macrocycles.^1^ To facilitate the discovery of such entities, we have focused on kinetically amphoteric molecules.^9^ Over the years, we have contributed to this area by methodically separating the Nu and E nodes by one or more atoms and evaluating the resulting molecules in various contexts. In particular, the aziridine-10 and boron-containing^11^ molecules developed in our lab have afforded new chemoselective processes. Our ongoing studies stress the importance of seeking topologically different combinations of Nu and E nodes in amphoteric molecules.^9^

**Fig. 1:**
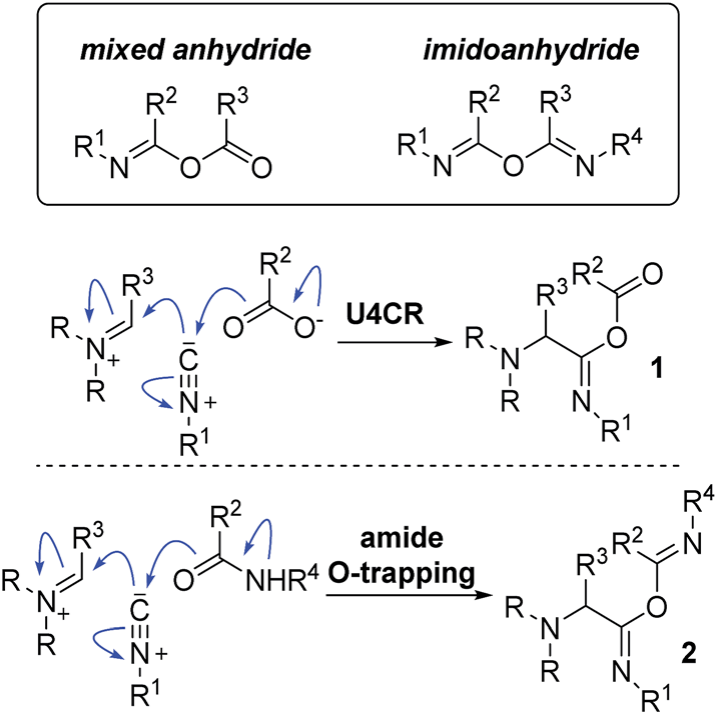
Comparison of the mixed anhydride *versus* the imidoanhydride intermediates in a multicomponent reaction.

When two different amphoteric molecules are forced to react in the presence of one another, cooperative behavior is often observed.^12^ In our effort to develop new applications of aziridine aldehydes, we have been exploring their reactivity with isocyanides. Isocyanides exemplify a [1,1] type of amphoteric species and are associated with two quintessential multicomponent reactions (MCRs) – the Passerini and the Ugi processes.^13^–^20^ The mixed anhydride **1** (Fig. 1) is the engine of the Ugi process, amalgamating the elements of isocyanide with those of imine and carboxylic acid.^21^,^22^ Comparatively, the imidoanhydride pathway, in which an amide acts to trap the nitrilium ion, has been much less explored.^23^,^24^ Much of the work in this field has centered on combining amines and carbonyl compounds with α-isocyano amides in a three-component-four-centered reaction to form a variety of heterocycles.^25^–^27^ Imidoanhydride intermediates, in the form of 5-iminooxazolines, were implicated in Zhu's approach to peptide macrocyclization, but only as precursors towards an activated spironolactone species, which served as the penultimate intermediate for macrocylization.^28^–^30^ Formation of imidoanhydrides by amide attack of nitrilium ions is not limited to the use of α-isocyano amides. For example, Herdtweck and coworkers utilized a three-component reaction with amino acid amides, aldehydes, and isocyanides towards the formation of substituted 2-(cyanomethyl-amino)-acetamides.^31^

In this paper, we evaluate multicomponent reactivity between isocyanides, aziridine aldehydes, and linear peptides towards peptide macrocycles, an increasingly important class of molecules.^32^ As part of this study, we have identified a pathway that involves an imidoanhydride intermediate (**2**). This pathway differs substantially from conventional Ugi MCR reactivity. We present evidence that implicates imidoanhydrides in aziridine aldehyde-driven macrocyclization of peptides. Our study underscores the notion that kinetically amphoteric molecules provide fertile grounds towards the deployment of underexplored intermediates and their application in the synthesis of valuable products.

### The disrupted Ugi reaction with aziridine aldehyde dimers

**Scheme 1:**
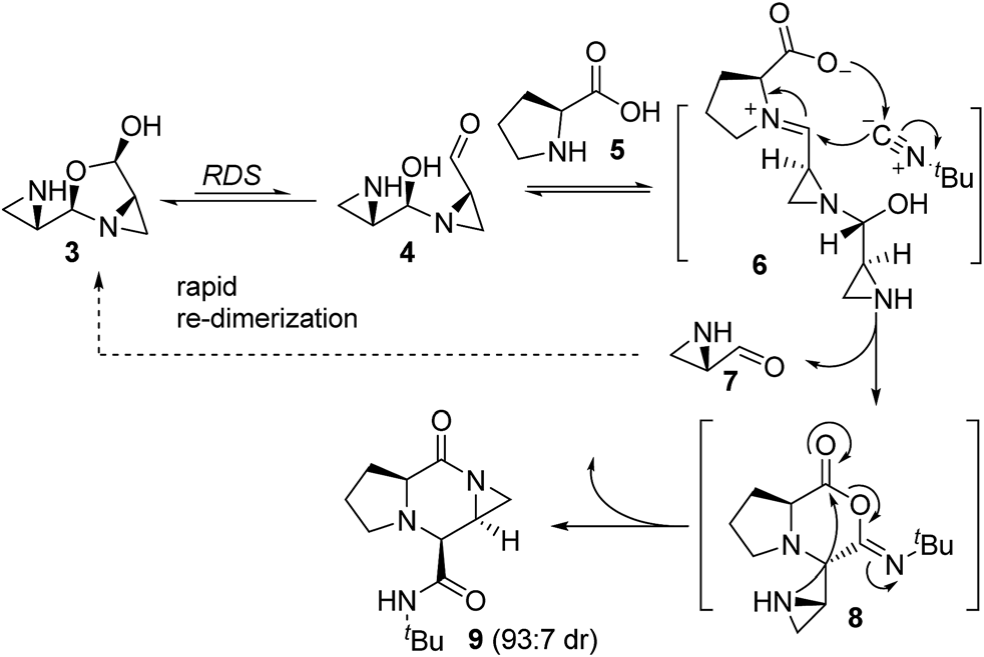
Pendant aziridine nucleophile intercepts the mixed anhydride intermediate in the disrupted Ugi reaction to generate aziridine amide-bearing piperazinones.

Since 2010, we have been investigating the utility of aziridine aldehydes in the synthesis of chiral molecules.^9^,^33^ Because of their importance in medicinal chemistry,^34^–^38^ piperazinones became one of our targets (Scheme 1).^39^ A distinguishing feature of the disrupted Ugi reaction with aziridine aldehydes is that, due to a high kinetic barrier imposed on condensation, the aziridine amine functionality is orthogonal in reactivity to the aldehyde carbon. This allows the NH aziridine and aldehyde to behave independently.^10^,^33^ In piperazinone synthesis, the aldehyde group initiates the multicomponent process by engaging the amino acid amine, whereas the NH aziridine acts as the terminal nucleophile at a later stage.^21^,^40^,^41^ Using this chemistry, a variety of aziridine amide-bearing piperazinones have been prepared from a multitude of primary and secondary amino acids with substrate-dependent diastereoselectivity.^42^

A recent kinetics study, focused on the formation of piperazinone **9**, suggested that the rate-determining step was the formation of open dimer **4** (Scheme 1).^43^ A subsequent DFT computational study of the transition states and intermediates at the MPWPW91/6-31G(d) level of theory suggested that attack of isocyanide onto intermediate **6** (the iminium ion produced from compounds **4** and **5**), is a concerted asynchronous process with carboxylate group engagement that takes place at the same time as the isocyanide addition, directing the face of isocyanide attack to form mixed anhydride **8** (Scheme 1).^43^ From here, the pivotal mixed anhydride is intercepted by the pendant amine nucleophile to generate piperazinone **9**, bearing a characteristic aziridine amide. Subsequently, the disrupted Ugi reaction with aziridine aldehydes was extended to the cyclization of peptide substrates.^39^ Here, we sought to gain insight on the mechanism of the macrocyclization, particularly the formation of the mixed anhydride intermediate.

## Results

### Kinetics of peptide macrocyclization and sequence-dependent reactivity

**Scheme 2:**
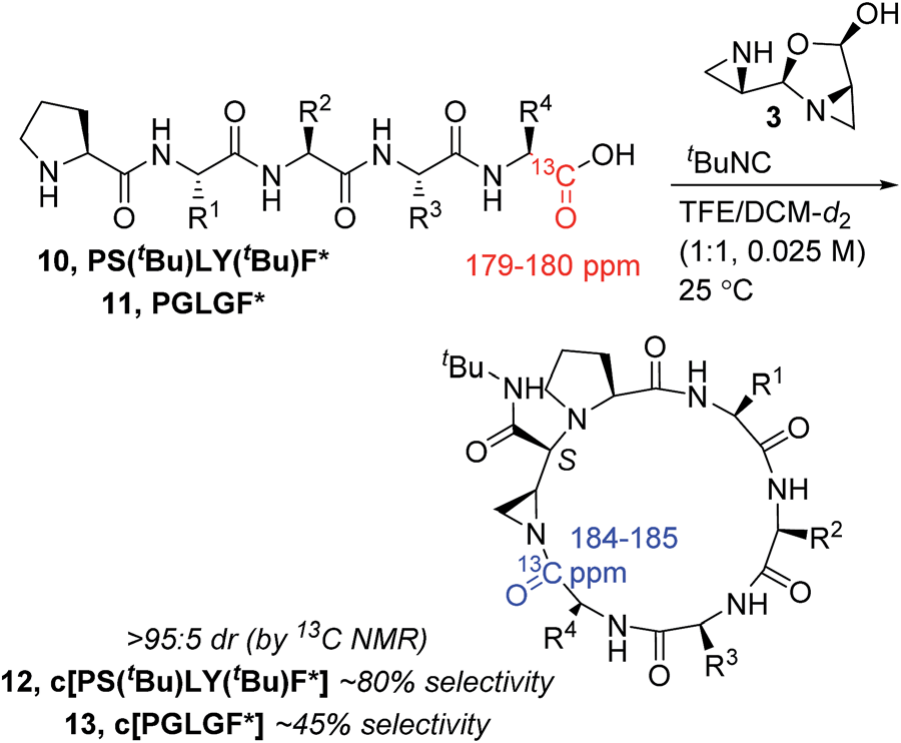
Substrate-dependent selectivity of peptide macrocyclization monitored by ^13^C NMR.

Our first objective was to study the kinetics of the macrocyclization process and evaluate it in the context of other aziridine aldehyde-driven reactions for which we have already gathered mechanistic support.^33^,^42^,^43^ Due to the increased degrees of freedom in linear peptide substrates, we anticipated the kinetic profile to be different from the reaction with a single amino acid. We began by studying the macrocyclization of *C*-terminal ^13^C-labelled PS(^*t*^Bu)LY(^*t*^Bu)F in a TFE/DCM (1 : 1) mixture.^44^ The protected ^13^C-labelled PS(^*t*^Bu)LY(^*t*^Bu)F precursor **10** reacted with excellent selectivity for macrocycle **12** (Scheme 2). The abundance of ^13^C-labelled carbonyl signal made it possible to observe the minor diastereomer with *R* stereochemistry at the exocyclic amide by ^13^C NMR, revealing greater than 95 : 5 diastereoselectivity.

We then turned to pseudo-first order reaction conditions to investigate the kinetics of the macrocyclization process and resorted to the ^13^C NMR methodology previously used in our study of piperazinone formation.^43^ The *k*_obs_ values were derived from the lines of best fit for the logarithmic kinetics plots (see ESI†) and were invariant with respect to concentration of peptide or isocyanide. From the product formation curves (see ESI†), we observed no change in the half-life of the reaction with different concentrations of peptide or isocyanide.

The ^13^C NMR methodology used for peptide kinetics gave us an opportunity to study the conversion and selectivity of the macrocyclization of ^13^C-labelled PGLGF, **11**. Cyclization of **11** was studied over the course of seven hours. The conversion and yield peaked at nearly three hours of reaction time, while the selectivity for **13** remained at approximately 45% throughout the process (see ESI†). This data mirrored the experimental observations that the isolated yields could not be readily optimized past 34% for the unlabelled substrate.^45^ Next, we turned to varying the amino acid residues following proline in the linear sequence, as well as varying stereochemistry, in order to study the sequence-dependent reactivity (Table 1).

**Table 1:** Selected examples of linear peptide (**A**) cyclization leading to macrocyclic (**B**) and amidine-based products (**C** and **D**)

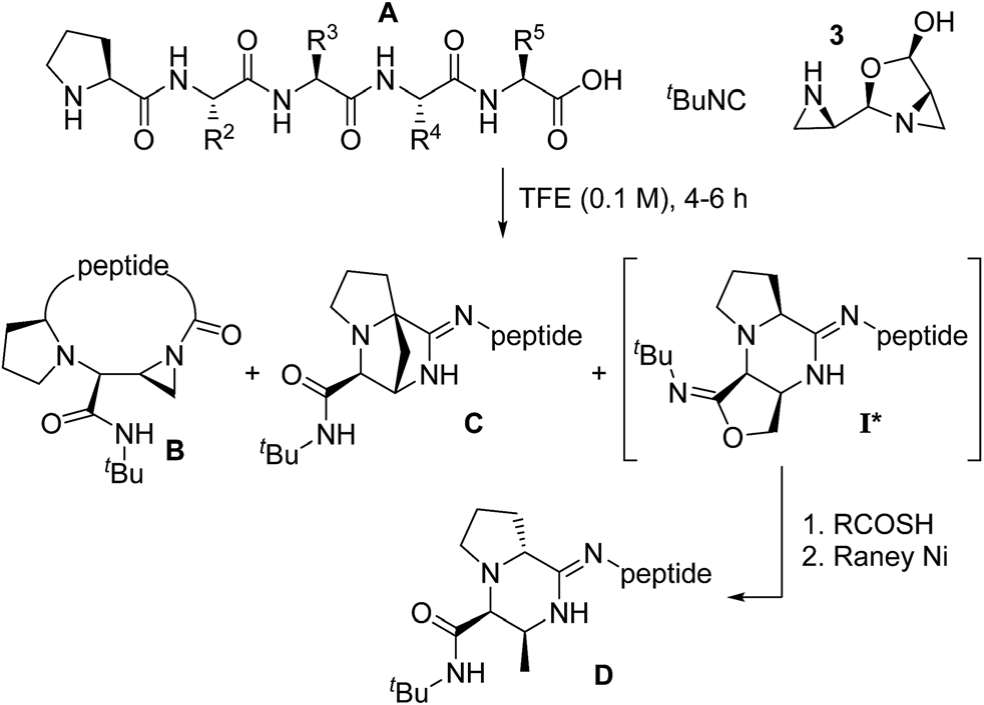		
Linear peptide (**A**)	Sequence	Major product isolated yield
**14**	PGLGF	34% **B**^45^
**15**	PGlGF	28% **B**
**16**	PALGF	12% **B**
**17**	PGLAF	9% **B**^*a*^
**18**	PGLGf	12% **C**
**19**	PS(^*t*^Bu)LGF	49% **B**^*b*^
**20**	PS(^*t*^Bu)LY(^*t*^Bu)F	22% **B**
**21**	PGLGK(Boc)	23% **B**^*a*^
**22**	PGlGk(Boc)	8% **D**^*a*^
**23**	PGLK(Boc)	8% **D**^*a*^
**24**	PGF	8% **C**

^*a*^Isolated after telescopic synthesis.

^*b*^Cyclized with d-leucine-derived dimer.

When selecting linear substrates to study the sequence-dependent reactivity, we focused on peptide **11** (PGLGF*) as the parent sequence, which contained all hydrocarbon side chains. During the course of these studies, two products were identified in addition to the aziridine amide-bearing macrocycle (**B**). These were assigned to a bridged-amidine (**C**) and an amidine (**D**). Bridged-amidine products (**C**) failed to react with several different nucleophiles, confirming that no aziridine ring was present in the structure, while amidine products (**D**) were isolated after telescopic ring-opening with thioacids and desulfurization.^46^ The imidate intermediate I* has also been isolated in select instances (Table 1, see ESI†).

**Fig. 2:**
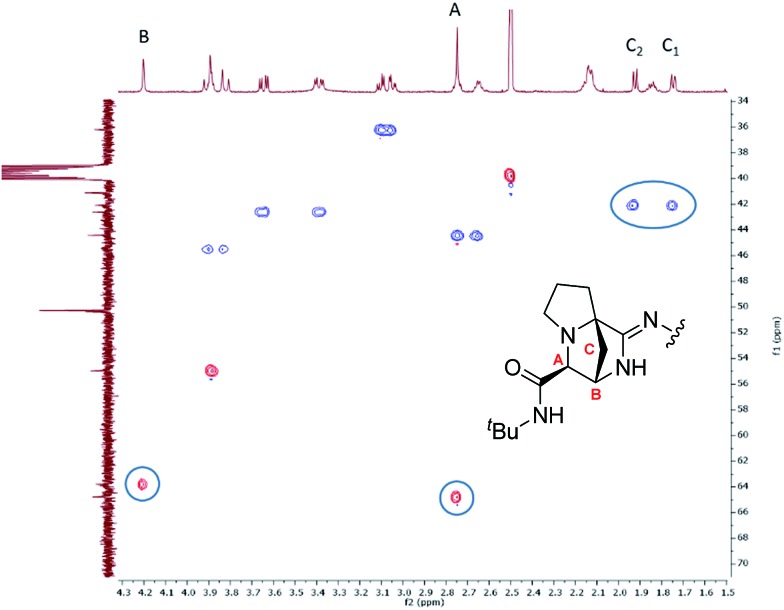
^1^H–^13^C HSQC NMR of **18C** showing the three newly incorporated signals that did not correspond to the chemical shifts observed in aziridine amide-bearing macrocycles.

The structure of the bridged-amidine products was assigned by a combination of 1D and 2D NMR. In the ^1^H NMR spectrum of **18C** in DMSO-*d*_6_, the G_2NH_ (glycine residue adjacent to proline) and P_Hα_ resonances were missing. We identified three new signals (Fig. 2) instead of the expected resonance from the aziridine linker region. Based on ^1^H–^1^H TOCSY and COSY NMR (see ESI†), the connectivity of A → B → C was determined and ^1^H–^13^C HSQC NMR established that resonances A and B were both due to methines, whereas the C resonances emanated from a methylene group (Fig. 2). The ^13^C NMR chemical shifts in DMSO-*d*_6_ for the aziridine linker methine and methylene groups in aziridine-containing macrocycles (*e.g.***14B–16B**) were found around 45 and 20 ppm for the methine and methylene positions, respectively. In contrast, the ^13^C and ^1^H–^13^C HSQC NMR spectra of **18C** were devoid of these resonances, ruling out the possibility that **18C** still contained an aziridine unit.

P_Cα_ was designated as a quaternary center in **18C** as no corresponding P_Hα_ resonance could be observed in either the ^1^H or ^1^H–^13^C HSQC NMR. Another diagnostic difference in the ^1^H–^13^C HMBC NMR was the fact that the F_C=O_ and P_C=O_ chemical shifts were observed at 173 ppm and 169 ppm, respectively. The F_C=O_ chemical shift was notably more upfield than a typical carbonyl of an aziridine amide (>180 ppm),^47^ which was consistent with the free carboxylate structure of **18C**.

**Scheme 3:**
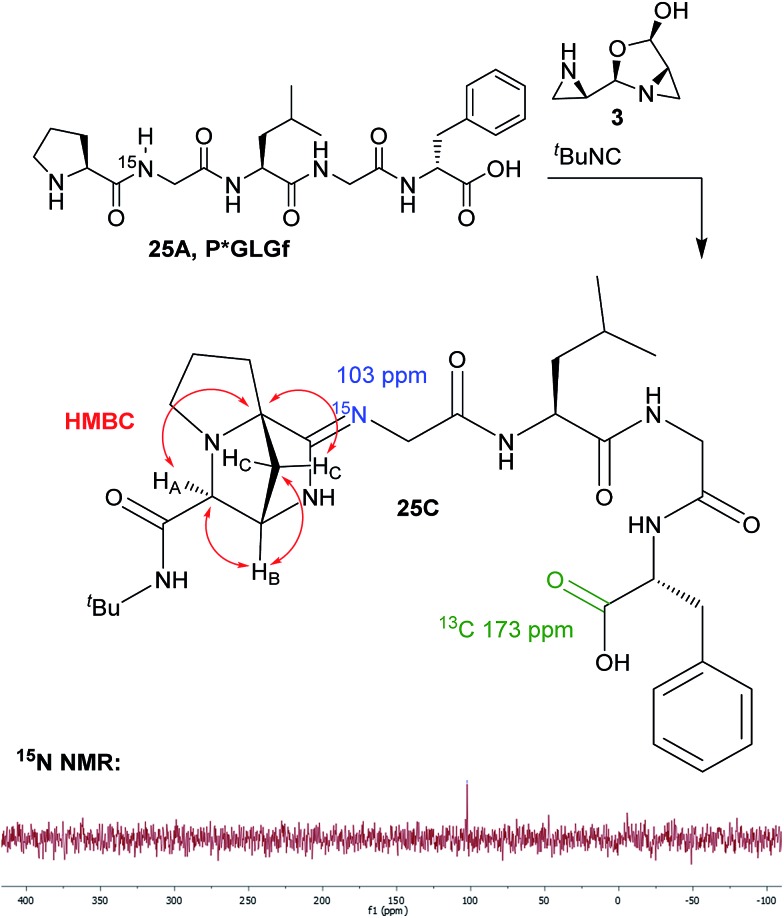
Structure elucidation of a bridged-amidine peptide by ^1^H–^13^C HMBC NMR and ^15^N NMR.

The identity of the nitrogen atoms was confirmed by ^15^N, ^1^H–^15^N HSQC, and ^1^H–^15^N HMBC NMR (see ESI†). In all cases, only the L_NH_, G_4NH_, F_NH_, and exocyclic_NH_ chemical shifts could be found in the typical ^15^N amide region of 110–130 ppm (referenced to liquid NH_3_). An ^15^N-labelled bridged-amidine peptide, **25C**, was isolated and studied by ^15^N NMR. Gratifyingly, a ^15^N signal at 103 ppm was observed for the labelled compound **25C** in DMSO-*d*_6_ with 0.1% formic acid (Scheme 3). By ^1^H–^15^N HMBC NMR, there was a cross peak from the G_2Hα_ to the signal at 103 ppm. The addition of acid was crucial, as no ^15^N signal could be observed without it. Signal sharpening with acid also signified that the labelled nitrogen was basic. The chemical shift at 103 ppm was too far downfield to be consistent with an amine moiety (35–70 ppm), and too far upfield to be consistent with an amide (110–130 ppm), but was in good agreement with reported chemical shifts for protonated amidines.^48^,^49^ Furthermore, the 169 ppm chemical shift of P_C=N_ was also in line with the literature reported chemical shift of amidine carbons.^49^

Further spectroscopic evidence of the bridged-amidine structure arose from ^1^H–^13^C HMBC NMR (Scheme 3). The quaternary P_Cα_ showed a new direct connection to all three atoms of the linker, suggesting that a new C–C bond had formed between P_Cα_ and a bridging methylene group (see ESI†). Finally, a lack of 2D ROESY through-space cross peaks from the phenylalanine residue to the linker atoms and the upfield carbonyl resonance (relative to an aziridine amide at >180 ppm) confirmed that the phenylalanine was still in its carboxylate form and that the rest of the peptide was linked to the bridged-amidine structure through G_2N_.

**Scheme 4:**
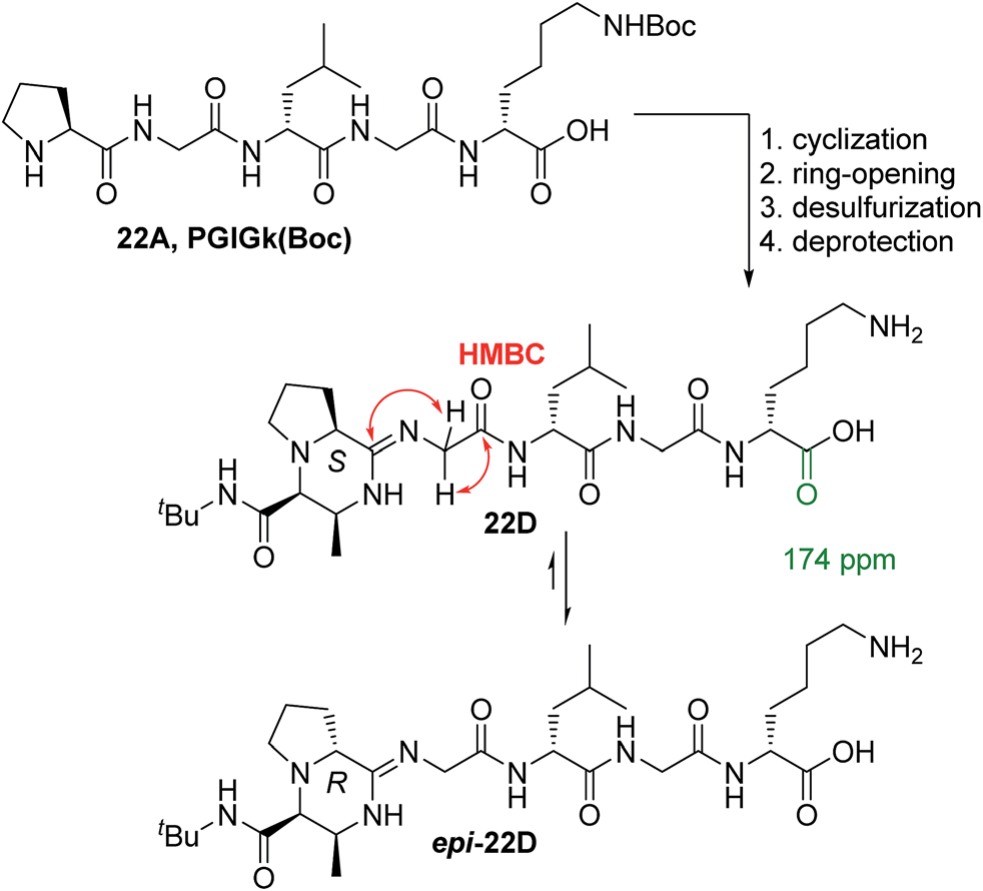
Formation of amidine peptide products along with P_Hα_ epimerization.

To isolate the non-bridged amidine products, we employed the telescopic method^46^ for ring-opening with a thioacid, followed by reductive desulfurization, and side chain deprotection to yield a tractable product. We focused our attention on the cyclization of PGlGk (**22A**). Using a similar set of NMR methods as those used for the structure elucidation of **18C** and **25C** (see ESI†), we deduced the structure of **22D** to also be an amidine (Scheme 4). The ^1^H–^13^C HMBC NMR showed key cross peaks from the G_2Hα_ to both the amidine carbon atom as well as the G_2C=O_.

During reversed-phase HPLC purification of **22D** and other amidine derivatives, we noticed that two peaks corresponding to the amidine product could be resolved, yet after purification they had a tendency to coalesce into one product by NMR. The P_Hα_ atom is allylic with respect to the amidine moiety and thus potentially labile and prone to epimerization to a more thermodynamically stable epimer. Using 2D ROESY NMR, the major isomer of **22D** was assigned as ***epi*-22D** (Scheme 4).^42^

### Model substrates for amidine synthesis

**Scheme 5:**
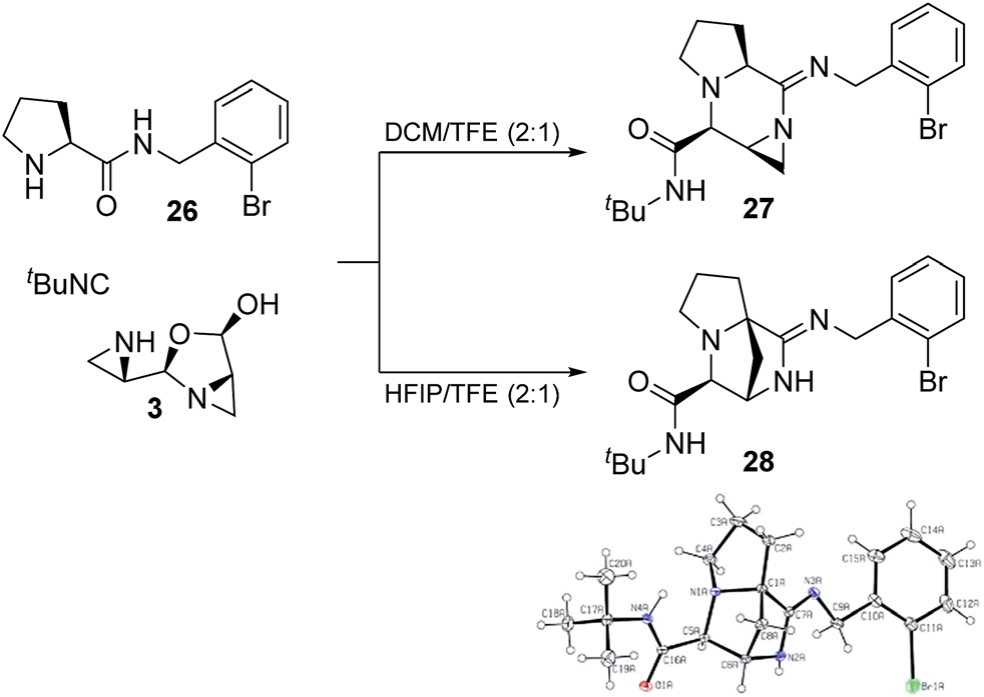
Multicomponent reaction of α-amino amides with aziridine aldehyde dimers and *tert*-butyl isocyanide to form amidine products.

From the structures of both amidine products it was evident that the proline amide participated in the reaction and that the carboxylate end of the peptide was left unmodified. To further investigate this process, we employed proline-amide **26** as a model substrate and exposed it to aziridine aldehyde dimer and *tert*-butyl isocyanide (Scheme 5). In a mixture of DCM and TFE, the isolated product was the aziridine-amidine **27**, which was confirmed by NMR. When the acidic solvent HFIP was used in place of DCM, the bridged-amidine was formed exclusively. Fortuitously, we obtained a crystal structure of **28**, which matched the proposed structure of the bridged-amidine with the hypothesized stereochemistry (Scheme 5). These results indicate that a multicomponent reaction is possible between the proline amide, aziridine aldehyde dimer, and isocyanide.

### Model substrates for mechanism investigation

As the formation of both the bridged-amidine (**C**) and amidine (**D**) products involved the proline amide, we sought to perturb the system by varying either the *N*-terminal proline residue or the amino acid adjacent to proline and studying the effects on the cyclization outcome. First, we employed *N*-methylated amino acids in an attempt to affect the nucleophilicity of the proline amide. We observed no productive reaction from sarcosine- or *N*-methyl-leucine-containing peptides P-Sar-LGF and P-MeLeu-LGF and could not isolate neither the peptide macrocycles nor the amidine and bridged-amidine products (see ESI†).

Next, we attempted to prevent bridged-amidine formation in analogues of parent peptide PGLGf (**18**). (l-β-homoPro)-GLGf failed to produce any of the expected products (see ESI†). With (l-α-MePro)-GLGf, formation of the amidine product was observed by NMR, but no bridged-amidine product was observed or isolated (see ESI†).

Finally, we turned to an analogue of the ^13^C-labelled PS(^*t*^Bu)LY(^*t*^Bu)F linear peptide **10** to obtain finer detail on the features that govern productive macrocyclization. (l-β-HomoPro)-S(^*t*^Bu)LY(^*t*^Bu)F failed to react productively to make the macrocycle or either of the amidine products (see ESI†).

In the formation of piperazinones with proline, we observed a strong dependence for diastereoselectivity on the match or mismatch of amino acid and aziridine aldehyde stereochemistry.^42^*S*-Aziridine aldehyde dimer, **3**, produced piperazinones from l-proline with good diastereoselectivity, whereas the heterochiral pairing of *R*-aziridine aldehyde dimer with l-proline led to poor diastereoselectivity. When **10** was exposed to heterochiral pairing conditions (*R*-aziridine aldehyde dimer instead of *S*-aziridine aldehyde dimer **3**), only trace aziridine amide peaks were detected by ^13^C NMR (see ESI†). The majority of the labelled signals were found in the carboxylic acid region of the spectrum. Resonances in this region were earlier observed in the amidine and bridged-amidine products (Schemes 3 and 4). The appearance of two small aziridine amide peaks suggested a diastereomeric mixture, similar to what was seen in the case of piperazinone formation with proline under heterochiral pairing conditions.^42^

## Discussion

**Scheme 6:**
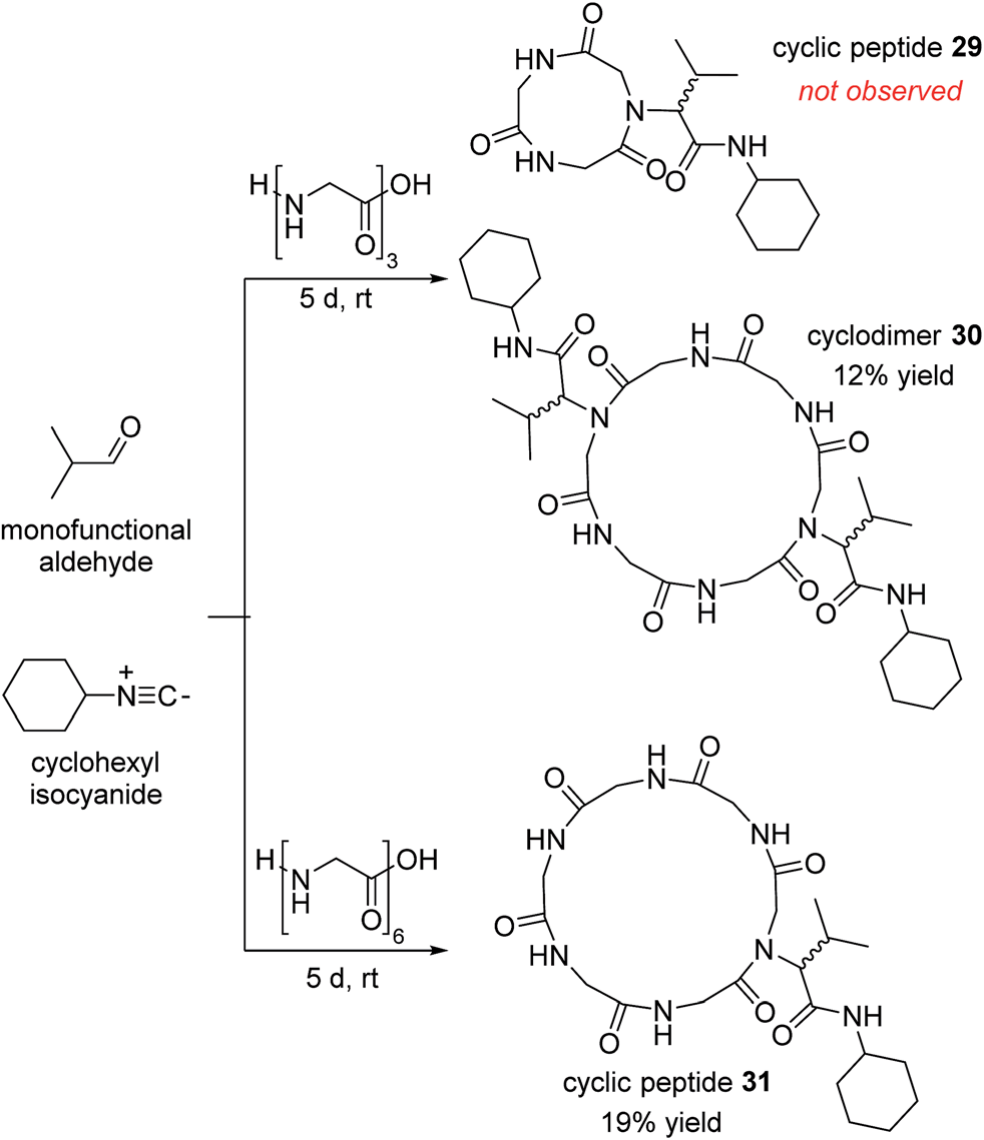
Götz's polyglycine peptide cyclization by Ugi MCR.

The mixed anhydride **1** (Fig. 1) is the cornerstone of the Ugi MCR. The anhydride's formation is the penultimate step in which all four components of the reaction participate. A subsequent Mumm rearrangement then produces the desired α-acylaminoamide products.^12^,^16^–^18^,^50^–^52^ Götz and coworkers, and later the Wessjohann group, pioneered the use of the Ugi MCR in macrocyclization reactions.^53^–^56^ In both cases, the reactions have been proposed to go through the mixed anhydride intermediates to yield macrocyclic amides.^57^ The classic issue of oligomerization in macrocyclization reactions is also present in the Ugi MCR variants. For example, Götz reported that when the tripeptide (glycine)_3_ was exposed to isobutyraldehyde and cyclohexyl isocyanide, cyclodimer **30** was the only isolated cyclic product (Scheme 6). An additional limitation of the Götz protocol was the lack of diastereoselectivity and long reaction times. On the other hand, the Wessjohann and Zhu groups have realized the utility of forming medium- and large-sized rings *via* multiple multicomponent reactions.^53^,^58^,^59^ The use of bifunctional building blocks such as diamines or diacids was crucial to this strategy. As a result, multiple Ugi reactions created a variety of topologically diverse macrocycles.^54^,^57^

The multicomponent macrocyclization with aziridine aldehyde dimers, peptides, and isocyanides has been a departure from the expected reaction outcome of a macrocyclization by an Ugi MCR. We have observed fast reaction times, excellent diastereoselectivity, and no formation of oligomers. It stood to reason that, in addition to the late-stage participation of the exocyclic aziridine nucleophile, there were other factors that led to these favourable outcomes.

The kinetics and sequence-dependent behaviour of the cyclization reaction led us to formulate an experimentally-derived mechanistic rationale for our macrocyclization.^39^ At the outset, we expected the kinetics and rate-limiting step of the peptide macrocyclization reaction to differ substantially from the cyclization reaction with simple α-amino acids (Scheme 1). The kinetics, however, proved that the reaction behaved very similarly, at least for the highly selective cyclization of ^13^C-labelled PS(^*t*^Bu)LY(^*t*^Bu)F (**10**, Scheme 2), where the kinetics of the reaction were not dependent on the concentration of amino acid or isocyanide (see ESI†). The rate-limiting step appeared to be early in the reaction mechanism. Similar to the case of piperazinone formation, the kinetics of peptide cyclization suggested that the rate-limiting step was the dissociation of aziridine aldehyde dimer **3** to the open dimer species **4** (Scheme 1). While informative on the early steps of the cyclization process, the peptide kinetics did not yield any information on the downstream intermediates or whether the mixed anhydride intermediate was formed. We turned to studying the substrate-dependent reactivity in the macrocyclization process to gain further insight on a plausible mechanism for macrocyclization and formation of amidine products.

**Scheme 7:**
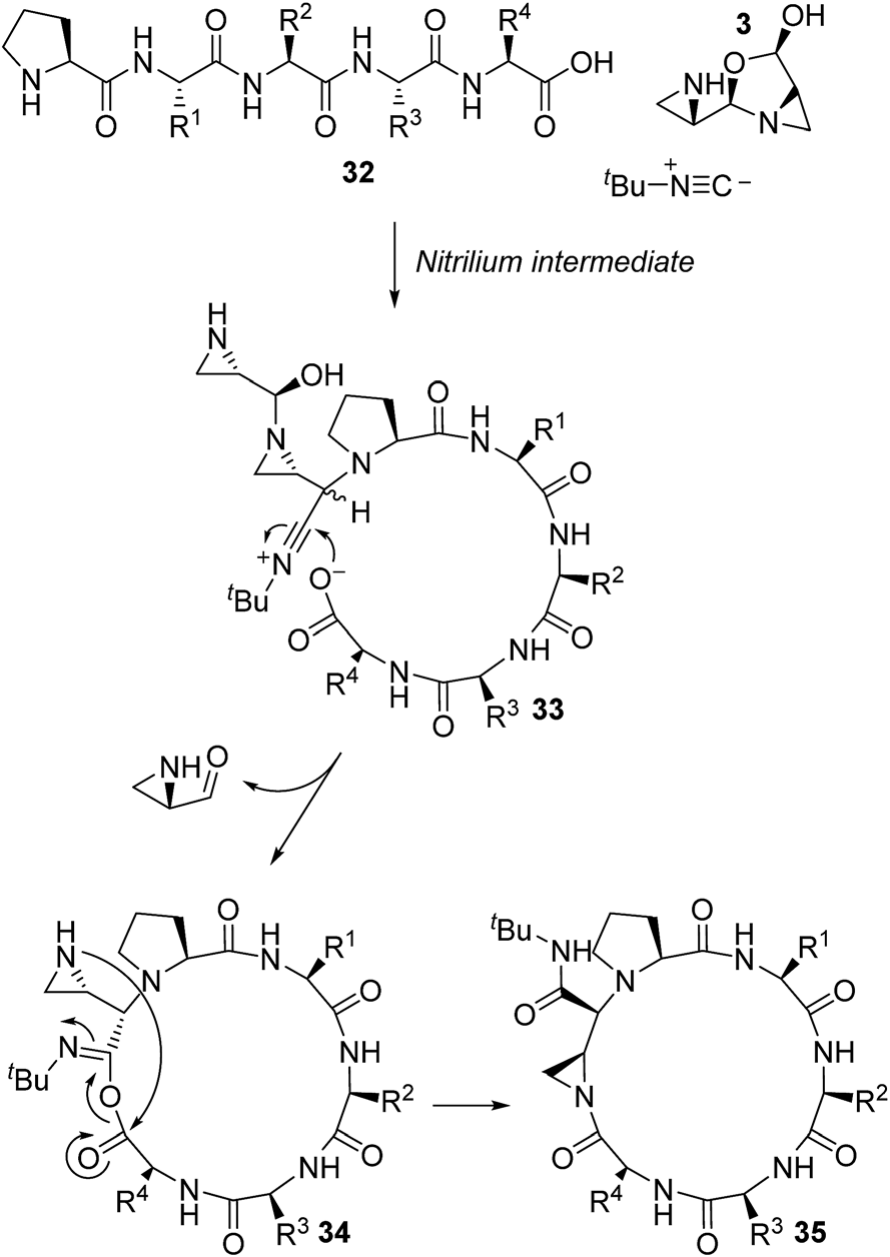
A stepwise pathway with a nitrilium intermediate towards the formation of a cyclic mixed anhydride.

The nitrilium ion is a crucial intermediate in Ugi MCRs.^22^ In our earlier work, we proposed the formation of nitrilium ions that were trapped by the carboxylate functionality as the sole engine of macrocyclization;^60^ however, the sequence-dependent reactivity and formation of amidine products has led us to consider alternate pathways. In the conventional Ugi MCR pathway, attack of isocyanide onto the iminium ion formed from peptide **32** and aziridine aldehyde dimer **3** would form the nitrilium intermediate, **33** (Scheme 7). With no directing carboxylate group, the diastereoselectivity of isocyanide addition would then be lowered, which stands in stark contrast to the excellent diastereoselectivity observed in the disrupted Ugi reaction with peptides. There is a possibility that the carboxylate pre-arranges and participates in the isocyanide addition; however, this would imply that the diastereoselectivity would depend greatly on the peptide sequence, which we have not observed in our study. Accordingly, we do not expect the most favourable pathway for the reaction to proceed by way of a distinct nitrilium intermediate **33**.

**Scheme 8:**
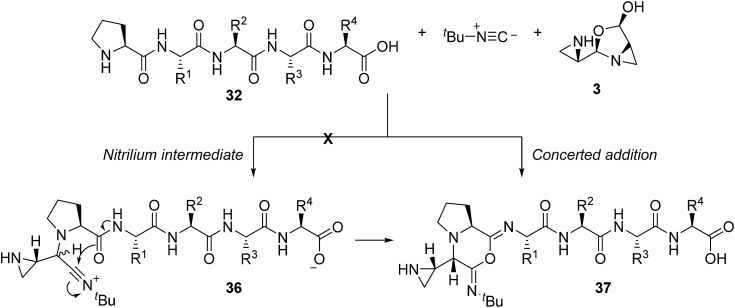
Formation of an imidoanhydride in the disrupted Ugi reaction by a concerted diastereoselective process.

If, however, the proline amide group is considered as a surrogate for the carboxylic acid in the context of aziridine aldehyde-mediated piperazinone formation (Scheme 1), a pathway to imidoanhydride **37** can be envisioned (Scheme 8). In such a case, the proline amide could participate in the stepwise mechanism through nitrilium attack (**36**) or in a concerted addition, similar to the piperazinone chemistry (Scheme 1), to deliver imidoanhydride **37**. Based on the excellent diastereoselectivity of our process (Scheme 2), we expect concerted addition to be the predominant pathway and the root cause of the diastereoselective isocyanide addition.

**Scheme 9:**
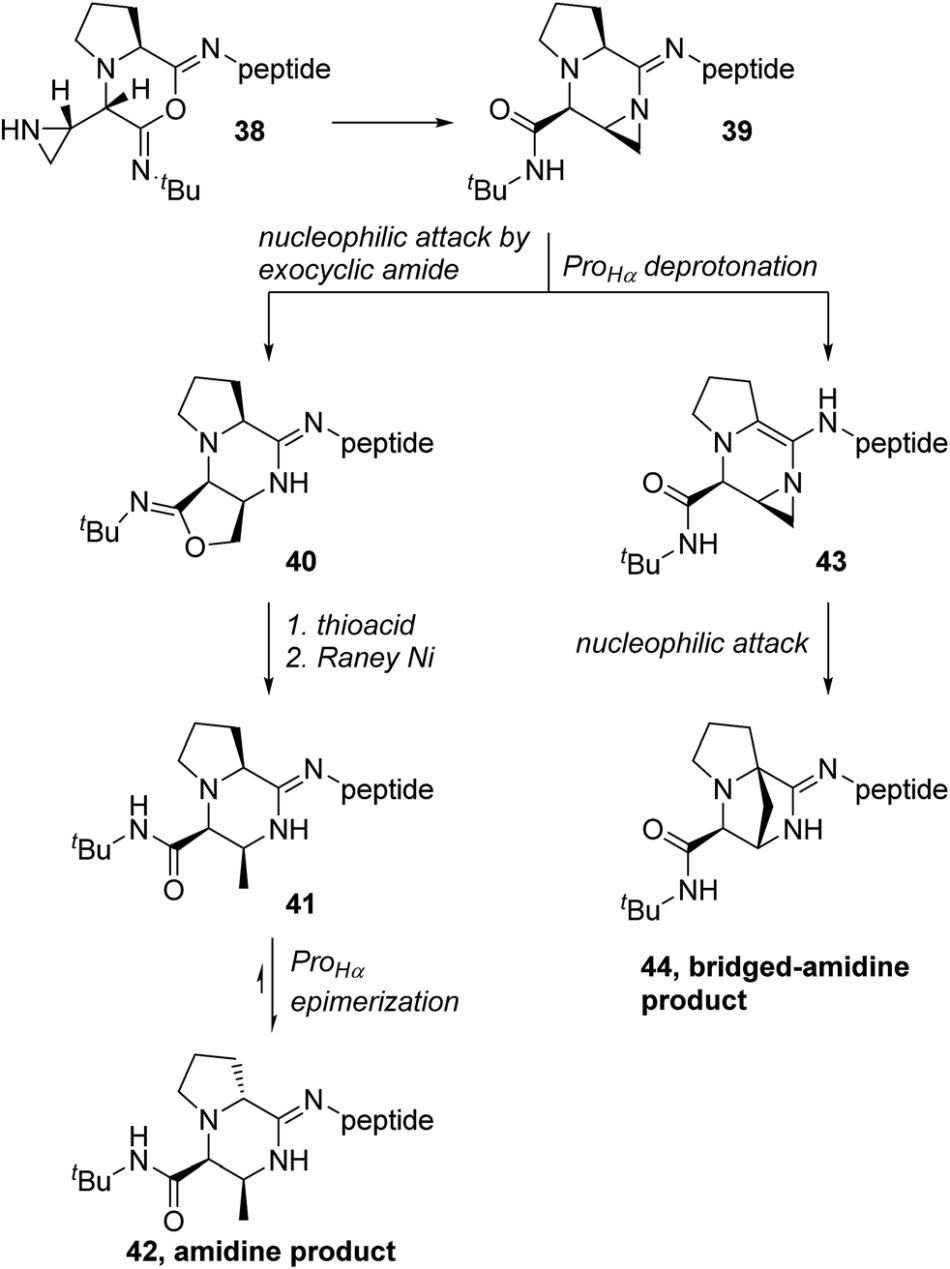
Nucleophilic attack by the pendent aziridine in imidoanhydrides leads to aziridine amidines, which may undergo further transformations to generate the characterized amidine and bridged-amidine products.

The imidoanhydride intermediate **37** bears a close resemblance to the structures identified as the amidine and bridged-amidine products (**C** and **D**, Table 1). If we consider a general proline-terminated peptide, then attack by the aziridine onto imidoanhydride **38** would generate aziridine amidine **39** (Scheme 9). Aziridine amide-containing structures exhibit poor conjugation across the amide bond and are electrophilic at both the carbonyl and aziridine portions.^61^,^62^ Likewise, aziridine amidine **39** possesses an amidine moiety that also exhibits poor conjugation, and in turn, would be quite electrophilic. The carbonyl group of the exocyclic amide has been previously shown to attack aziridines after the Ugi reaction with aziridine aldehydes.^63^ When the exocyclic amide was involved in nucleophilic attack on aziridine amides embedded within piperazinone rings (Scheme 1), a *cis* relationship between the exocyclic amide and aziridine ring was required.^42^ The *trans* isomers did not exhibit the same mode of amide attack. The formation of the aziridine amidine **39** with exceptional diastereoselectivity positioned the exocyclic amide for unencumbered attack onto the aziridine to generate imidate **40**. These imidate products then underwent nucleophilic attack by a thioacid, followed by desulfurization to give amidine **41**. The presence of basic tertiary amine and amidine functionalities next to the P_Hα_ would greatly decrease its p*K*_a_. This was evidenced by the fact that the l-proline-derived amidines spontaneously isomerized into the d-proline analogues (**42**).

Due to poor n–π* overlap, the imine-like character of the aziridine amidine **39** and acidity of the P_Hα_ would enable direct deprotonation of P_Hα_ to make enamine **43** (Scheme 9). C–C bond formation between P_Cα_ and the methylene of the aziridine could occur *via* nucleophilic attack to finally generate the bridged-amidine product **44**. We have not been able to elucidate whether the C–C bond formation would occur by an S_N_1 or an S_N_2 mechanism; however, transannular reactivity has precedence in the formation of conformationally restricted analogues of γ-amino butyric acids and there could also be participation of solvent *via* double inversion.^64^ The bridged-amidine structure of **44** is consistent with the lack of reactivity with nucleophiles as opposed to the aziridine (**39**) or imidate-containing (**40**) intermediates (Scheme 9). Model substrates **27** and **28** were direct proof of the formation of imidoanhydrides such as **38**.

**Scheme 10:**
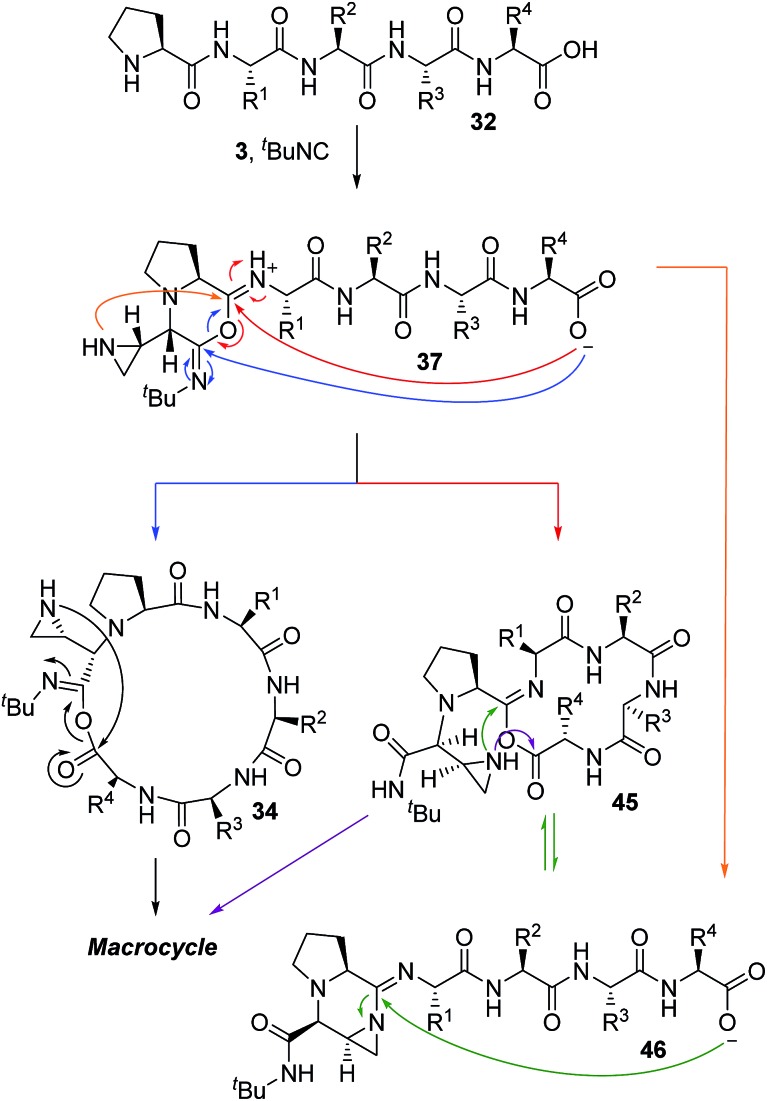
Proposed pathway for macrocyclization of peptides in the MCR with aziridine aldehyde dimers and isocyanides.

Based on the crucial involvement of the proline amide to form the imidoanhydride and its downstream products, we propose that the macrocyclization pathway can also proceed through an imidoanhydride intermediate (Scheme 10). As outlined previously, the high diastereoselectivity for the *cis* diastereomer suggested a highly ordered transition state for isocyanide addition. While downstream pathways to other products did affect the selectivity of the cyclization, the product was always the *cis* diastereomer, thereby casting doubt that isocyanide addition and carboxylate attack by the peptide *C*-terminus were connected. A stepwise pathway to produce nitrilium ion **33** was also doubtful due to the improbability of generating the *cis* diastereomer without a directing group. Thus, we hypothesized that the proline amide group could also be participating in the isocyanide addition in the macrocycle-forming pathway and imidoanhydride **37** could be generated. In a strikingly similar fashion to aziridine aldehyde-mediated piperazinone chemistry, the mismatch case of peptide **10** with *R*-aziridine aldehyde dimer also resulted in a mixture of two diastereomers and was not selective for the macrocyclic product (see ESI†).

**Fig. 3:**
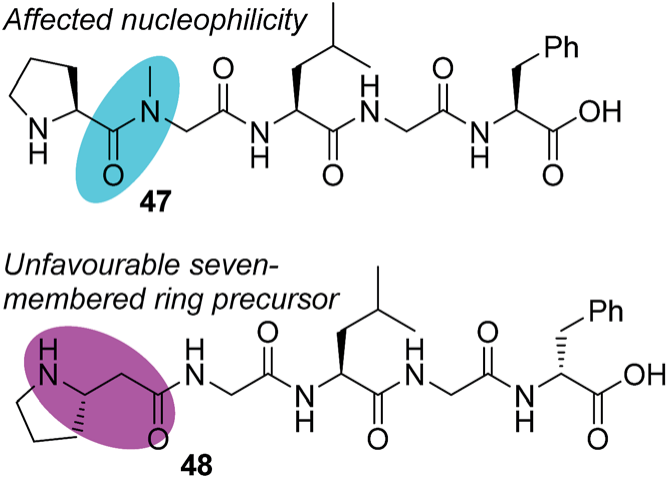
Unfavourable substrates for achieving the desired macrocyclic product.

Further proof for the involvement of **37** in the macrocyclization process arose from evidence borne out of amino acid residue modifications to the peptide *N*-terminus. *N*-Alkyl analogues such as **47** would exhibit reduced nucleophilicity if the reaction was to go through the imidoanhydride intermediate **37**, and these peptides were unreactive under our conditions (Fig. 3). When proline was replaced by β-homo-proline (*e.g.* substrate **48**), the reaction also failed, presumably due to the comparably disfavoured seven-membered ring formation requirement.

Based on the unfavourable cyclizations of **47** and **48**, we conclude that the proline amide is vital to the cyclization and these results imply that imidoanhydride **37** would be the first expected intermediate and the driving force for the diastereoselectivity (Scheme 10). From there, the carboxylate may attack anhydride **37** at either of the two positions, one of which would generate the macrocycle precursor **34** or the 14-membered ring **45**. Compound **45** is yet another mixed anhydride which can undergo intramolecular transacylation with the aziridine to generate the macrocycle. The aziridine can also attack the other position of mixed anhydride **45** to generate **46**, the aziridine amidine that is the precursor to the amidine products. Intermediate **46** can also be formed directly from **37** as presented in the amidine pathways (Scheme 9).

In order to rationalize favourable conditions and a peptide sequence that will result in a macrocycle, it is important to note the duality of bond-forming events in the proposed mechanism (Scheme 10). The first step is similar to the aziridine aldehyde-mediated piperazinone chemistry (Scheme 1) with the formation of a six-membered cyclic intermediate (**37**, Scheme 10) and requires an unperturbed proline amide (Fig. 3). The factors that govern this aspect of reactivity are substitution at the *N*-terminus and match of stereochemistry between the *N*-terminal amino acid and aziridine aldehyde dimer.^42^ After imidoanhydride **37** is formed at the *N*-terminus, the linear peptide tail has to fold in for attack by the *C*-terminal carboxylate. While the experimental evidence obtained in the course of our work is consistent with the intermediacy of **37**, parallel macrocyclization pathways cannot be discounted (Scheme 10).

The abundance of intramolecular pathways leading to amidine and bridged-amidine products (Schemes 9 and 10) ensures no intermolecular reactivity to form oligomers takes places, even at high concentrations.^39^ We attribute the high selectivity for intramolecular reactivity to increased effective molarity provided by the imidoanhydride pathway.^65^,^66^ The imidoanhydride intermediate sets the stereochemistry of the exocyclic amide group and also assembles all parts of the multicomponent reaction. Afterwards, an unfavourable arrangement of amino acid residues, where the carboxylate tail is unable to fold in and reach the piperazinone core, would yield to competing intramolecular amidine pathways and not intermolecular reactivity. The latter observation stands in stark contrast to previously described Ugi MCR macrocyclization chemistry, where, presumably due to the slow transacylation reactivity, cyclodimers were formed. Our results suggest that the imidoanhydride pathway, in conjunction with the pendant nucleophilic aziridine of the aziridine aldehyde dimer, is an empowering engine of macrocyclization.

## Conclusions

We have probed the mechanism of the aziridine aldehyde-promoted macrocyclization reaction. This investigation unveiled the features governing both diastereo- and chemoselectivity of the process. Through kinetic analysis and product characterization, we have found evidence for an imidoanhydride-driven pathway during macrocyclization with aziridine aldehydes. With the *N*-terminal amide acting as a kinetic trap to avoid intermolecular reactivity with the nitrilium ion, the occurrence of multiple intramolecular pathways from the imidoanhydride intermediate avoids the oligomerization issues seen with conventional Ugi reactions to make macrocycles. This serves to increase the effective molarity of the reaction. The amide participation suggests a way for selecting a cyclization-friendly sequence. These considerations will help improve the cyclization process and enable pre-selection of cyclization-friendly linear peptide sequences. In broader terms, the interception of nitrilium ions by nucleophilic amides to yield imidoanhydrides may play a role in other MCRs,^30^ giving rise to new methods for developing heterocycles.

## Supplementary Material

Supplementary information

Crystal structure data
