# Supplementary material for: Mechanistic investigation of aziridine aldehyde-driven peptide macrocyclization: the imidoanhydride pathway
Source: Chem Sci. 2015 Jul 7;6(10):5446–55. doi: 10.1039/c5sc01958c (PMC5949604; doi:10.1039/c5sc01958c)
Supplement: Supplementary file 1 [file SC-006-C5SC01958C-s001.pdf]

Electronic Supplementary Material (ESI) for Chemical Science.
This journal is © The Royal Society of Chemistry 2015

Supplementary Information for

Mechanistic investigation of aziridine aldehyde-driven peptide macrocyclization: the imidoanhydride pathway

*Serge Zaretsky,[1] Jennifer L. Hickey,[1,2] Joanne Tan,[1] Dmitry Pichugin,[3] Megan A. St. Denis,[1,2] Spencer Ler,[1] Benjamin K. W. Chung,[1] Conor C. G. Scully,[1] and Andrei K. Yudin[1]**

[1]Davenport Research Laboratories, Department of Chemistry, University of Toronto, 80 St. George Street, Toronto, Ontario M5S 3H6, Canada

[2]Encycle Therapeutics Inc., 101 College Street, Suite 314, Toronto, Ontario M5G 1L7, Canada

[3]Center for Structural Investigations of Complex Organic Molecules and Polymers, Department of Chemistry, University of Toronto, 80 St. George Street, Toronto, Ontario M5S 3H6, Canada

SUPPLEMENTARY METHODS *3*
General Information 3
Chromatography 3
Nuclear magnetic resonance spectra 3
Mass Spectrometry 3
LC/MS 3
RP-HPLC SP 4
General telescopic synthesis 4
General aziridine-containing peptide synthesis 4
SUPPLEMENTARY DISCUSSION *5*
Assay yield for macrocycle formation 5
Kinetics of peptide macrocyclization 6
Crystal structure of S1 9
Additional figures for structural assignment of bridged-amidine structures 10
Model systems for mechanism of cyclization 11
Isolation of imidate intermediate 13
COMPOUND CHARACTERIZATION *15*
REFERENCES *24*
NMR SPECTRA *25*
CRYSTALLOGRAPHIC DATA *70*

Supplementary Methods

General Information: TFE (2,2,2,-trifluoroethanol) and HFIP (1,1,1,3,3,3-hexafluoro-2-isopropanol) were of reagent grade quality. Linear peptide precursors were synthesized by Fmoc solid-phase-based peptide synthesis using 2-chlorotrityl chloride resin and double coupling steps with HBTU. Amino acid reagents were sourced from AAPPTec LLC, Louisville, Kentucky, USA and P3 BioSystems, LLC, Shelbyville, Kentucky, USA. Peptide grade DIPEA, *tert*-butyl isocyanide, thiobenzoic acid, and Raney®-Nickel (W.R. Grace and Co. Raney®2800, slurry, in H_2O, active catalyst) were sourced from Sigma Aldrich (Oakville, ON). Peptide grade NMP and DMF were sourced from Caledon Laboratories Ltd., Georgetown, Ontario, Canada. ^{13}C-labelled amino acids were sourced from Cambridge Isotope Laboratories, Inc., Tewksbury, Massachusetts, USA. Aziridine aldehyde dimer **3**, the D-serine-based enantiomer, and the D-leucine-derived aziridine aldehyde dimer were prepared as per literature procedures.[1–4]

Chromatography: Flash column chromatography was carried out using Silicycle 230-400 mesh silica gel. Thin-layer chromatography (TLC) was performed on Macherey Nagel pre-coated glass backed TLC plates (SIL G/UV254, 0.25 mm) and visualized using a UV lamp (254 nm) and iodine stain.

Nuclear magnetic resonance spectra: ^{1}H and ^{13}C NMR spectra were recorded on Varian Mercury 400 and Agilent 500 MHz, 600 MHz, and 700 MHz spectrometers. ^{1}H NMR spectra were referenced to $CDCl_3$ (δ 7.26 ppm), CD_3OD (δ 3.30 ppm), DMSO-d_6 (δ 2.50 ppm), Acetone-d_6 (δ 2.05 ppm), and TFE-d_3 (δ 5.02 ppm). ^{13}C NMR spectra were referenced to $CDCl_3$ (δ 77.2 ppm), CD_3OD (δ 49.0 ppm), DMSO-d_6 (δ 39.52 ppm), Acetone-d_6 (δ 29.92 ppm), and TFE-d_3 (δ 126.28 ppm). Peak multiplicities are designated by the following abbreviations: s, singlet; bs, broad singlet; d, doublet; t, triplet; q, quartet; m, multiplet; ds, doublet of singlets; dd, doublet of doublets; dt, doublet of triplets; ddd, doublet of doublet of doublets; bt, broad triplet; td, triplet of doublets; tdd, triplet of doublets of doublets.

Mass Spectrometry: High-resolution mass spectra were obtained on a VG 70-250S (double focusing) mass spectrometer at 70 eV on a QStar XL (AB Sciex, Concord, ON, Canada) mass spectrometer with electrospray ionization (ESI) source, MS/MS and accurate mass capabilities. Alternatively, a JEOL AccuTOF model JMS-T1000LC mass spectrometer equipped with a Direct Analysis in Real Time (DART) ion source was used to acquire high-resolution mass spectra.

LC/MS: Low-resolution mass spectra (ESI) were collected on an HPLC paired to a single-quad mass spectrometer. Compounds were resolved on an Agilent Poroshell 120 EC-C_{18}, 2.7 µm, 4.6 x 50 mm^2 column at room temperature with a flow of 1 mL/min. The gradient consisted of eluents A (0.1% formic acid in double distilled water) and B (0.1% formic acid in HPLC-grade acetonitrile). The gradient method started at 5% of B for the first 1.0 minutes, followed by a linear gradient from 5% to 95% B in 8.0 minutes. The column was then washed with 95% B for 1.0 minutes and equilibrated at 5% B for 1.5 minutes.

***RP-HPLC SP xx to yy (defined individually)**:* Compounds were resolved on an Agilent Zorbax SB-C18, 5.0 µm, 9.4 x 250 mm^2 column on an Agilent semi-preparative purification system at room temperature with a flow of 5 mL/min, or on a RediSep Rf Gold® C18 30 g column on a Teledyne ISCO Combiflash® Rf 200 at room temperature with a flow of 35 mL/min. The gradient consisted of eluents A (0.1% formic acid in double distilled water) and B (0.1% formic acid in HPLC-grade acetonitrile). The gradient method started at xx% of B for the first 3.0 minutes, followed by a linear gradient from xx% to yy% B in 25 minutes. The column was then washed with 95% B for 5.0 minutes and equilibrated at 5% B for 2 minutes.

General telescopic synthesis: To a two-dram vial equipped with a stir bar was added linear peptide (0.15 mmol), parent aziridine aldehyde dimer (21.5 mg, 0.15 mmol), and TFE (1.5 mL). After stirring for two minutes, ^{t}BuNC (18.8 µL, 0.3 mmol) was added and the mixture was stirred at room temperature for six hours. Thiobenzoic acid (70 µL, 0.6 mmol) was then added and following stirring for an additional 1.5 hours, Raney Ni (approx. 2g of slurry) was added and the reaction was left to stir overnight. The mixture was then filtered over celite and concentrated under reduced pressure. Deprotection of the Boc side chain protecting groups was accomplished by dissolving peptides in 50% TFA/DCM (4 mL) and stirring for two hours. Subsequently, the cleavage mixture was evaporated under reduced pressure and the peptides were precipitated twice from diethyl ether. Peptide macrocycles were then purified by RP-HPLC and lyophilized.

General aziridine-containing peptide synthesis: To a two-dram vial equipped with a stir bar was added linear peptide (0.15 mmol), parent aziridine aldehyde dimer (21.5 mg, 0.15 mmol), and TFE (1.5 mL). After stirring for two minutes, ^{t}BuNC (18.8 µL, 0.3 mmol) was added and the mixture was stirred at room temperature for four hours. Then, the mixture was evaporated under reduced pressure, the peptide was re-dissolved in DCM, and purified by flash column chromatography (EtOAc→MeOH).

Supplementary Discussion

Assay yield for macrocycle formation

In order to quantify the assay yield of **13**, the reaction was followed by ^{13}C NMR with ^{13}C-labelled substrate **11** (Supplementary Scheme 1). A ^{13}C-labelled analogue **11** was prepared by solid-phase peptide synthesis (SPPS) with the *C*-terminal L-phenylalanine residue having a ^{13}C-labelled carboxylate. The resulting linear peptide was cyclized with aziridine aldehyde **3** and *tert*-butyl isocyanide in a 1:1 mixture of TFE/DCM-d_2 in a 3 mm NMR tube. The starting material was observed at 179.6 ppm and the product was observed at 184.0 ppm (Supplementary Figure 1).

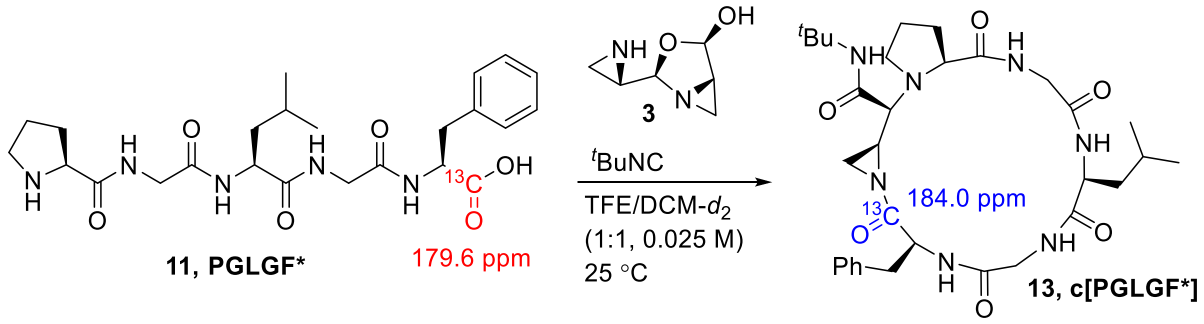

Supplementary Scheme 1 Conversion of **11** to **13** as observed by ^{13}C NMR

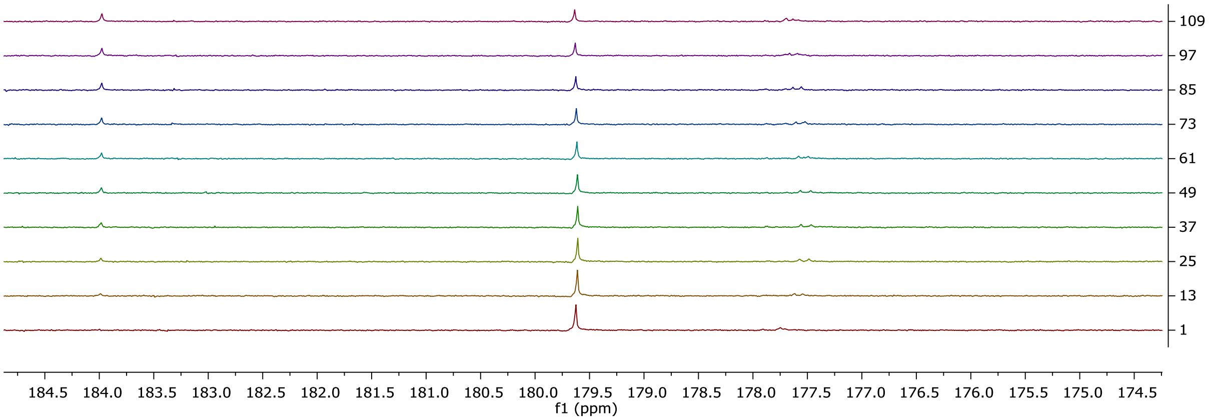

Supplementary Figure 1 Conversion of **11** to **13** as observed by ^{13}C NMR (each shown spectrum was collected 360 seconds apart)

The reaction was followed periodically for seven hours and the ^{13}C peaks for the starting material and product were integrated at each time point (every 30 seconds, Supplementary Figure 2). Conversion was calculated by subtracting the area of the starting material peak at the initial time point. Yield was determined by comparing the relative area of the product peak to the initial area of the starting material peak. Selectivity was calculated by dividing the yield by conversion.

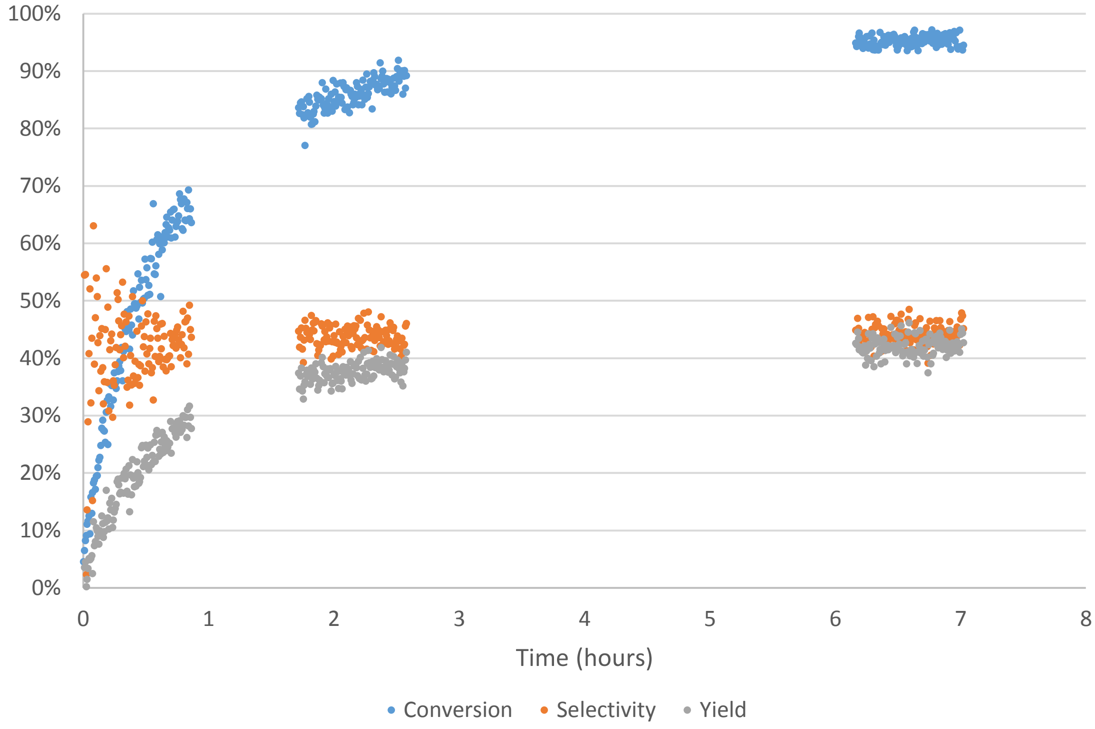

Supplementary Figure 2 Conversion of **11** to **13** as observed by ^{13}C NMR over seven hours

Kinetics of peptide macrocyclization

The reaction kinetics for cyclization of PS(^{t}Bu)LY(^{t}Bu)F were followed by making a ^{13}C-labelled analogue **10** and following the progress by ^{13}C NMR. Selectivity was studied as per the PGLGF results (see previous section), and was determined to be approximately 80% throughout the reaction time.

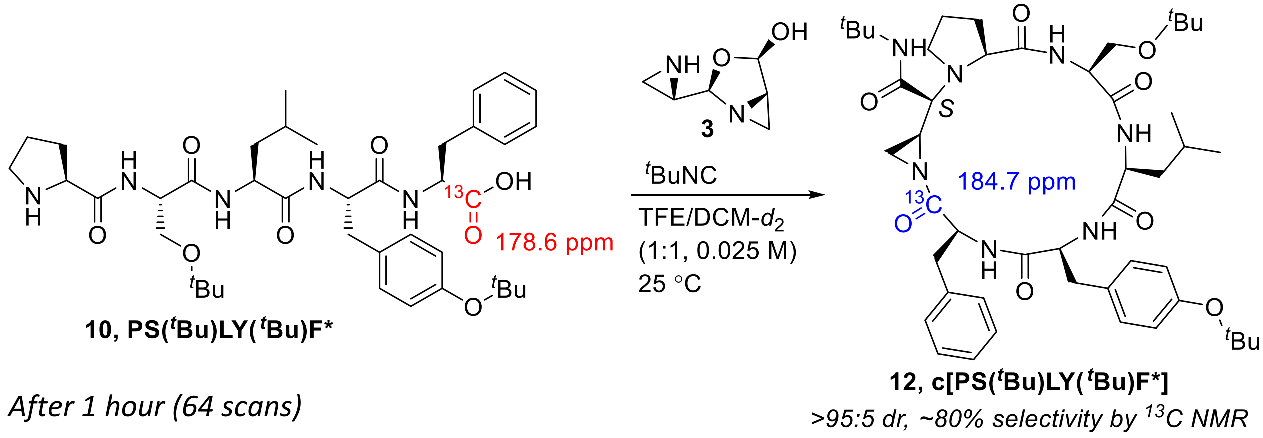

After 1 hour (64 scans)

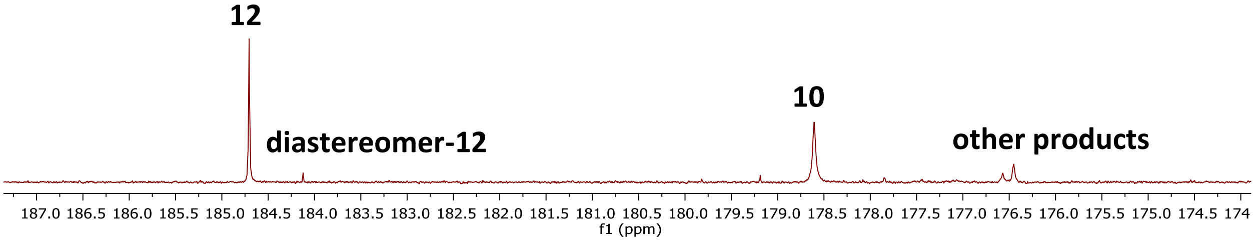

Supplementary Figure 3 Cyclization of **10** produces **12** with good selectivity

Linear peptide **10** was cyclized under a variety of pseudo-first order reaction conditions in aziridine aldehyde dimer. With the initial concentration of 6.25 mM of aziridine aldehyde dimer **3**, a maximum of 12.5 mM of product could be produced. Accordingly, the initial conditions were set to 125 mM in peptide and *tert*-butyl isocyanide, which corresponds to ten-fold excesses of each component. The conditions were then varied to encompass both 15- and 20-fold excesses of peptide or isocyanide (Supplementary Table 1).

Supplementary Table 1 Conditions for pseudo-first order cyclization of linear peptide **10**

Reactant	Initial conditions	15x 10 conditions	15x ^{t}BuNC conditions	20x ^{t}BuNC conditions	20x 10 conditions
**[3]**	6.25	6.25	6.25	6.25	6.25
**[10]**	125	188	125	125	250
**[^{t}BuNC]**	125	125	188	250	125

Concentrations in mM.

The reactions were performed in 3 mm NMR tubes and monitored every 15 seconds, to include a 12 second relaxation delay. The average integrated area of aziridine amide ^{13}C peak

for three successive time points (over 45 seconds) was then plotted and nearly identical curves were observed for all five conditions (Supplementary Figure 4). The k_{obs} values were derived from the lines of best fit for the logarithmic plots (Supplementary Figure 5) and were invariant with respect to concentration of peptide or isocyanide (Supplementary Table 2). From the product formation curves, we saw no change in the half-life of the reaction with different concentrations of peptide or isocyanide. The R^2 value for the 20-fold excess of peptide (Supplementary Table 2, entry 5) was much lower than the other values and could be attributed to limits of solubility for the linear peptide in the reaction conditions.

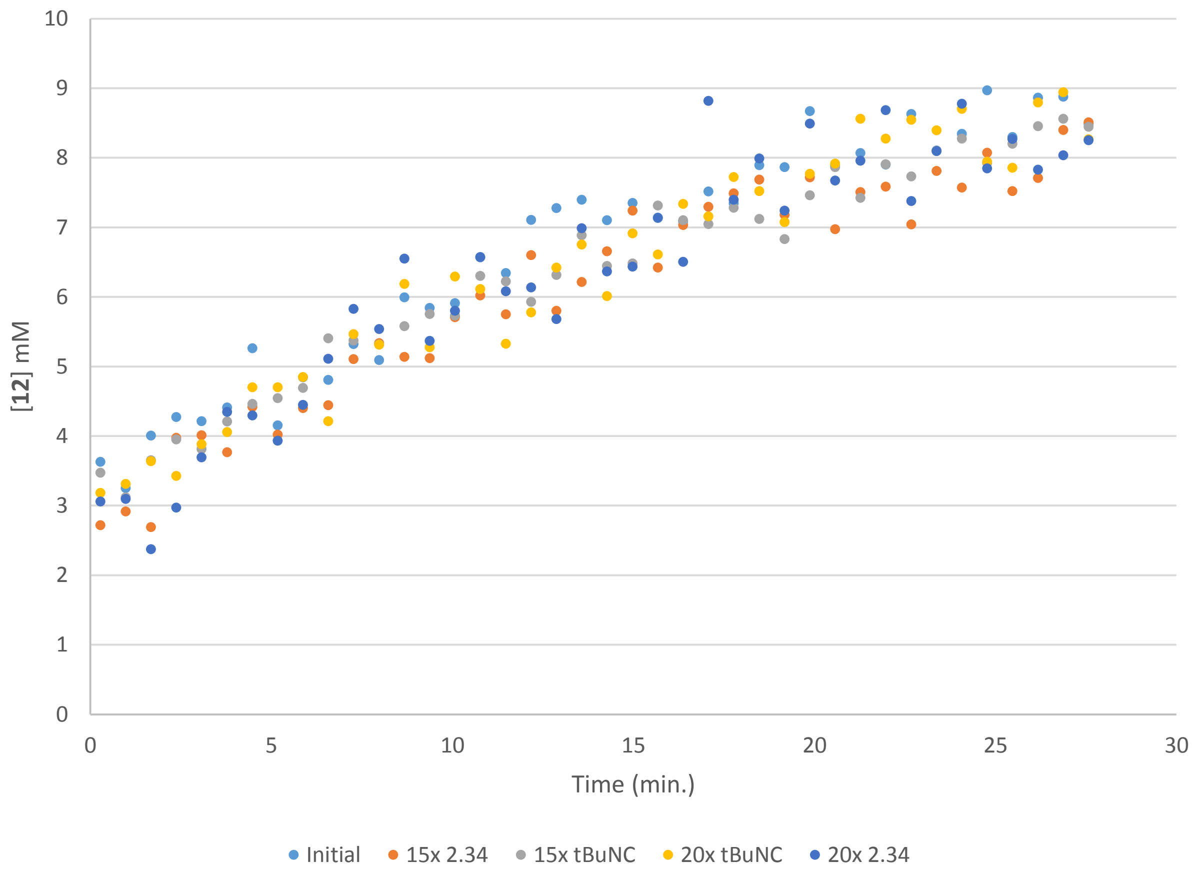

Supplementary Figure 4 Formation of **12** under pseudo-first order conditions in **3**

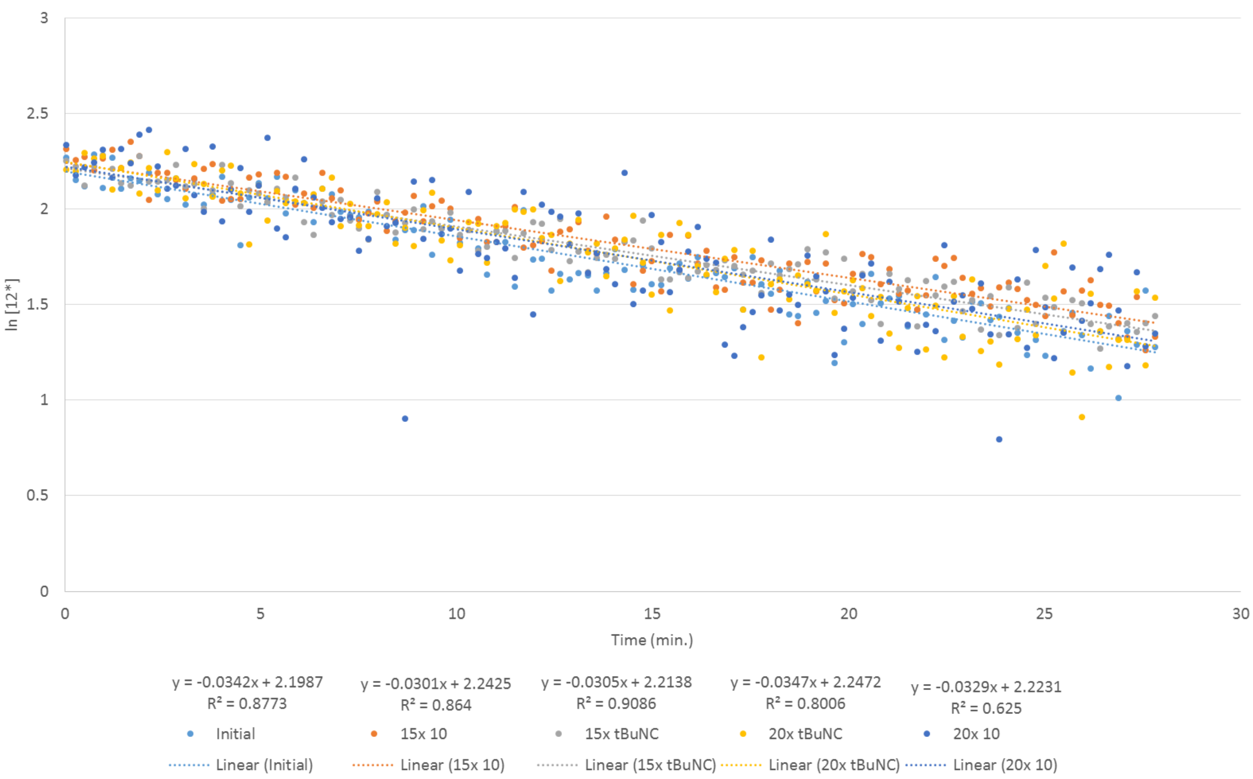

Supplementary Figure 5 Logarithmic plots for kinetics of **12** formation

Supplementary Table 2 k_{obs} calculated from logarithmic plots for the conversion of **10** to **12**

	**Equation**	**R^2**	**k_{obs} (min^{-1})**
**Initial**	y = -0.0342x + 2.1987	0.877	0.034
**15x 10**	y = -0.0301x + 2.2425	0.864	0.030
**15x ^{t}BuNC**	y = -0.0305x + 2.2138	0.909	0.031
**20x ^{t}BuNC**	y = -0.0347x + 2.2472	0.801	0.035
**20x 10**	y = -0.0329x + 2.2231	0.625	0.033

Crystal structure of S1

We have previously shown that the cyclization of secondary amines, both in piperazinone formation and peptide macrocyclization, proceeds with very high selectivity.[5] The linear peptide **16**, PALGF, was cyclized with aziridine aldehyde dimer **3** by the telescopic method to render aziridine ring-opened macrocycle **S1** (Supplementary Figure 6). In an X-ray

crystal structure of **S1**, the stereocenter at the exocyclic amide was determined to be *S*-configured, which parallels our earlier findings.[5]

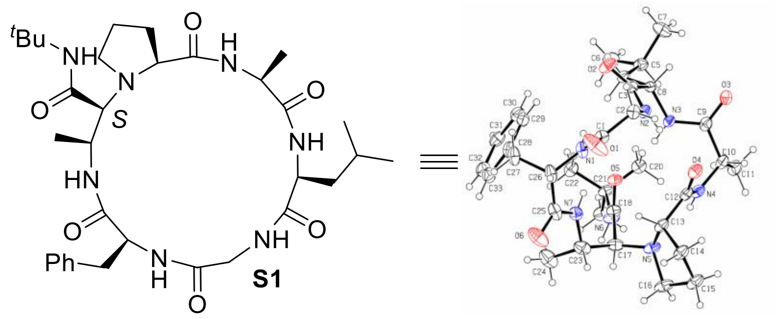

Supplementary Figure 6 Aziridine ring-opened macrocycle **S1**, derived from **16B**, with ORTEP depiction of the crystal structure of **S1** exhibiting *S*-stereochemistry at the newly-derived stereocenter

Additional figures for structural assignment of bridged-amidine structures

Newly noted resonances for the linker protons were found in the ^{1}H NMR of **18C** (Supplementary Figure 7). ^{1}H-^{13}C HMBC NMR was used to establish connectivity to linker carbon atoms (Supplementary Figure 8).

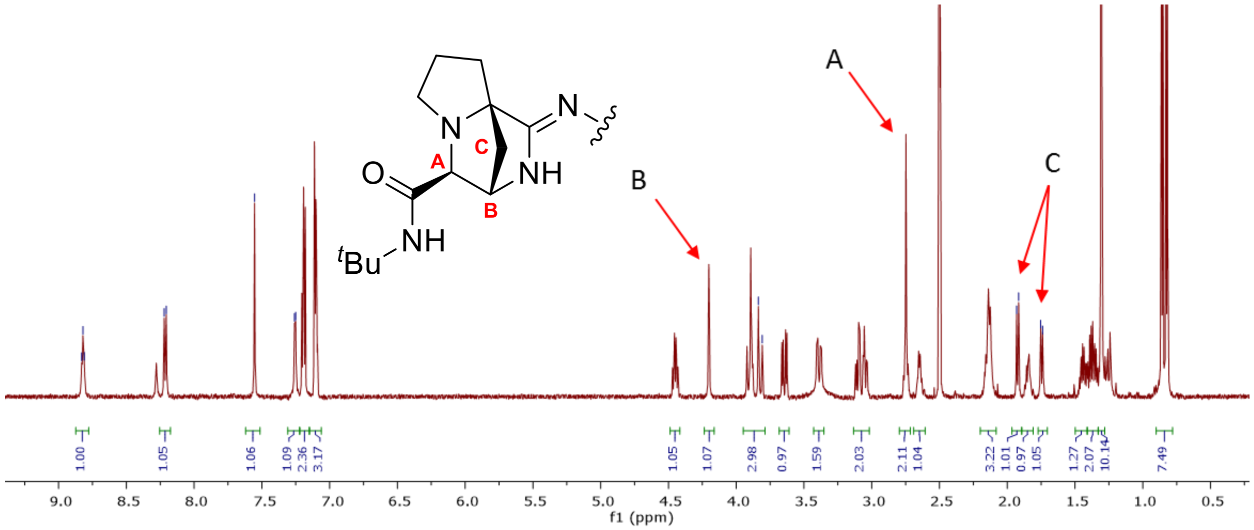

Supplementary Figure 7 ^{1}H NMR of **18C** showing the three newly incorporated linker signals

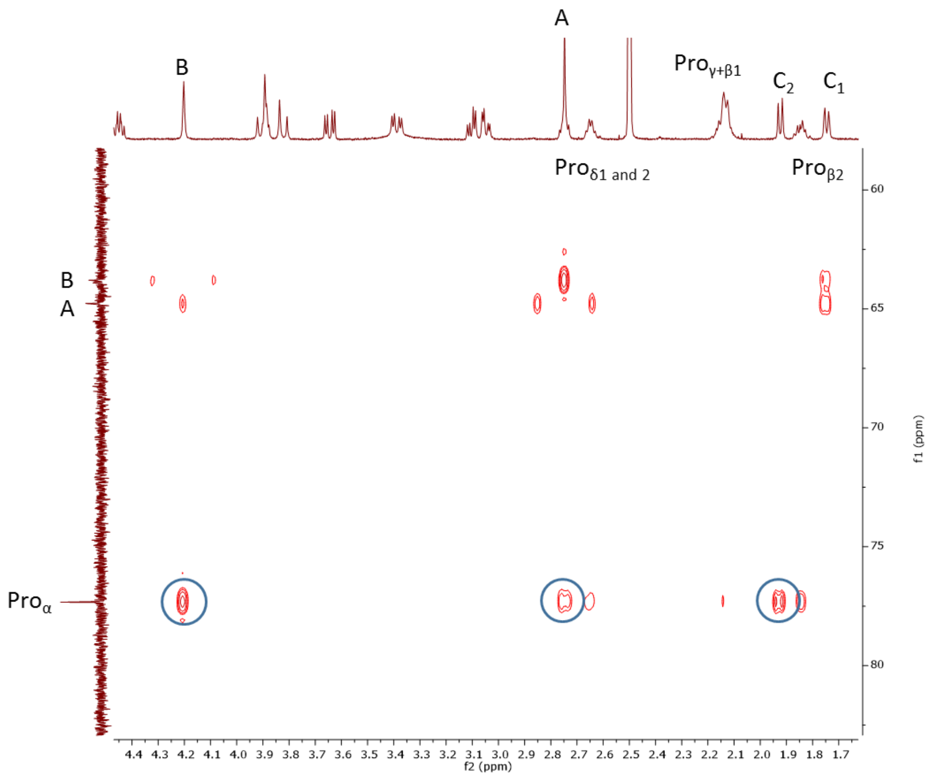

Supplementary Figure 8 ^{1}H-^{13}C HMBC NMR of **18C** showing cross peaks between the P$_\alpha$-C and the new proton resonances A, B, and C

Model systems for mechanism of cyclization

Cyclization of peptides with *N*-methyl amino acids in the second position was not successful (Supplementary Figure 9). Proline analogues also failed to undergo the cyclization reaction (Supplementary Figure 10). The cyclization of PS(^{t}Bu)LYF(^{t}Bu) under heterochiral pairing conditions with aziridine aldehyde dimer enantiomer-**3**, led to only trace macrocycle formation and as a mixture of diastereomers. Finally, no reaction was observed with the L-β-homo-proline derivative **S8** (Supplementary Figure 11).

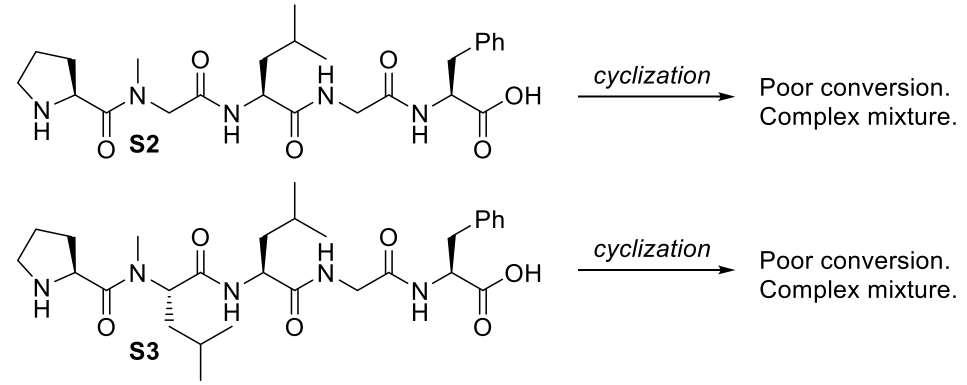

Supplementary Figure 9 Submission of *N*-methylated analogues **S2** and **S3** to cyclization conditions did not lead to formation of macrocyclic or amidine products

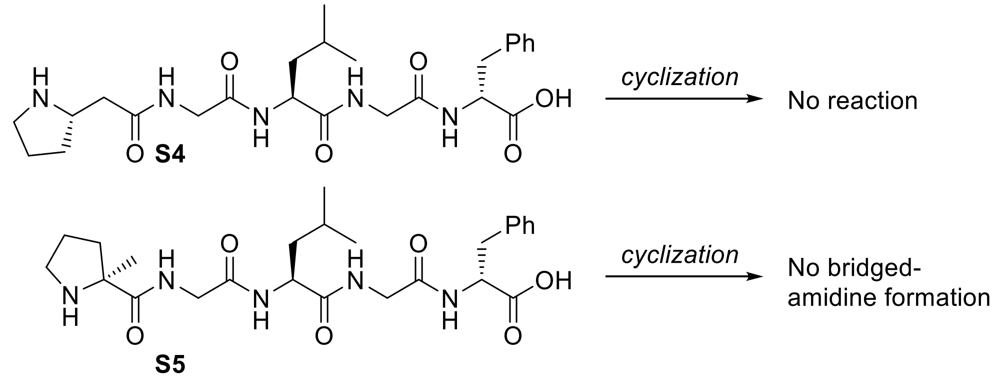

Supplementary Figure 10 Proline analogues **S4** and **S5** block the formation of bridged-amidine products

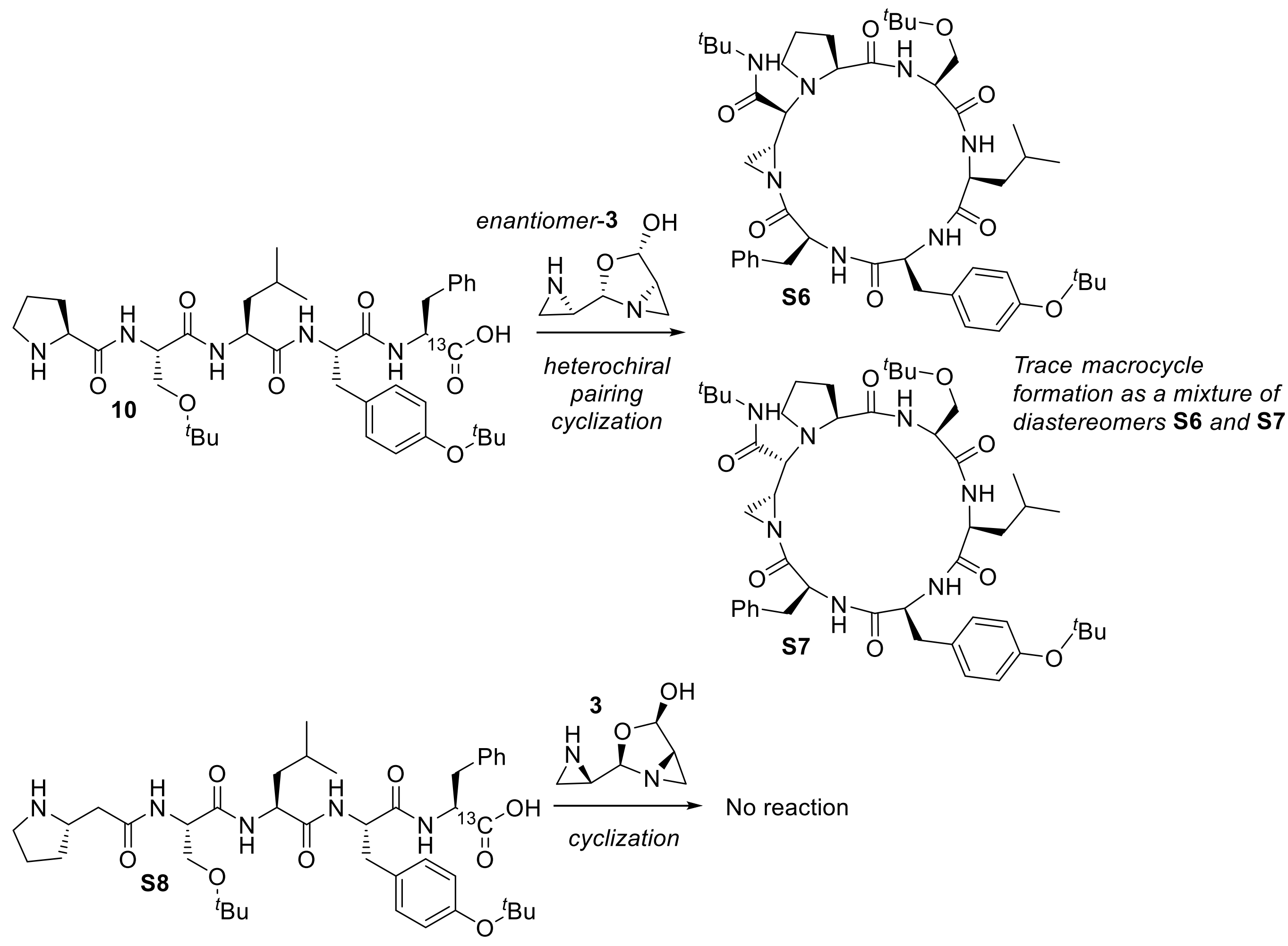

Supplementary Figure 11 Challenging the cyclization of the PS(^{t}Bu)LYF(^{t}Bu) sequence with linear peptide **10** under mismatch conditions with *R*-aziridine aldehyde dimer (*enantiomer*-**3**) or with L-β-homo-proline linear peptide derivative **S8** and *S*-aziridine aldehyde dimer **3** led to poor reactivity

Isolation of imidate intermediate

In order to isolate the imidate intermediate, we resorted to flash chromatography for purification as the imidate was not stable to reversed-phase purification. The dipeptide PL was exposed to cyclization conditions with aziridine aldehyde dimer and isocyanide, resulting in a 21% yield of the imidate produt.

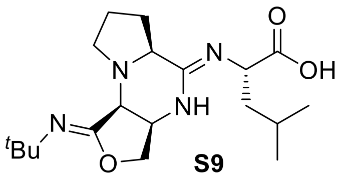

S9

Supplementary Figure 12 Imidate **S9** isolated from PL dipeptide cyclization

Compound Characterization

c[PGLGF] (14B)
Previously reported.[5,6]

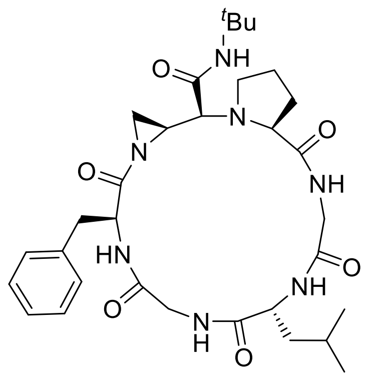

c[PGlGF] (15B)
The peptide was cyclized at 0.2 mmol scale. Following purification by flash chromatography (EtOAc→MeOH), the pure fractions were pooled to afford 35.0 mg of the title compound in 28% overall yield. ESI MS $[M+H]^+$ expected: 626.4, experimental: 626.4. ESI-TOF MS $[M+H]^+$ expected: 626.3661, experimental: 626.3656. ^{1}H NMR (DMSO-d_6, 500 MHz) δ 8.34 (dd, J = 7.2, 5.3 Hz, 1H), 8.18 (d, J = 6.8 Hz, 1H), 8.04 (dd, J = 7.9, 4.3 Hz, 1H), 7.58 (d, J = 8.5 Hz, 1H), 7.30 (s, 1H), 7.27 – 7.21 (m, 2H), 7.21 – 7.16 (m, 3H), 4.54 (td, J = 8.0, 6.7 Hz, 1H), 4.17 (dd, J = 16.0, 8.0 Hz, 1H), 4.12 – 4.04 (m, 1H), 3.73 (dd, J = 16.1, 7.2 Hz, 1H), 3.43 (ddd, J = 16.0, 7.4, 4.7 Hz, 2H), 3.13 – 2.98 (m, 4H), 2.79 (dd, J = 13.3, 6.7 Hz, 1H), 2.43 – 2.34 (m, 1H), 2.35 – 2.29 (m, 1H), 2.25 – 2.20 (m, 1H), 2.12 – 2.01 (m, 1H), 2.01 (dd, J = 3.4, 1.2 Hz, 1H), 1.64 (m, 4H), 1.54 – 1.32 (m, 2H), 1.27 (s, 9H), 0.90 – 0.85 (m, 6H). ^{13}C NMR (DMSO-d_6, 126 MHz) δ 180.9, 174.5, 172.6, 169.6, 168.8, 168.7, 137.5, 129.2, 128.2, 126.4, 70.6, 65.2, 55.1, 52.1, 51.6, 50.2, 42.9, 41.7, 40.3, 38.0, 37.0, 31.5, 30.9, 28.4, 24.2, 23.6, 22.9, 21.6.

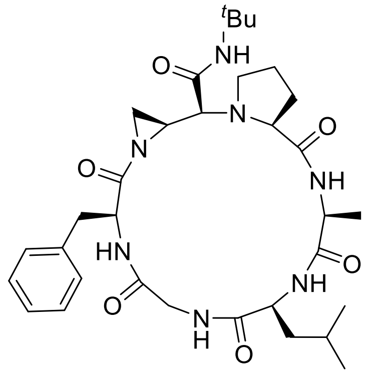

c[PALGF] (16B)
The peptide was cyclized at 0.08 mmol scale. Following purification by flash chromatography (EtOAc→MeOH), the pure fractions were pooled to afford 5.9 mg of the title compound in 12% overall yield. ESI MS $[M+H]^+$ expected: 640.4, experimental: 640.4. LC/MS retention time 6.647 min. ^{1}H NMR (DMSO-d_6, 500 MHz) δ 8.37 (dd, J = 7.0, 5.0 Hz, 1H), 8.18 (d, J = 7.2 Hz, 1H), 8.07

(dd, J = 8.5, 6.7 Hz, 2H), 7.78 (s, 1H), 7.31 – 7.14 (m, 6H), 4.62 (td, J = 9.3, 5.7 Hz, 1H), 4.22 (p, J = 7.3 Hz, 1H), 4.17 – 4.10 (m, 1H), 3.92 (dd, J = 15.6, 7.0 Hz, 1H), 3.55 – 3.49 (m, 1H), 3.23 (dd, J = 15.5, 4.9 Hz, 1H), 3.16 – 3.04 (m, 3H), 2.87 (d, J = 8.0 Hz, 1H), 2.84 – 2.71 (m, 2H), 2.40 (d, J = 5.9 Hz, 1H), 2.18 – 2.14 (m, 1H), 2.05 (ddd, J = 13.7, 8.4, 3.8 Hz, 1H), 1.75 – 1.56 (m, 5H), 1.56 – 1.44 (m, 1H), 1.27 (d, J = 3.4 Hz, 12H), 0.92 – 0.84 (m, 6H). ^{13}C NMR (DMSO-d_6, 126 MHz) δ 182.1, 175.0, 172.6, 171.9, 170.1, 168.5, 137.5, 129.2, 128.1, 126.3, 67.8, 66.8, 63.6, 55.4, 53.1, 51.5, 51.2, 50.7, 50.6, 49.4, 42.9, 36.9, 36.1, 31.0, 30.1, 29.0, 28.5, 28.3, 28.1, 28.0, 24.2, 23.8, 23.1, 23.0, 21.8, 21.4, 18.1, 16.6, 13.7, 13.3.

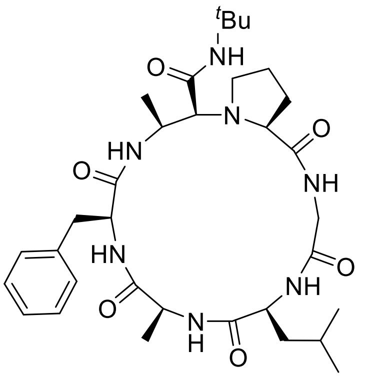

c*[PGLAF] (17B[a])

After cyclization, filtration, and deprotection 45.8 mg of peptide was recovered. Following purification by RP-HPLC SP 15 to 35, the pure fractions were pooled to afford 8.7 mg of the title compound in 9.0% overall yield. ESI MS [M+H]$^+$ expected: 642.4, experimental: 642.4. ESI-TOF MS [M+H]$^+$ expected: 642.3974, experimental: 642.3974. LC/MS retention time 5.771 min. ^{1}H NMR (DMSO-d_6, 500 MHz) δ 8.72 (d, J = 2.9 Hz, 1H), 8.44 (dd, J = 6.9, 5.6 Hz, 1H), 7.94 (s, 1H), 7.32 – 7.11 (m, 6H), 7.05 (d, J = 8.6 Hz, 1H), 6.57 (s, 1H), 4.33 (ddd, J = 11.5, 8.0, 3.4 Hz, 1H), 4.08 – 3.99 (m, 1H), 3.88 (s, 1H), 3.84 – 3.73 (m, 2H), 3.59 (ddd, J = 15.5, 7.0, 1.2 Hz, 1H), 3.27 – 3.06 (m, 4H), 2.90 – 2.82 (m, 1H), 1.94 – 1.83 (m, 1H), 1.77 – 1.61 (m, 5H), 1.39 – 1.29 (m, 1H), 1.21 (s, 9H), 1.10 (d, J = 7.1 Hz, 3H), 1.03 (d, J = 6.4 Hz, 3H), 0.92 (dd, J = 16.0, 6.5 Hz, 6H). ^{13}C NMR (DMSO-d_6, 126 MHz) δ 174.1, 173.5, 172.1, 169.3, 169.2, 168.8, 139.1, 129.1, 128.1, 126.1, 64.9, 64.7, 64.7, 51.4, 50.4, 50.3, 49.7, 45.2, 45.2, 43.3, 41.7, 34.2, 28.8, 28.1, 28.1, 24.3, 23.7, 23.1, 21.1, 18.7, 16.0.

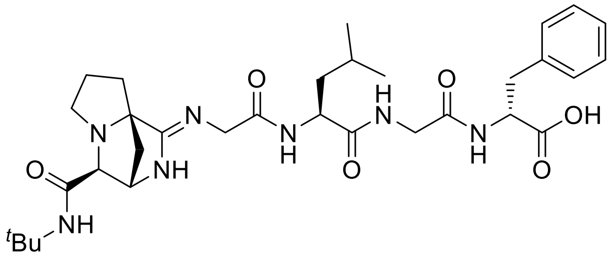

br[PGLGf] (18C)

The peptide was cyclized at 0.51 mmol scale and ring-opened with thiobenzoic acid (2.5 mmol) for one hour prior to solvent evaporation. Following purification first by flash chromatography (EtOAc→MeOH), then by RP-HPLC SP 10 to 50, the pure fractions were pooled to afford 39.0

mg of the title compound in 12% overall yield. ESI MS $[M+H]^+$ expected: 626.4, experimental: 626.4. ESI-TOF MS $[M+H]^+$ expected: 626.3661, experimental: 626.3654. LC/MS retention time 5.840 min. ^{1}H NMR (DMSO-d_6, 500 MHz) δ 8.83 (d, *J* = 6.2 Hz, 1H), 8.21 (d, *J* = 9.1 Hz, 1H), 7.56 (s, 1H), 7.25 (d, *J* = 5.0 Hz, 1H), 7.19 (dd, *J* = 8.0, 7.0 Hz, 2H), 7.10 (ddt, *J* = 7.1, 3.2, 1.5 Hz, 3H), 4.46 (td, *J* = 8.8, 6.3 Hz, 1H), 4.21 (s, 1H), 3.98 – 3.76 (m, 3H), 3.65 (dd, *J* = 16.6, 6.0 Hz, 1H), 3.38 (dd, *J* = 16.6, 5.9 Hz, 1H), 3.08 (m, 2H), 2.81 – 2.70 (m, 2H), 2.66 (q, *J* = 8.1, 7.5 Hz, 1H), 2.21 – 2.07 (m, 3H), 1.93 (d, *J* = 9.4 Hz, 1H), 1.90 – 1.77 (m, 1H), 1.75 (dd, *J* = 9.6, 2.0 Hz, 1H), 1.54 – 1.32 (m, 3H), 1.31 (s, 9H), 0.84 (dd, *J* = 16.7, 6.4 Hz, 6H). ^{13}C NMR (DMSO-d_6, 126 MHz) δ 172.6, 171.9, 169.0, 168.1, 167.4, 165.9, 138.6, 129.7, 127.8, 125.7, 77.3, 64.8, 63.8, 55.0, 50.4, 50.3, 45.4, 44.4, 42.6, 42.1, 41.1, 36.2, 28.4, 26.5, 24.2, 22.6, 22.1, 20.0.

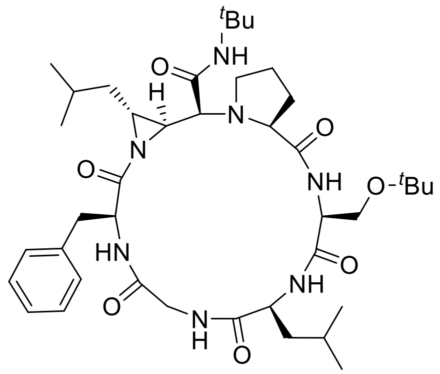

dLeu[PS(^{t}Bu)LGF] (19B[b])

The peptide was cyclized at 0.15 mmol scale. Following purification by flash chromatography (EtOAc→35% MeOH), the pure fractions were pooled to afford 55.9 mg of the title compound in 49% overall yield. ESI MS $[M+H]^+$ expected: 768.5, experimental: 768.5. ESI-TOF MS $[M+H]^+$ expected: 768.5017, experimental: 768.5005. ^{1}H NMR (DMSO-d_6, 500 MHz) δ 8.61 (d, *J* = 6.7 Hz, 1H), 8.14 (t, *J* = 6.1 Hz, 1H), 8.03 (s, 1H), 7.65 (dd, *J* = 16.3, 9.0 Hz, 2H), 7.29 – 7.12 (m, 5H), 4.63 (td, *J* = 9.5, 4.7 Hz, 1H), 4.52 (dt, *J* = 8.9, 4.4 Hz, 1H), 3.95 (dd, *J* = 16.7, 7.7 Hz, 1H), 3.88 (s, 1H), 3.56 (ddd, *J* = 21.3, 9.5, 4.7 Hz, 2H), 3.45 (dd, *J* = 9.2, 4.5 Hz, 1H), 3.24 (dd, *J* = 16.7, 4.7 Hz, 1H), 3.21 – 3.08 (m, 3H), 3.01 (dd, *J* = 6.1, 3.6 Hz, 2H), 2.80 (d, *J* = 6.7 Hz, 1H), 2.76 – 2.68 (m, 1H), 2.17 – 2.06 (m, 1H), 1.98 – 1.90 (m, 1H), 1.88 – 1.65 (m, 6H), 1.64 – 1.53 (m, 1H), 1.27 (s, 9H), 1.09 (d, *J* = 1.0 Hz, 10H), 1.00 – 0.92 (m, 6H), 0.92 – 0.84 (m, 6H).

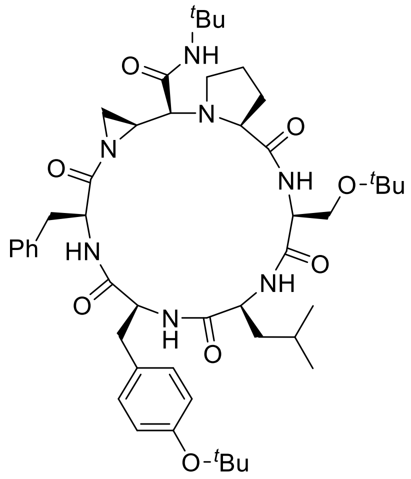

c[PS(^{t}Bu)LY(^{t}Bu)F] (20B)

The peptide was cyclized at 0.15 mmol scale. Following purification by flash chromatography (EtOAc→MeOH), the pure fractions were pooled to afford 28.2 mg of the title compound in 22% overall yield. ESI MS $[M+H]^+$ expected: 874.5, experimental: 874.5. ESI-TOF MS $[M+H]^+$ expected: 874.5437, experimental: 874.5429. ^{1}H NMR (DMSO-d_6, 500 MHz) δ 8.06 (d, *J* = 7.4 Hz, 1H), 8.00 (d, *J* = 9.0 Hz, 1H), 7.92 (m, 2H), 7.49 (s, 1H), 7.32 – 7.16 (m, 5H), 7.03 – 6.96 (m, 2H), 6.87 – 6.78 (m, 2H), 4.60 (td, *J* = 8.5, 6.5 Hz, 1H), 4.32 (dt, *J* = 9.2, 5.2 Hz, 1H), 4.02 – 3.94 (m, 1H), 3.69 (ddd, *J* = 11.4, 7.2, 4.6 Hz, 1H), 3.52 (dd, *J* = 9.1, 5.0 Hz, 1H), 3.44 (dd, *J* = 9.2, 5.4 Hz, 1H), 3.35 (d, *J* = 3.6 Hz, 1H), 3.10 (dd, *J* = 13.6, 6.4 Hz, 1H), 3.02 (td, *J* = 10.3, 4.0 Hz, 2H), 2.96 (ddd, *J* = 8.9, 5.8, 3.6 Hz, 1H), 2.92 – 2.83 (m, 1H), 2.80 (dd, *J* = 13.9, 5.5 Hz, 1H), 2.74 – 2.66 (m, 1H), 2.64 (d, *J* = 8.3 Hz, 1H), 2.29 (d, *J* = 6.1 Hz, 1H), 2.14 (d, *J* = 3.4 Hz, 1H), 2.04 – 1.99 (m, 1H), 1.82 – 1.64 (m, 3H), 1.54 (m, 2H), 1.26 (m, 19H), 1.12 (s, 9H), 0.86 (d, *J* = 6.6 Hz, 3H), 0.78 (d, *J* = 6.5 Hz, 3H).

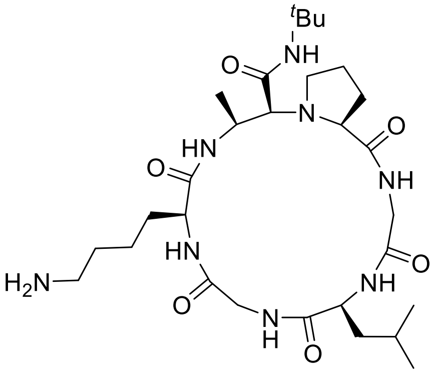

c[PGLGK] (21B[a])

The peptide was cyclized at 0.15 mmol scale with telescopic aziridine ring-opening. After cyclization, filtration, and deprotection 92.9 mg of peptide was recovered. Following purification by RP-HPLC SP 5 to 10, the pure fractions were pooled to afford 24.1 mg of the title compound in 26% overall yield. ESI MS $[M+H]^+$ expected: 609.4, experimental: 609.4; $[M+2H]^{2+}$ expected: 305.2, experimental: 305.2. ESI-TOF MS $[M+2H]^{2+}$ expected: 305.2078, experimental: 305.2088. LC/MS retention time 3.793 min. ^{1}H NMR (CD_3OD, 400 MHz) δ 4.27 (ddd, *J* = 22.4, 12.1, 5.6 Hz, 4H), 4.08 – 3.95 (m, 2H), 3.84 – 3.75 (m, 1H), 3.59 (d, *J* = 15.8 Hz, 1H), 3.38 (dd, *J* =

8.8, 5.1 Hz, 1H), 3.17 – 3.02 (m, 3H), 2.93 (dd, *J* = 16.3, 8.8 Hz, 3H), 2.13 – 1.94 (m, 2H), 1.84 – 1.60 (m, 10H), 1.52 – 1.38 (m, 3H), 1.34 (d, *J* = 9.3 Hz, 12H), 1.30 (d, *J* = 6.7 Hz, 3H), 0.97 (dd, *J* = 6.0, 4.8 Hz, 6H). ^{1}H NMR (DMSO-d_6, 700 MHz) δ 9.07 (t, *J* = 5.2 Hz, 1H), 8.50 – 8.36 (m, 1H), 8.13 (t, *J* = 6.3 Hz, 1H), 7.55 (d, *J* = 7.6 Hz, 1H), 7.23 (d, *J* = 9.0 Hz, 1H), 6.45 (s, 1H), 4.32 (td, *J* = 8.2, 4.6 Hz, 1H), 4.11 (td, *J* = 9.3, 6.6 Hz, 1H), 4.04 – 3.96 (m, 1H), 3.76 (ddd, *J* = 14.6, 9.9, 4.8 Hz, 2H), 3.66 (dd, *J* = 15.5, 7.0 Hz, 1H), 3.52 – 3.46 (m, 1H), 3.25 (q, *J* = 8.0 Hz, 1H), 3.13 (t, *J* = 7.7 Hz, 1H), 2.90 (ddd, *J* = 8.9, 6.9, 4.1 Hz, 1H), 2.83 (d, *J* = 9.5 Hz, 1H), 2.73 (dq, *J* = 13.2, 7.2, 6.6 Hz, 2H), 1.89 (m, 2H), 1.72 – 1.58 (m, 2H), 1.58 – 1.45 (m, 7H), 1.37 – 1.27 (m, 2H), 1.27 – 1.17 (m, 12H), 0.88 (dd, *J* = 8.1, 4.0 Hz, 6H). ^{13}C NMR (DMSO-d_6, 126 MHz) δ 174.1, 173.3, 169.9, 169.7, 169.2, 169.1, 66.1, 64.8, 52.7, 50.8, 50.5, 46.3, 44.9, 43.9, 43.2, 41.9, 38.5, 29.5, 29.2, 28.1, 26.9, 24.3, 23.2, 23.1, 22.6, 22.1, 18.8.

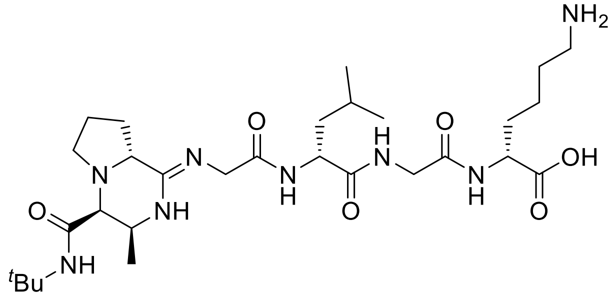

am*[PGlGk] (22D[a])

The peptide was cyclized at 0.15 mmol scale with telescopic aziridine ring-opening. After cyclization, filtration, and deprotection 63.1 mg of peptide was recovered. Following purification by RP-HPLC SP 5 to 15, the pure fractions were pooled to afford 7.9 mg of the title compound in 8.6% overall yield. ESI MS $[M+H]^+$ expected: 609.4, experimental: 609.4; $[M+2H]^{2+}$ expected: 305.2, experimental: 305.2. ESI-TOF MS $[M+2H]^{2+}$ expected: 305.2078, experimental: 305.2089. LC/MS retention time 3.899 min. ^{1}H NMR (DMSO-d_6, 500 MHz) δ 8.83 (s, 1H), 8.60 (d, *J* = 8.9 Hz, 1H), 7.38 (d, *J* = 6.2 Hz, 1H), 7.33 (s, 1H), 4.40 (q, *J* = 7.9 Hz, 1H), 4.13 – 3.97 (m, 3H), 3.91 (d, *J* = 16.8 Hz, 1H), 3.75 (dd, *J* = 16.8, 6.2 Hz, 2H), 3.43 (dd, *J* = 16.5, 5.7 Hz, 1H), 3.19 (s, 1H), 2.97 (d, *J* = 7.9 Hz, 1H), 2.70 (t, *J* = 7.7 Hz, 2H), 2.58 (s, 1H), 2.41 – 2.28 (m, 1H), 1.82 – 1.61 (m, 4H), 1.48 (dq, *J* = 19.4, 7.9, 7.3 Hz, 6H), 1.24 (d, *J* = 8.2 Hz, 11H), 1.06 (d, *J* = 6.6 Hz, 3H), 0.84 (dd, *J* = 18.9, 6.3 Hz, 6H). ^{13}C NMR (DMSO-d_6, 126 MHz) δ 173.7, 172.1, 168.3, 167.3, 166.7, 164.4, 55.1, 53.7, 53.2, 51.1, 50.2, 47.0, 44.0, 42.4, 40.4, 38.8, 31.5, 29.9, 28.3, 27.0, 24.2, 23.2, 22.7, 21.9, 21.7, 16.2.

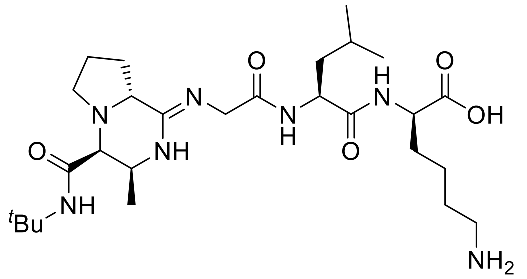

am*[PGLK] (23D[a])

The peptide was cyclized at 0.15 mmol scale with telescopic aziridine ring-opening. After cyclization, filtration, and deprotection 64.5 mg of peptide was recovered. Following purification by RP-HPLC SP 5 to 15, the pure fractions were pooled to afford 8.1 mg of the title compound in 9.8% overall yield. ESI MS $[M+H]^+$ expected: 552.4, experimental: 552.4; $[M+2H]^{2+}$ expected: 276.7, experimental: 276.6. ESI MS $[M+H]^+$ expected: 552.3868, experimental: 552.3862. LC/MS retention time 3.848 min. ^{1}H NMR (DMSO-d_6, 700 MHz) δ 8.52 (s, 1H), 7.64 (s, 1H), 7.56 – 7.47 (m, 1H), 4.17 (q, J = 7.6 Hz, 1H), 4.04 (d, J = 17.6 Hz, 1H), 3.96 (d, J = 14.0 Hz, 2H), 3.88 – 3.79 (m, 2H), 3.29 (d, J = 7.5 Hz, 1H), 2.94 (d, J = 7.4 Hz, 1H), 2.76 – 2.65 (m, 3H), 2.34 (d, J = 14.6 Hz, 1H), 1.80 – 1.75 (m, 1H), 1.65 (m, 4H), 1.50 (m, 5H), 1.25 (d, J = 2.9 Hz, 11H), 1.22 – 1.17 (m, 3H), 0.91 – 0.80 (m, 6H). ^{13}C NMR (DMSO-d_6, 126 MHz) δ 174.2, 171.4, 169.3, 168.5, 167.5, 61.0, 54.8, 53.5, 52.6, 52.3, 50.2, 43.9, 40.7, 38.7, 31.9, 30.8, 28.4, 26.9, 24.3, 23.2, 23.2, 22.3, 21.4, 16.3.

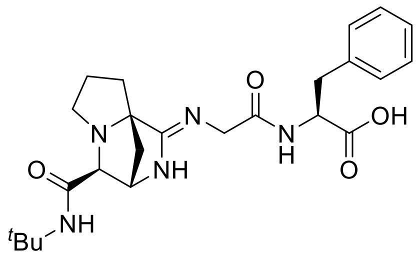

br[PGF] (24C)

The peptide was cyclized at 0.45 mmol scale. Following purification by flash chromatography (EtOAc→MeOH), the pure fractions were pooled to afford 15.7 mg of the title compound in 8% overall yield. ESI MS $[M+H]^+$ expected: 456.4, experimental: 456.4. ^{1}H NMR (DMSO-d_6, 500 MHz) δ 8.07 – 7.82 (m, 2H), 7.50 (s, 1H), 7.22 – 7.09 (m, 5H), 4.23 (q, J = 6.4 Hz, 1H), 3.95 (s, 1H), 3.74 – 3.52 (m, 2H), 3.06 (dd, J = 13.5, 5.1 Hz, 1H), 2.92 (dd, J = 13.5, 6.5 Hz, 1H), 2.79 (s, 1H), 2.66 (dt, J = 8.2, 6.3 Hz, 1H), 2.40 – 2.26 (m, 1H), 2.17 – 1.96 (m, 3H), 1.82 (d, J = 8.9 Hz, 1H), 1.73 – 1.60 (m, 1H), 1.55 (dd, J = 9.2, 1.9 Hz, 1H), 1.26 (s, 9H). ^{13}C NMR (DMSO-d_6, 126 MHz) δ 173.4, 168.6, 168.1, 165.1, 138.5, 129.4, 127.8, 125.8, 76.6, 66.1, 63.8, 54.6, 50.1, 48.2, 44.5, 40.7, 37.4, 35.8, 28.4, 26.8, 20.4.

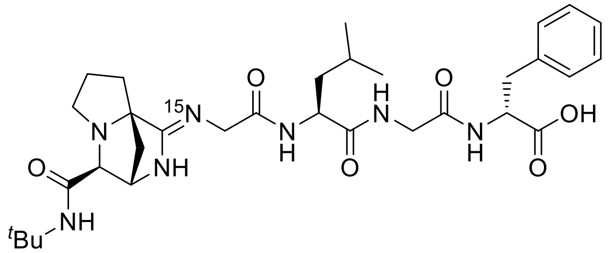

br[P^{15N}GLGf] – ^{15}N-labelled (25C)

The peptide was cyclized at 0.06 mmol scale and ring-opened with thiobenzoic acid (0.13 mmol) for one hour prior to solvent evaporation. Following purification by RP-HPLC SP 20 to 40, the pure fractions were pooled to afford 5.8 mg of the title compound in 15% overall yield. ESI-TOF MS $[M+H]^+$ expected: 627.3631, experimental: 627.3635. ^{1}H NMR (DMSO-d_6, 700 MHz) δ 8.80 (s, 1H), 8.22 (d, J = 9.0 Hz, 1H), 7.54 (s, 1H), 7.26 (d, J = 5.5 Hz, 1H), 7.22 – 7.05 (m, 5H), 4.49 – 4.39 (m, 1H), 4.16 (s, 1H), 3.93 – 3.75 (m, 3H), 3.63 (dd, J = 16.6, 6.0 Hz, 1H), 3.47 – 3.36 (m, 1H),

3.06 (ddd, J = 43.5, 13.2, 4.7 Hz, 2H), 2.80 – 2.70 (m, 2H), 2.60 (d, J = 9.6 Hz, 1H), 2.14 (d, J = 9.2 Hz, 3H), 1.89 (d, J = 9.3 Hz, 1H), 1.82 (d, J = 11.0 Hz, 1H), 1.71 (d, J = 9.3 Hz, 1H), 1.51 – 1.33 (m, 2H), 1.30 (s, 10H), 0.84 (dd, J = 21.2, 6.5 Hz, 6H). ^{1}H NMR (DMSO-d_6 + formic acid, 700 MHz) δ 8.42 (t, J = 5.9 Hz, 1H), 8.31 (d, J = 8.4 Hz, 1H), 7.78 (d, J = 7.0 Hz, 1H), 7.66 (s, 1H), 7.27 – 7.12 (m, 5H), 4.35 (td, J = 8.9, 5.7 Hz, 1H), 4.27 – 4.19 (m, 2H), 3.99 – 3.89 (m, 2H), 3.61 (qd, J = 16.7, 5.9 Hz, 2H), 3.05 (dd, J = 13.6, 5.2 Hz, 1H), 3.00 (s, 1H), 2.91 (dd, J = 13.6, 7.3 Hz, 1H), 2.75 – 2.63 (m, 2H), 2.22 – 2.15 (m, 1H), 2.14 – 2.01 (m, 2H), 1.92 – 1.81 (m, 1H), 1.75 (dd, J = 9.7, 2.0 Hz, 1H), 1.52 (dt, J = 13.4, 6.9 Hz, 1H), 1.46 – 1.34 (m, 2H), 1.26 (s, 10H), 0.83 (dd, J = 26.2, 6.6 Hz, 6H). ^{15}N NMR (DMSO-d_6 + formic acid, 500 MHz) δ 102.5.

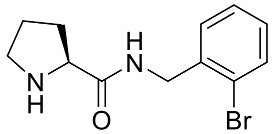

(S)-N-(4-bromobenzyl)pyrrolidine-2-carboxamide (26)

Fmoc-Pro-OH (1.43 g, 4.0 mmol) in a 50 mL RB flask was dissolved in THF (20 mL) and triethylamine (0.56 mL, 4.0 mmol) was added. The reaction was cooled by stirring over ice and then ethylchloroformate (0.38 mL, 4.0 mmol) was added dropwise over 15 minutes. Afterwards, the mixture was left to stir for 25 minutes and then 2-bromobenzylamine was added (0.51 mL, 4.0 mmol). The reaction was allowed to stir for an additional thirty minutes on ice and then left to stir at room temperature for 16 hours. The reaction was then diluted with EtOAc and filtered. After evaporation of solvent the mixture was purified by flash column chromatography (Hex → EtOAc). Rf (DCM) = 0.33. Pure fractions were concentrated to a white powder which was immediately dissolved in MeCN (40 mL) and piperidine (0.60 mL) was added. The reaction was allowed to stir at room temperature for two hours before being evaporated and purified by flash column chromatography (Hex → EtOAc → MeOH, all buffered with 1% triethylamine). The final compound was isolated as a yellow oil in 62% overall yield. ESI-TOF MS $[M+H]^+$ expected: 283.0441, experimental: 283.0451. ^{1}H NMR (DMSO-d_6, 500 MHz) δ 8.45 (t, J = 6.2 Hz, 1H), 7.58 (dd, J = 7.9, 1.3 Hz, 1H), 7.34 (td, J = 7.5, 1.3 Hz, 1H), 7.22 (dd, J = 7.7, 1.7 Hz, 1H), 7.18 (td, J = 7.6, 1.8 Hz, 1H), 4.28 (d, J = 6.2 Hz, 2H), 3.59 (dd, J = 8.8, 5.5 Hz, 1H), 2.85 (dt, J = 10.1, 6.7 Hz, 1H), 2.79 (dt, J = 10.1, 6.4 Hz, 1H), 1.96 (ddt, J = 12.2, 8.5, 7.0 Hz, 1H), 1.70 (dtd, J = 12.4, 7.1, 5.5 Hz, 1H), 1.64 – 1.56 (m, 2H). ^{13}C NMR (DMSO-d_6, 126 MHz) δ 174.7, 138.0, 132.3, 128.8, 128.4, 127.7, 122.3, 60.3, 46.8, 42.3, 30.5, 25.8.

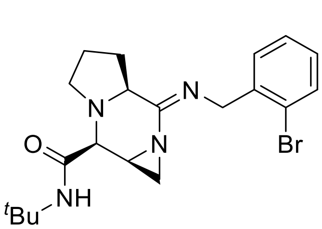

(3aS,8S,8aS,Z)-3-((2-bromobenzyl)imino)-N-(tert-butyl)hexahydro-1H,3H-azirino[1,2-a]pyrrolo[1,2-d]pyrazine-8-carboxamide (27)

In a two-dram vial equipped with a magnetic stirring bar were added **26** (50 mg, 0.176 mmol), **3** (530 µL, 0.2 M solution in TFE, 0.106 mmol), DCM (1000 µL) and ^{t}BuNC (39.8 µL, 0.36 mmol)

and stirred for five hours. The mixture was then concentrated by N_2 purge and purified by silica gel chromatography (Hex → EtOAc). Pure fractions were concentrated to an off-white powder (7.0 mg, 10% yield). ESI-TOF MS $[M+H]^+$ expected: 419.1441, experimental: 419.1441. ^1H NMR ($CDCl_3$, 500 MHz) δ 7.63 (ddt, *J* = 7.7, 1.7, 0.9 Hz, 1H), 7.54 (dd, *J* = 8.0, 1.3 Hz, 1H), 7.31 (td, *J* = 7.5, 1.3 Hz, 1H), 7.14 – 7.06 (m, 1H), 6.28 (s, 1H), 4.76 (d, *J* = 17.5 Hz, 1H), 4.58 (d, *J* = 18.1 Hz, 1H), 3.34 (d, *J* = 5.6 Hz, 1H), 3.16 (dt, *J* = 9.9, 5.1 Hz, 1H), 2.95 – 2.92 (m, 1H), 2.87 (ddt, *J* = 9.1, 7.4, 1.5 Hz, 1H), 2.34 (dd, *J* = 5.1, 1.0 Hz, 1H), 2.29 – 2.12 (m, 2H), 2.03 – 1.95 (m, 1H), 1.87 – 1.79 (m, 3H), 1.34 (s, 9H). ^{13}C NMR ($CDCl_3$, 126 MHz) δ 169.5, 163.0, 140.0, 132.4, 129.3, 128.0, 127.4, 123.6, 65.6, 62.4, 54.5, 51.4, 51.1, 37.2, 29.9, 29.0, 24.3, 21.5.

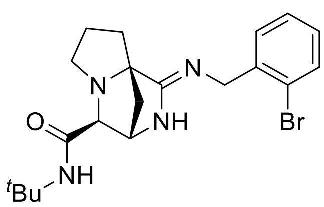

(3S,4S,8aS,Z)-1-((2-bromobenzyl)imino)-N-(tert-butyl)hexahydro-6H-3,8a-methanopyrrolo[1,2-a]pyrazine-4-carboxamide (28)

In a two-dram vial equipped with a magnetic stirring bar were added **26** (25 mg, 0.088 mmol), **3** (265 µL, 0.2 M solution in TFE, 0.053 mmol), HFIP (500 µL) and ^{t}BuNC (19.9 µL, 0.18 mmol) and stirred for five hours. The mixture was then concentrated by N_2 purge and purified by silica gel chromatography (Hex → EtOAc → MeOH). Pure fractions were concentrated to an off-white powder (9.9 mg, 27% yield). Crystals of **28** formed spontaneously upon concentration of pure fractions from Hex/EtOAc. ESI-TOF MS $[M+H]^+$ expected: 419.1441, experimental: 419.1435. ^1H NMR ($CDCl_3$ + 0.3% formic acid, 700 MHz) δ 7.58 – 7.56 (m, 1H), 7.45 – 7.43 (m, 1H), 7.32 (td, *J* = 7.5, 1.2 Hz, 1H), 7.22 (td, *J* = 7.7, 1.6 Hz, 1H), 7.17 – 7.12 (m, 1H), 4.72 – 4.60 (m, 2H), 4.51 (s, 1H), 3.43 – 3.38 (m, 1H), 3.06 – 3.00 (m, 1H), 2.71 (d, *J* = 8.1 Hz, 1H), 2.44 – 2.37 (m, 1H), 2.35 – 2.28 (m, 2H), 2.22 (d, *J* = 11.5 Hz, 1H), 2.04 – 1.97 (m, 2H), 1.35 (s, 9H). ^{13}C NMR ($CDCl_3$ + 0.3% formic acid, 126 MHz) δ 168.0, 167.3, 133.3, 131.3, 130.7, 128.3, 124.0, 79.5, 65.7, 65.2, 52.0, 48.3, 45.3, 43.0, 28.6, 26.7, 20.9.

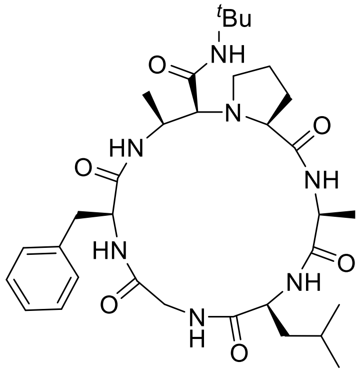

c*[PALGF] (S1)

The peptide was cyclized at 0.10 mmol scale with telescopic aziridine ring-opening. Following purification by RP-HPLC SP 20 to 60, the pure fractions were pooled to afford 14.8 mg of the

title compound in 22% overall yield ESI MS [M+H]+ expected: 642.4, experimental: 642.4. ESI-TOF MS $[M+H]^+$ expected: 642.3974, experimental: 642.3974. LC/MS retention time 6.550 min. ^{1}H NMR (DMSO-d_6, 500 MHz) δ 8.87 (t, *J* = 4.9 Hz, 1H), 8.45 (d, *J* = 8.4 Hz, 1H), 7.83 (d, *J* = 7.7 Hz, 1H), 7.47 (d, *J* = 8.6 Hz, 1H), 7.41 (d, *J* = 7.5 Hz, 1H), 7.28-7.17 (m, 5H), 6.58 (s, 1H), 4.27-4.20 (m, 3H), 4.15 (ddt, *J* = 15.6, 8.7, 5.7 Hz, 1H), 3.68 (dd, *J* = 15.3, 4.3 Hz, 1H), 3.41 (dd, *J* = 15.1, 5.6 Hz, 1H), 3.36 (m, 1H), 3.29 (m, 1H), 3.18 (t, *J* = 7.4 Hz, 1H), 2.96 (dt, *J* = 7.8, 6.2 Hz, 1H), 2.82-2.77 (m, 2H), 1.97 (m, 1H), 1.73 (m, 1H), 1.67 (m, 2H), 1.58 (t, *J* = 6.6 Hz, 2H), 1.52 (m, 1H), 1.30 (d, *J* = 5.0 Hz, 3H), 1.28 (d, *J* = 5.3 Hz, 3H), 1.22 (s, 9H), 0.89 (t, *J* = 6.3 Hz, 6H). ^{13}C NMR (DMSO-d_6, 126 MHz) δ 173.8, 172.9, 172.3, 170.2, 169.5, 168.4, 138.5, 128.8, 128.3, 126.2, 66.2, 65.2, 54.9, 50.7, 50.5, 49.6, 46.9, 45.3, 43.7, 41.0, 35.4, 30.0, 28.2, 24.3, 23.5, 23.1, 22.0, 18.4, 17.8.

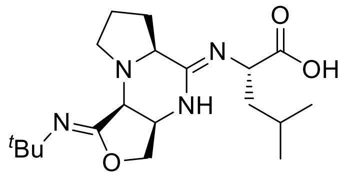

S9

The dipeptide was cyclized at 9.4 mmol scale. Following purification by flash chromatography (EtOAc→MeOH), the pure fractions were pooled to afford 725 mg of the title compound in 21% overall yield. ESI MS $[M+H]^+$ expected: 365.3, experimental: 365.3. ^{1}H NMR (DMSO-d_6, 500 MHz) δ 4.48 (t, *J* = 7.7 Hz, 1H), 4.16 – 4.06 (m, 1H), 3.99 – 3.92 (m, 1H), 3.70 (dd, *J* = 9.4, 7.7 Hz, 1H), 3.25 – 3.17 (m, 1H), 2.98 (d, *J* = 5.9 Hz, 1H), 2.84 – 2.76 (m, 1H), 2.25 (tt, *J* = 6.8, 4.9 Hz, 1H), 2.20 (s, 1H), 1.81 – 1.69 (m, 2H), 1.68 – 1.48 (m, 4H), 1.28 – 1.21 (m, 1H), 1.18 (d, *J* = 5.9 Hz, 9H), 0.90 – 0.82 (m, 6H). ^{13}C NMR (DMSO-d_6, 126 MHz) δ 172.7, 160.7, 156.0, 74.0, 61.4, 59.2, 55.1, 54.0, 53.0, 50.3, 39.2, 28.3, 25.6, 24.5, 22.9, 21.9, 20.3.

References

1. S. Zaretsky, S. Adachi, B. H. Rotstein, J. L. Hickey, C. C. G. Scully, J. D. St Denis, R. Courtemanche, J. C. Y. Yu, B. K. W. Chung, and A. K. Yudin, *J. Org. Chem.*, 2014, **79**, 9948–9957.

2. B. Rotstein and A. Yudin, *Synthesis*, 2012, **44**, 2851–2858.

3. B. H. Rotstein, V. Rai, R. Hili, and A. K. Yudin, *Nat. Protoc.*, 2010, **5**, 1813–22.

4. R. Hili and A. K. Yudin, *J. Am. Chem. Soc.*, 2006, **128**, 14772–3.

5. S. Zaretsky, C. C. G. Scully, A. J. Lough, and A. K. Yudin, *Chem. Eur. J.*, 2013, **19**, 17668–72.

6. R. Hili, V. Rai, and A. K. Yudin, *J. Am. Chem. Soc.*, 2010, **132**, 2889–91.

NMR Spectra

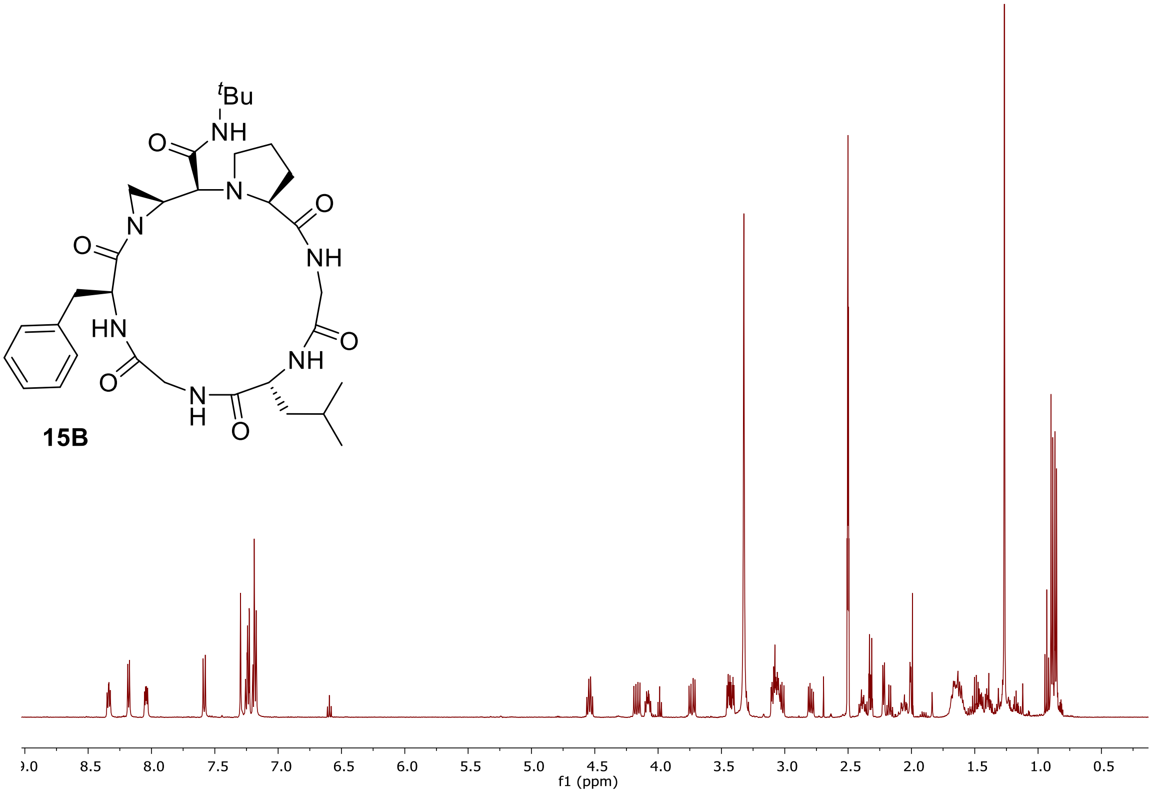

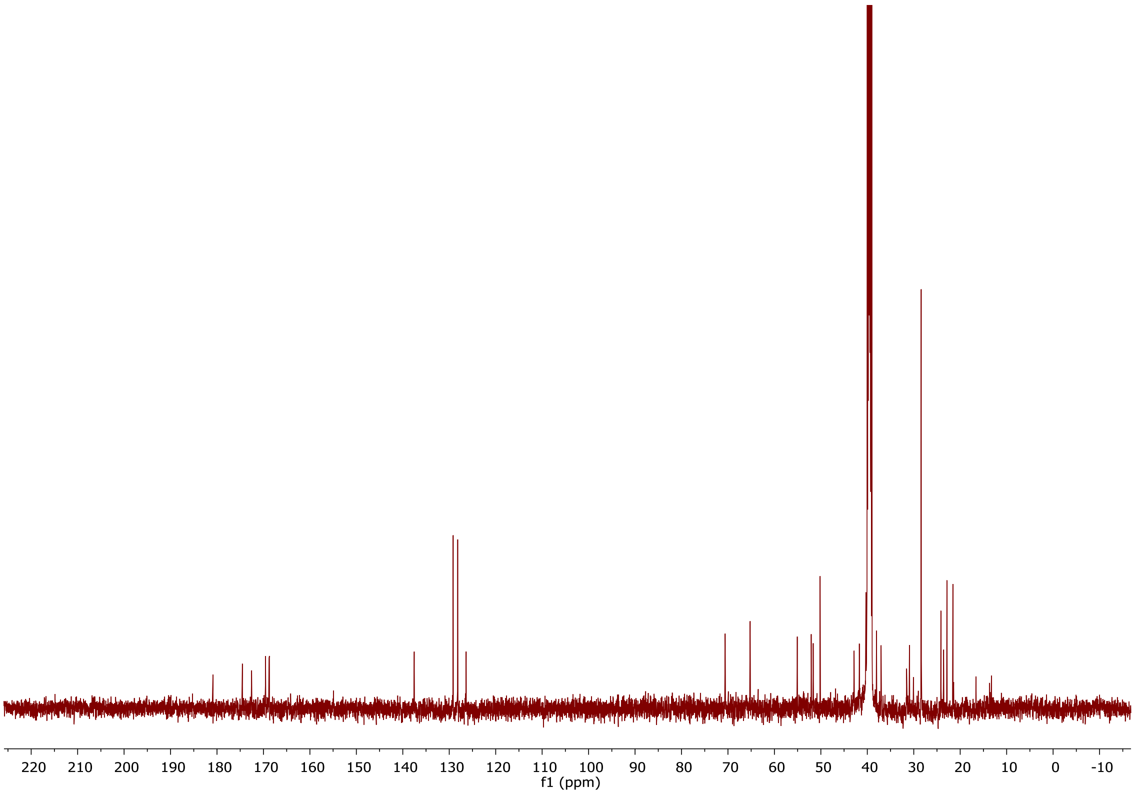

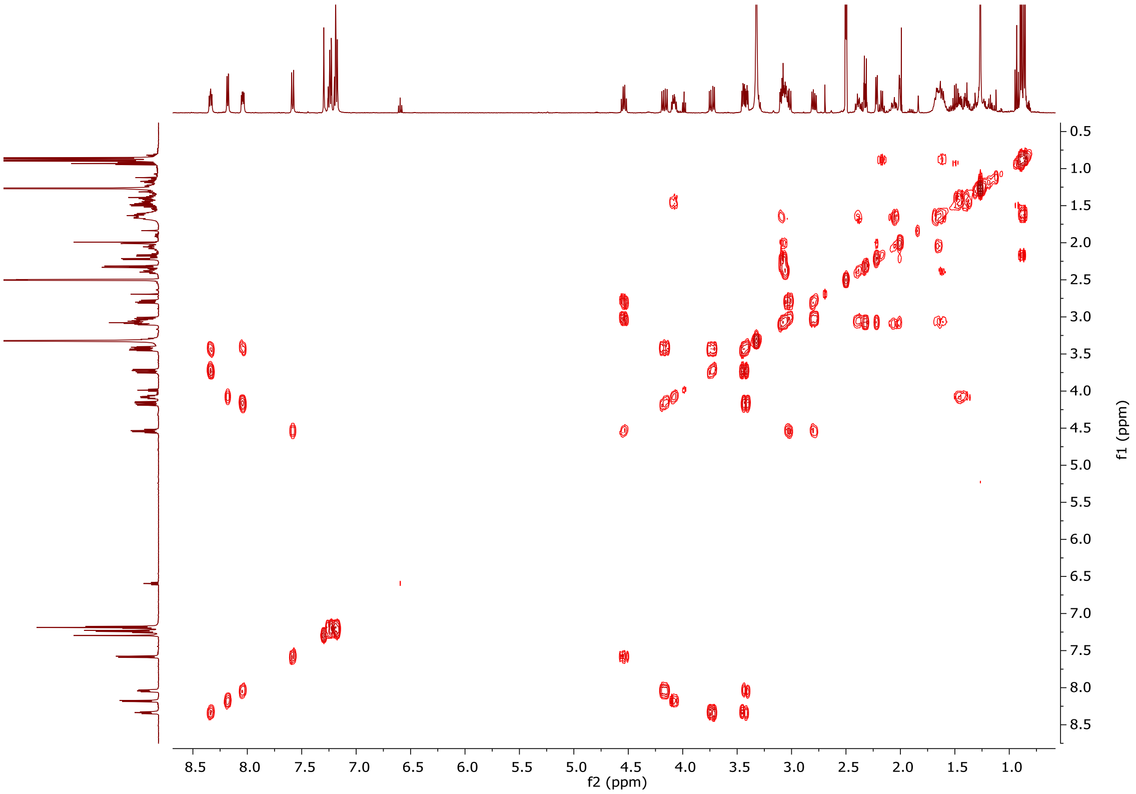

COSY (DMSO-*d6*, 25 ^{o}C)

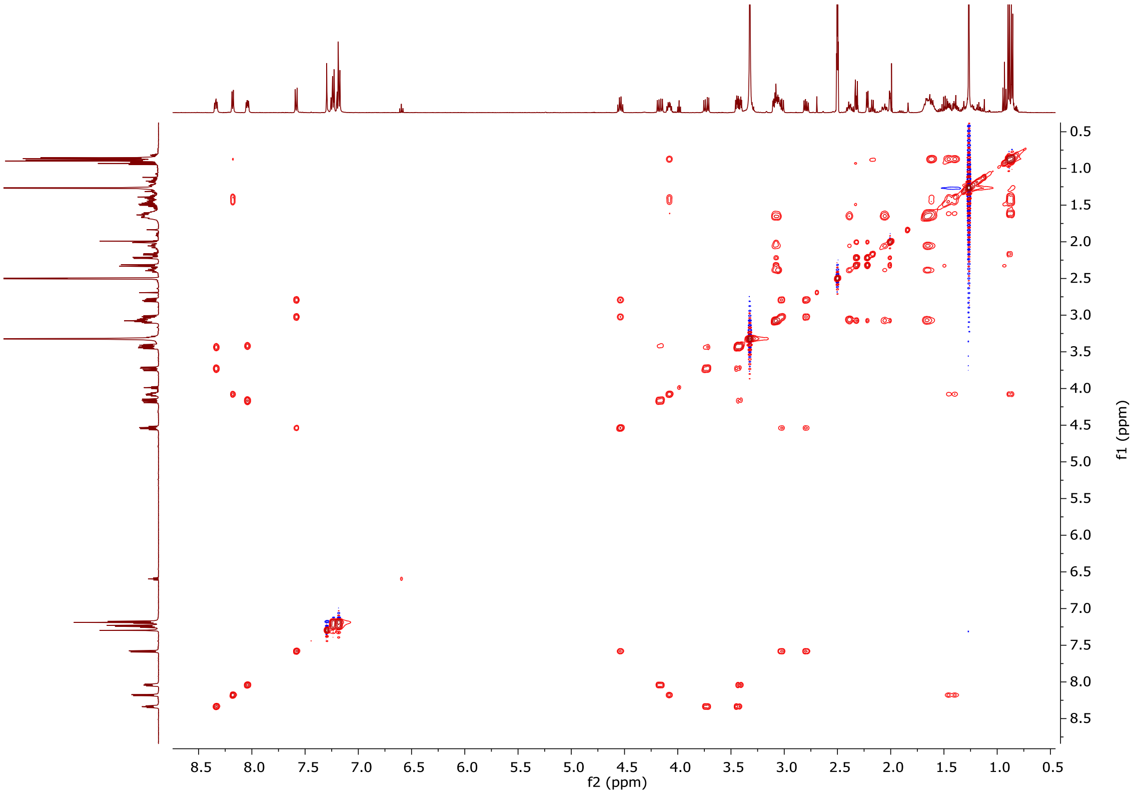

TOCSY (DMSO-*d6*, 25 ^{o}C)

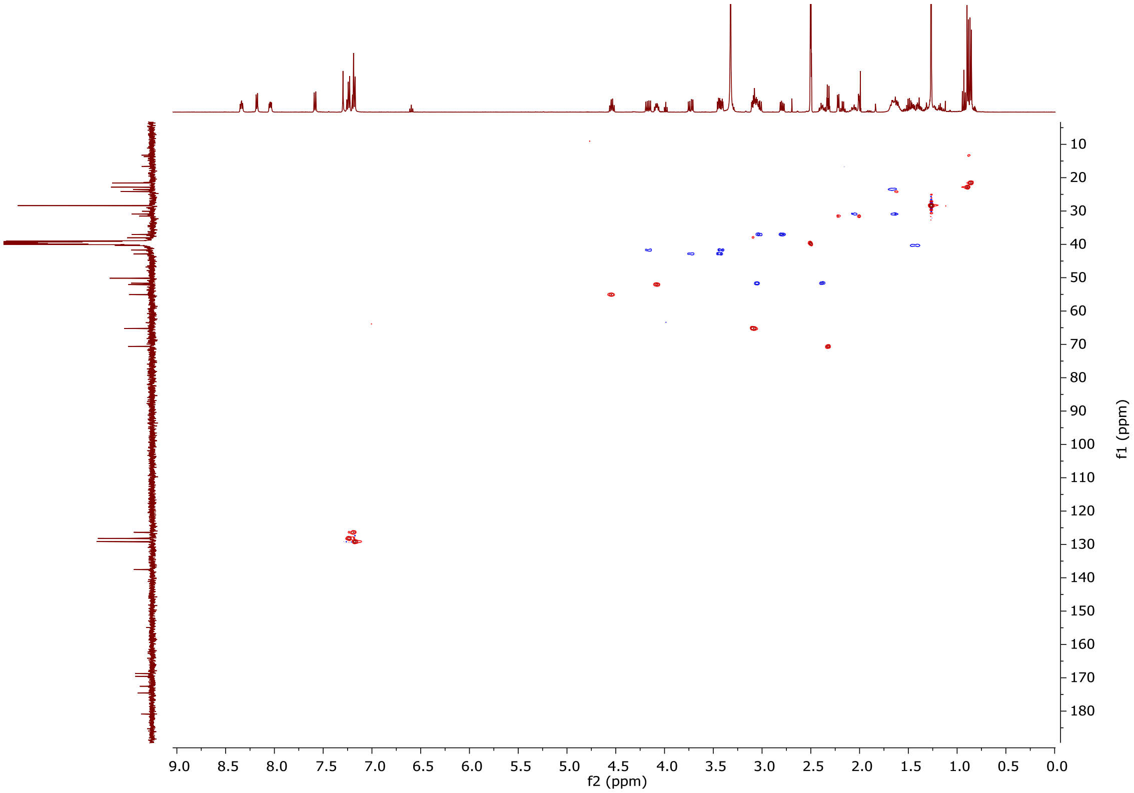

HSQC (DMSO-*d6*, 25 °C)

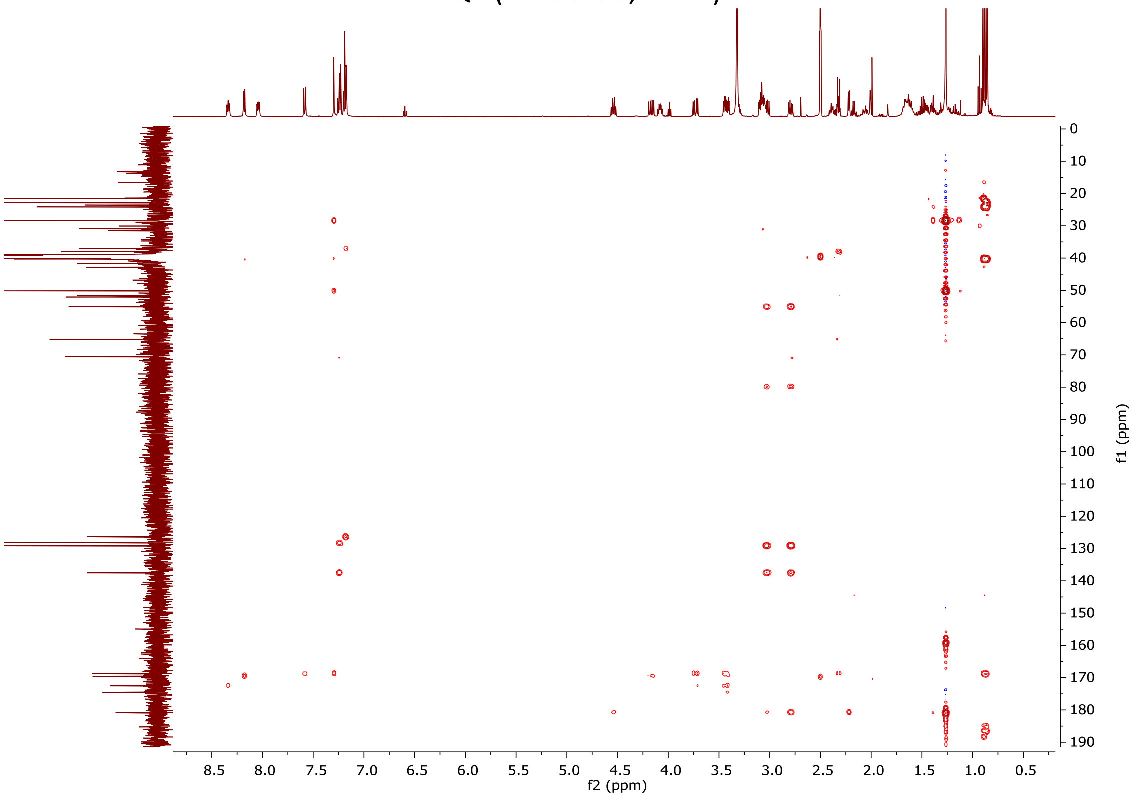

HMBC (DMSO-*d6*, 25 °C)

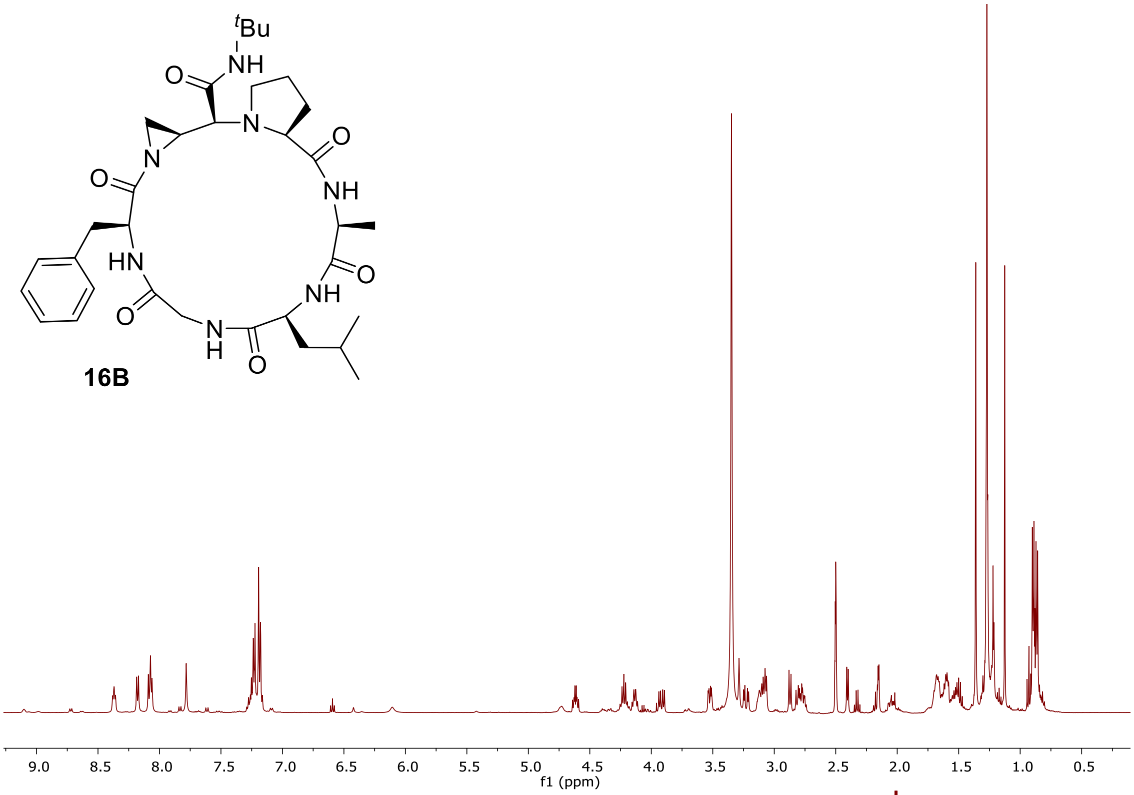

^{t}Bu
NH
O
N
O
N
O
NH
HN
O
NH
O
N
H
O
16B
9.0 8.5 8.0 7.5 7.0 6.5 6.0 5.5 5.0 4.5 4.0 3.5 3.0 2.5 2.0 1.5 1.0 0.5
f1 (ppm)

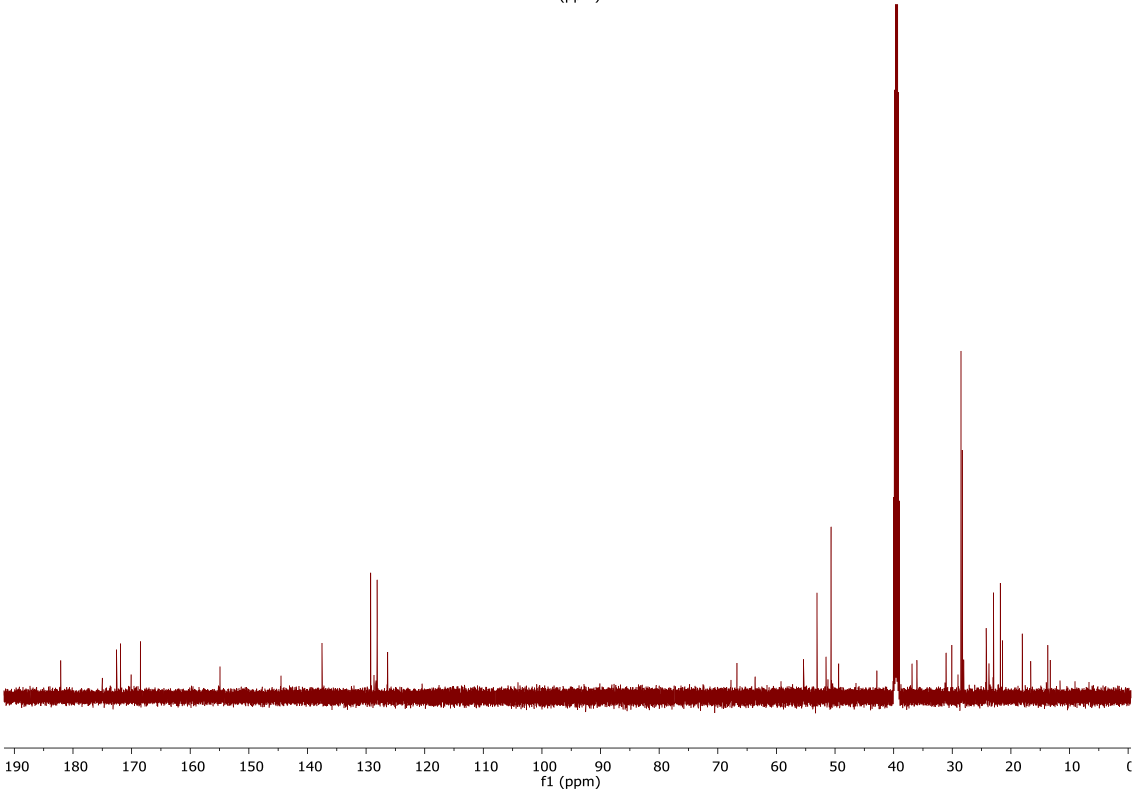

190 180 170 160 150 140 130 120 110 100 90 80 70 60 50 40 30 20 10
f1 (ppm)

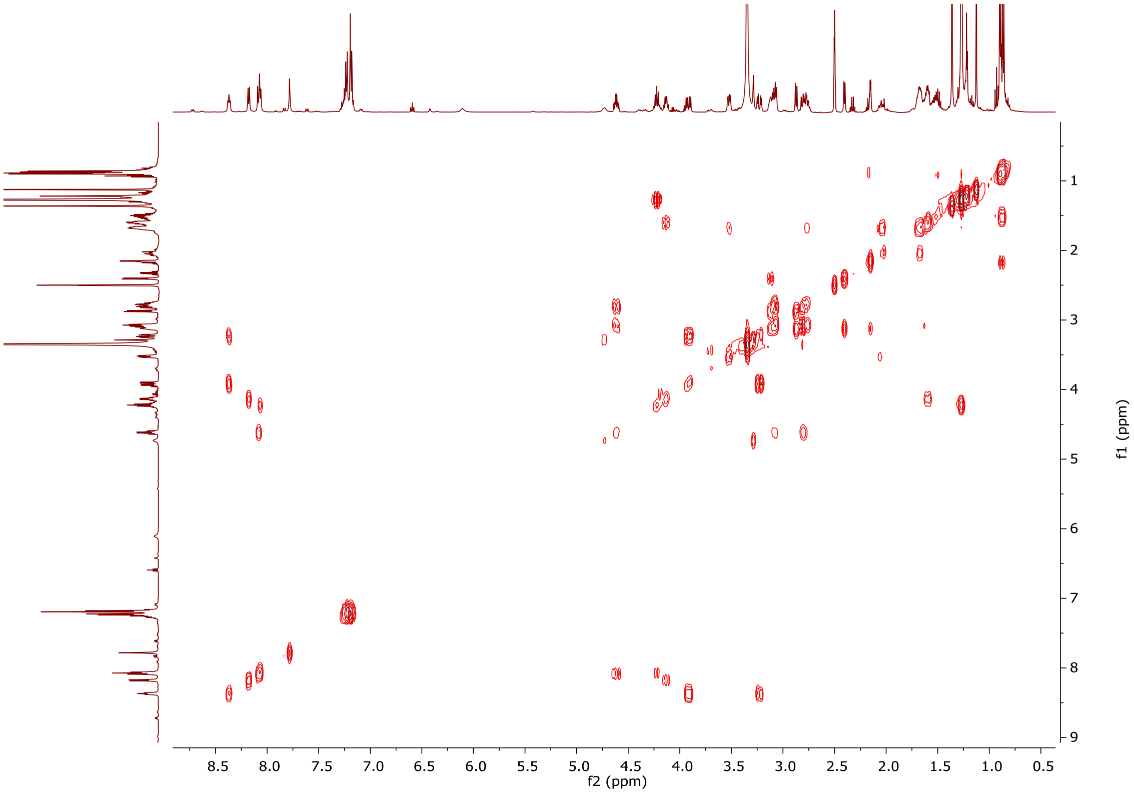

COSY (DMSO-*d6*, 25 ^{o}C)

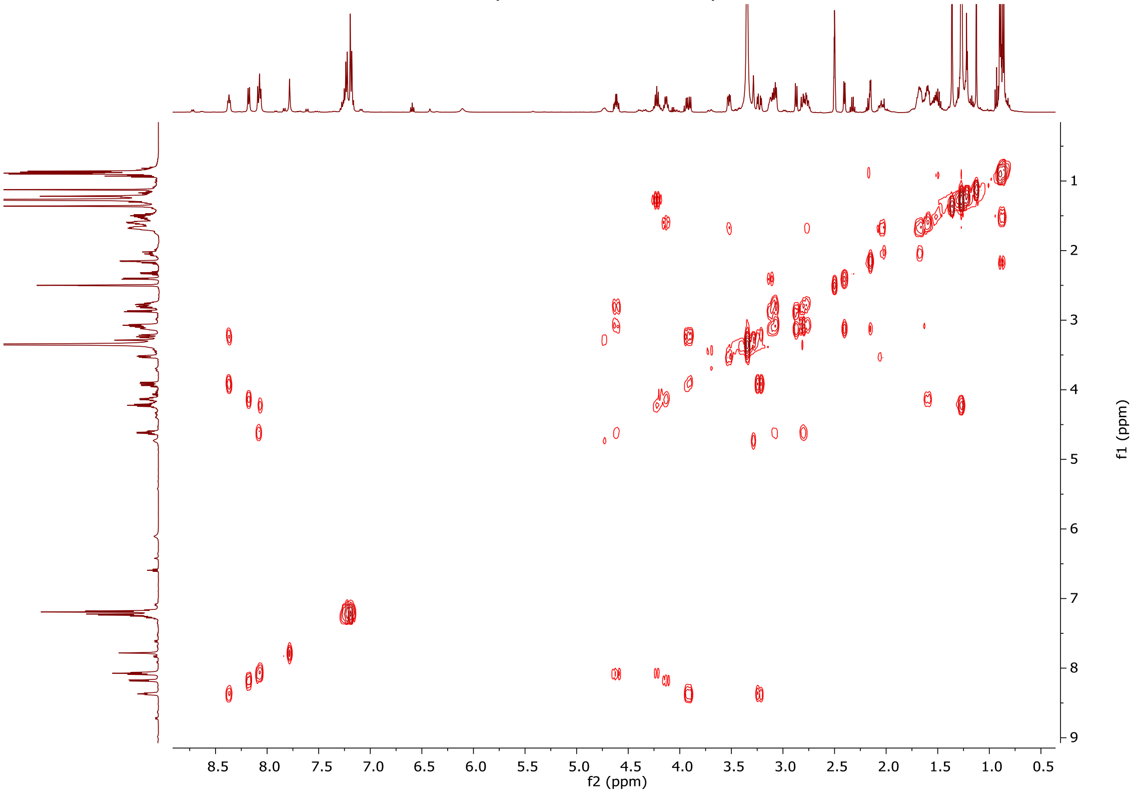

TOCSY (DMSO-*d6*, 25 ^{o}C)

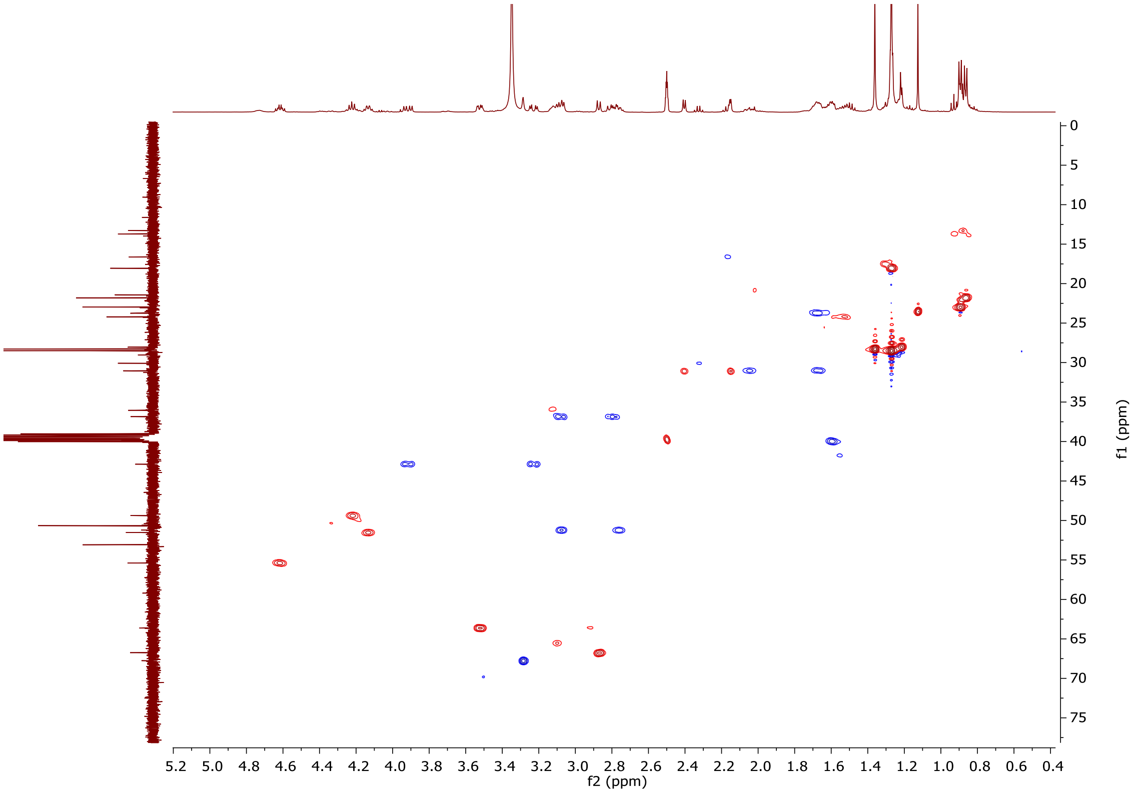

HSQC (DMSO-*d6*, 25 °C)

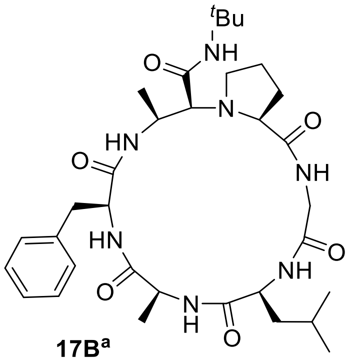

17B[a]

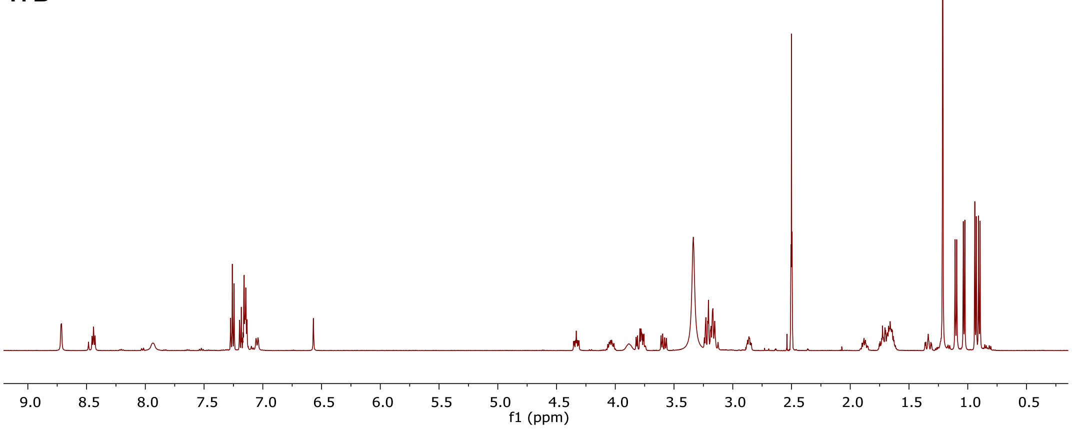

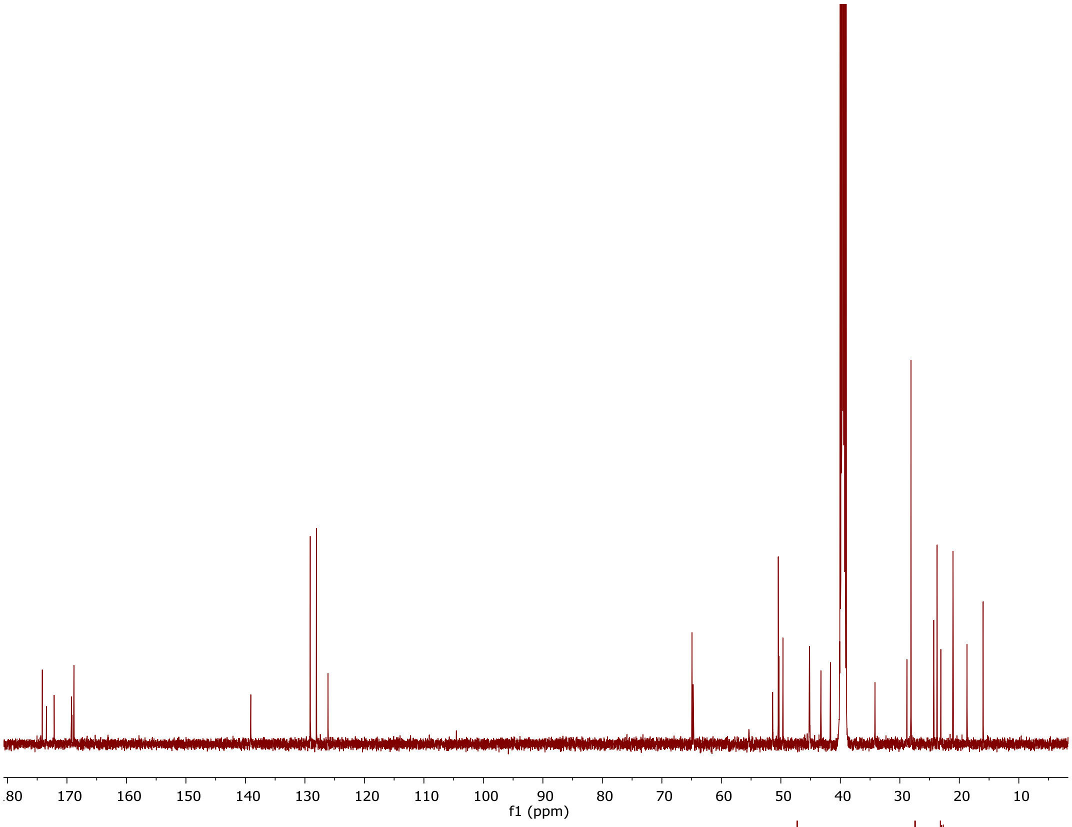

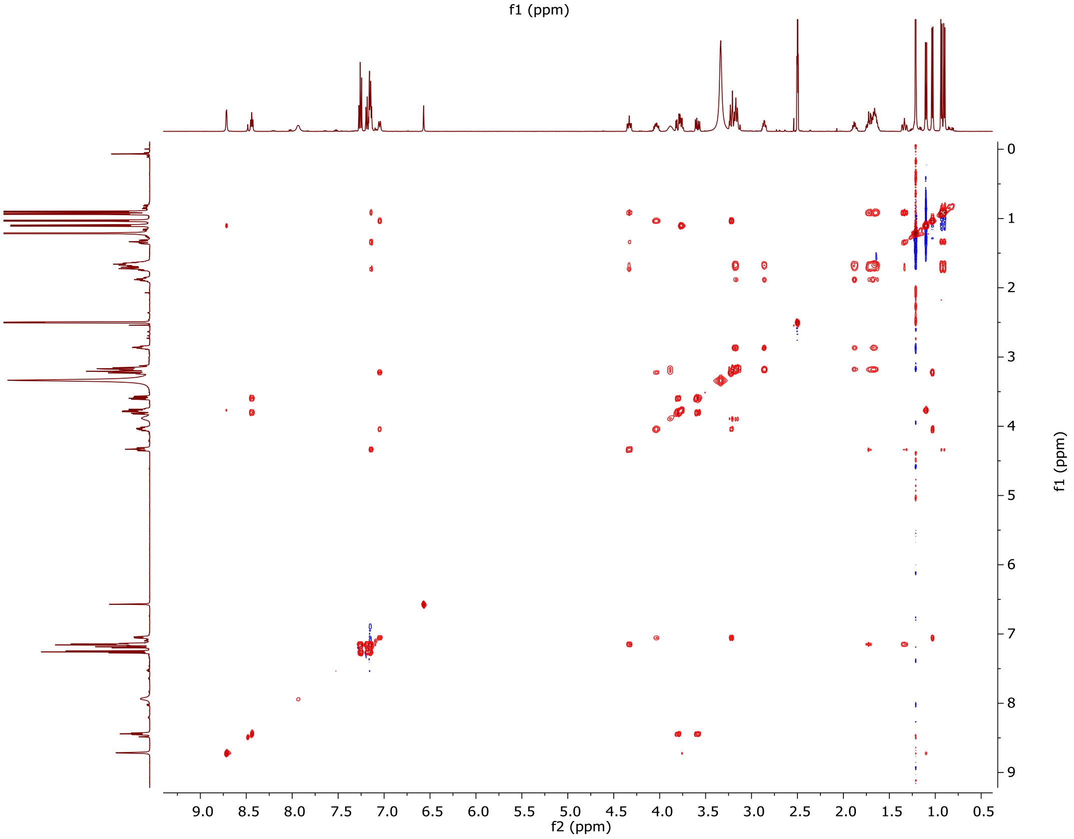

TOCSY (DMSO-*d6*, 25 °C)

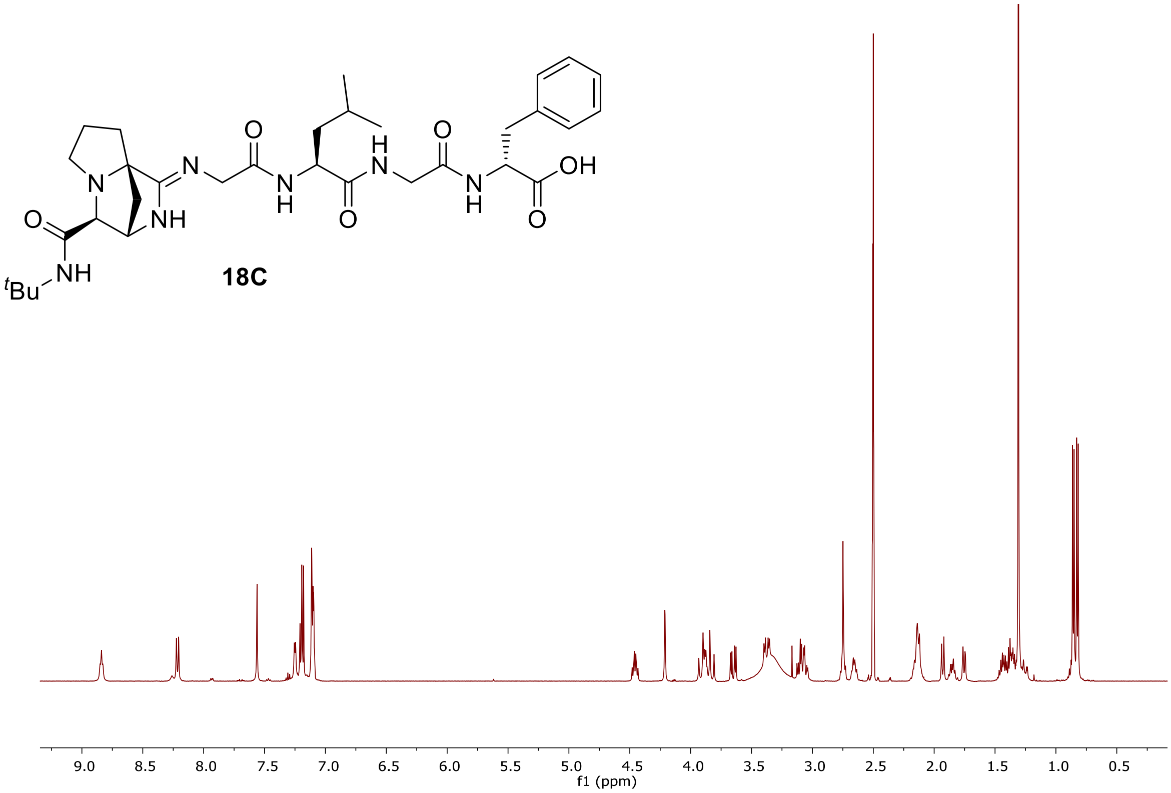
O
N
N
NH
NH
tBu
O
N
H
O
H
N
O
N
H
OH
O
9.0 8.5 8.0 7.5 7.0 6.5 6.0 5.5 5.0 4.5 4.0 3.5 3.0 2.5 2.0 1.5 1.0 0.5
f1 (ppm)

18C

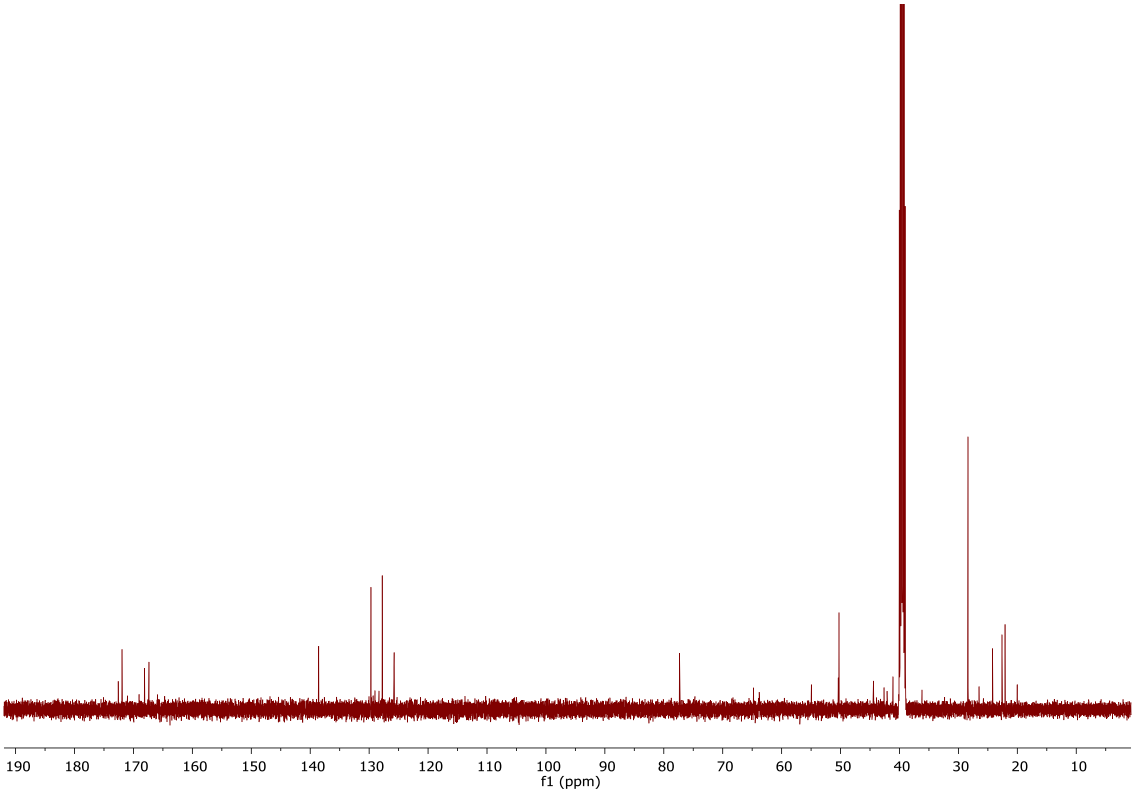
190 180 170 160 150 140 130 120 110 100 90 80 70 60 50 40 30 20 10
f1 (ppm)

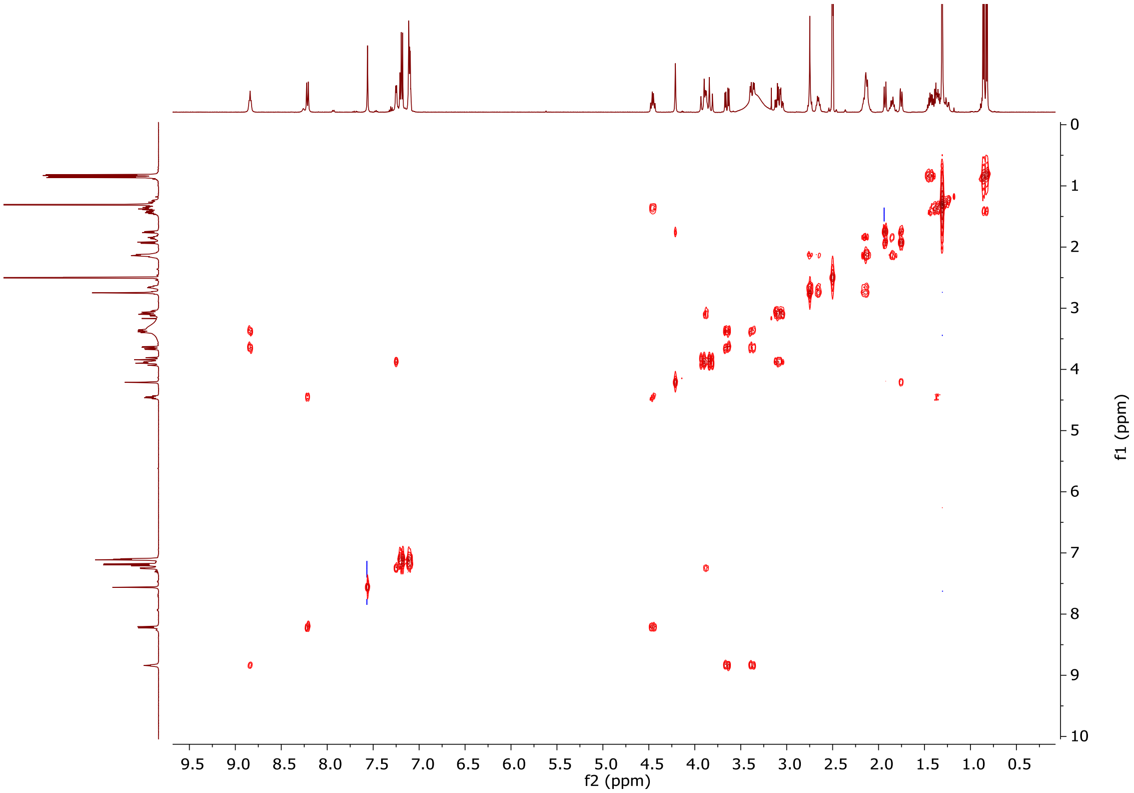

COSY (DMSO-*d6*, 25 °C)

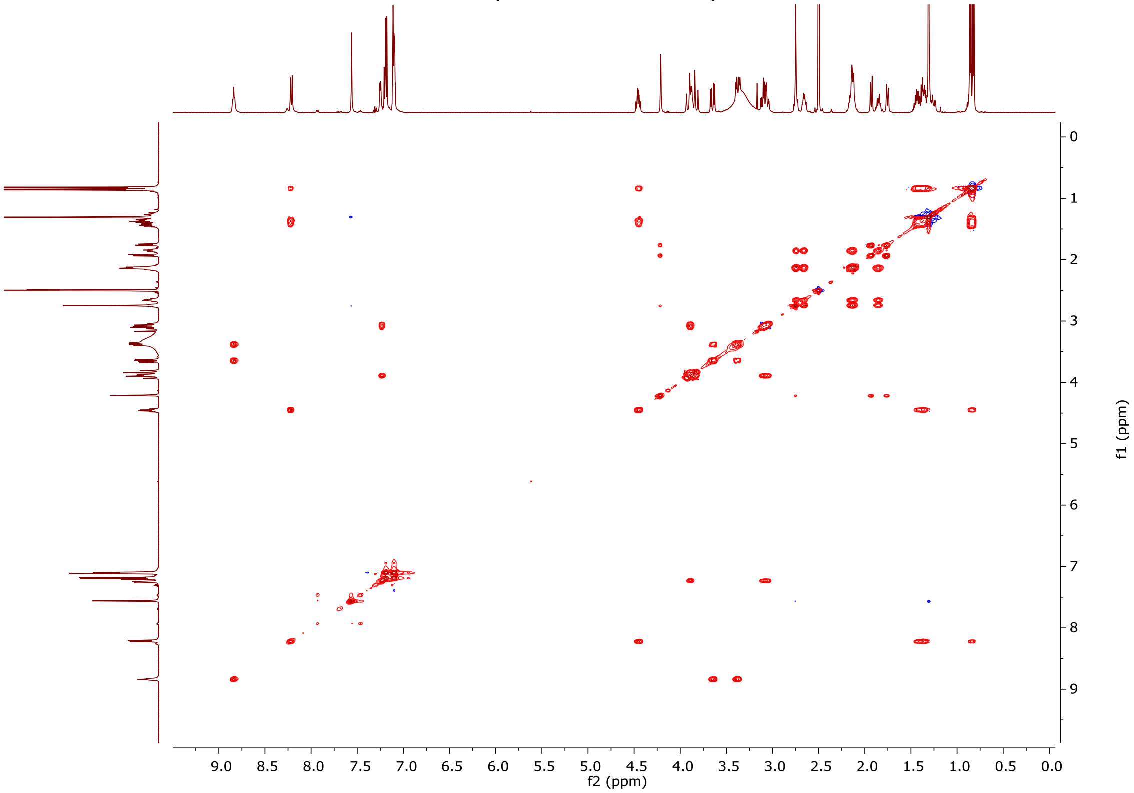

TOCSY (DMSO-*d6*, 25 °C)

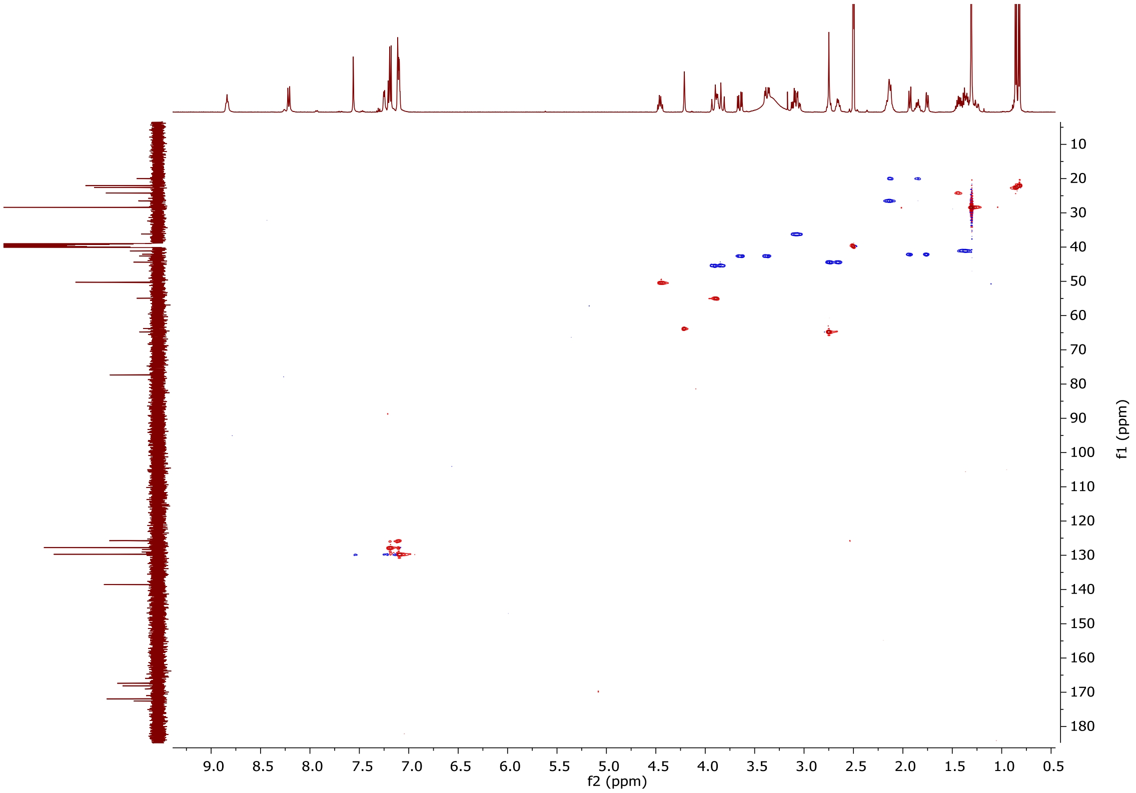

HSQC (DMSO-*d6*, 25 ^{o}C)

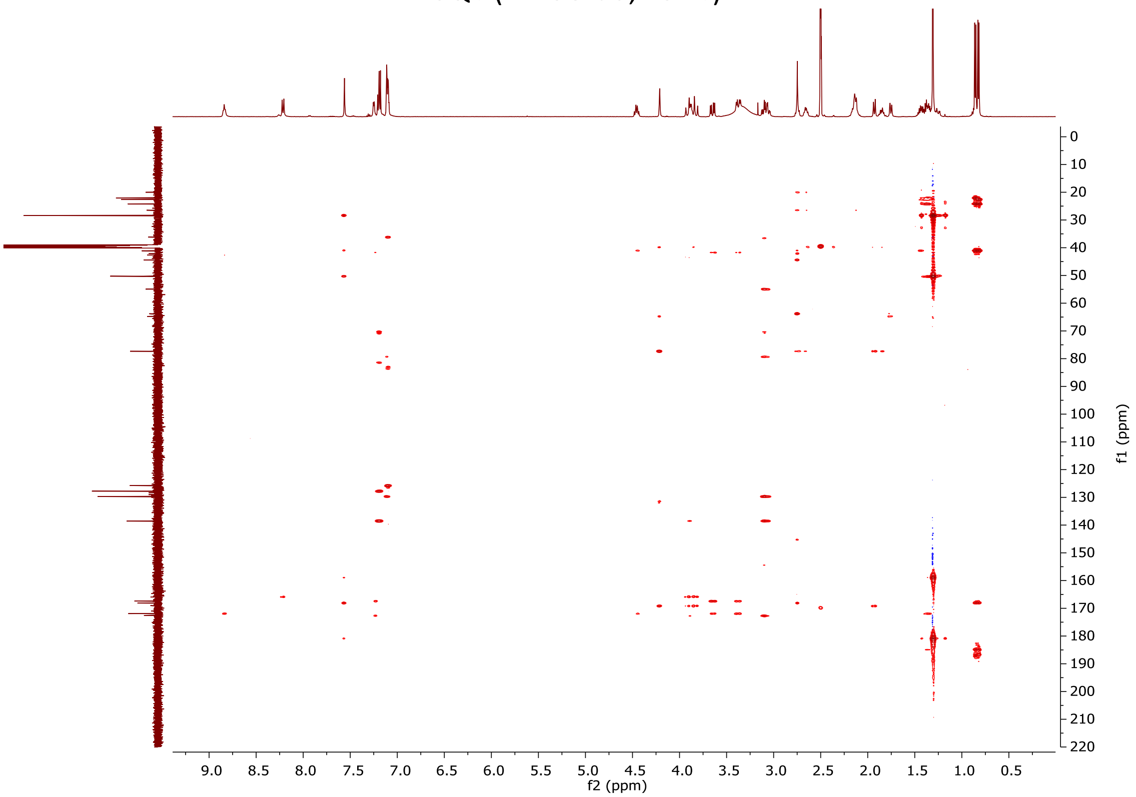

HMBC (DMSO-*d6*, 25 ^{o}C)

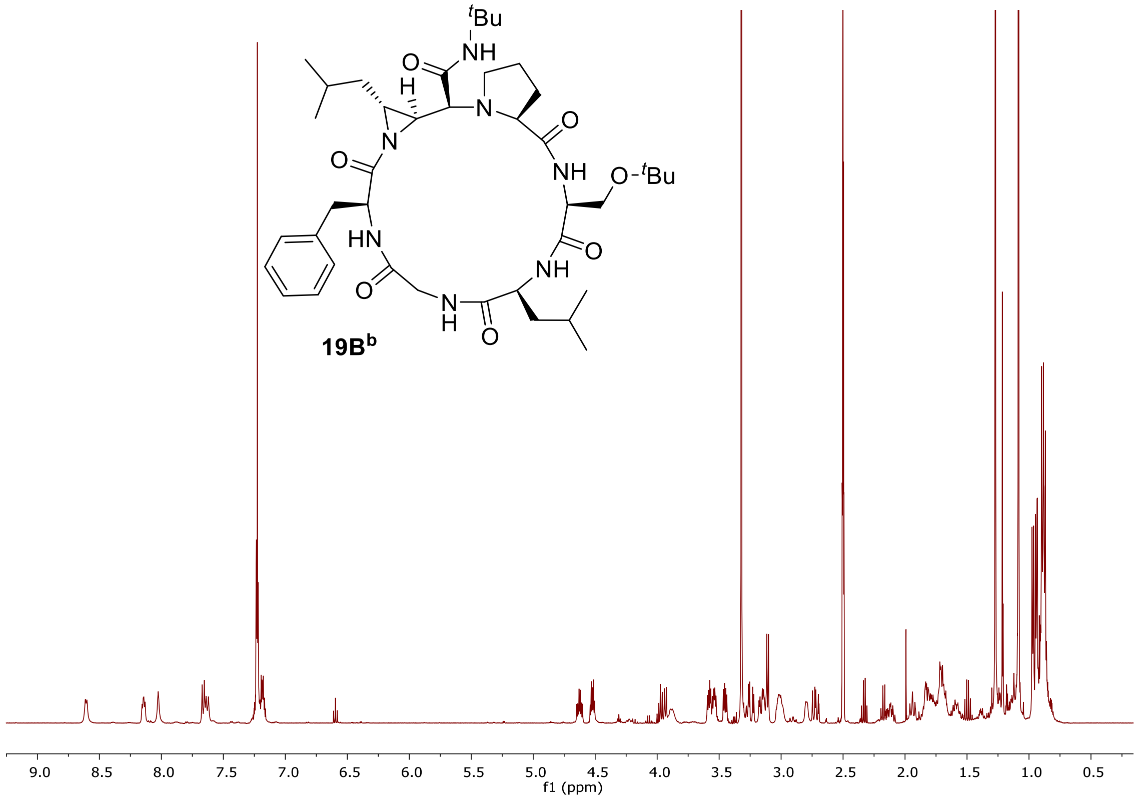

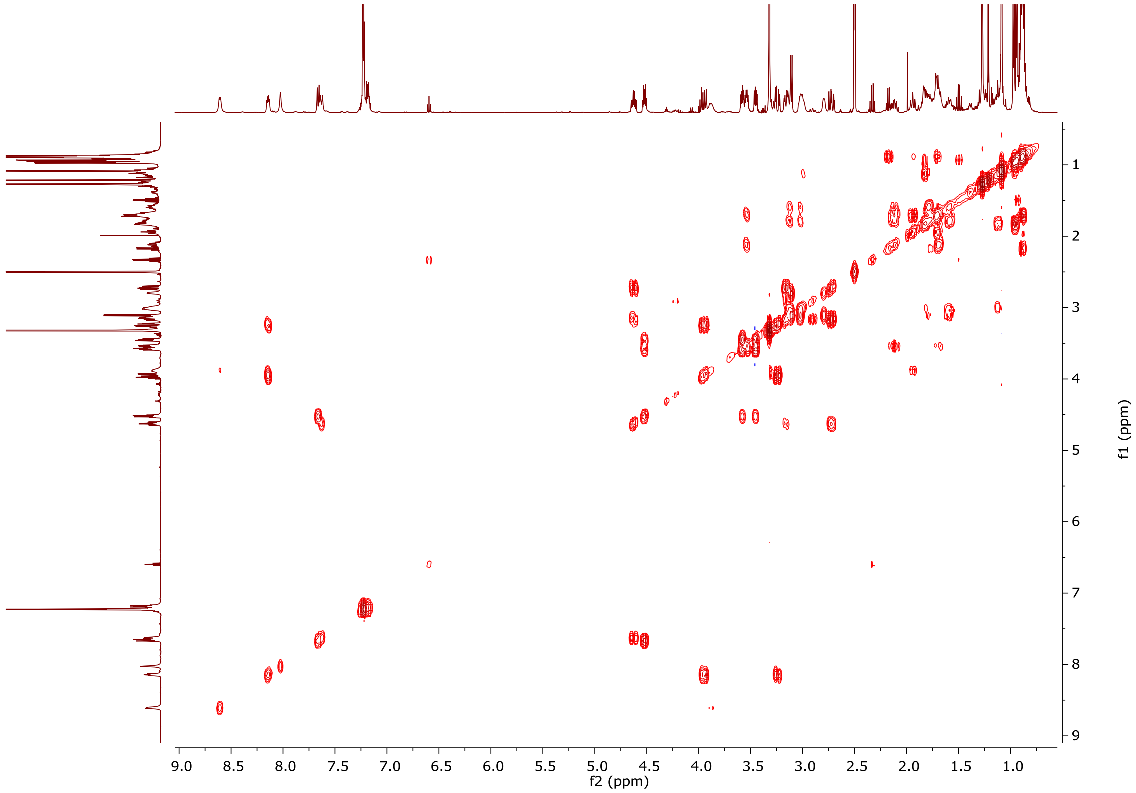

COSY (DMSO-*d6*, 25 °C)

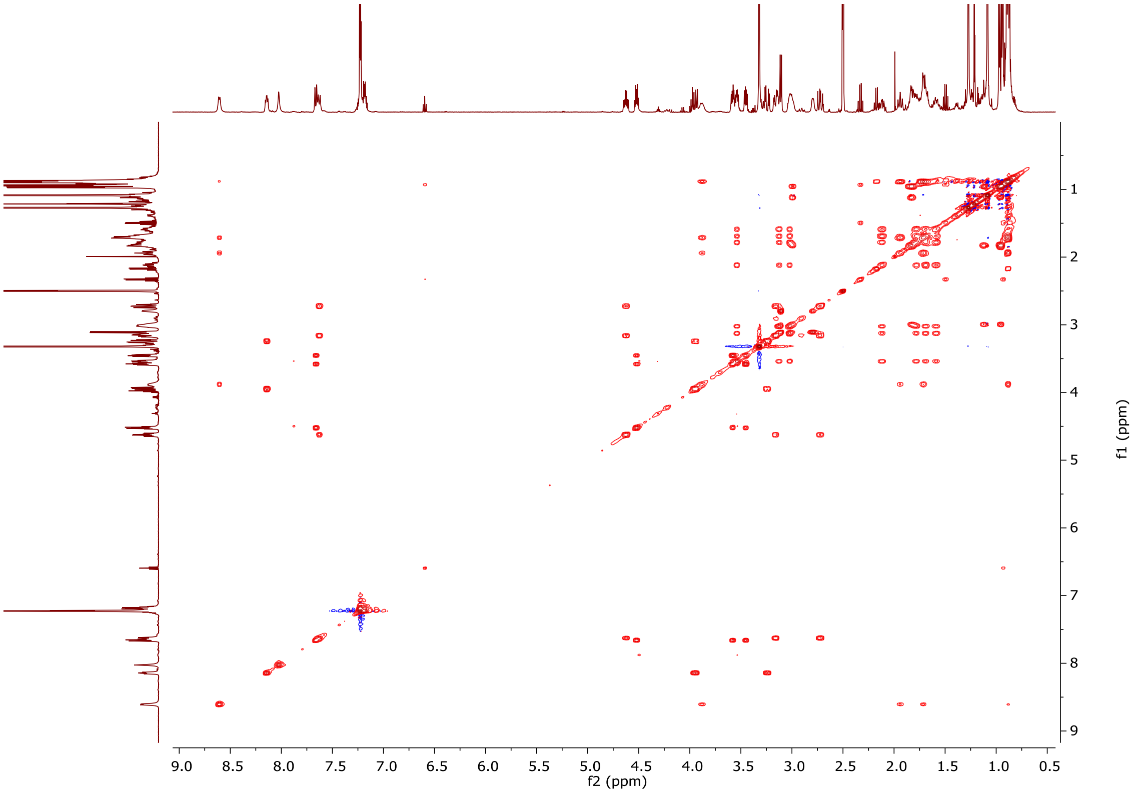

TOCSY (DMSO-*d6*, 25 ^{o}C)

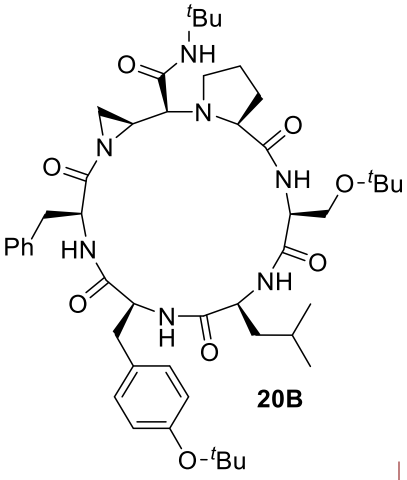

20B

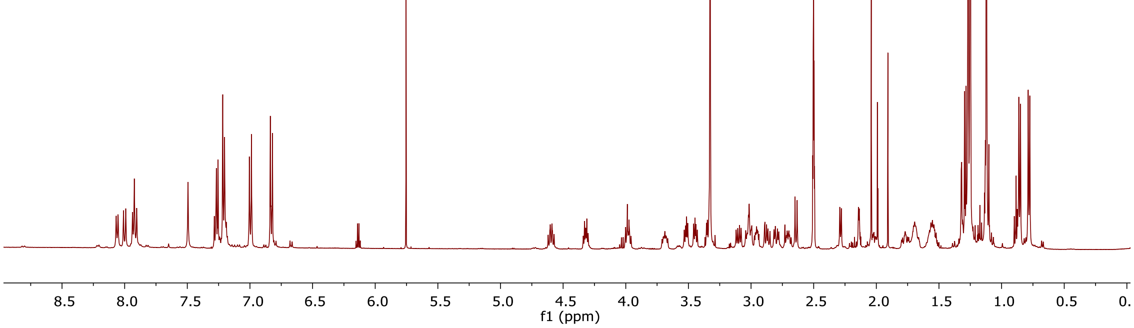

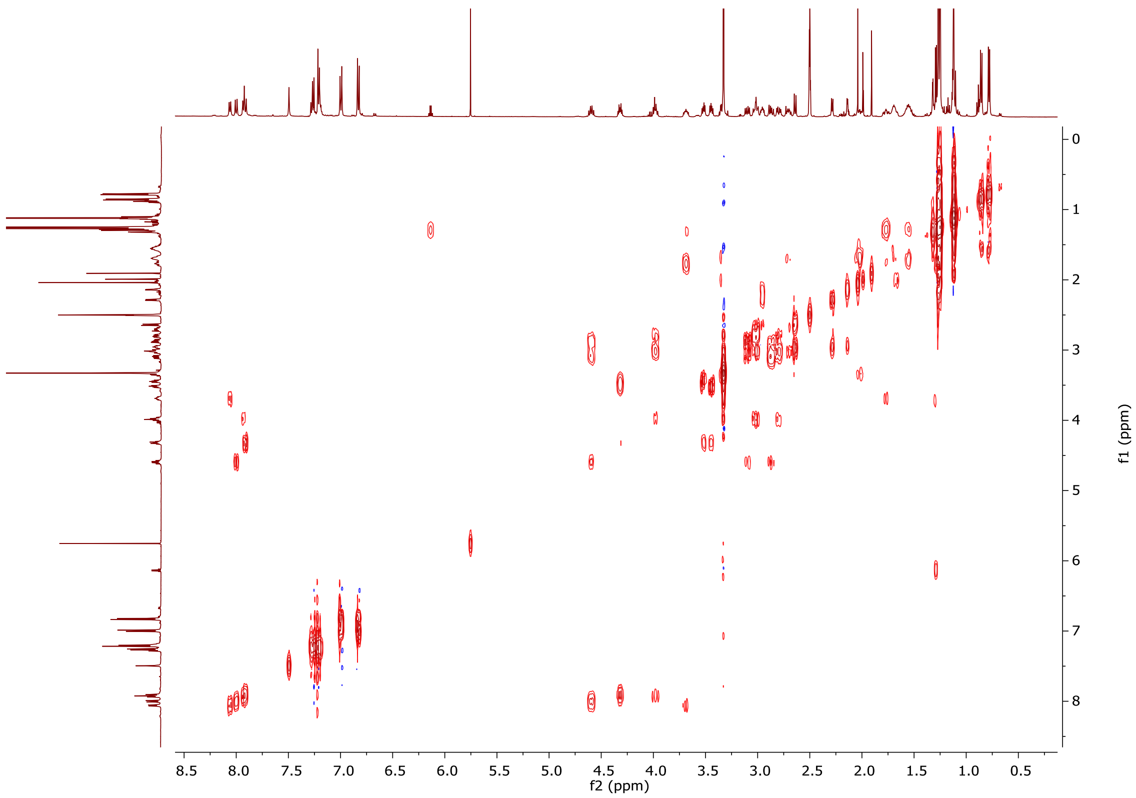

COSY (DMSO-d_6, 25 ^oC)

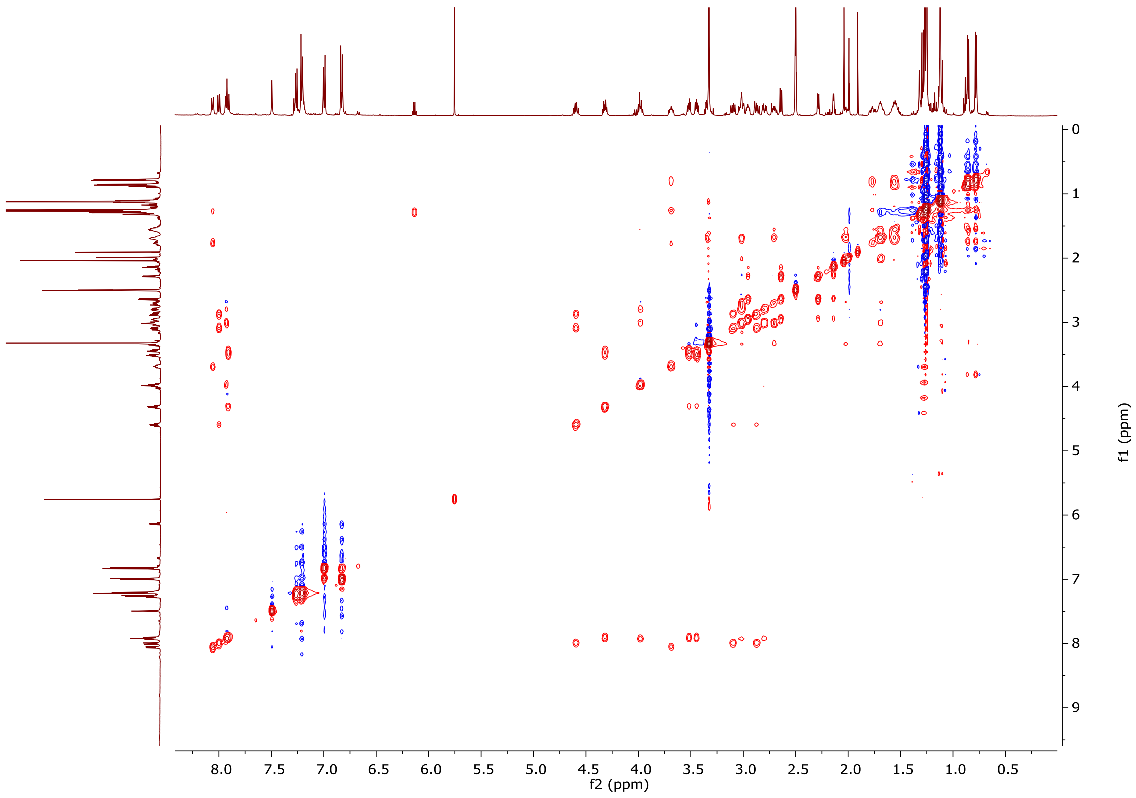

TOCSY (DMSO-d_6, 25 ^oC)

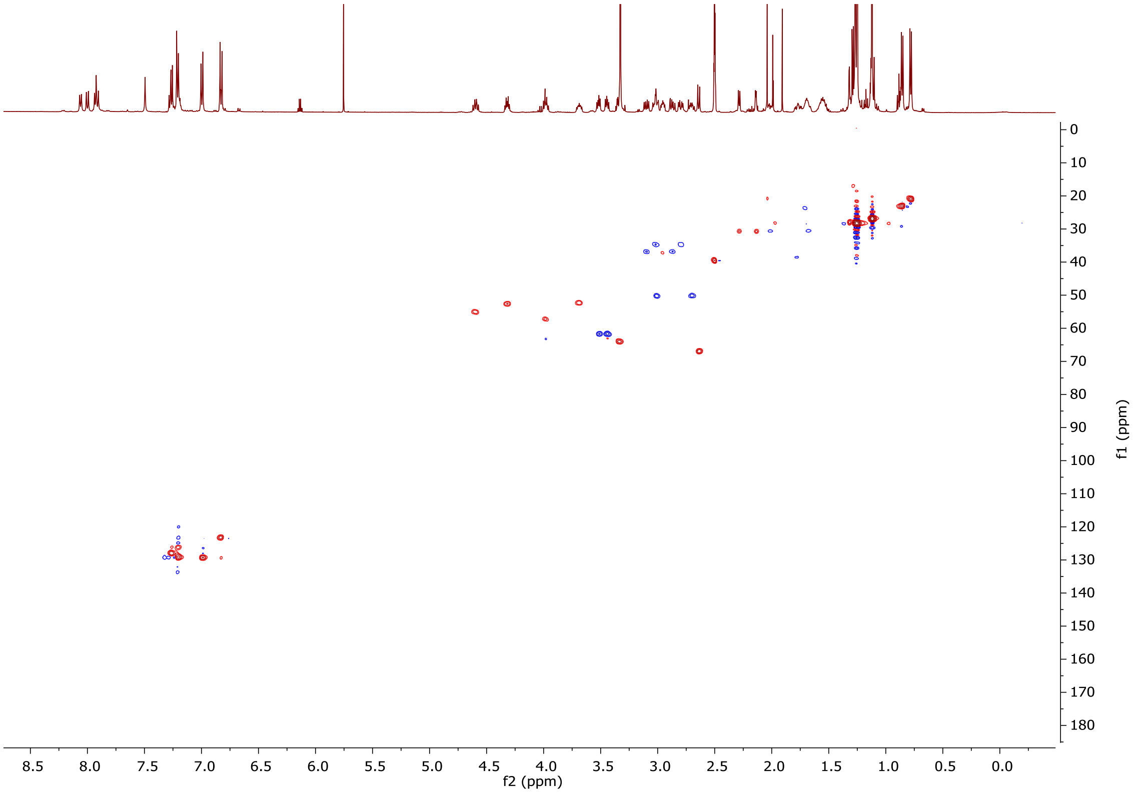

HSQC (DMSO-d_6, 25 ^oC)

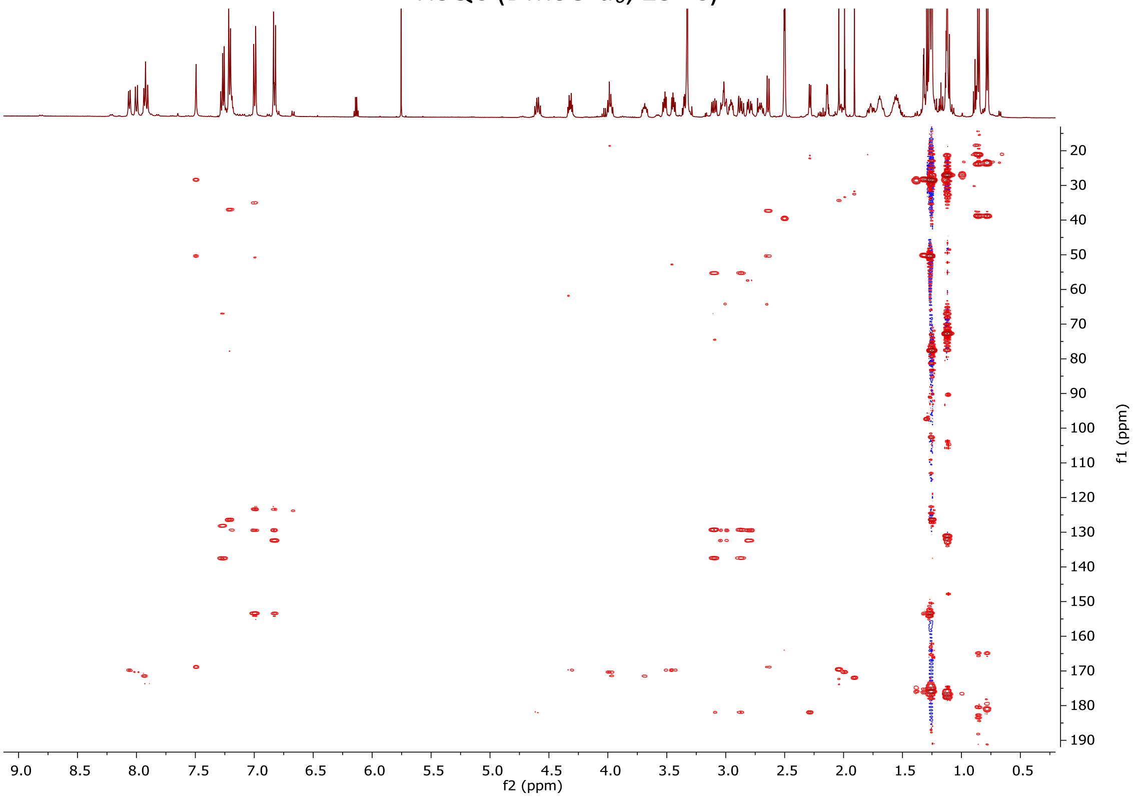

HMBC (DMSO-d_6, 25 ^oC)

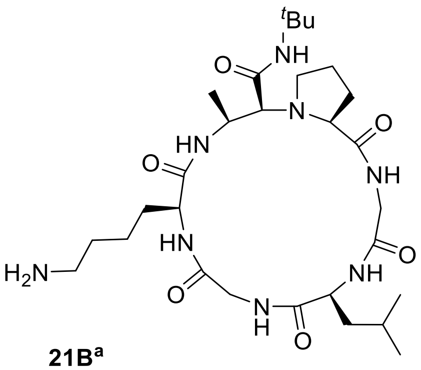

21B[a]

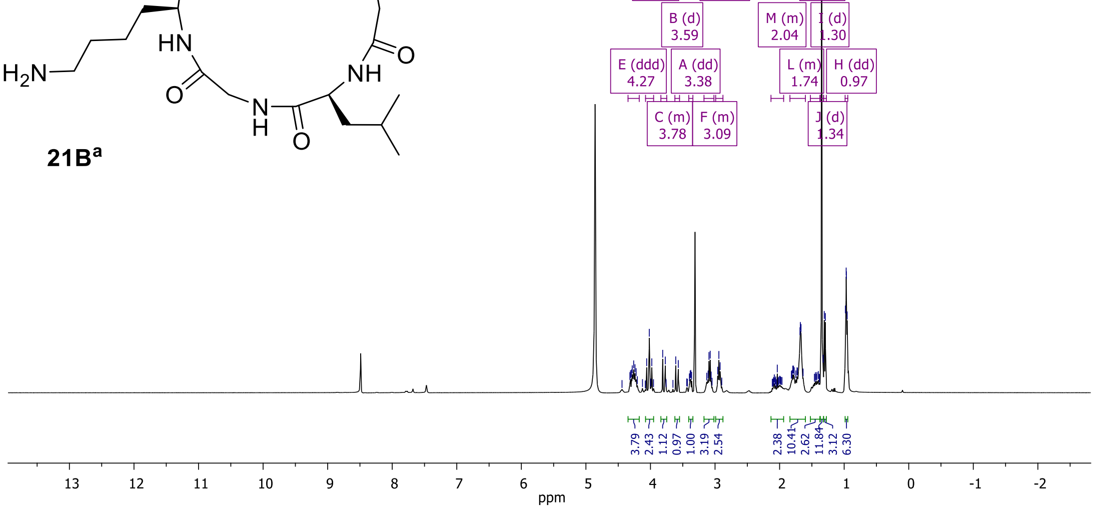

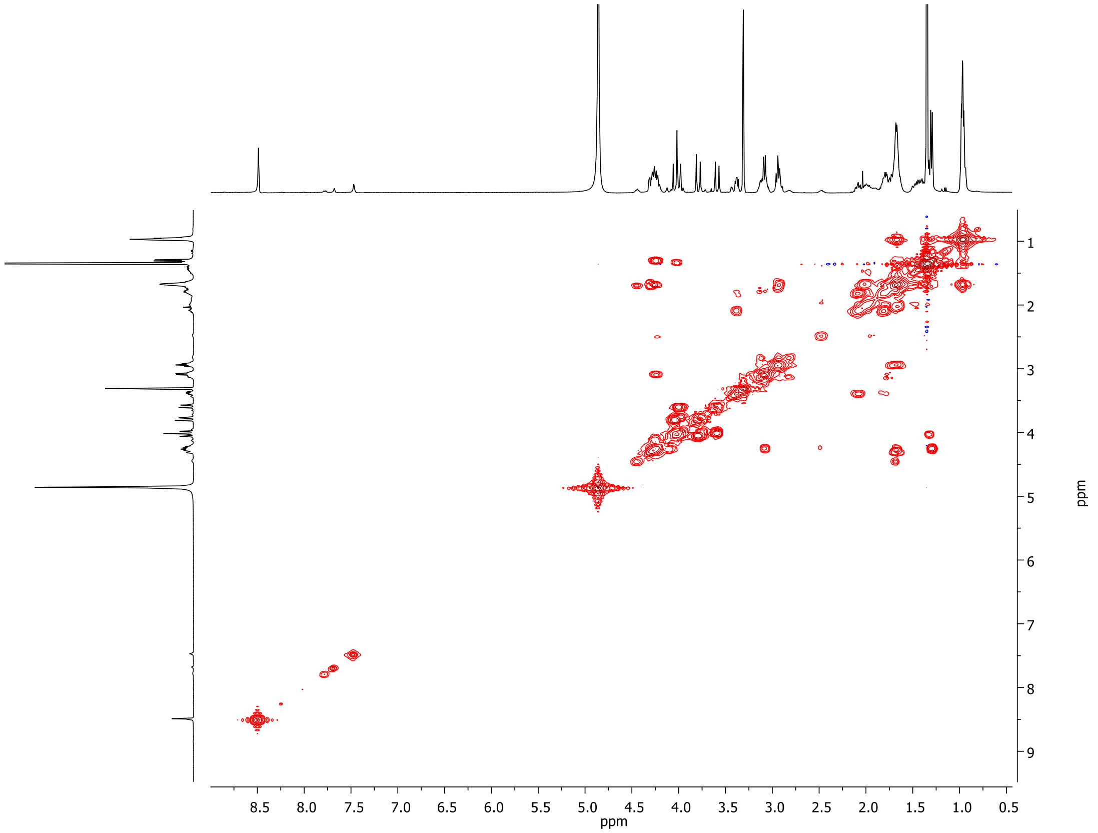

COSY (CD_3OD, 25 °C)

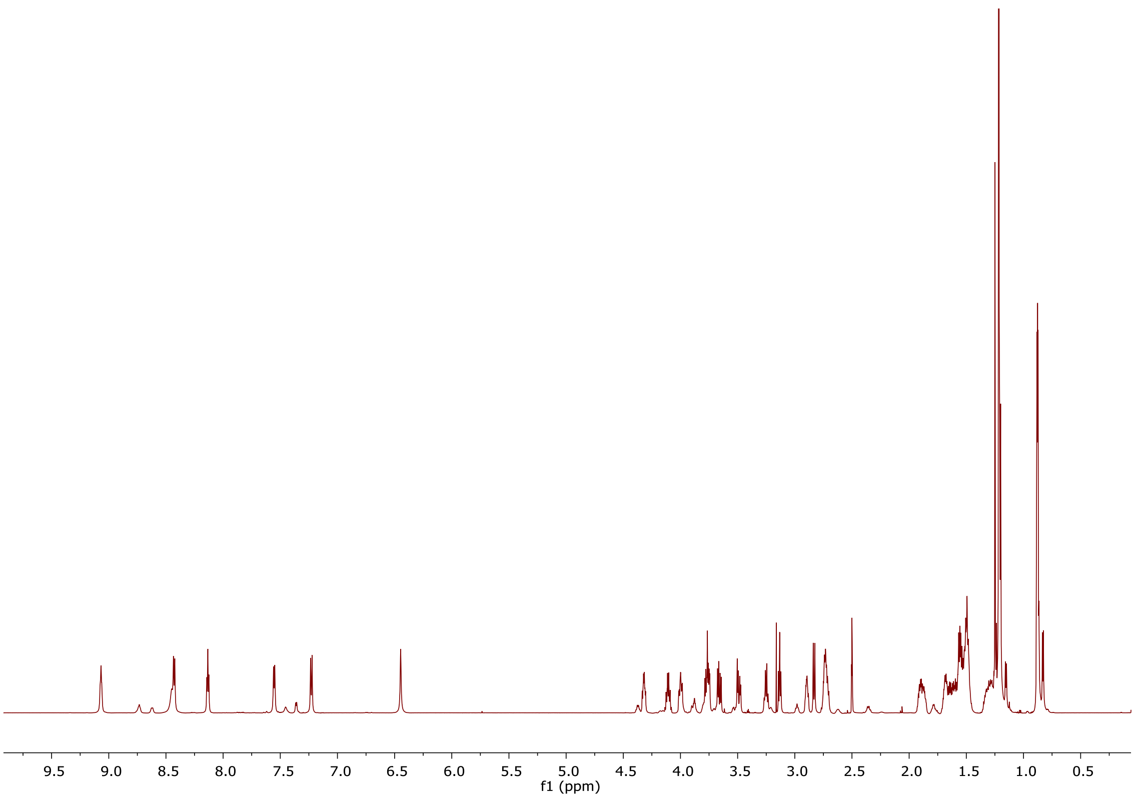
9.5
9.0
8.5
8.0
7.5
7.0
6.5
6.0
5.5
5.0
4.5
4.0
3.5
3.0
2.5
2.0
1.5
1.0
0.5
f1 (ppm)

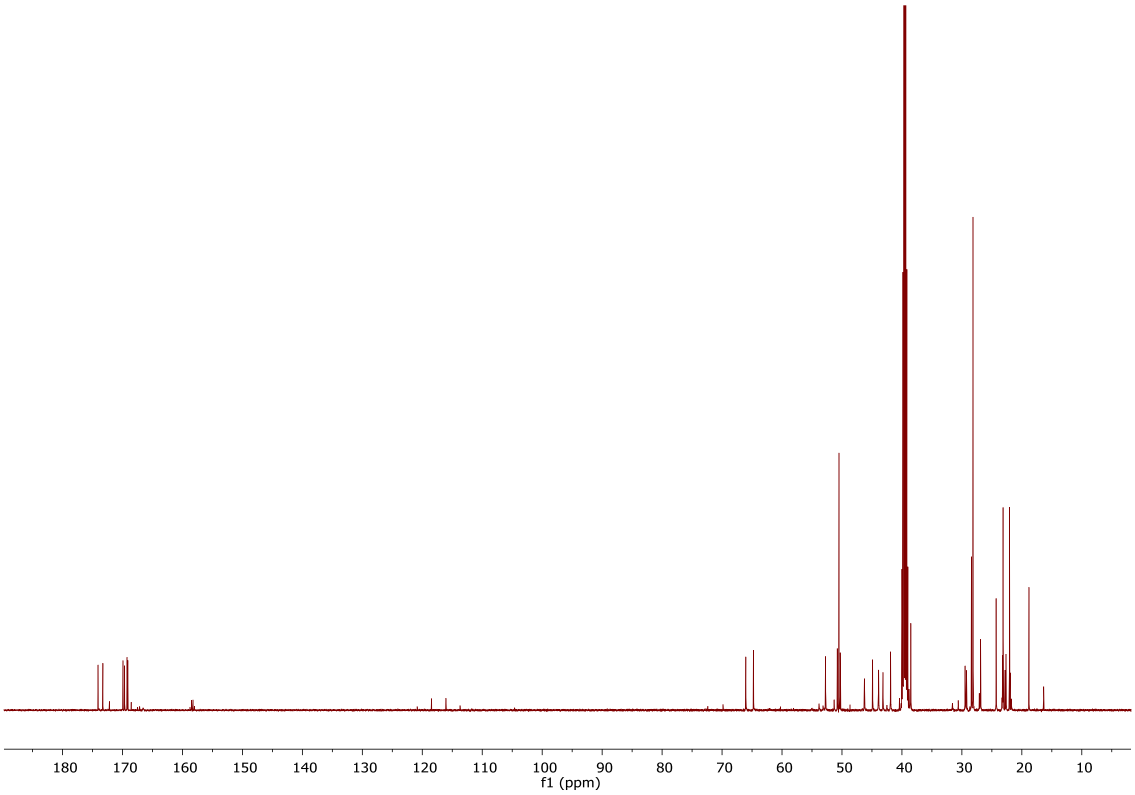
180
170
160
150
140
130
120
110
100
90
80
70
60
50
40
30
20
10
f1 (ppm)

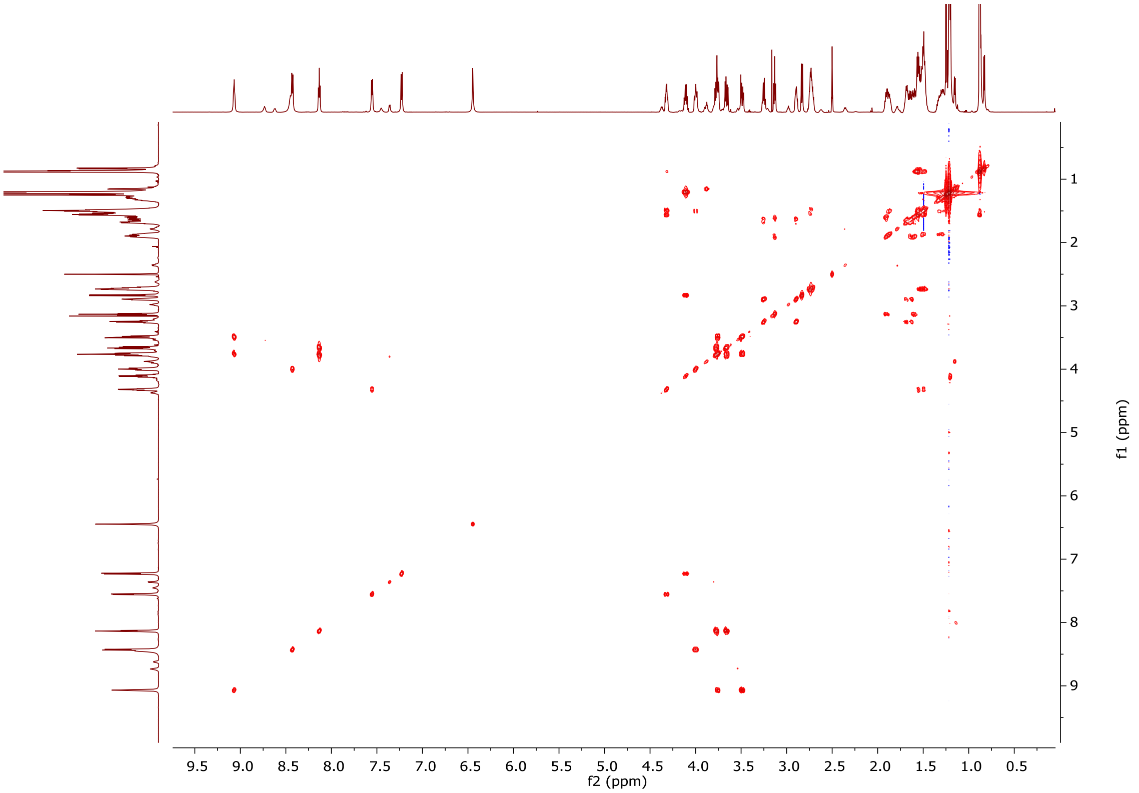

COSY (DMSO-*d6*, 25 °C)

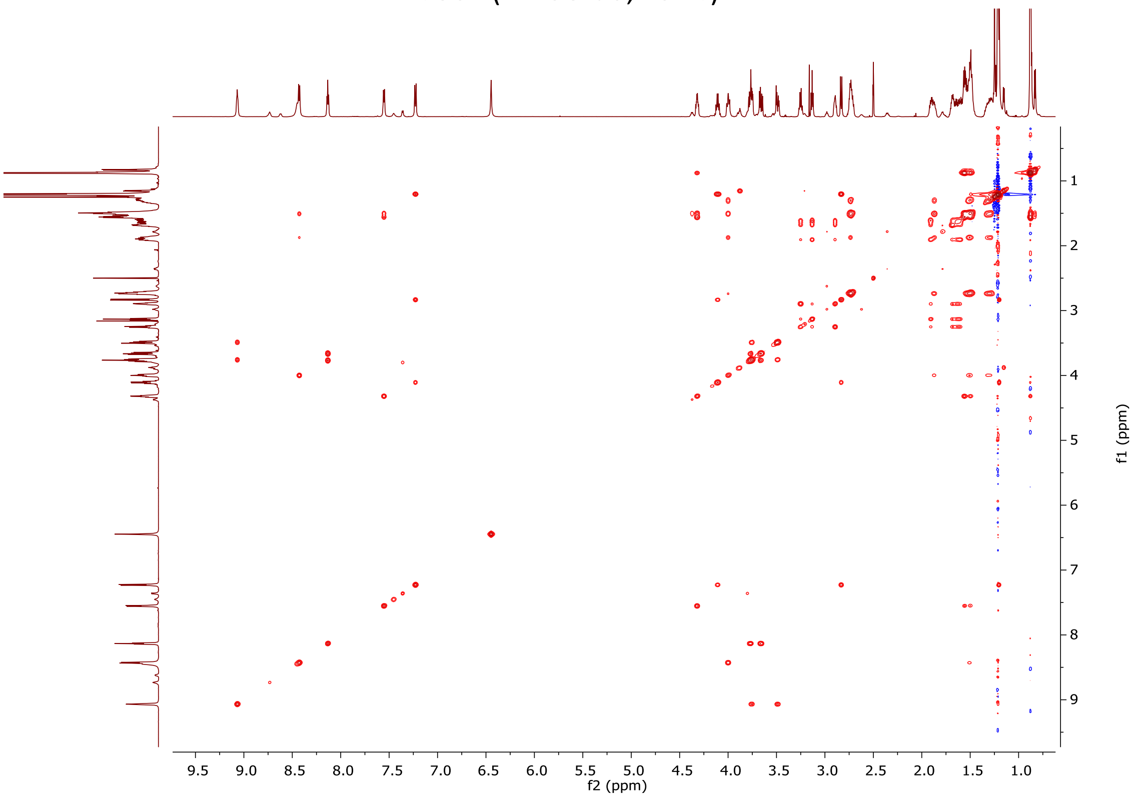

TOCSY (DMSO-*d6*, 25 °C)

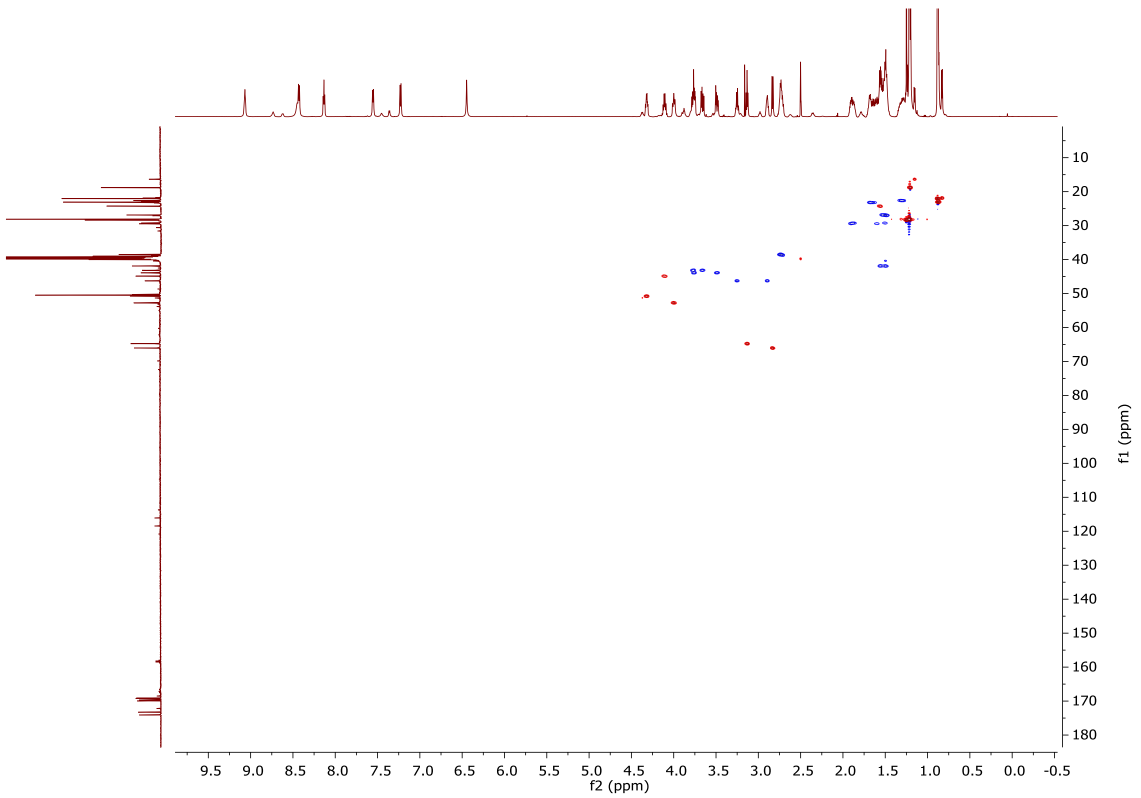

HSQC (DMSO-*d6*, 25 ºC)

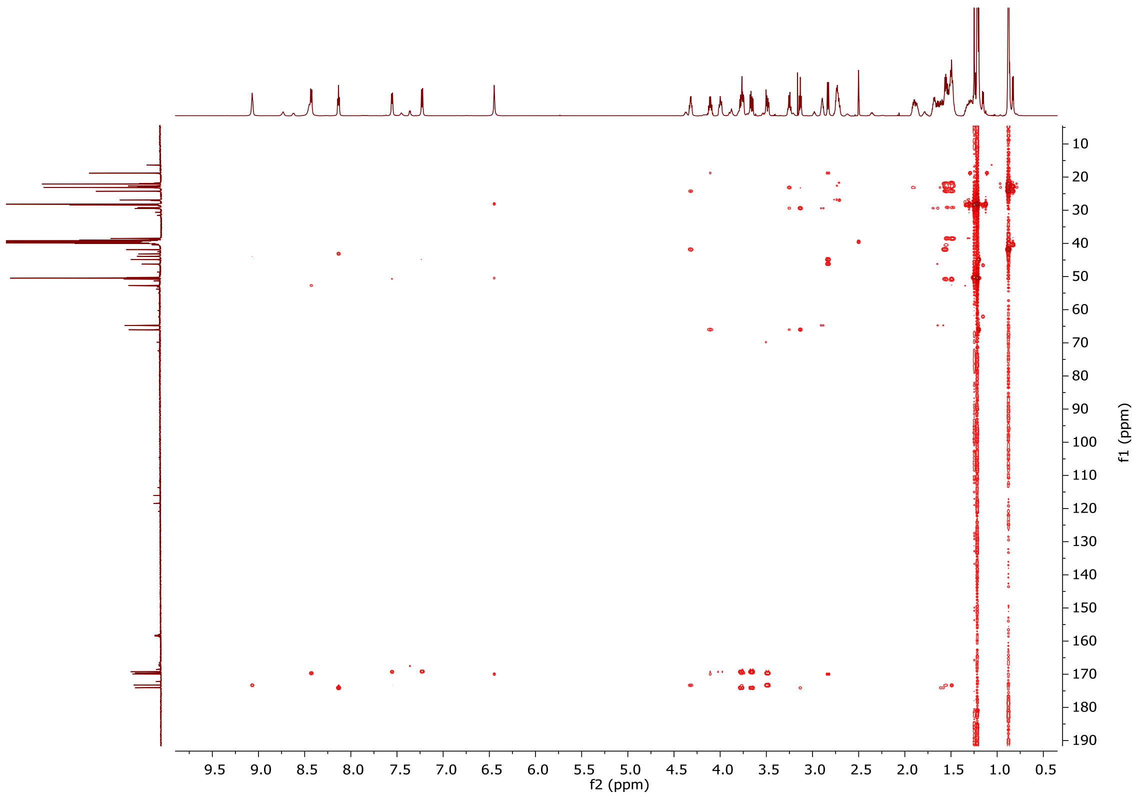

HMBC (DMSO-*d6*, 25 ºC)

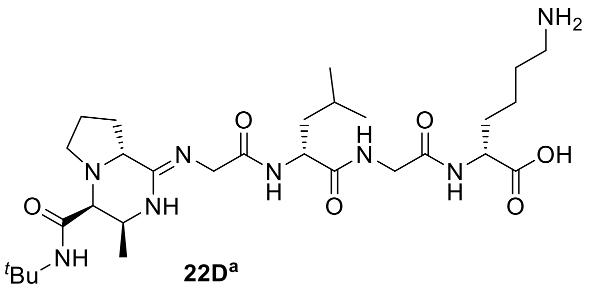

22D[a]

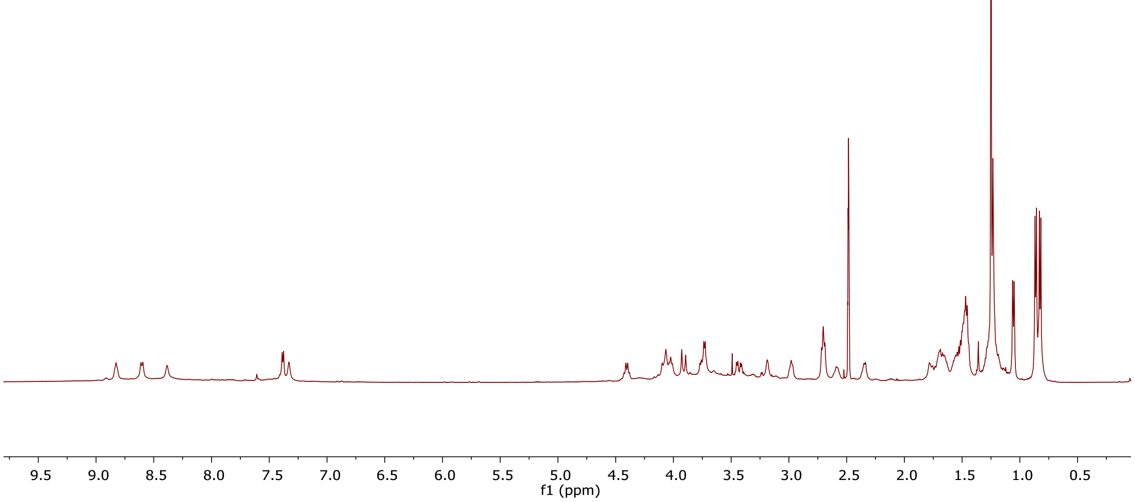

9.5
9.0
8.5
8.0
7.5
7.0
6.5
6.0
5.5
5.0
4.5
4.0
3.5
3.0
2.5
2.0
1.5
1.0
0.5
f1 (ppm)

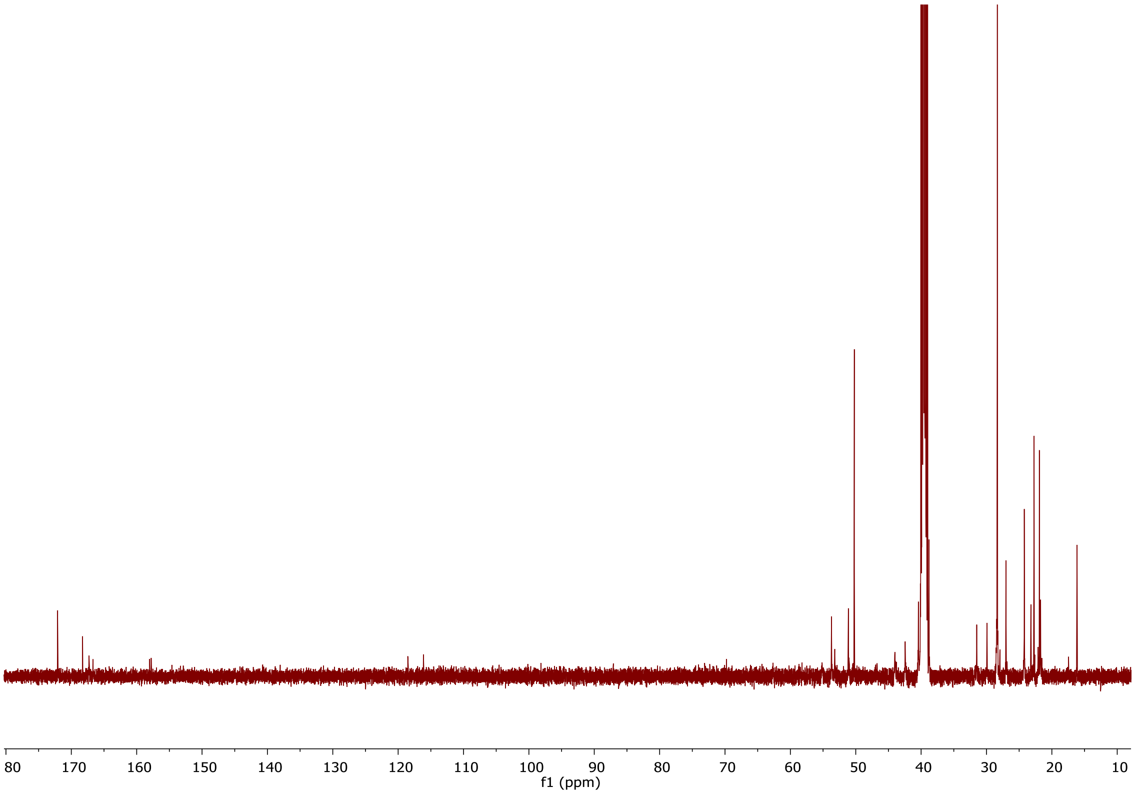

80
170
160
150
140
130
120
110
100
90
80
70
60
50
40
30
20
10
f1 (ppm)

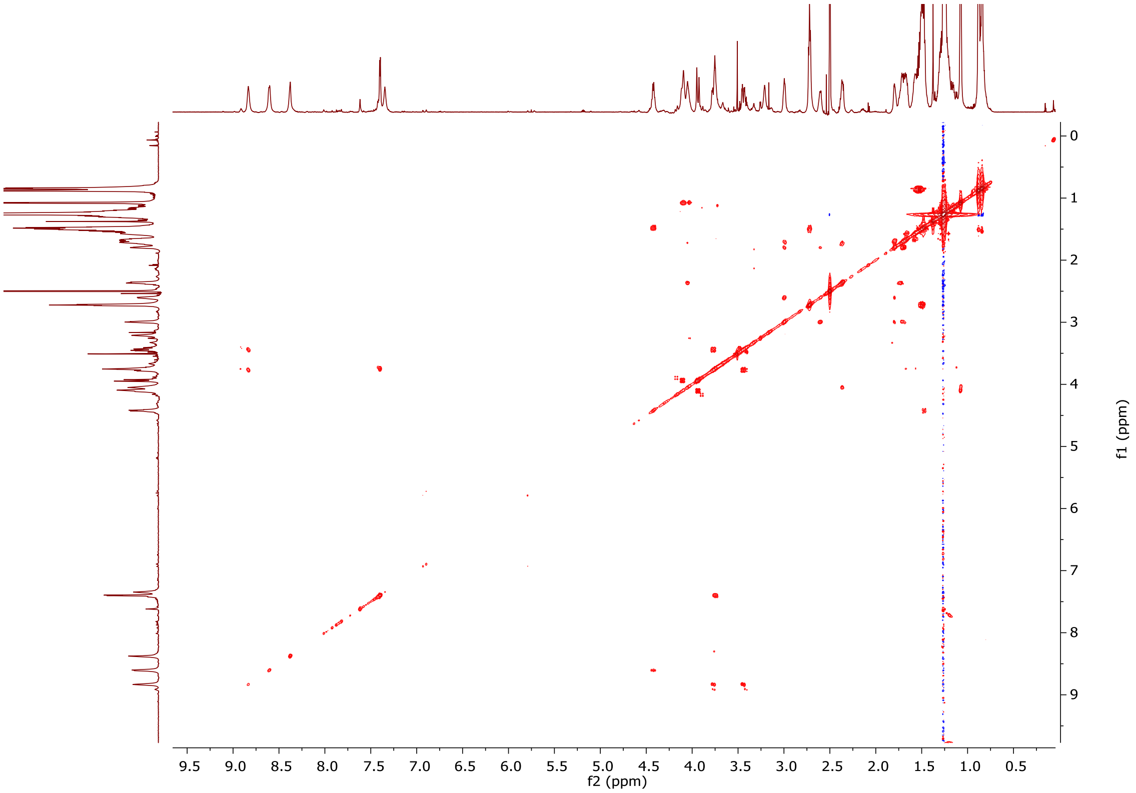

COSY (DMSO-*d6*, 25 ^{o}C)

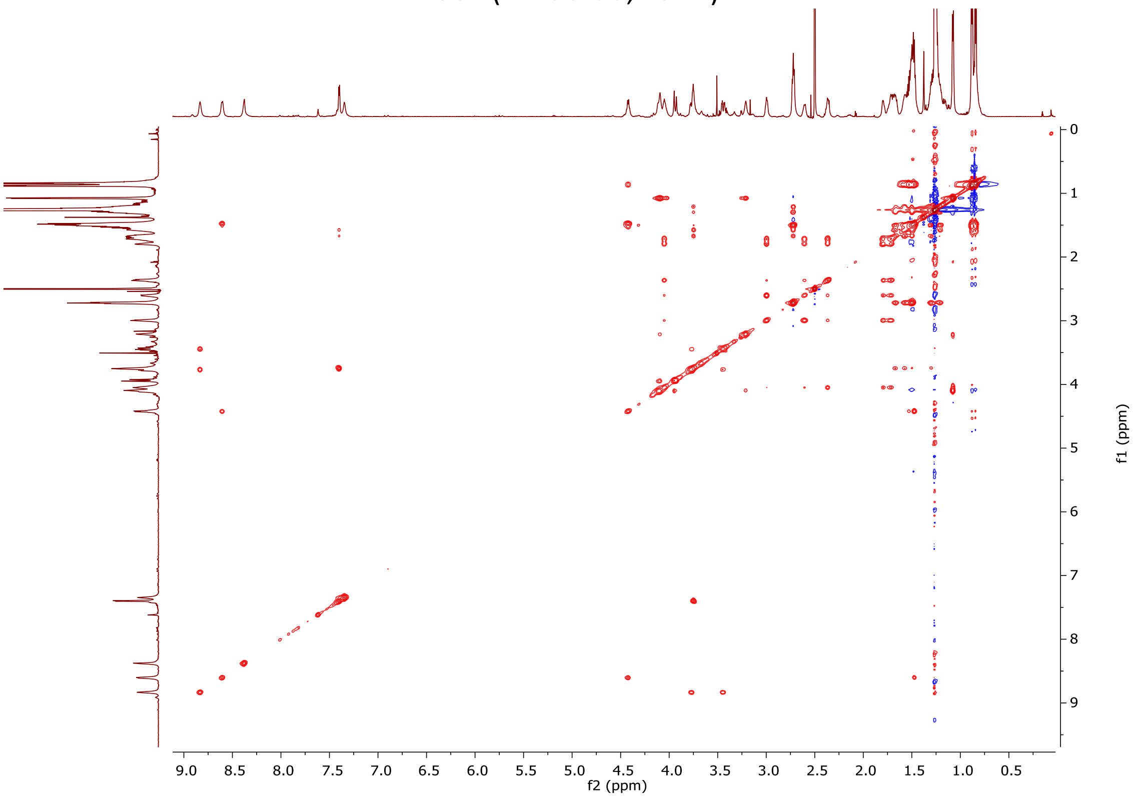

TOCSY (DMSO-*d6*, 25 ^{o}C)

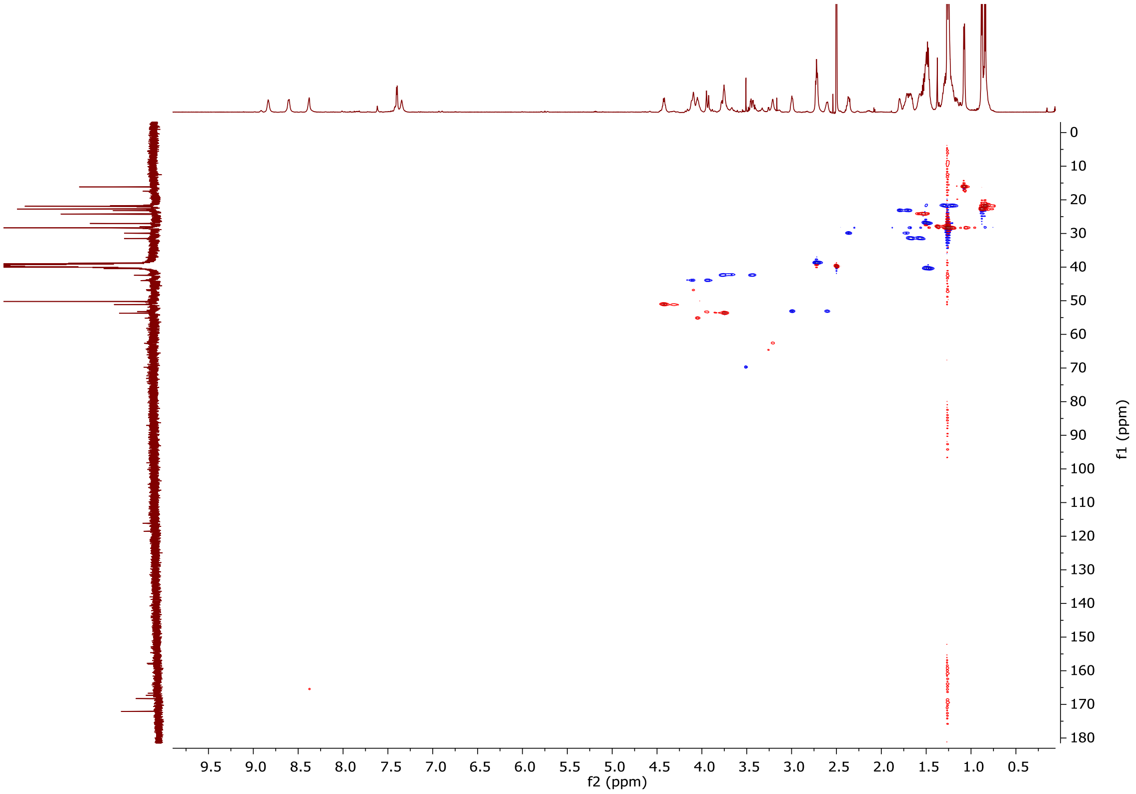

HSQC (DMSO-*d6*, 25 °C)

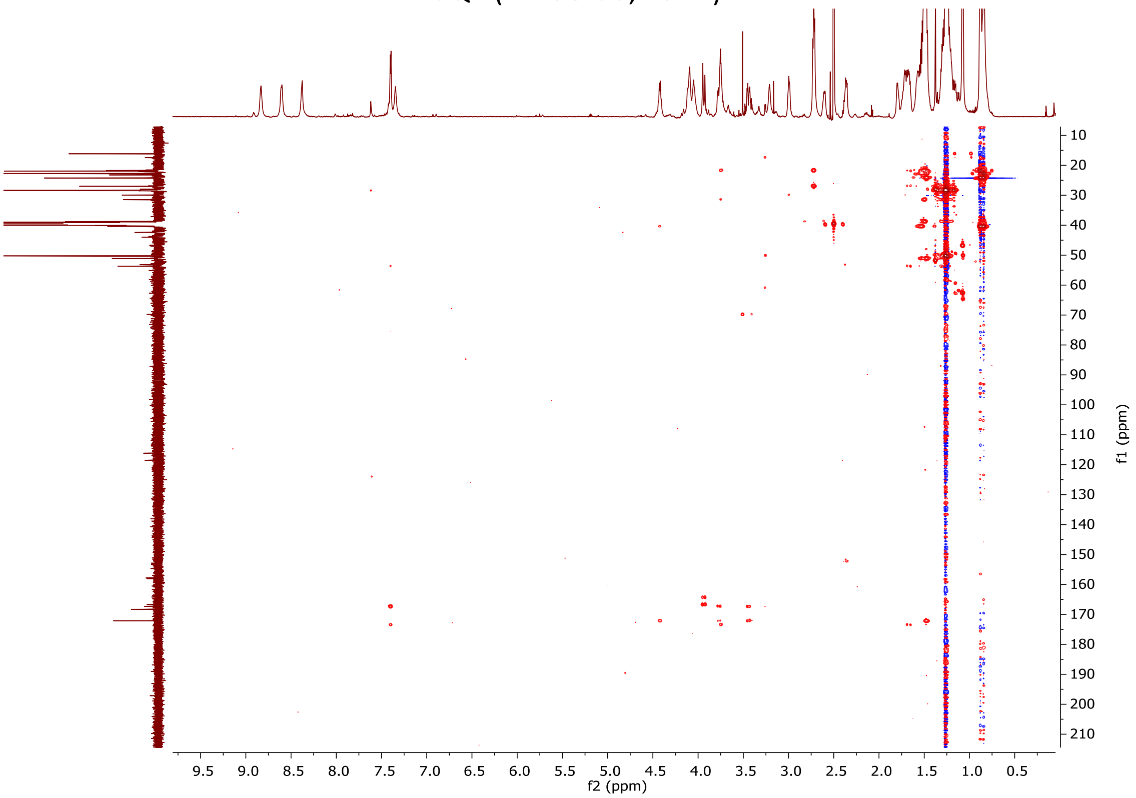

HMBC (DMSO-*d6*, 25 °C)

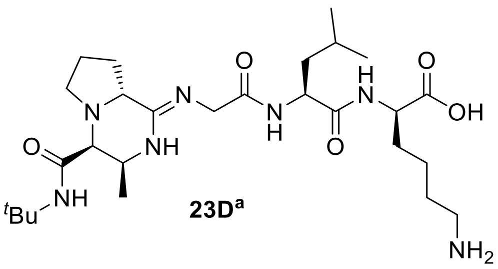
23D^a

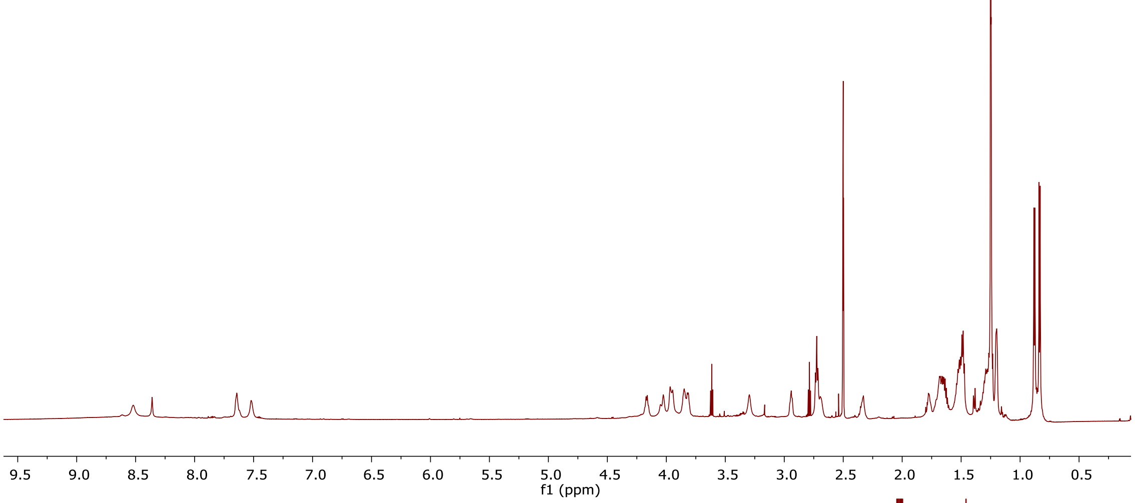
f1 (ppm)

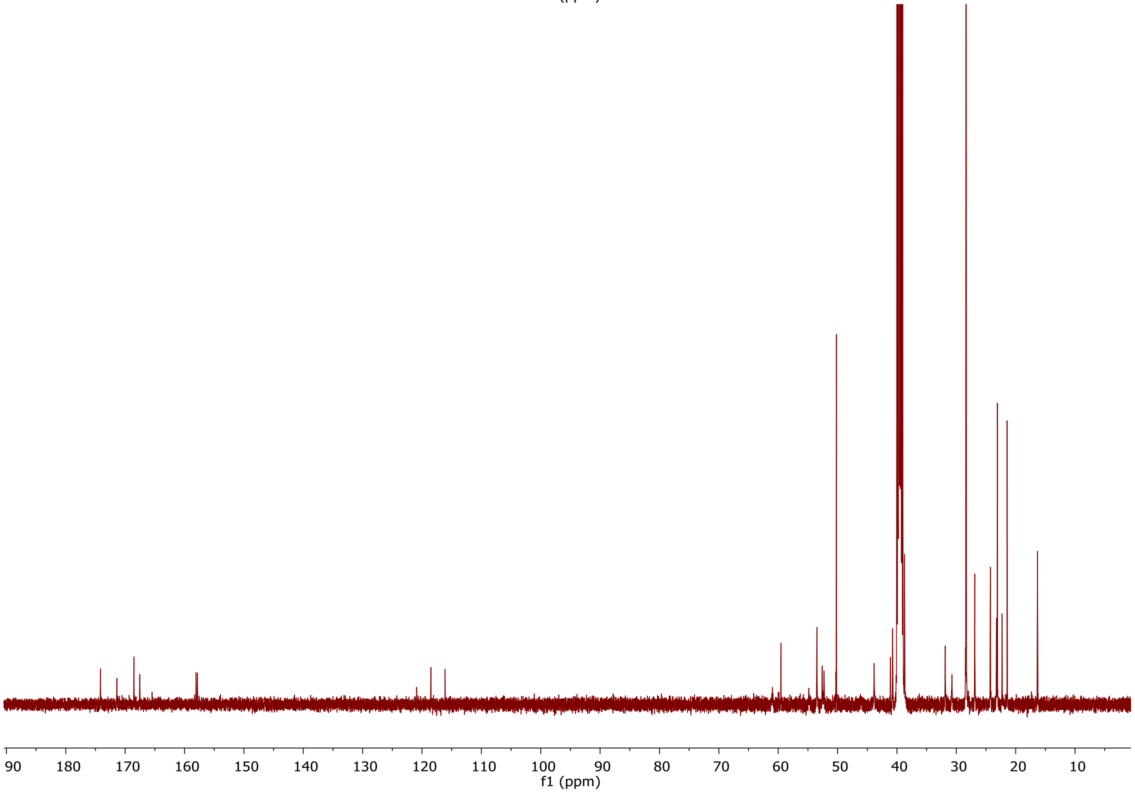
f1 (ppm)

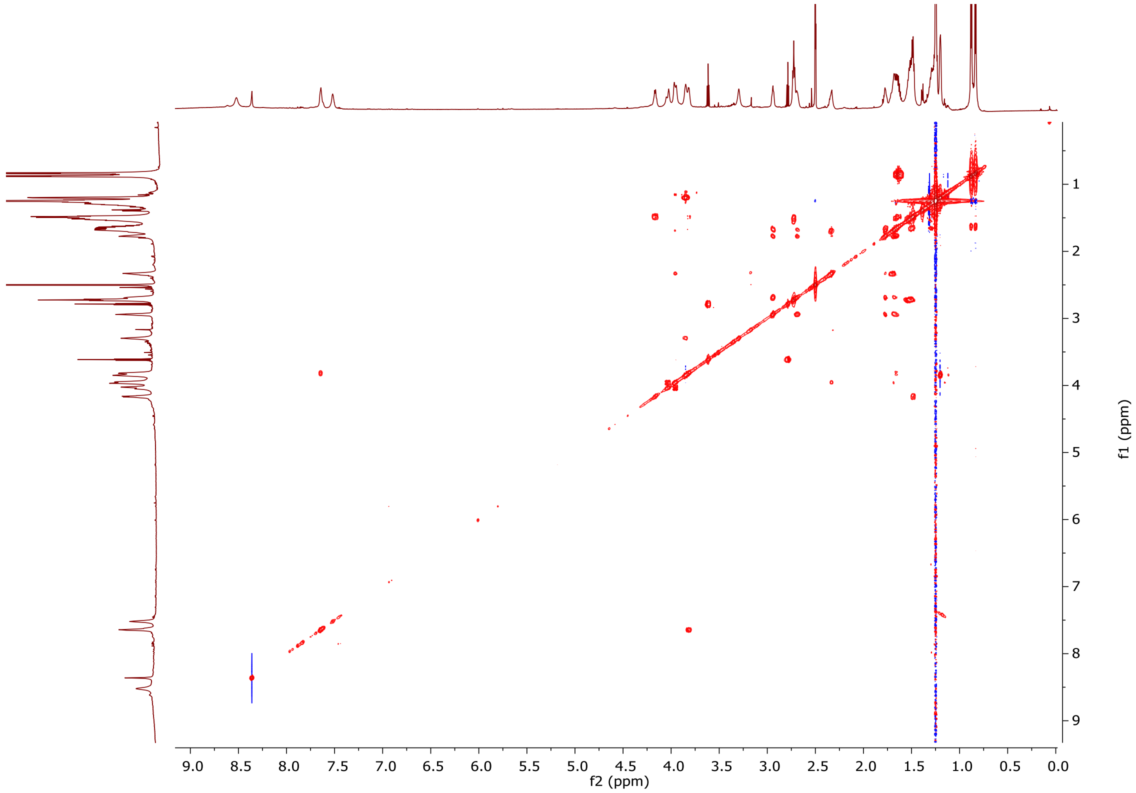

COSY (DMSO-*d6*, 25 °C)

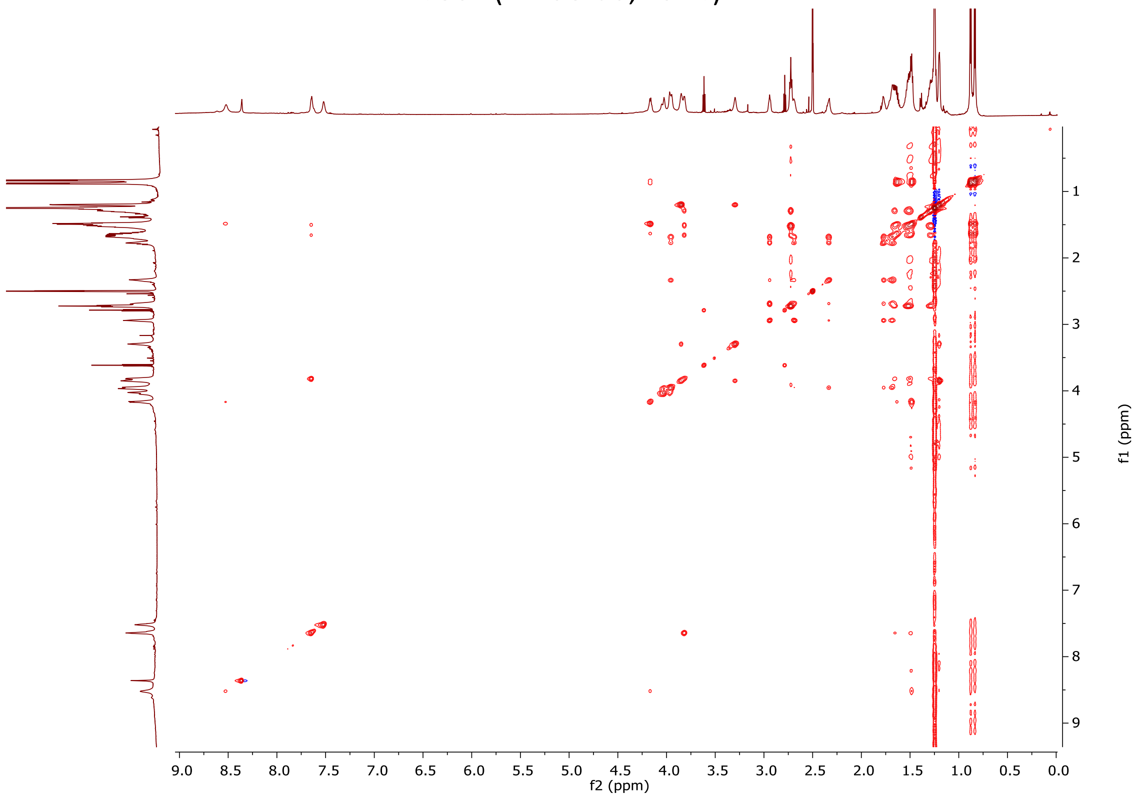

TOCSY (DMSO-*d6*, 25 °C)

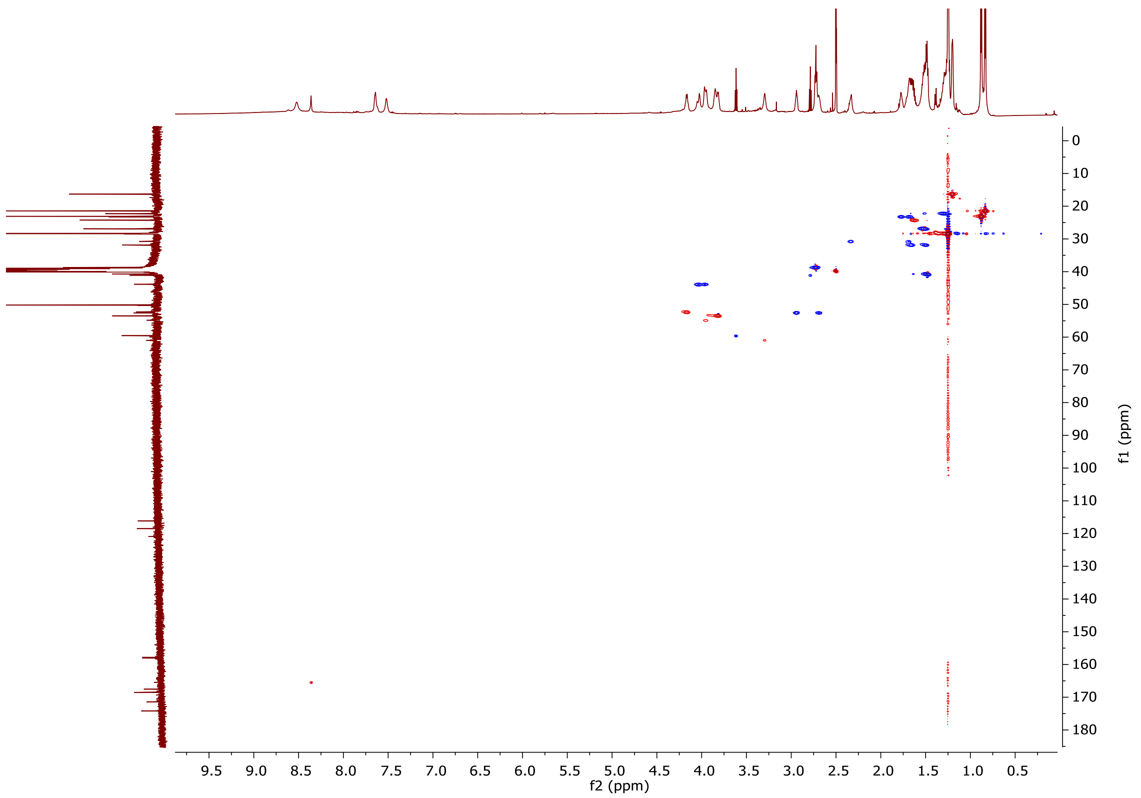

HSQC (DMSO-*d6*, 25 ^{o}C)

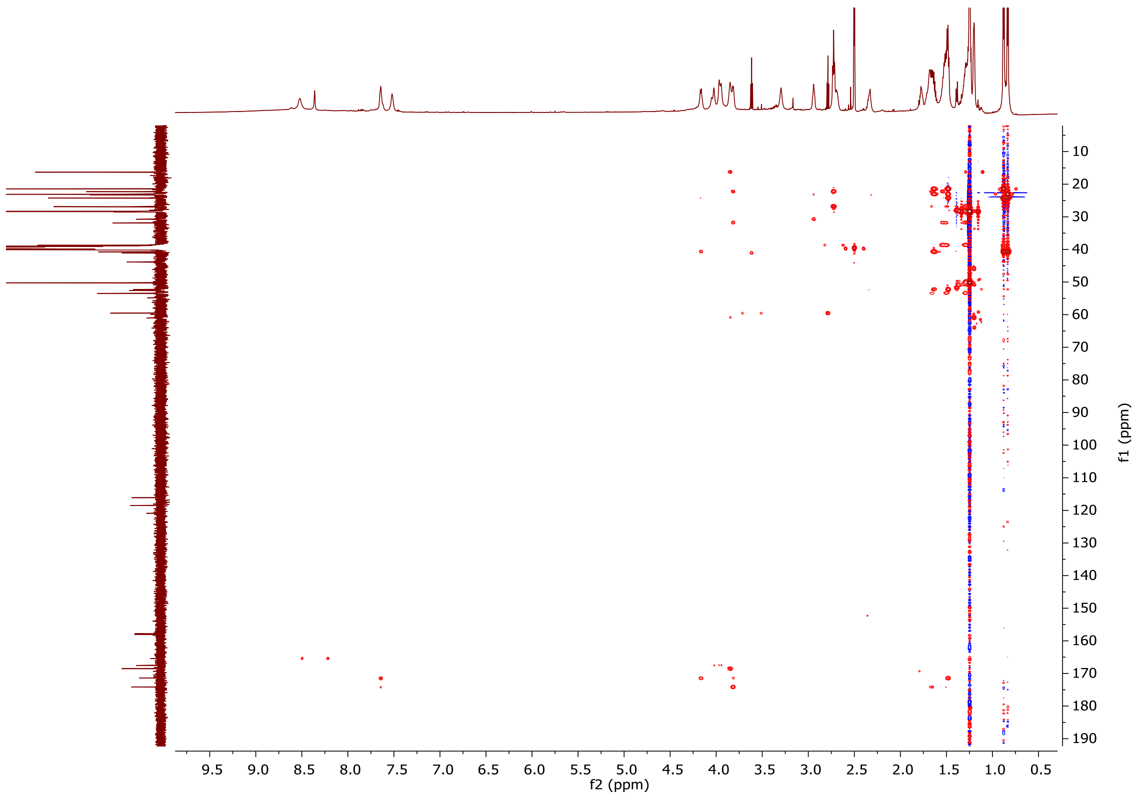

HMBC (DMSO-*d6*, 25 ^{o}C)

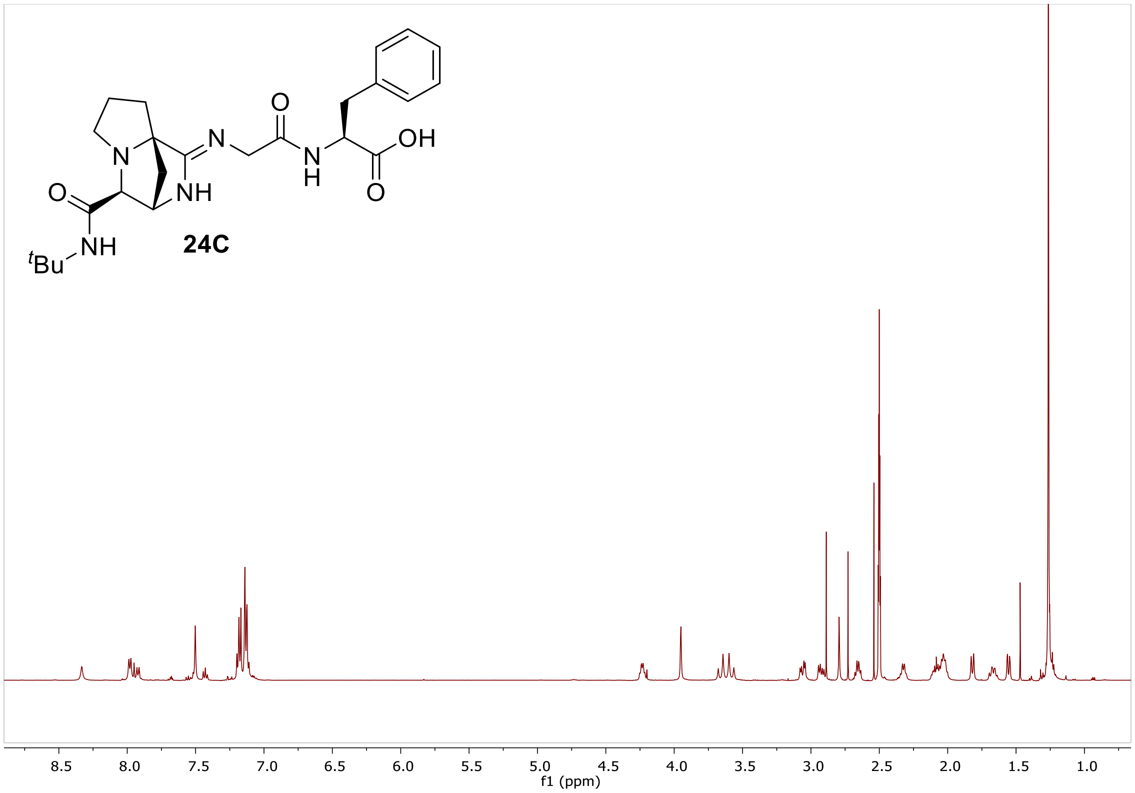

24C
8.5
8.0
7.5
7.0
6.5
6.0
5.5
5.0
4.5
4.0
3.5
3.0
2.5
2.0
1.5
1.0
f1 (ppm)

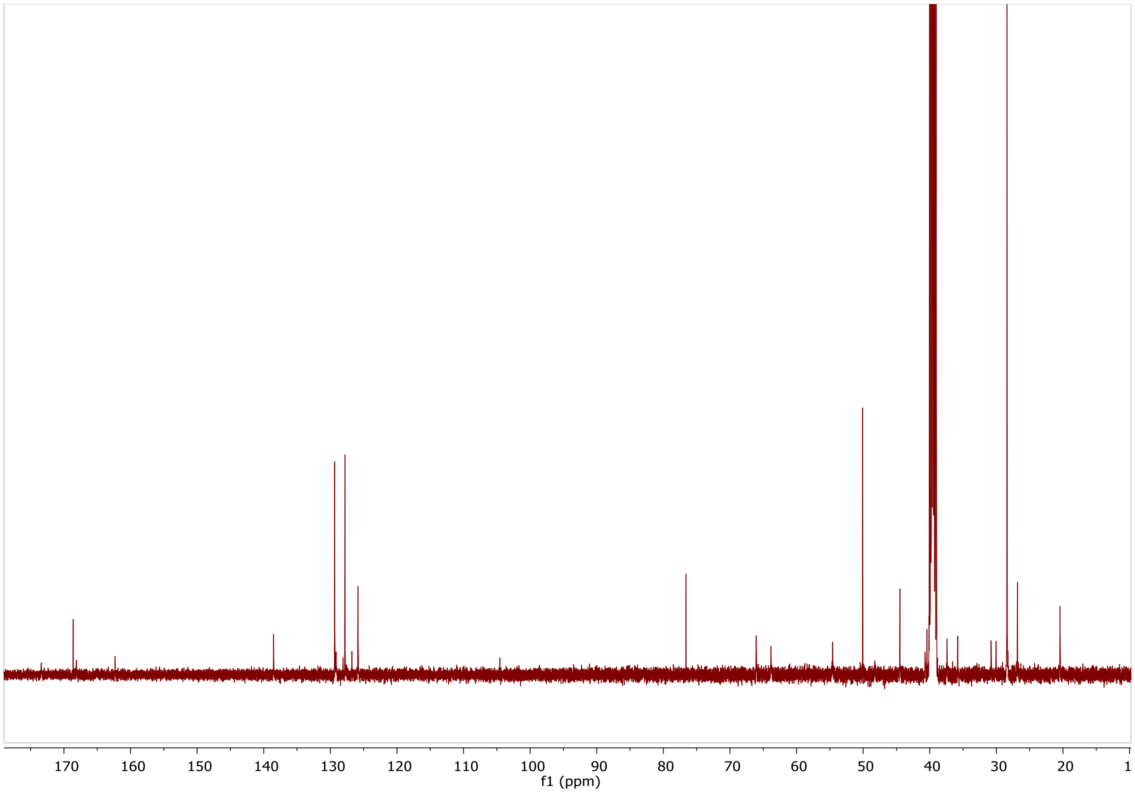

170
160
150
140
130
120
110
100
90
80
70
60
50
40
30
20
1
f1 (ppm)

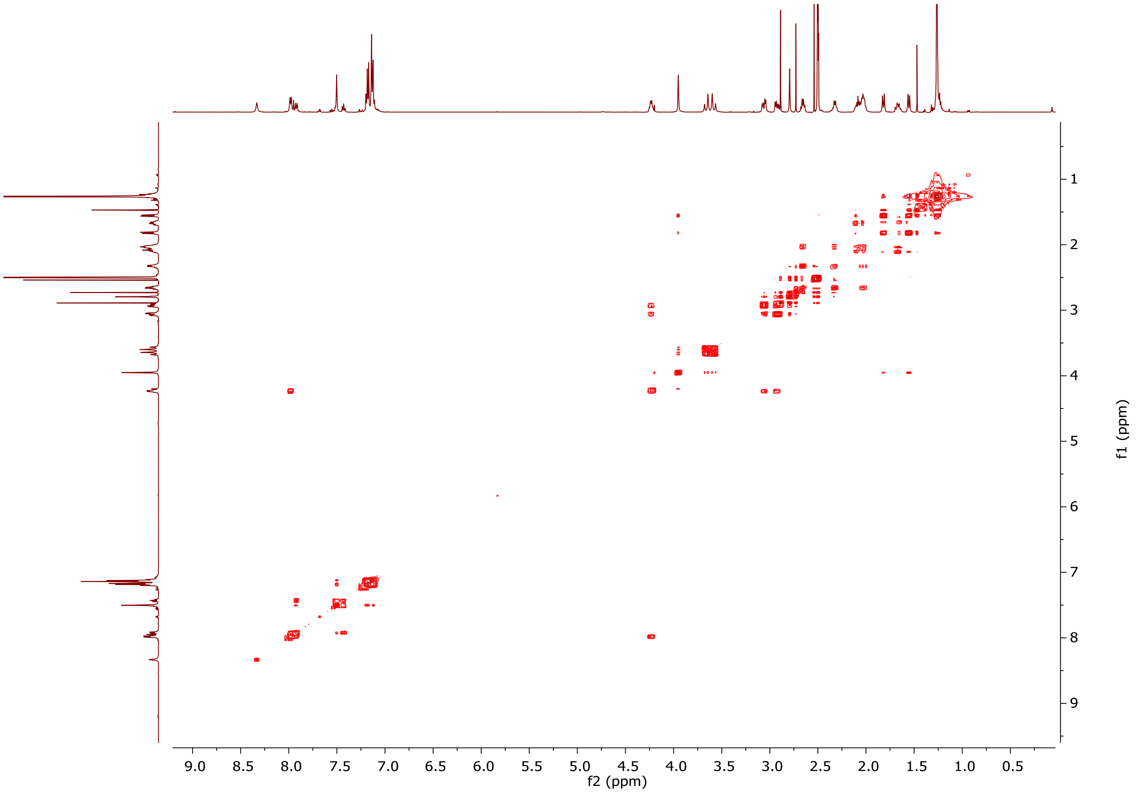

COSY (DMSO-*d6*, 25 °C)

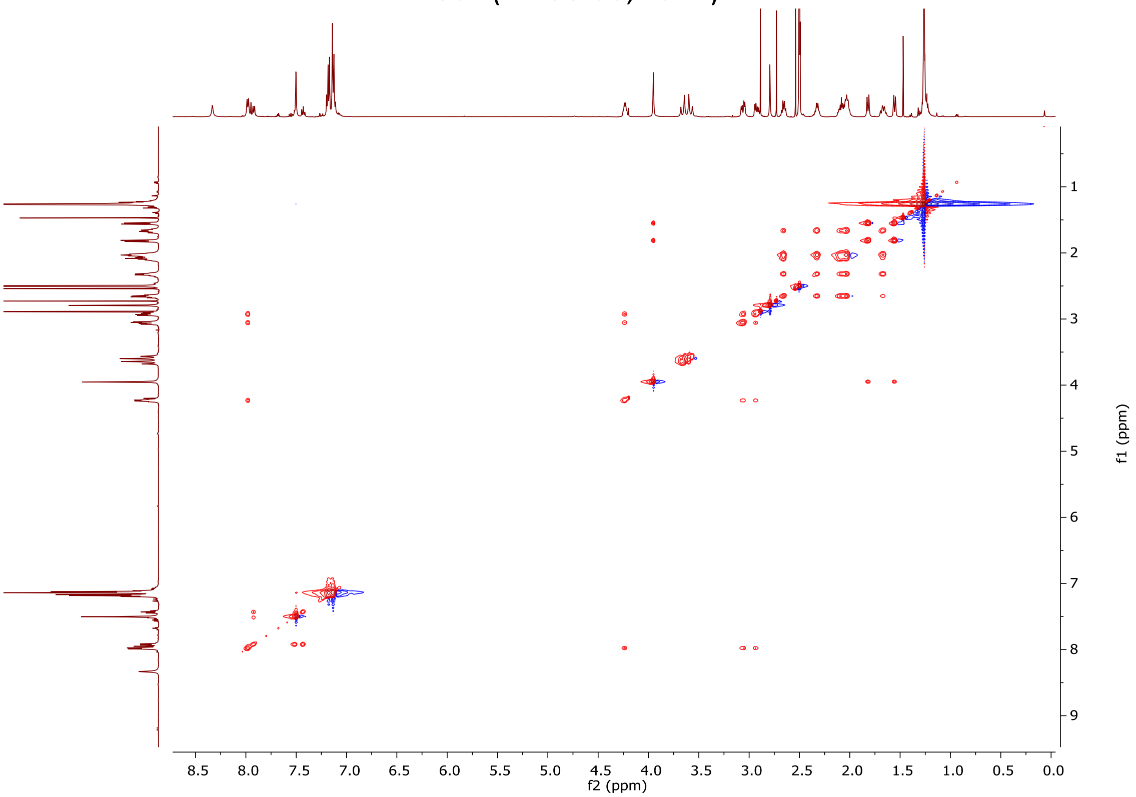

TOCSY (DMSO-*d6*, 25 °C)

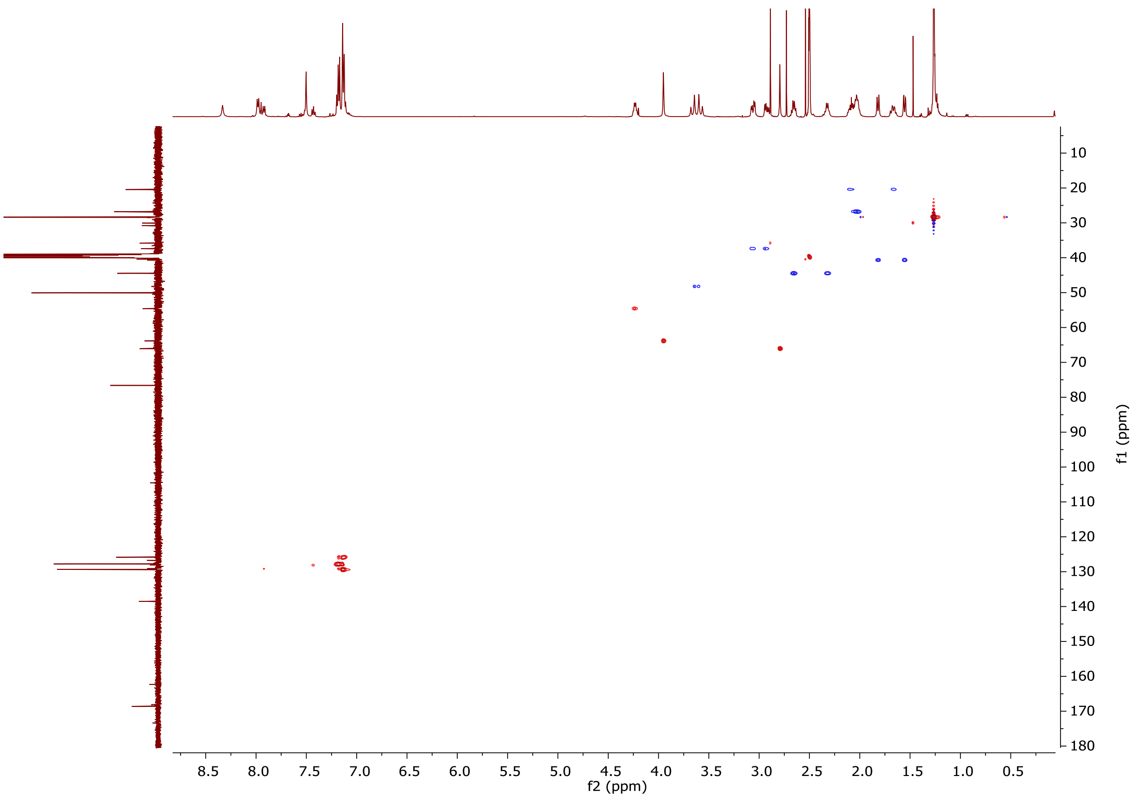

HSQC (DMSO-*d6*, 25 °C)

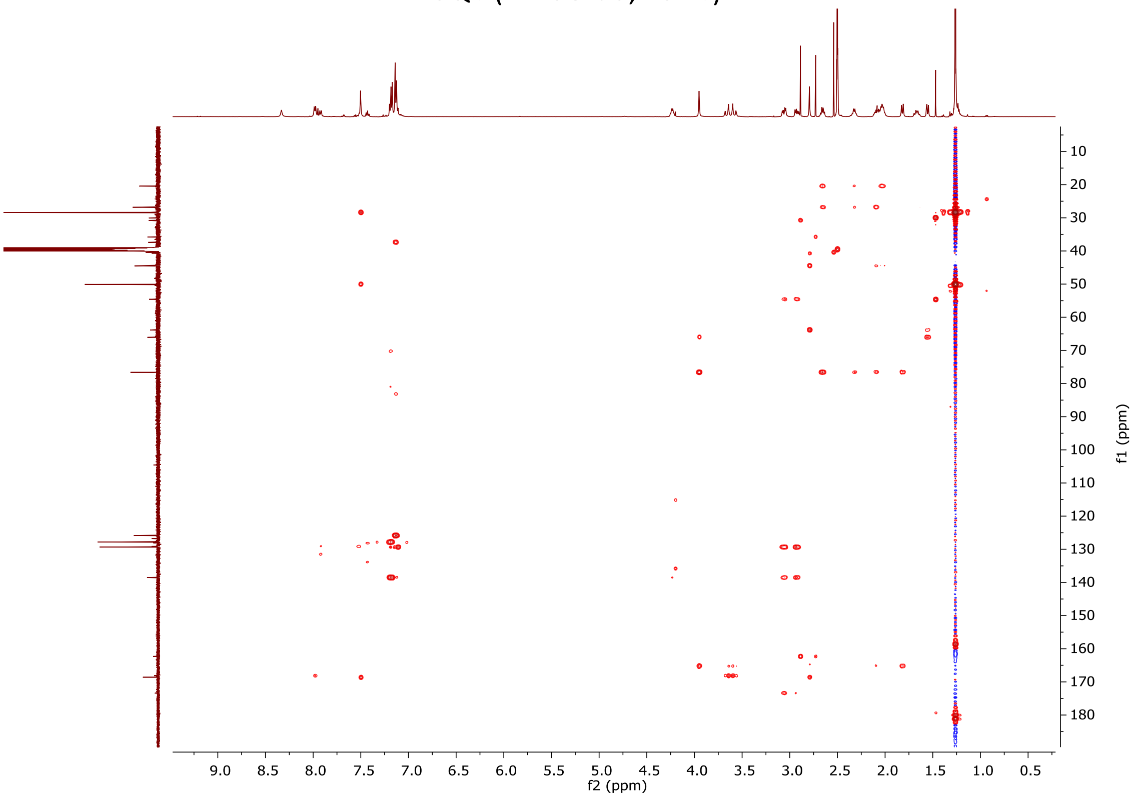

HMBC (DMSO-*d6*, 25 °C)

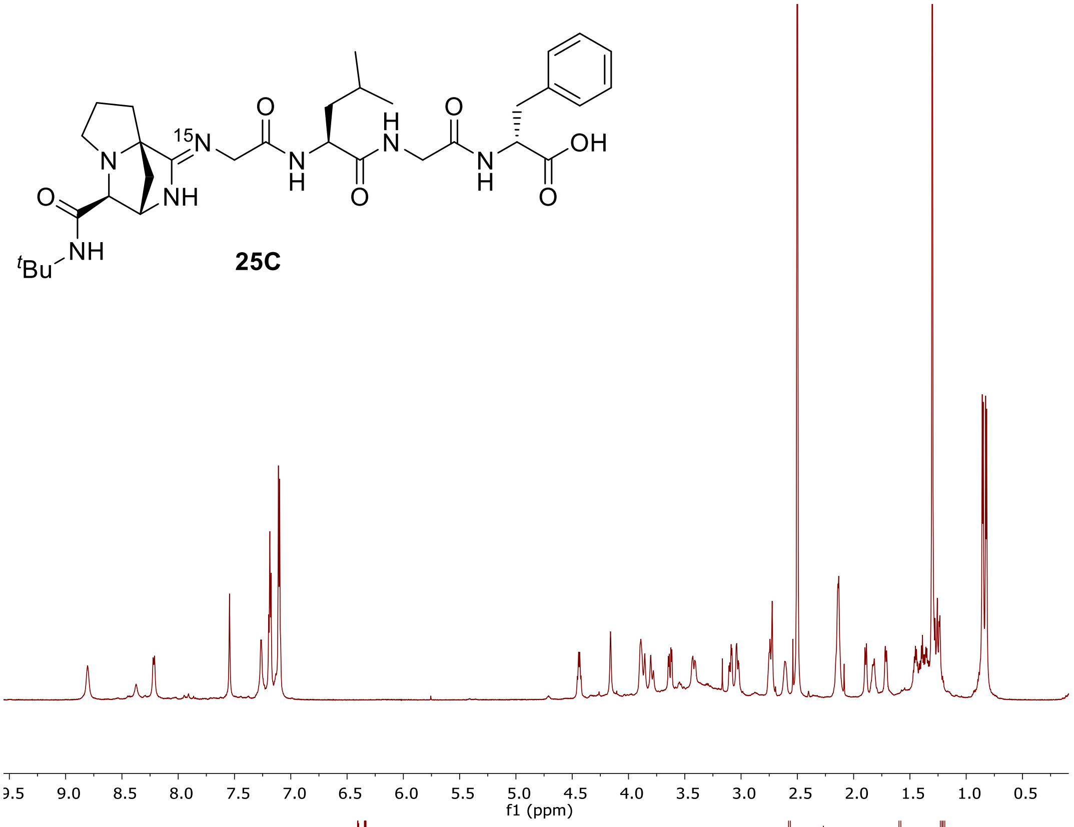

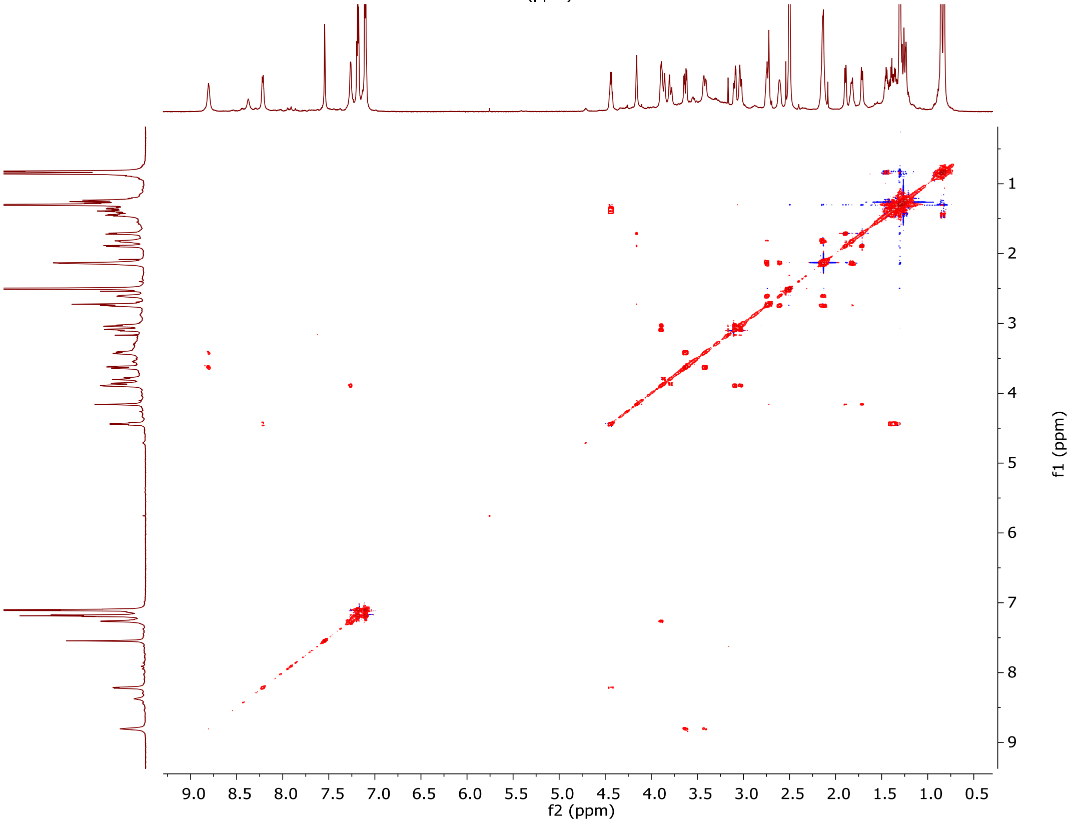

COSY (DMSO-*d6*, 25 ^{o}C)

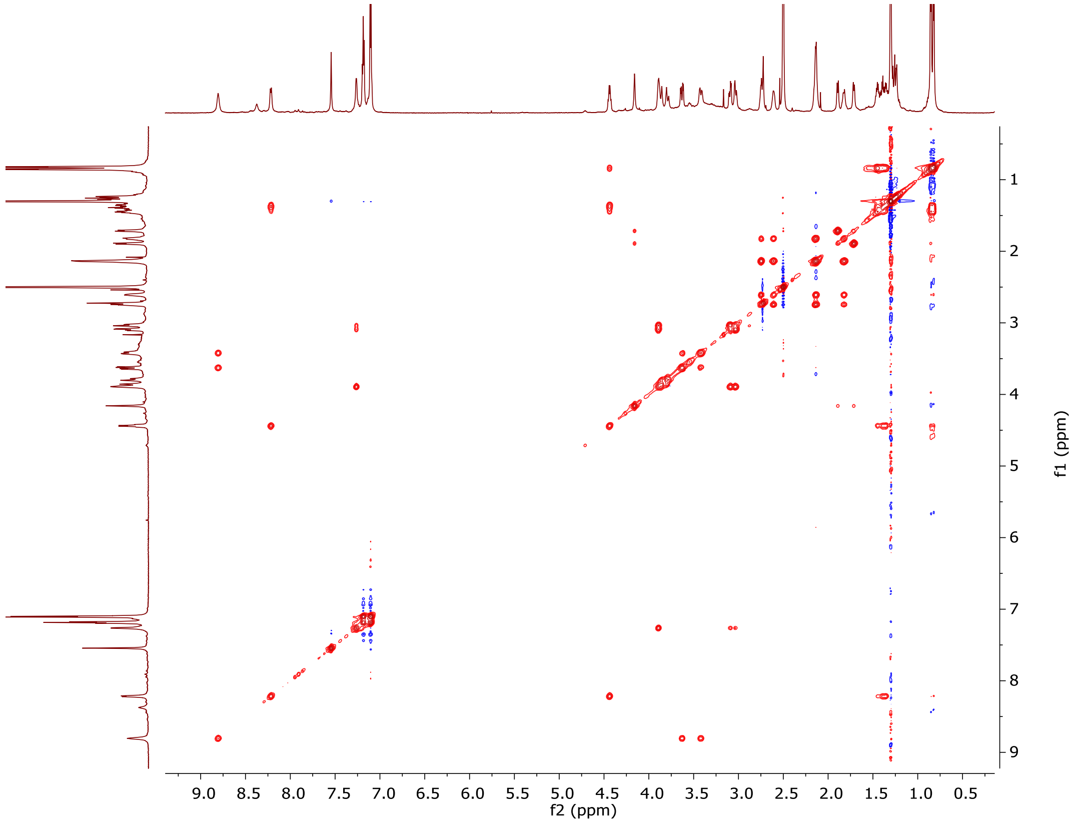

TOCSY (DMSO-*d6*, 25 °C)

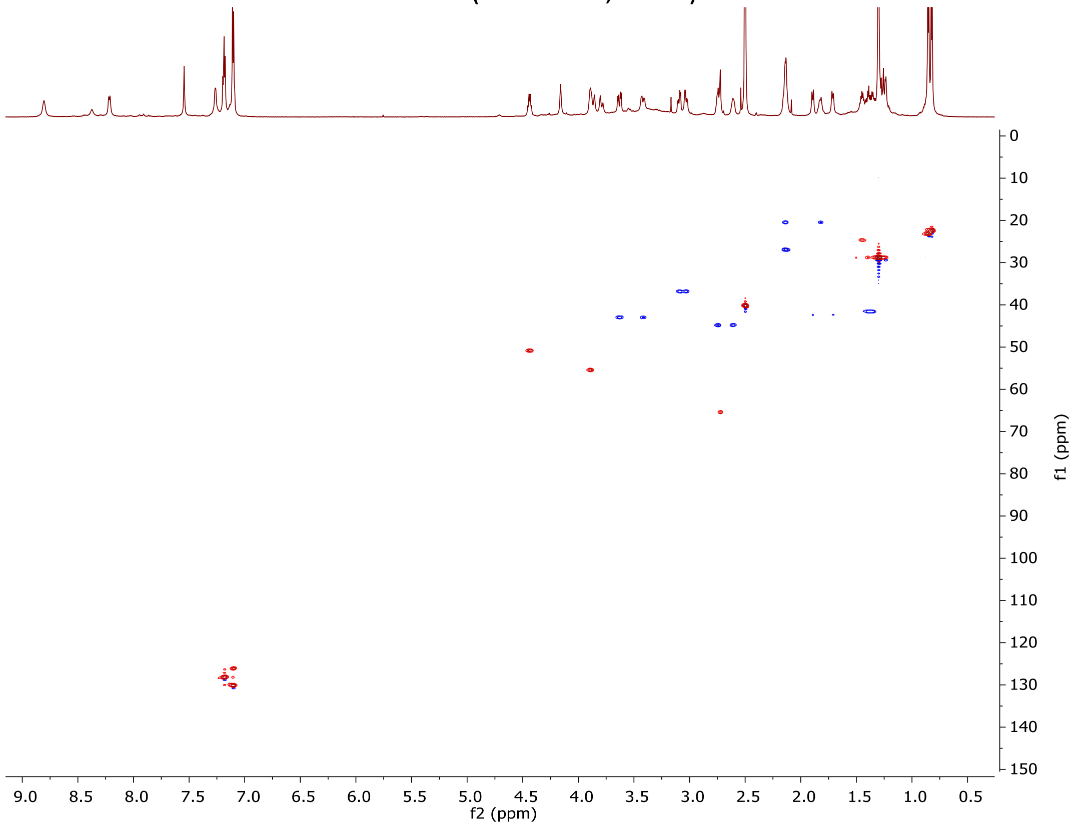

HSQC (DMSO-*d6*, 25 °C)

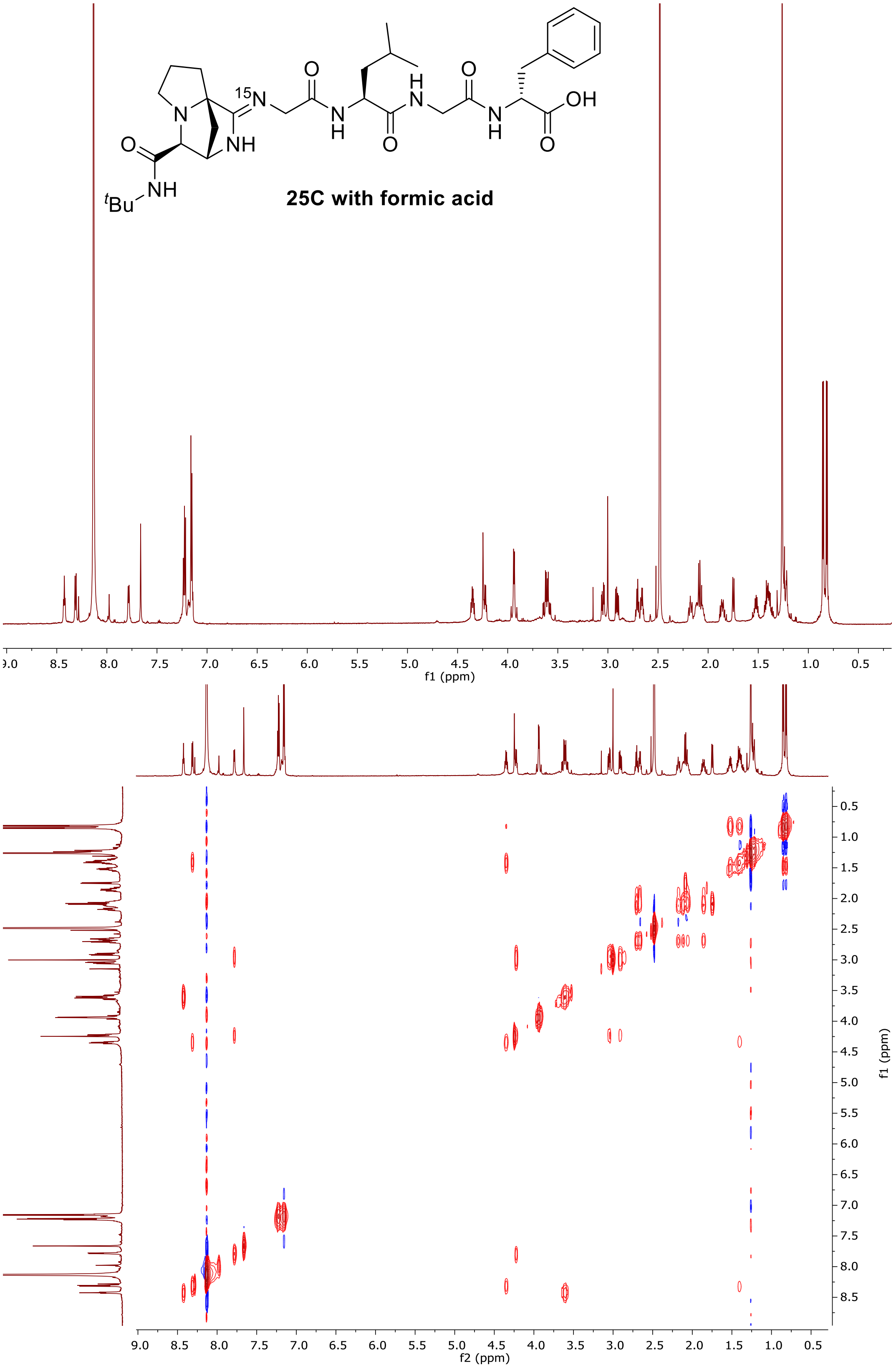

TOCSY (DMSO-*d6* + formic acid, 25 °C)

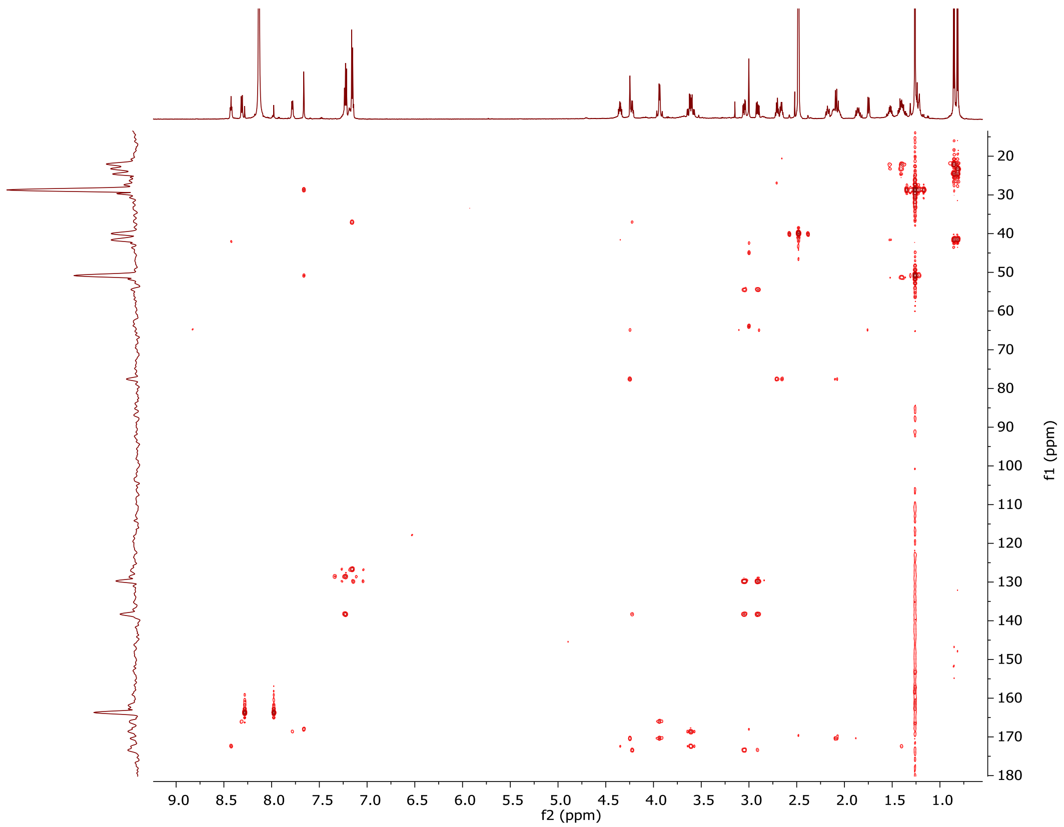

HMBC (DMSO-*d6* + formic acid, 25 ^{o}C)

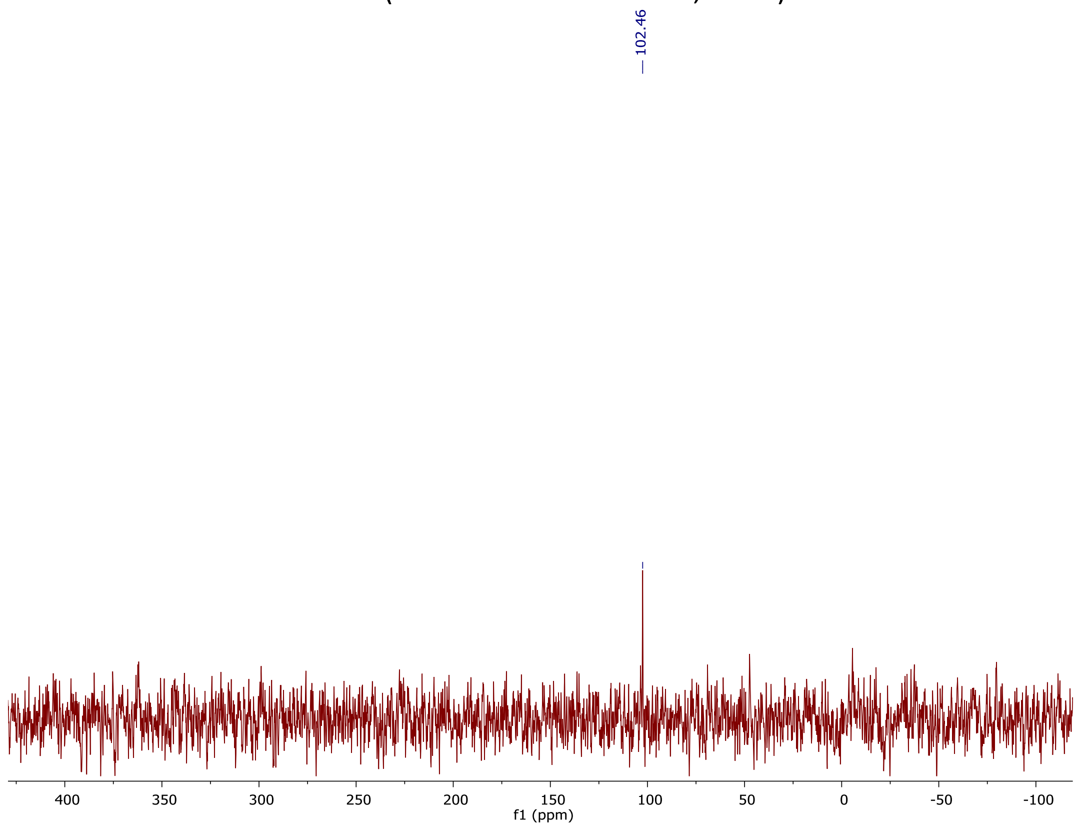

^{15}N (DMSO-*d6* + formic acid, 25 ^{o}C)

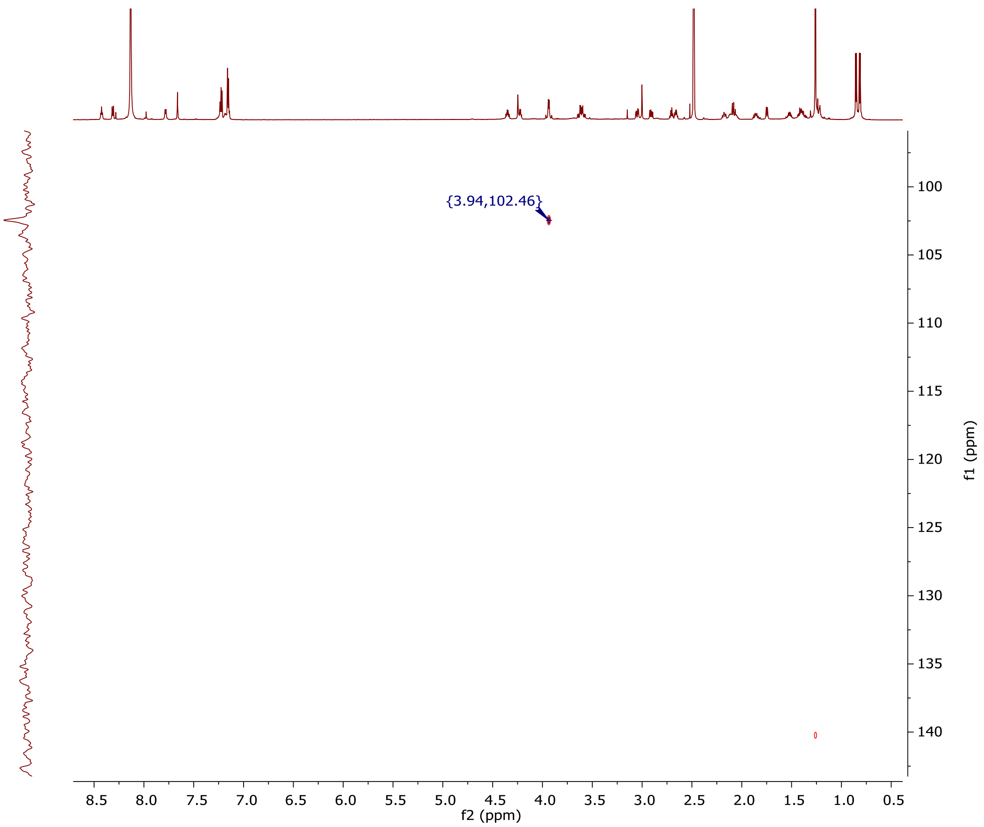

^{15}N-^{1}H HSQC (DMSO-*d6* + formic acid, 25 °C)

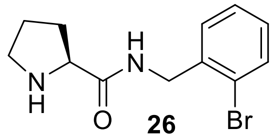

26

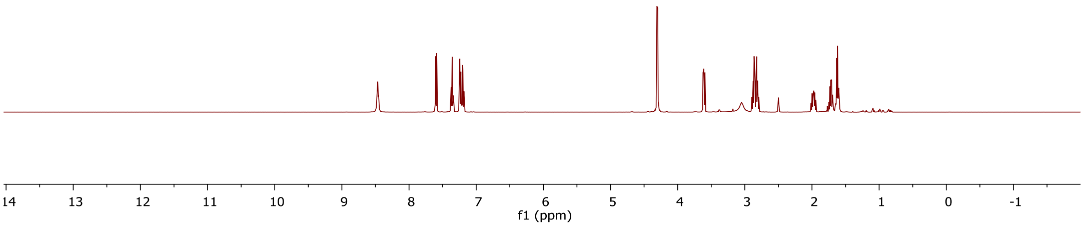

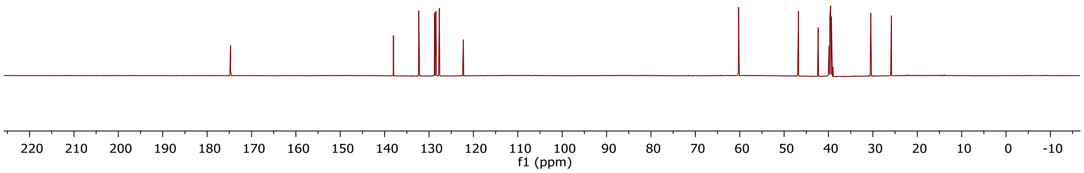

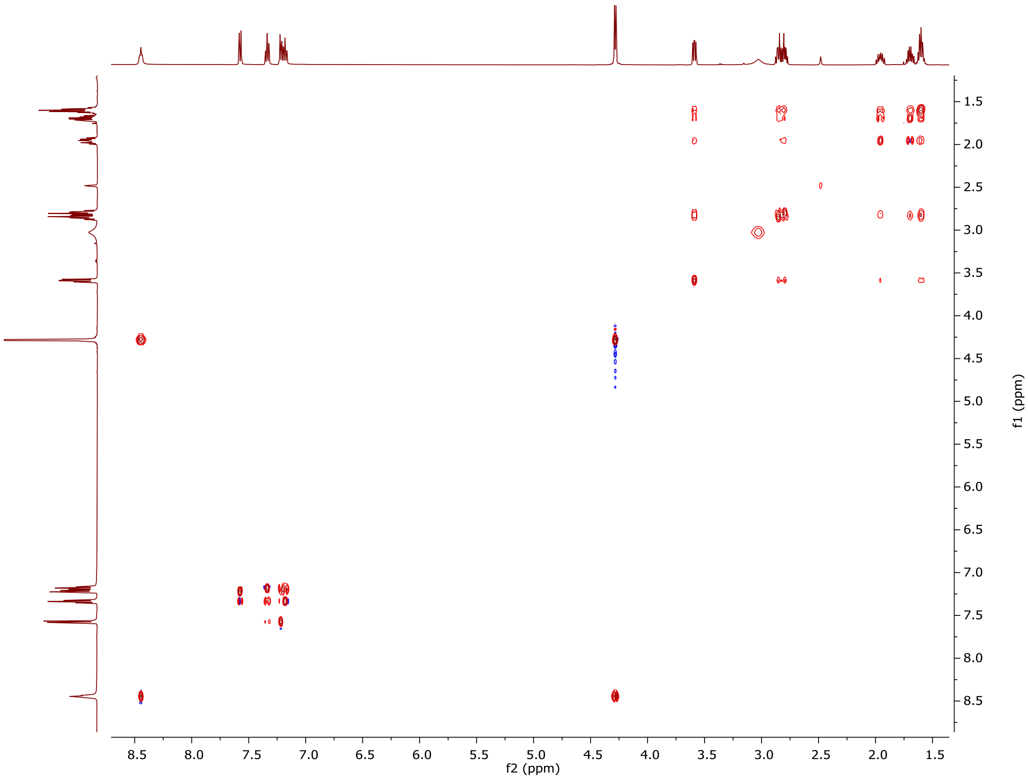

TOCSY (DMSO-*d6*, 25 ^{o}C)

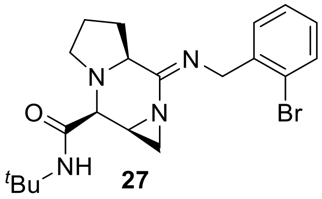
O
N
N
N
Br
NH
tBu

27

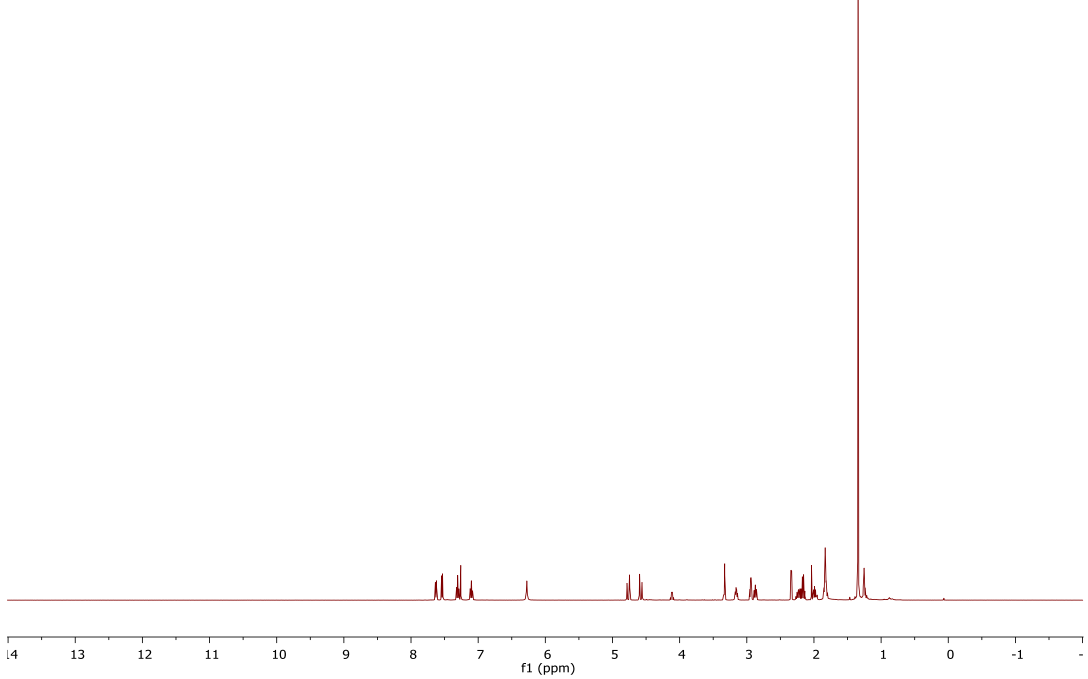
14
13
12
11
10
9
8
7
6
5
4
3
2
1
0
-1
f1 (ppm)

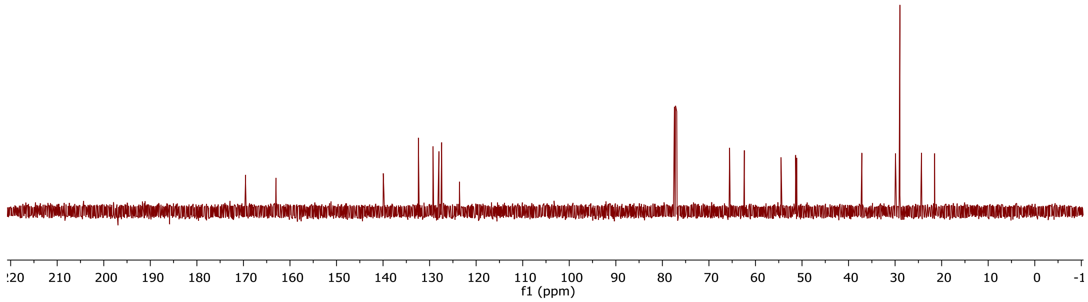
210
200
190
180
170
160
150
140
130
120
110
100
90
80
70
60
50
40
30
20
10
0
f1 (ppm)

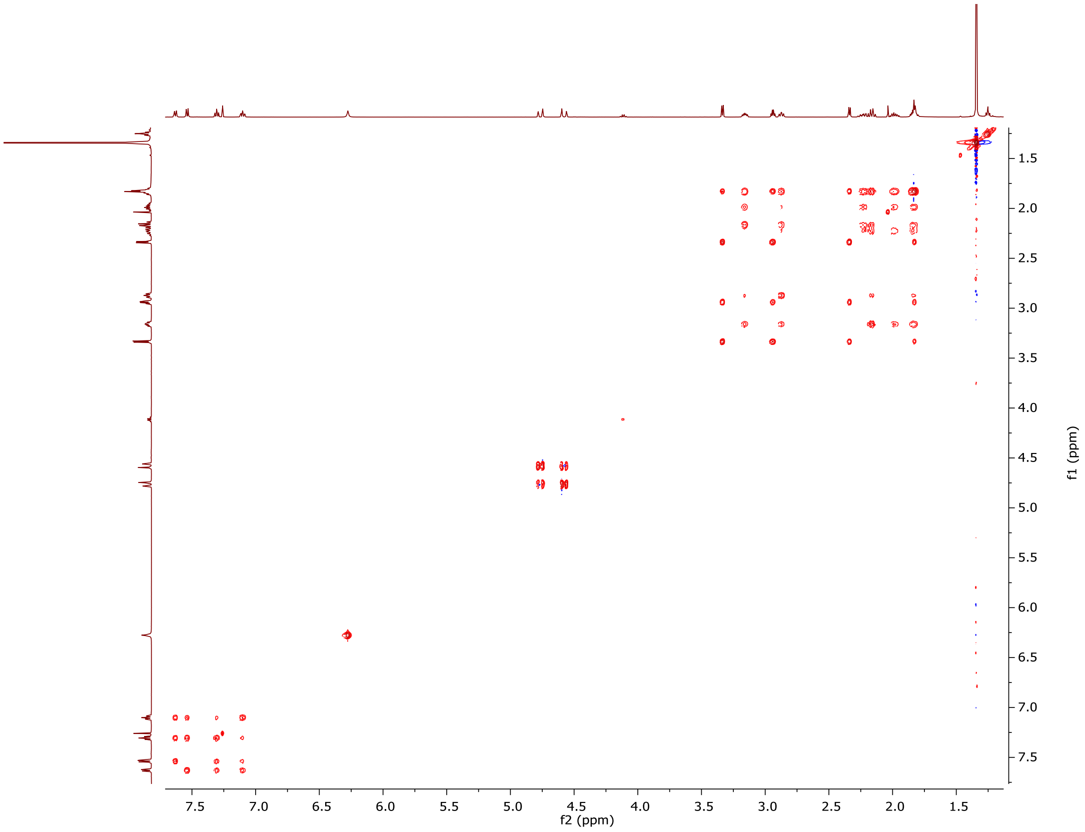

TOCSY (CDCl$_3$, 25 ^oC)

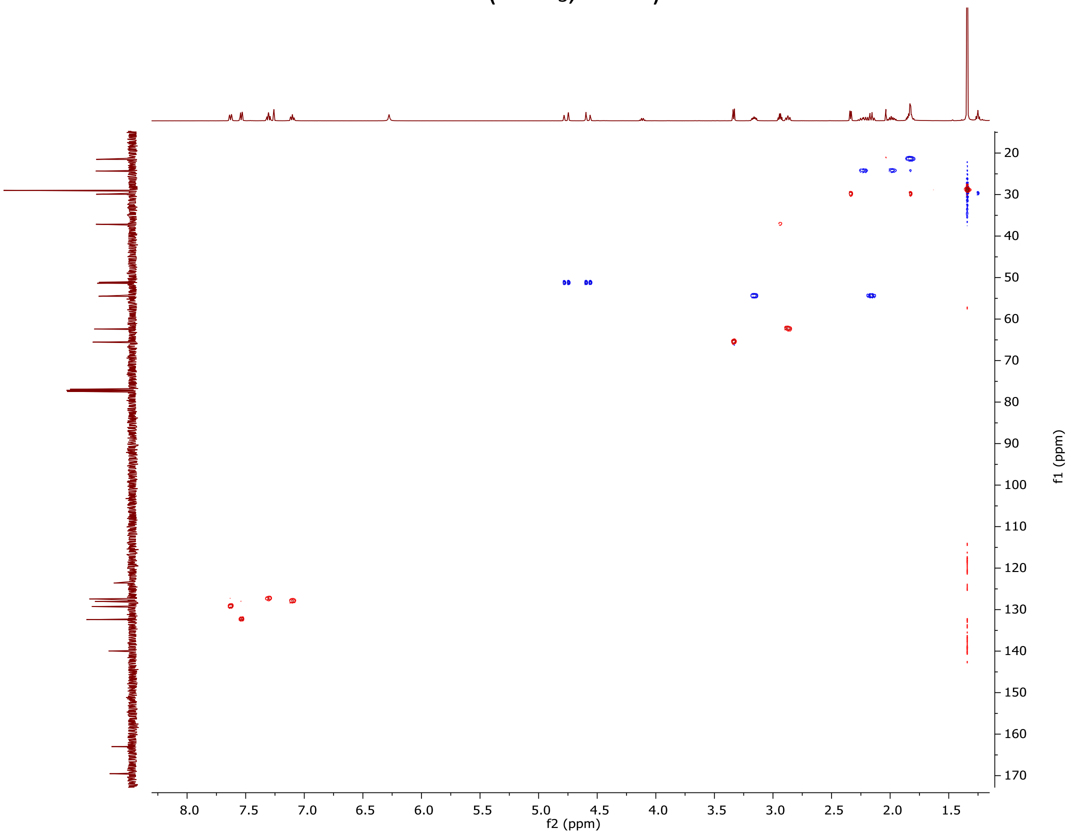

HSQC (CDCl$_3$, 25 ^oC)

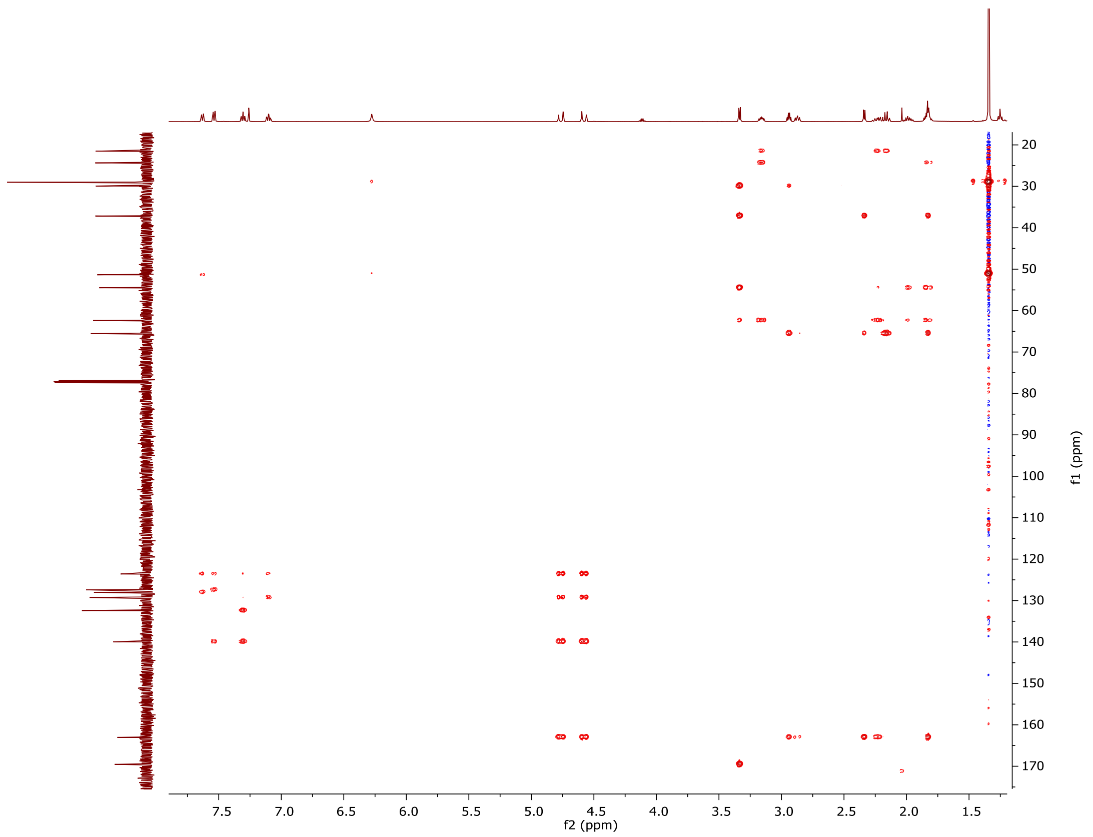

HMBC ($CDCl_3$, 25 °C)

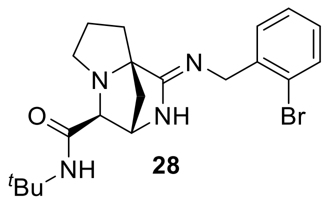

28

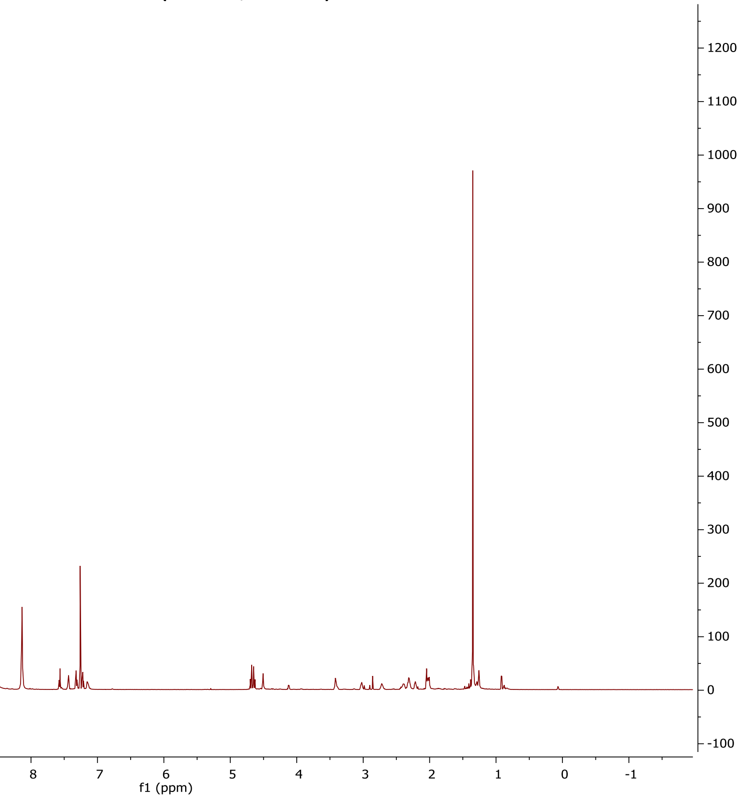

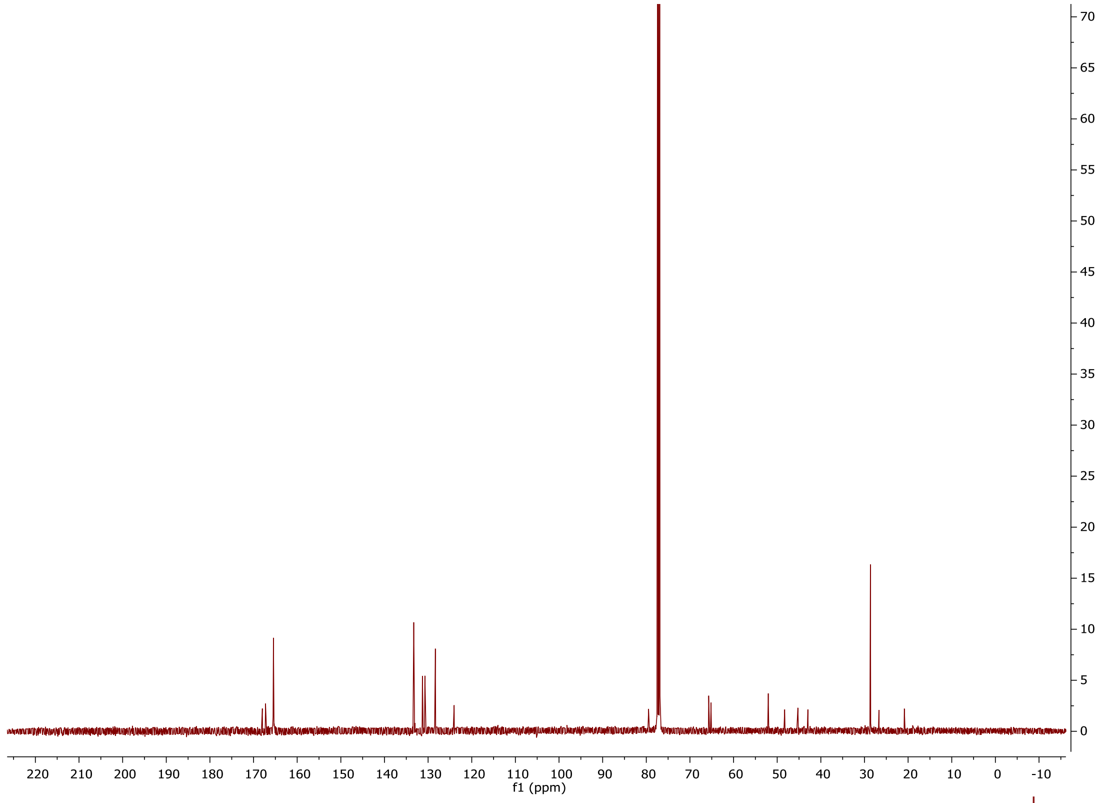

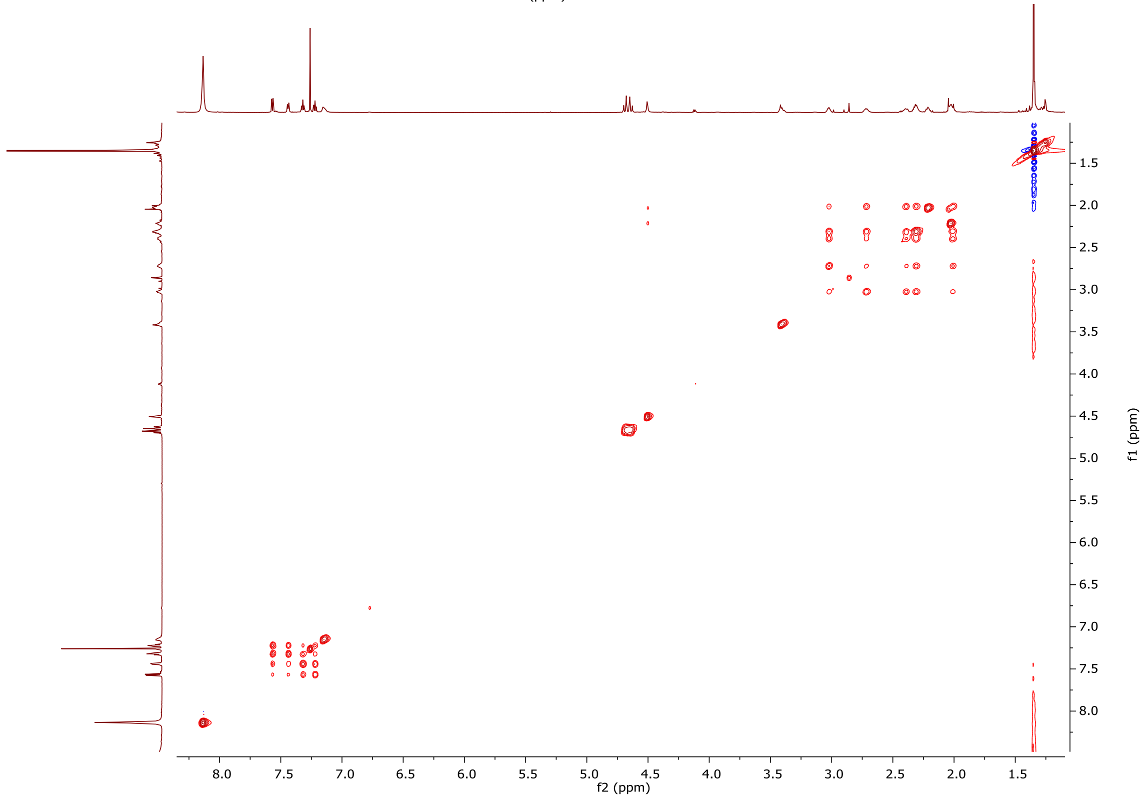

TOCSY ($CDCl_3$ + 0.3% formic acid, 25 ^{o}C)

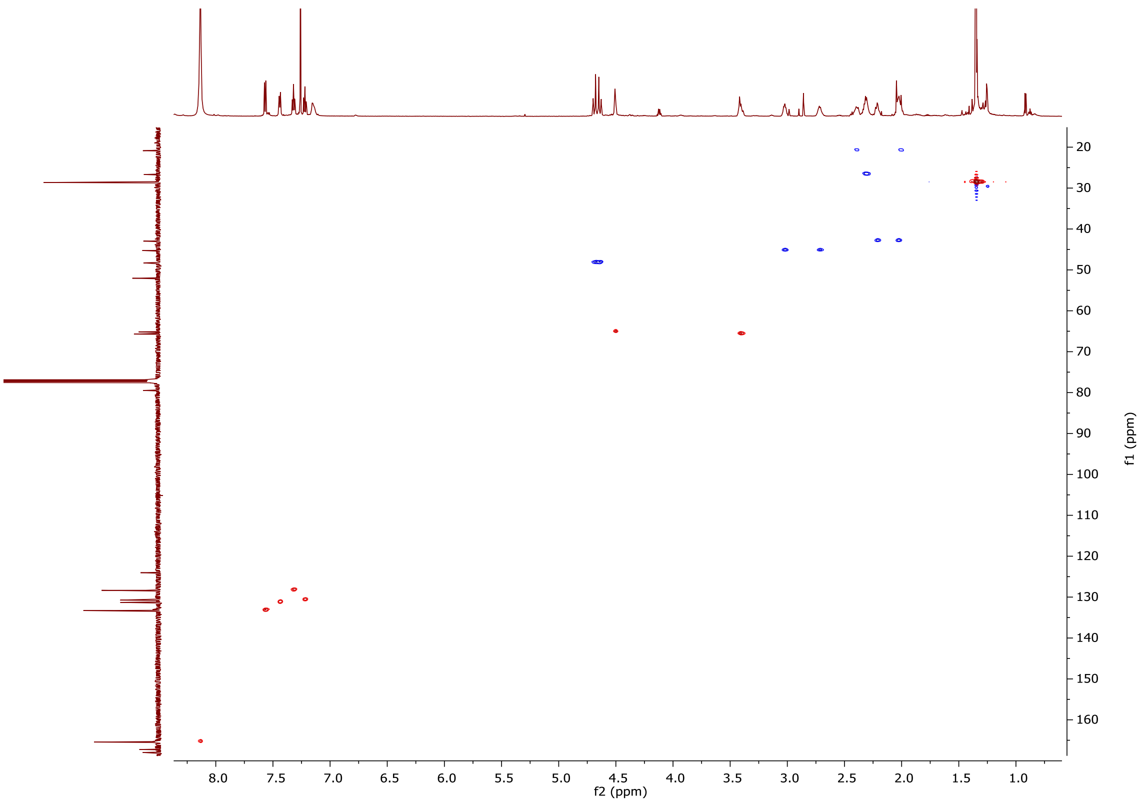

HSQC ($CDCl_3$ + 0.3% formic acid, 25 ^oC)

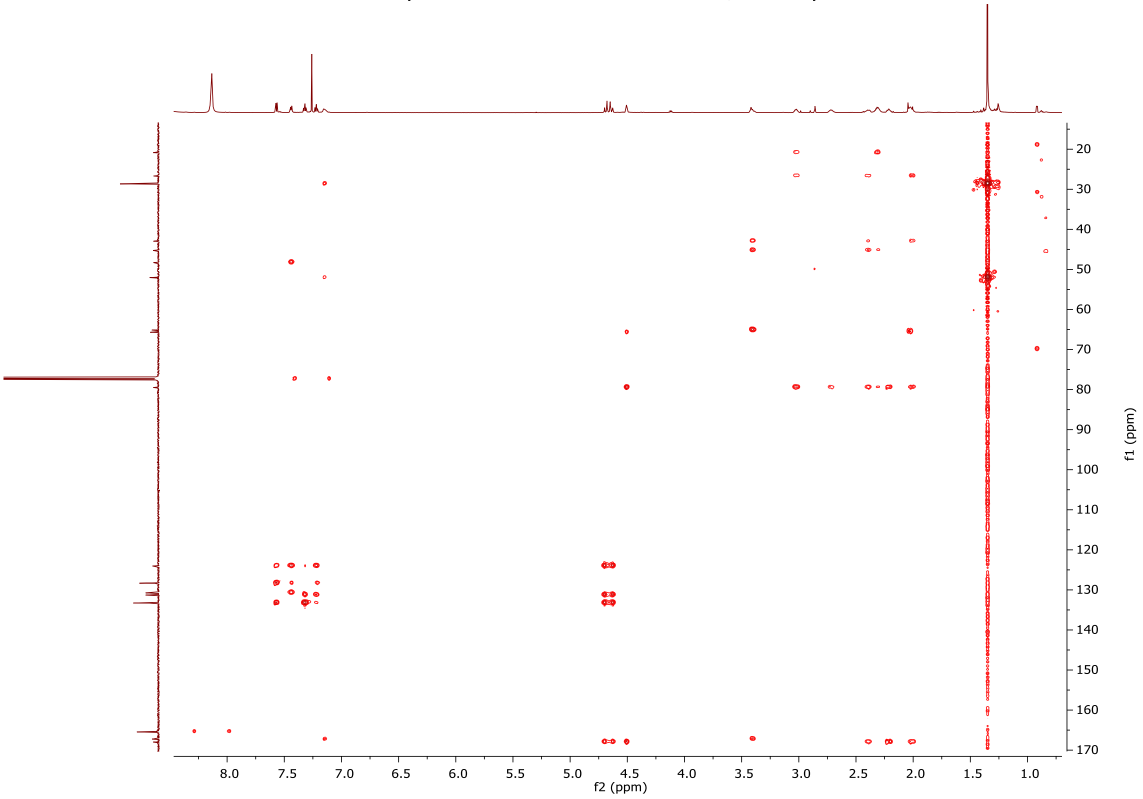

HMBC ($CDCl_3$ + 0.3% formic acid, 25 ^oC)

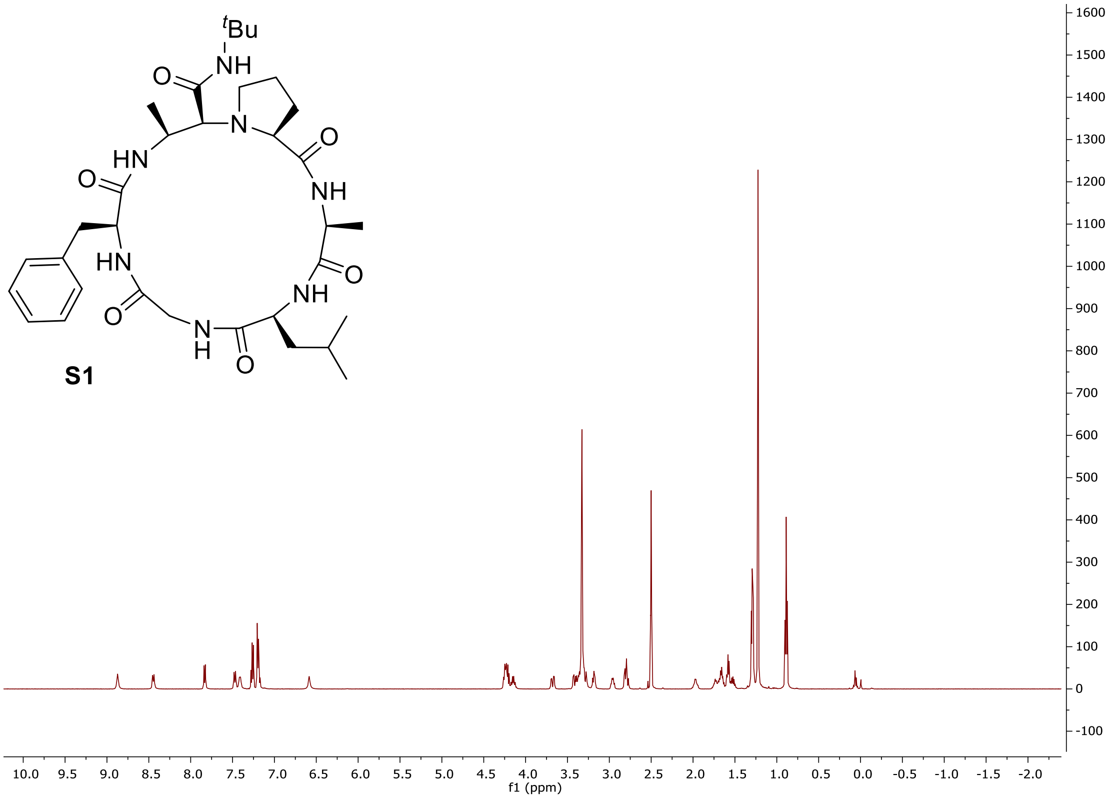
tBu
NH
O
N
O
HN
NH
O
HN
O
NH
O
N
H
O
S1
f1 (ppm)

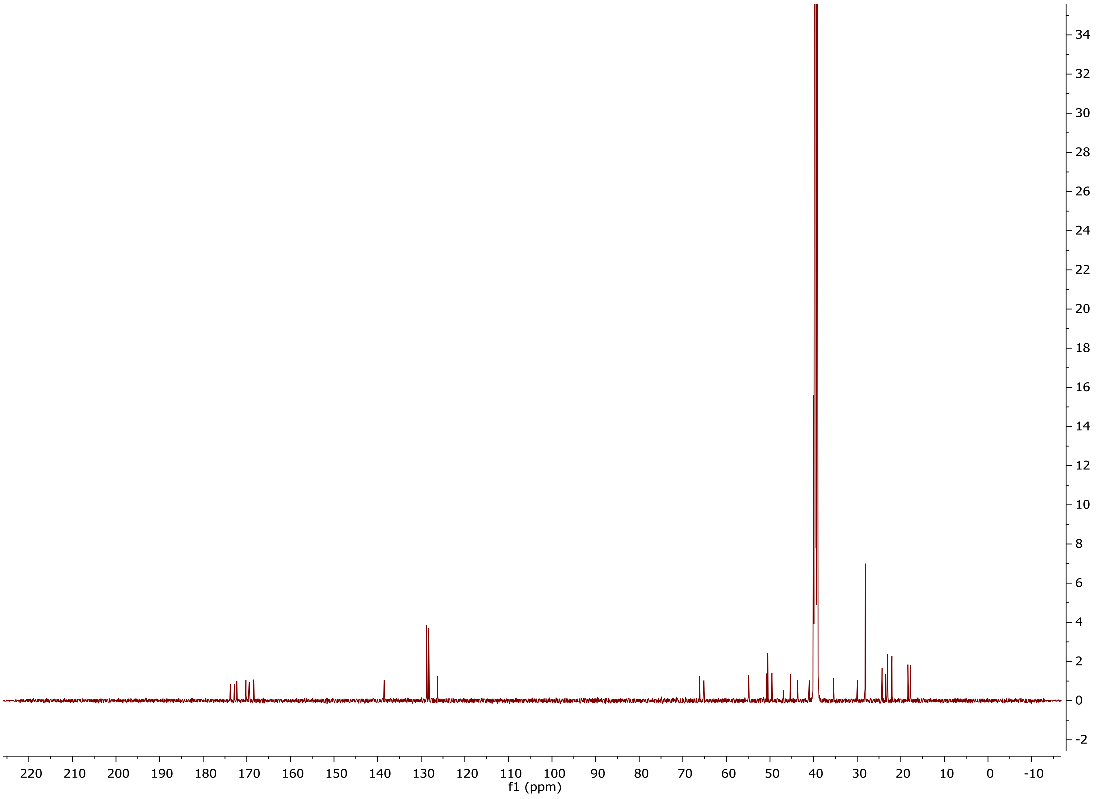
f1 (ppm)

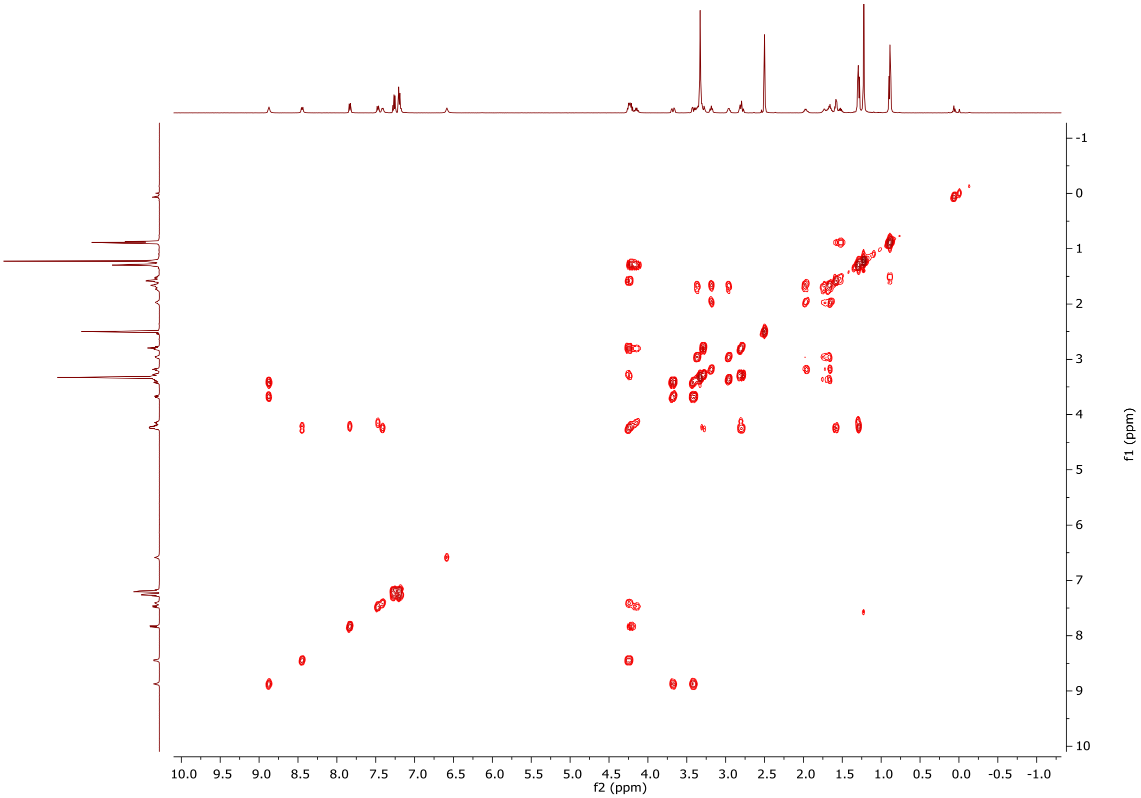

COSY (DMSO-*d6*, 25 ^{o}C)

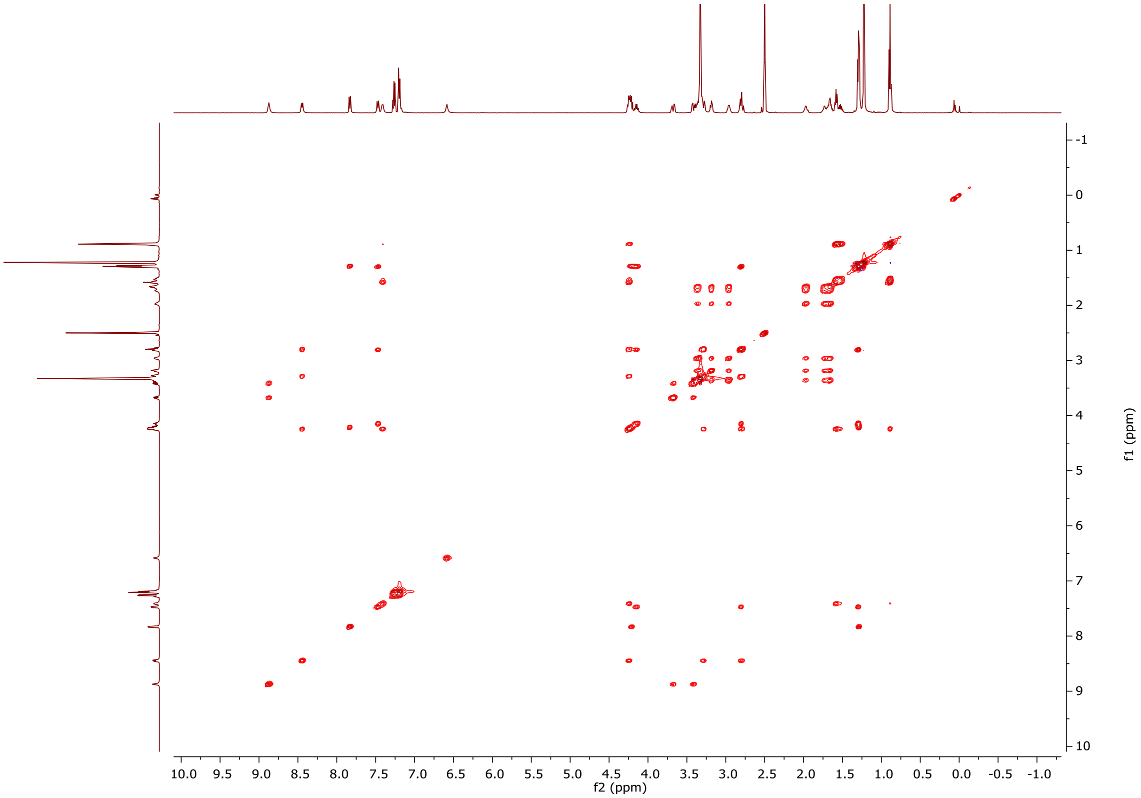

TOCSY (DMSO-*d6*, 25 ^{o}C)

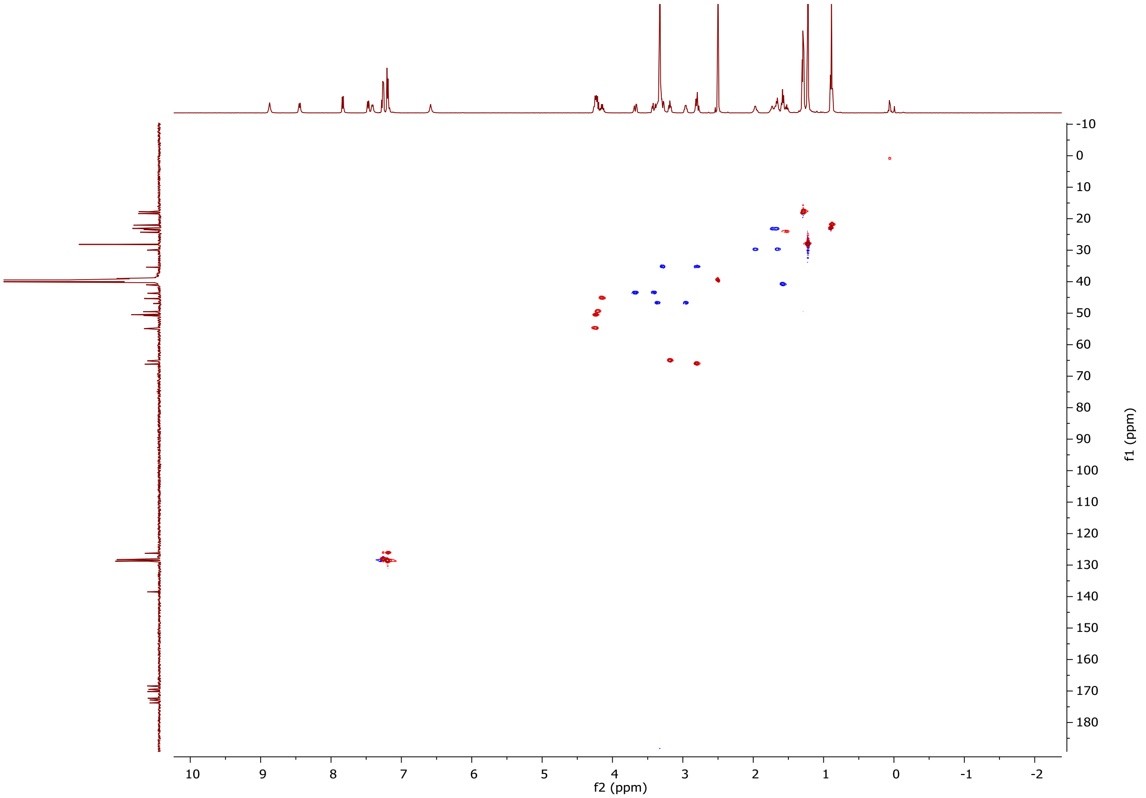

HSQC (DMSO-*d6*, 25 ^{o}C)

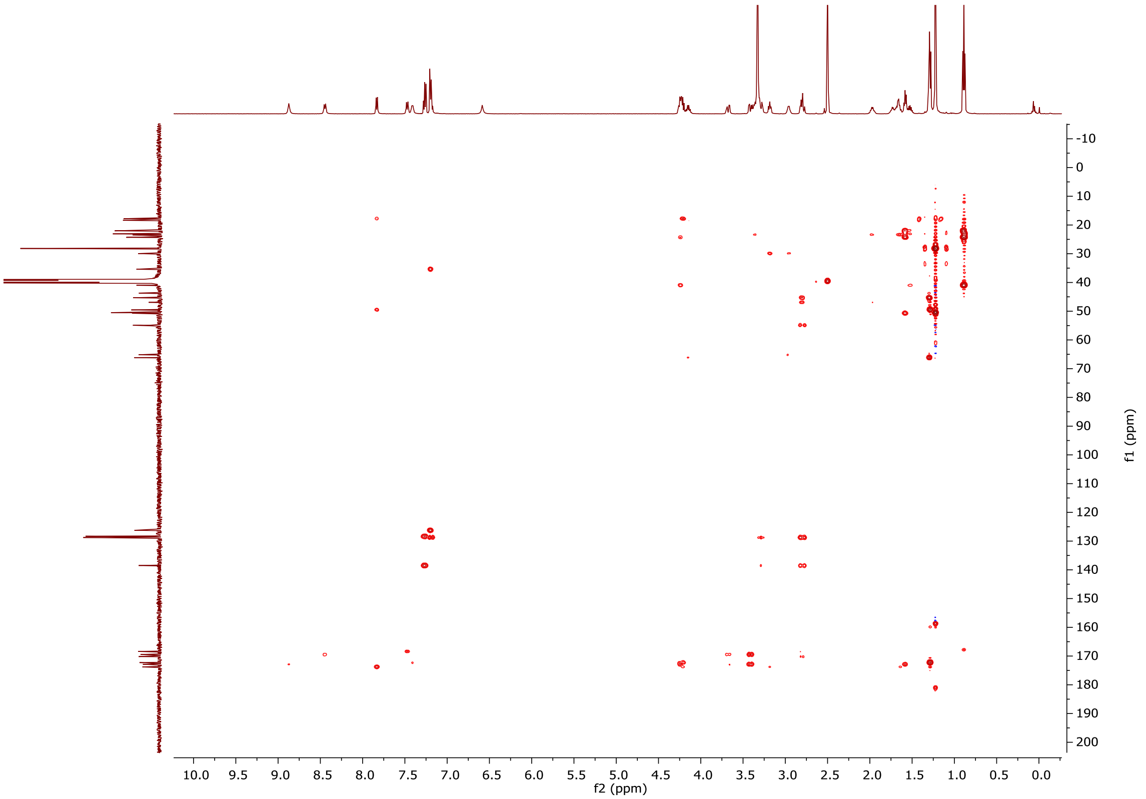

HMBC (DMSO-*d6*, 25 ^{o}C)

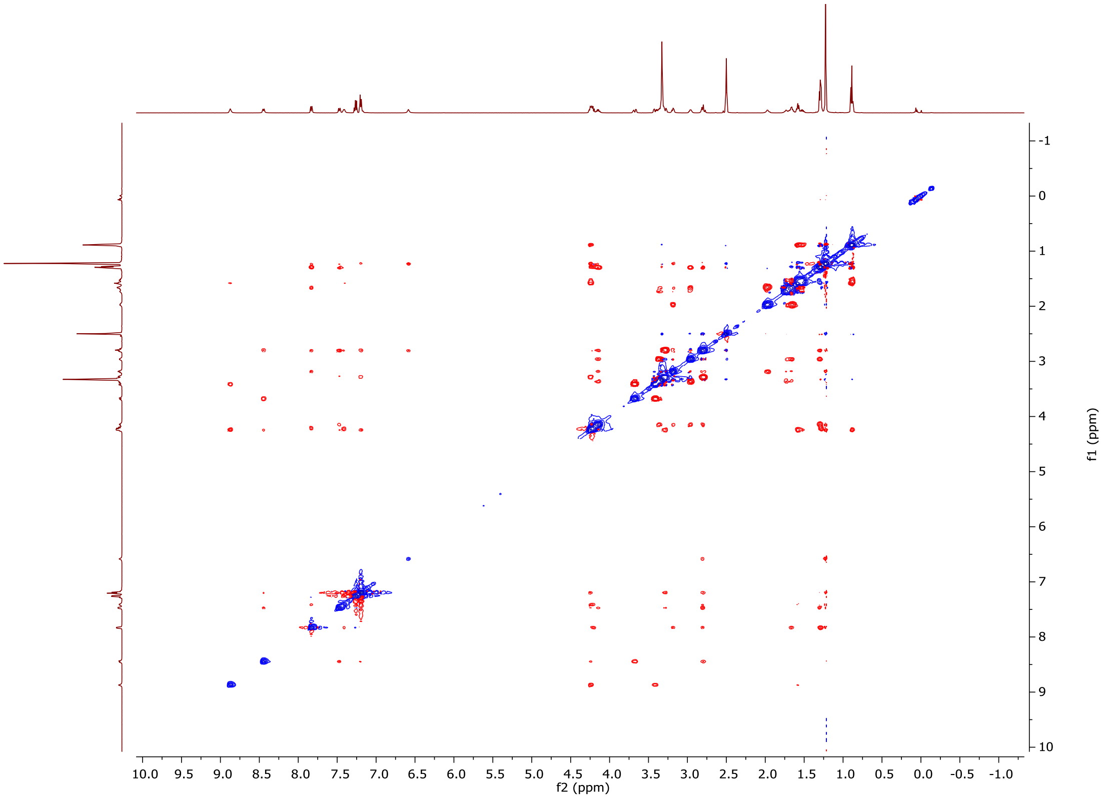

ROESY (DMSO-d_6) @ 25°C, 250 ms mixing time

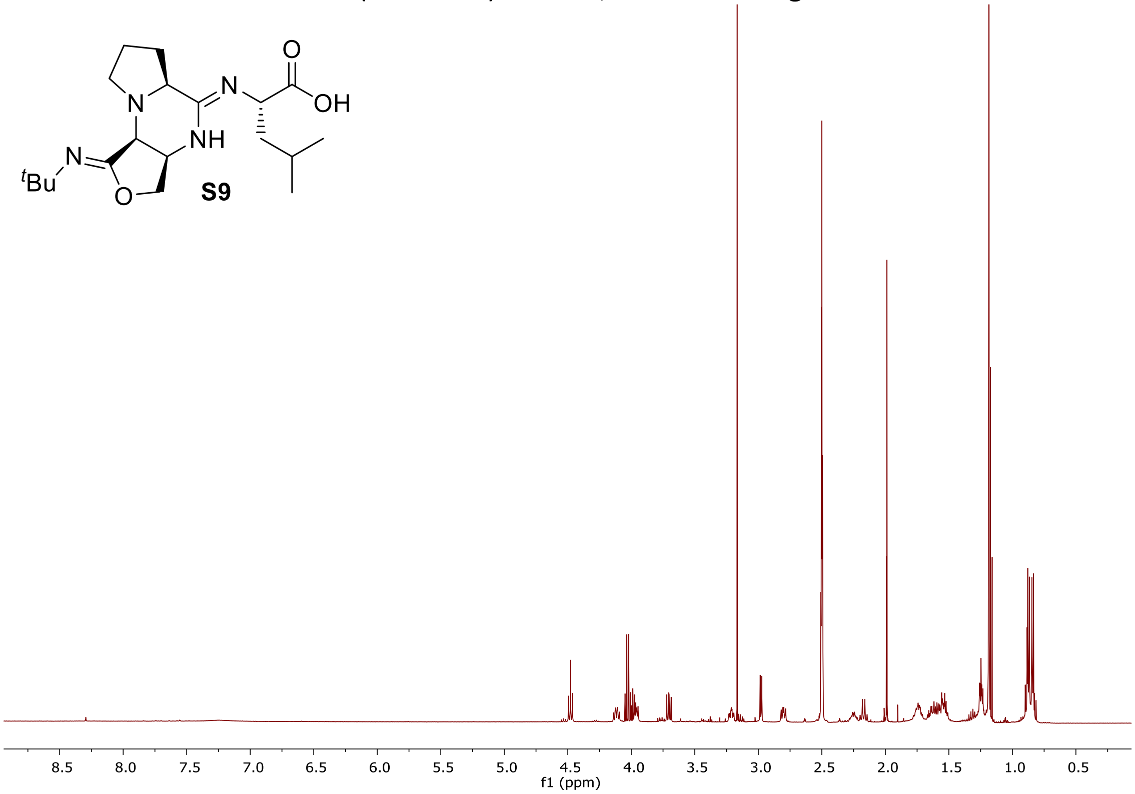

S9

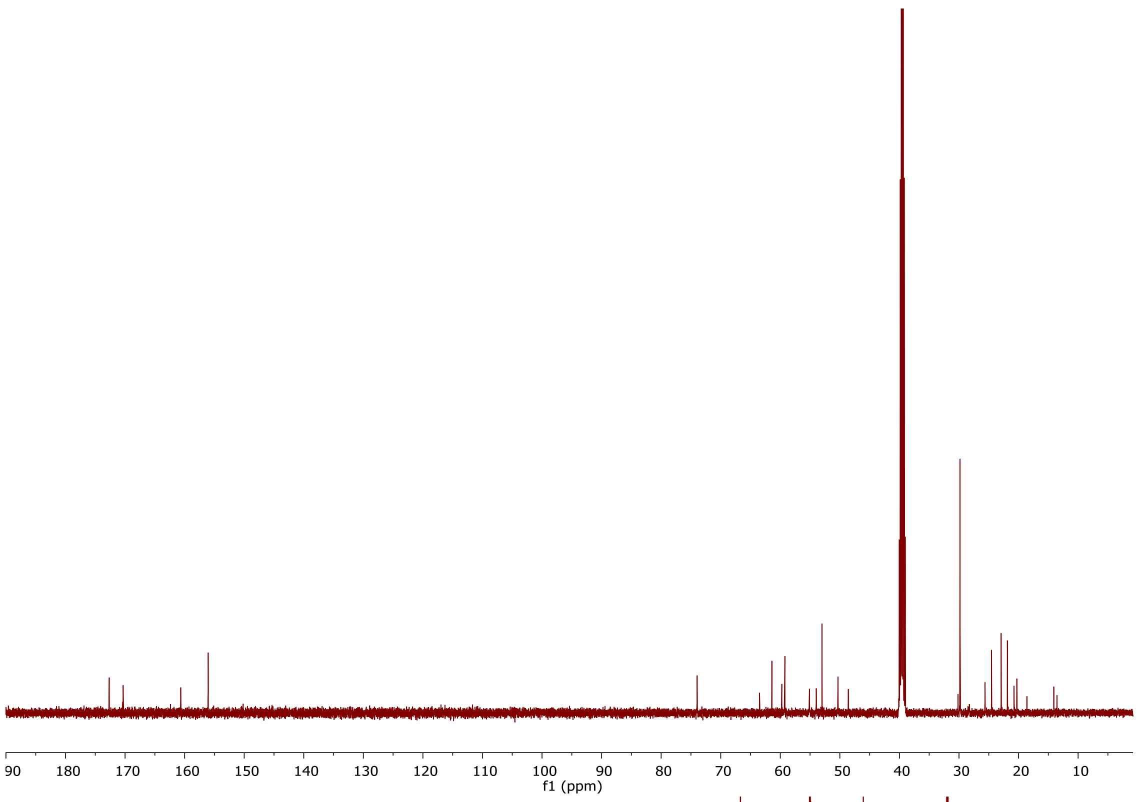

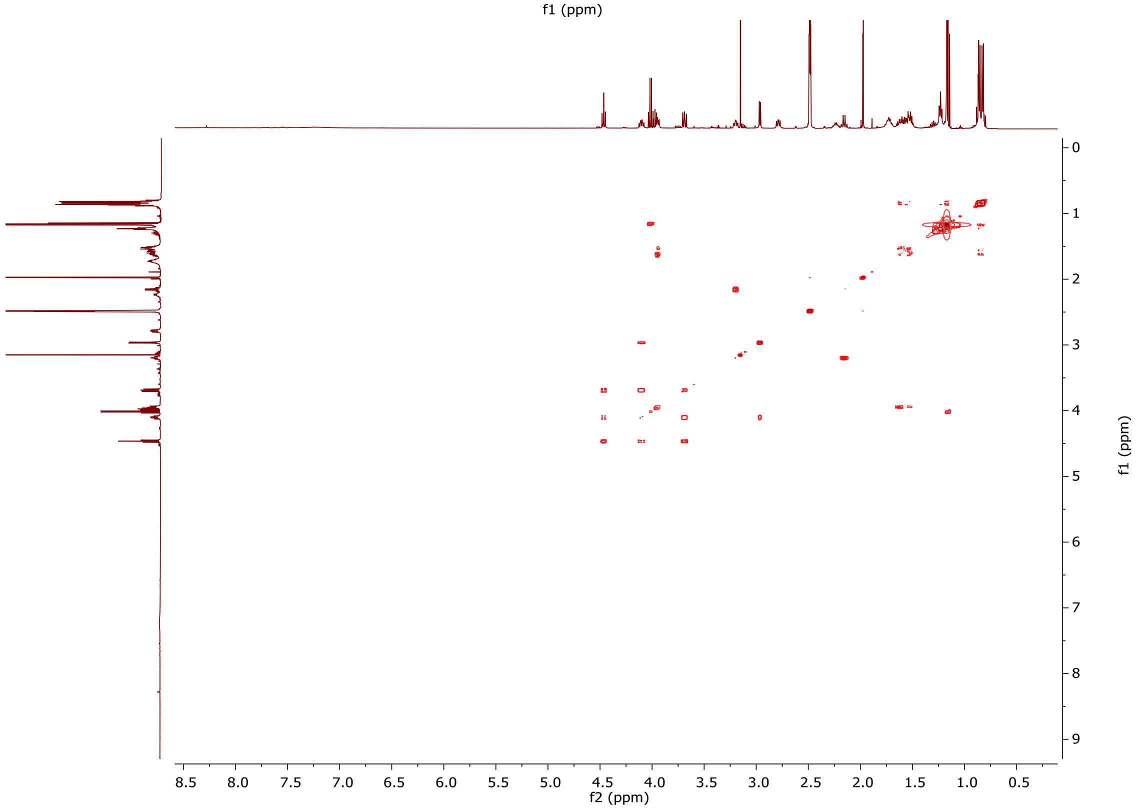

COSY (DMSO-*d6*, 25 ºC)

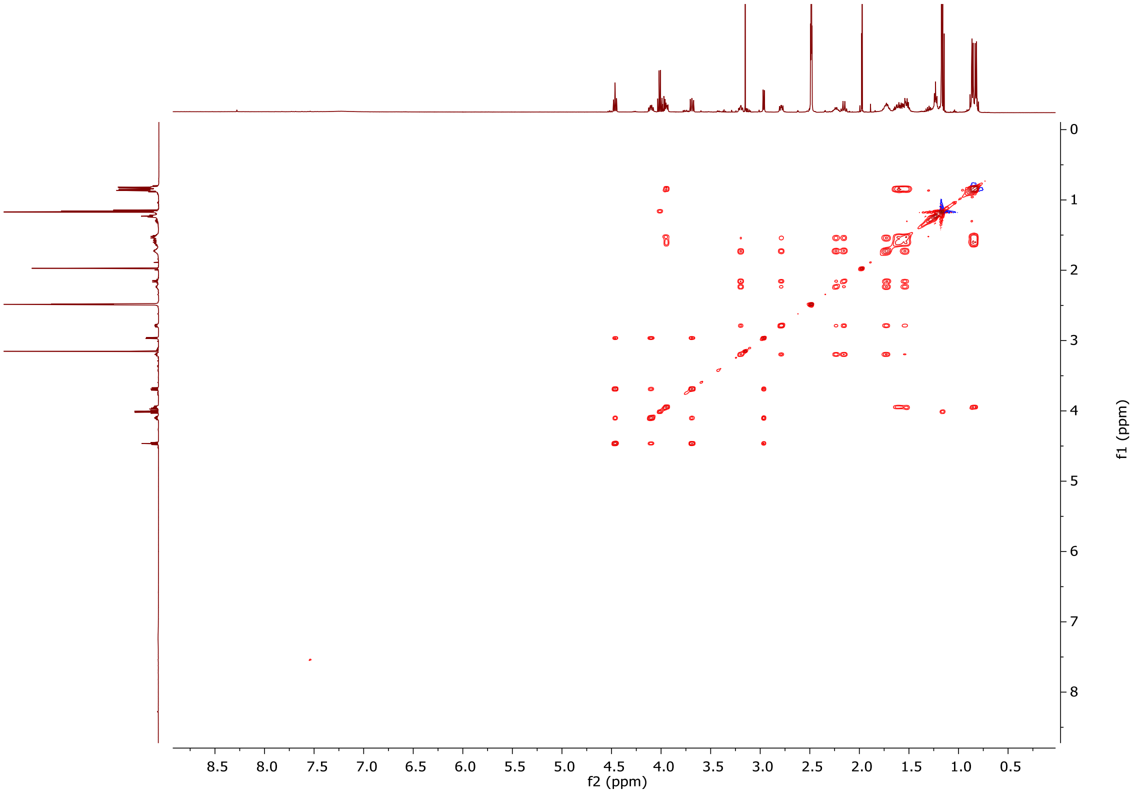

TOCSY (DMSO-*d6*, 25 ^{o}C)

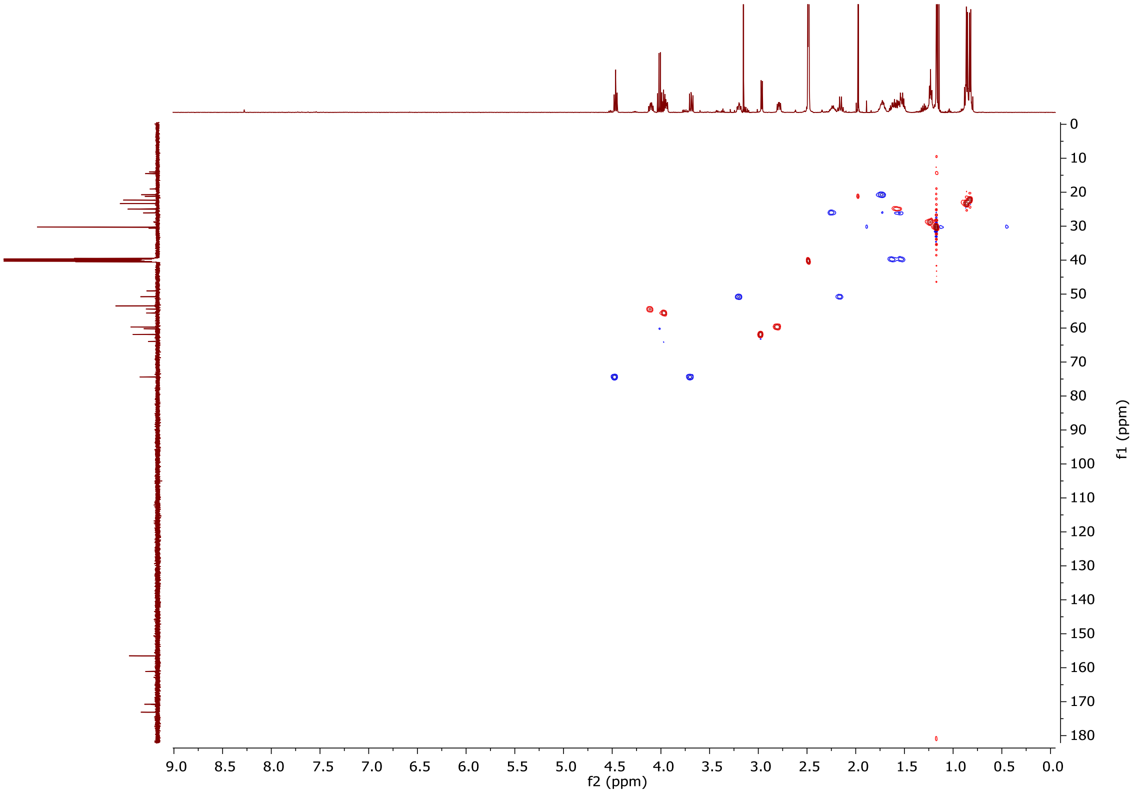

HSQC (DMSO-*d6*, 25 ^{o}C)

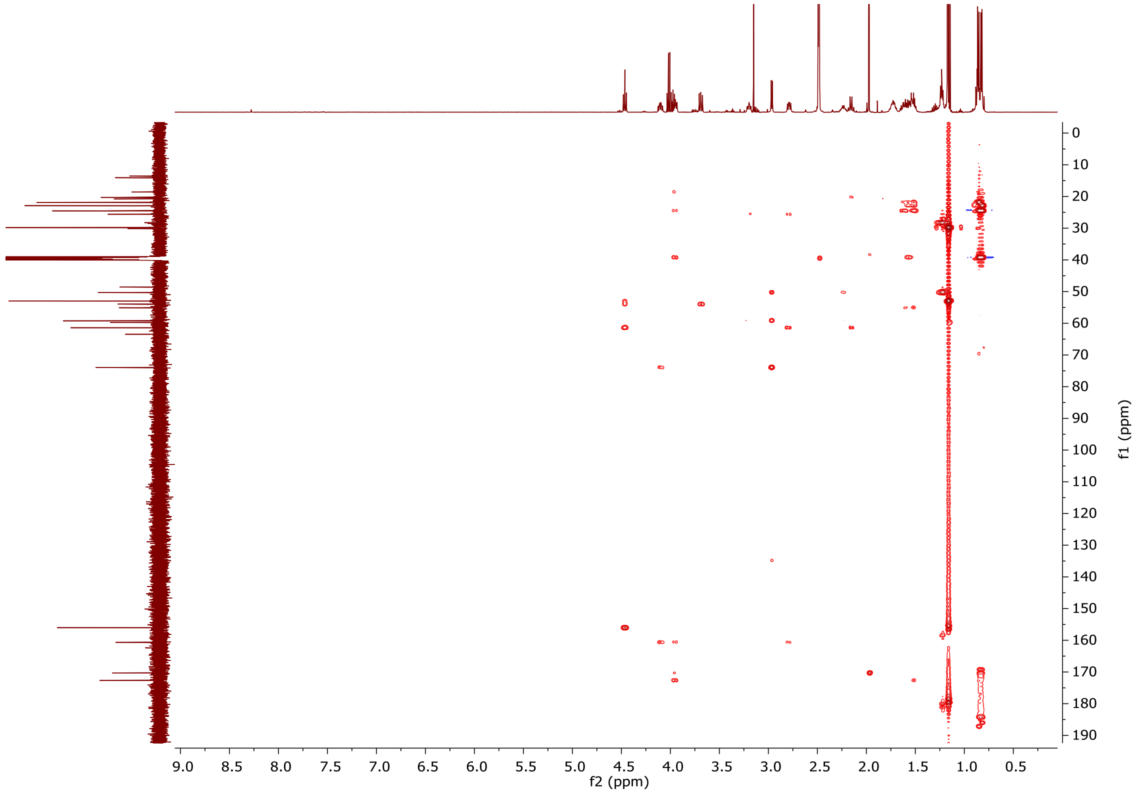

HMBC (DMSO-*d6*, 25 °C)

Crystallographic data

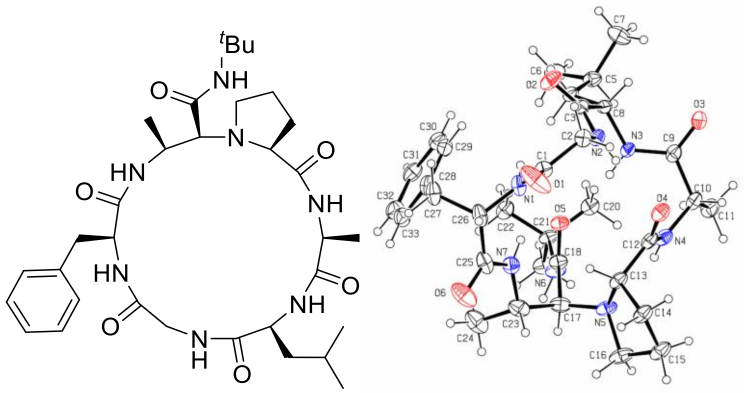

c*[PALGF] (S1)

Table 1. Crystal data and structure refinement for d13160_sq_d_x.

Identification code	d13160_sq_d	
Empirical formula	C33 H53 N7 O7	
Formula weight	659.82	
Temperature	147(2) K	
Wavelength	1.54178 Å	
Crystal system	Monoclinic	
Space group	P 21	
Unit cell dimensions	a = 10.0804(3) Å	α= 90°.
	b = 9.9982(3) Å	β= 103.2990(17)°.
	c = 19.7575(6) Å	γ = 90°.
Volume	1937.88(10) $Å^3$	
Z	2	
Density (calculated)	1.131 Mg/m^3	
Absorption coefficient	0.654 mm^{-1}	
F(000)	712	
Crystal size	0.100 x 0.060 x 0.050 mm^3	
Theta range for data collection	2.298 to 66.745°.	
Index ranges	-11<=h<=11, -11<=k<=11, -23<=l<=23	
Reflections collected	36769	
Independent reflections	6593 [R(int) = 0.0327]	
Completeness to theta = 67.679°	96.7 %	
Absorption correction	Semi-empirical from equivalents	
Max. and min. transmission	0.7528 and 0.7079	
Refinement method	Full-matrix least-squares on F^2	

Data / restraints / parameters	6593 / 1 / 425
Goodness-of-fit on F^2	1.065
Final R indices [I>2sigma(I)]	R1 = 0.0425, wR2 = 0.1073
R indices (all data)	R1 = 0.0444, wR2 = 0.1089
Absolute structure parameter	0.05(7)
Extinction coefficient	n/a
Largest diff. peak and hole	0.503 and -0.258 e.$Å^{-3}$

Table 2. Atomic coordinates (x 10^4) and equivalent isotropic displacement parameters ($Å^2$x 10^3) for d13160_sq_d_x. U(eq) is defined as one third of the trace of the orthogonalized U^{ij} tensor.

	x	y	z	U(eq)
O(1)	7232(3)	2290(4)	5999(2)	93(1)
O(2)	9269(3)	3352(2)	8246(1)	55(1)
O(3)	10819(2)	7892(2)	8048(1)	53(1)
O(4)	8325(2)	6976(2)	6422(1)	45(1)
O(5)	5819(2)	6668(2)	7721(1)	37(1)
O(6)	2933(3)	3577(4)	5981(2)	75(1)
N(1)	6455(3)	3284(3)	6841(1)	41(1)
N(2)	8901(3)	4661(2)	7300(1)	36(1)
N(3)	8810(2)	6911(2)	8064(1)	32(1)
N(4)	7496(3)	8604(3)	7000(1)	40(1)
N(5)	4875(3)	8121(3)	6366(1)	44(1)
N(6)	4387(3)	8242(3)	7983(1)	43(1)
N(7)	4258(3)	5034(3)	6696(1)	42(1)
C(1)	7435(3)	2998(3)	6519(2)	39(1)
C(2)	8835(3)	3578(3)	6811(2)	40(1)
C(3)	9176(3)	4466(3)	7988(2)	34(1)
C(4)	8908(3)	5527(3)	9112(1)	35(1)
C(5)	9463(3)	6537(3)	9691(2)	41(1)
C(6)	8747(4)	6331(4)	10289(2)	54(1)
C(7)	10986(4)	6427(5)	9955(2)	63(1)
C(8)	9407(3)	5725(3)	8449(1)	32(1)
C(9)	9574(3)	7874(3)	7866(2)	39(1)
C(10)	8832(4)	8990(3)	7404(2)	40(1)
C(11)	8745(4)	10230(3)	7834(2)	48(1)
C(12)	7344(3)	7625(3)	6530(1)	40(1)
C(13)	5911(3)	7316(3)	6130(1)	40(1)
C(14)	5730(4)	7674(3)	5349(2)	47(1)
C(15)	5068(4)	9045(4)	5302(2)	55(1)
C(16)	4088(4)	8889(4)	5764(2)	55(1)
C(17)	4015(3)	7502(4)	6795(2)	47(1)
C(18)	4830(3)	7428(3)	7552(1)	38(1)

C(19)	4856(3)	8312(3)	8755(1)	36(1)
C(20)	6319(3)	8809(3)	8942(2)	42(1)
C(21)	3921(4)	9309(4)	8995(2)	47(1)
C(22)	4723(4)	6941(3)	9061(2)	47(1)
C(23)	3311(4)	6170(4)	6546(2)	52(1)
C(24)	2100(4)	5952(6)	6866(2)	73(1)
C(25)	3995(3)	3854(4)	6397(2)	50(1)
C(26)	5085(3)	2756(4)	6604(2)	48(1)
C(27)	4687(4)	1763(4)	7123(2)	51(1)
C(28)	4524(3)	2395(3)	7796(2)	41(1)
C(29)	5625(3)	2632(3)	8340(2)	42(1)
C(30)	5449(4)	3208(3)	8957(2)	46(1)
C(31)	4159(4)	3517(4)	9024(2)	48(1)
C(32)	3055(4)	3286(4)	8486(2)	50(1)
C(33)	3223(3)	2734(4)	7867(2)	48(1)
O(1W)	2092(3)	9725(4)	7366(3)	109(2)

Table 3. Bond lengths [Å] and angles [°] for d13160_sq_d_x.

O(1)-C(1)	1.225(4)
O(2)-C(3)	1.220(4)
O(3)-C(9)	1.223(4)
O(4)-C(12)	1.241(4)
O(5)-C(18)	1.237(4)
O(6)-C(25)	1.222(4)
N(1)-C(1)	1.324(4)
N(1)-C(26)	1.451(4)
N(1)-H(1N)	0.9000
N(2)-C(3)	1.336(4)
N(2)-C(2)	1.443(4)
N(2)-H(2N)	0.9000
N(3)-C(9)	1.346(4)
N(3)-C(8)	1.462(3)
N(3)-H(3N)	0.9000
N(4)-C(12)	1.334(4)
N(4)-C(10)	1.451(4)
N(4)-H(4N)	0.9000
N(5)-C(13)	1.476(4)
N(5)-C(17)	1.479(5)
N(5)-C(16)	1.483(4)
N(6)-C(18)	1.327(4)
N(6)-C(19)	1.491(3)
N(6)-H(6N)	0.9000
N(7)-C(25)	1.318(5)
N(7)-C(23)	1.470(5)
N(7)-H(7N)	0.9000
C(1)-C(2)	1.512(4)
C(2)-H(2A)	0.9900
C(2)-H(2B)	0.9900
C(3)-C(8)	1.540(4)
C(4)-C(8)	1.520(4)
C(4)-C(5)	1.533(4)
C(4)-H(4A)	0.9900

C(4)-H(4B)	0.9900
C(5)-C(7)	1.507(5)
C(5)-C(6)	1.534(5)
C(5)-H(5A)	1.0000
C(6)-H(6A)	0.9800
C(6)-H(6B)	0.9800
C(6)-H(6C)	0.9800
C(7)-H(7A)	0.9800
C(7)-H(7B)	0.9800
C(7)-H(7C)	0.9800
C(8)-H(8A)	1.0000
C(9)-C(10)	1.523(4)
C(10)-C(11)	1.517(5)
C(10)-H(10A)	1.0000
C(11)-H(11A)	0.9800
C(11)-H(11B)	0.9800
C(11)-H(11C)	0.9800
C(12)-C(13)	1.511(5)
C(13)-C(14)	1.554(4)
C(13)-H(13A)	1.0000
C(14)-C(15)	1.519(5)
C(14)-H(14A)	0.9900
C(14)-H(14B)	0.9900
C(15)-C(16)	1.499(6)
C(15)-H(15A)	0.9900
C(15)-H(15B)	0.9900
C(16)-H(16A)	0.9900
C(16)-H(16B)	0.9900
C(17)-C(18)	1.534(4)
C(17)-C(23)	1.536(5)
C(17)-H(17A)	1.0000
C(19)-C(22)	1.517(5)
C(19)-C(20)	1.519(5)
C(19)-C(21)	1.521(5)
C(20)-H(20A)	0.9800
C(20)-H(20B)	0.9800

C(20)-H(20C)	0.9800
C(21)-H(21A)	0.9800
C(21)-H(21B)	0.9800
C(21)-H(21C)	0.9800
C(22)-H(22A)	0.9800
C(22)-H(22B)	0.9800
C(22)-H(22C)	0.9800
C(23)-C(24)	1.515(6)
C(23)-H(23A)	1.0000
C(24)-H(24A)	0.9800
C(24)-H(24B)	0.9800
C(24)-H(24C)	0.9800
C(25)-C(26)	1.540(6)
C(26)-C(27)	1.545(5)
C(26)-H(26A)	1.0000
C(27)-C(28)	1.515(4)
C(27)-H(27A)	0.9900
C(27)-H(27B)	0.9900
C(28)-C(29)	1.375(5)
C(28)-C(33)	1.393(5)
C(29)-C(30)	1.397(5)
C(29)-H(29A)	0.9500
C(30)-C(31)	1.372(5)
C(30)-H(30A)	0.9500
C(31)-C(32)	1.370(5)
C(31)-H(31A)	0.9500
C(32)-C(33)	1.386(5)
C(32)-H(32A)	0.9500
C(33)-H(33A)	0.9500
O(1W)-H(1WA)	0.8421
O(1W)-H(1WB)	0.8405

C(1)-N(1)-C(26)	121.8(2)
C(1)-N(1)-H(1N)	107.2
C(26)-N(1)-H(1N)	124.3
C(3)-N(2)-C(2)	122.6(3)

C(3)-N(2)-H(2N)	119.9
C(2)-N(2)-H(2N)	117.3
C(9)-N(3)-C(8)	122.5(2)
C(9)-N(3)-H(3N)	121.4
C(8)-N(3)-H(3N)	115.8
C(12)-N(4)-C(10)	121.4(3)
C(12)-N(4)-H(4N)	121.5
C(10)-N(4)-H(4N)	117.1
C(13)-N(5)-C(17)	120.2(3)
C(13)-N(5)-C(16)	108.4(2)
C(17)-N(5)-C(16)	113.6(3)
C(18)-N(6)-C(19)	127.6(3)
C(18)-N(6)-H(6N)	108.7
C(19)-N(6)-H(6N)	116.2
C(25)-N(7)-C(23)	123.2(3)
C(25)-N(7)-H(7N)	120.5
C(23)-N(7)-H(7N)	115.3
O(1)-C(1)-N(1)	122.0(3)
O(1)-C(1)-C(2)	120.1(3)
N(1)-C(1)-C(2)	117.9(2)
N(2)-C(2)-C(1)	116.0(3)
N(2)-C(2)-H(2A)	108.3
C(1)-C(2)-H(2A)	108.3
N(2)-C(2)-H(2B)	108.3
C(1)-C(2)-H(2B)	108.3
H(2A)-C(2)-H(2B)	107.4
O(2)-C(3)-N(2)	122.4(3)
O(2)-C(3)-C(8)	120.8(3)
N(2)-C(3)-C(8)	116.8(2)
C(8)-C(4)-C(5)	114.9(3)
C(8)-C(4)-H(4A)	108.5
C(5)-C(4)-H(4A)	108.5
C(8)-C(4)-H(4B)	108.5
C(5)-C(4)-H(4B)	108.5
H(4A)-C(4)-H(4B)	107.5
C(7)-C(5)-C(4)	112.2(3)

C(7)-C(5)-C(6)	110.3(3)
C(4)-C(5)-C(6)	109.3(3)
C(7)-C(5)-H(5A)	108.3
C(4)-C(5)-H(5A)	108.3
C(6)-C(5)-H(5A)	108.3
C(5)-C(6)-H(6A)	109.5
C(5)-C(6)-H(6B)	109.5
H(6A)-C(6)-H(6B)	109.5
C(5)-C(6)-H(6C)	109.5
H(6A)-C(6)-H(6C)	109.5
H(6B)-C(6)-H(6C)	109.5
C(5)-C(7)-H(7A)	109.5
C(5)-C(7)-H(7B)	109.5
H(7A)-C(7)-H(7B)	109.5
C(5)-C(7)-H(7C)	109.5
H(7A)-C(7)-H(7C)	109.5
H(7B)-C(7)-H(7C)	109.5
N(3)-C(8)-C(4)	112.1(2)
N(3)-C(8)-C(3)	111.5(2)
C(4)-C(8)-C(3)	111.6(2)
N(3)-C(8)-H(8A)	107.1
C(4)-C(8)-H(8A)	107.1
C(3)-C(8)-H(8A)	107.1
O(3)-C(9)-N(3)	122.8(3)
O(3)-C(9)-C(10)	119.7(3)
N(3)-C(9)-C(10)	117.6(3)
N(4)-C(10)-C(11)	111.0(3)
N(4)-C(10)-C(9)	113.6(3)
C(11)-C(10)-C(9)	110.3(3)
N(4)-C(10)-H(10A)	107.2
C(11)-C(10)-H(10A)	107.2
C(9)-C(10)-H(10A)	107.2
C(10)-C(11)-H(11A)	109.5
C(10)-C(11)-H(11B)	109.5
H(11A)-C(11)-H(11B)	109.5
C(10)-C(11)-H(11C)	109.5

H(11A)-C(11)-H(11C)	109.5
H(11B)-C(11)-H(11C)	109.5
O(4)-C(12)-N(4)	122.4(3)
O(4)-C(12)-C(13)	120.5(3)
N(4)-C(12)-C(13)	117.1(3)
N(5)-C(13)-C(12)	112.6(2)
N(5)-C(13)-C(14)	105.0(2)
C(12)-C(13)-C(14)	110.6(3)
N(5)-C(13)-H(13A)	109.5
C(12)-C(13)-H(13A)	109.5
C(14)-C(13)-H(13A)	109.5
C(15)-C(14)-C(13)	102.5(3)
C(15)-C(14)-H(14A)	111.3
C(13)-C(14)-H(14A)	111.3
C(15)-C(14)-H(14B)	111.3
C(13)-C(14)-H(14B)	111.3
H(14A)-C(14)-H(14B)	109.2
C(16)-C(15)-C(14)	102.2(3)
C(16)-C(15)-H(15A)	111.3
C(14)-C(15)-H(15A)	111.3
C(16)-C(15)-H(15B)	111.3
C(14)-C(15)-H(15B)	111.3
H(15A)-C(15)-H(15B)	109.2
N(5)-C(16)-C(15)	103.7(3)
N(5)-C(16)-H(16A)	111.0
C(15)-C(16)-H(16A)	111.0
N(5)-C(16)-H(16B)	111.0
C(15)-C(16)-H(16B)	111.0
H(16A)-C(16)-H(16B)	109.0
N(5)-C(17)-C(18)	109.0(3)
N(5)-C(17)-C(23)	117.9(3)
C(18)-C(17)-C(23)	112.0(3)
N(5)-C(17)-H(17A)	105.7
C(18)-C(17)-H(17A)	105.7
C(23)-C(17)-H(17A)	105.7
O(5)-C(18)-N(6)	124.9(3)

O(5)-C(18)-C(17)	120.7(3)
N(6)-C(18)-C(17)	114.4(3)
N(6)-C(19)-C(22)	109.2(2)
N(6)-C(19)-C(20)	109.0(2)
C(22)-C(19)-C(20)	111.7(3)
N(6)-C(19)-C(21)	106.0(2)
C(22)-C(19)-C(21)	110.5(3)
C(20)-C(19)-C(21)	110.3(3)
C(19)-C(20)-H(20A)	109.5
C(19)-C(20)-H(20B)	109.5
H(20A)-C(20)-H(20B)	109.5
C(19)-C(20)-H(20C)	109.5
H(20A)-C(20)-H(20C)	109.5
H(20B)-C(20)-H(20C)	109.5
C(19)-C(21)-H(21A)	109.5
C(19)-C(21)-H(21B)	109.5
H(21A)-C(21)-H(21B)	109.5
C(19)-C(21)-H(21C)	109.5
H(21A)-C(21)-H(21C)	109.5
H(21B)-C(21)-H(21C)	109.5
C(19)-C(22)-H(22A)	109.5
C(19)-C(22)-H(22B)	109.5
H(22A)-C(22)-H(22B)	109.5
C(19)-C(22)-H(22C)	109.5
H(22A)-C(22)-H(22C)	109.5
H(22B)-C(22)-H(22C)	109.5
N(7)-C(23)-C(24)	110.7(4)
N(7)-C(23)-C(17)	112.0(3)
C(24)-C(23)-C(17)	110.4(3)
N(7)-C(23)-H(23A)	107.9
C(24)-C(23)-H(23A)	107.9
C(17)-C(23)-H(23A)	107.9
C(23)-C(24)-H(24A)	109.5
C(23)-C(24)-H(24B)	109.5
H(24A)-C(24)-H(24B)	109.5
C(23)-C(24)-H(24C)	109.5

H(24A)-C(24)-H(24C)	109.5
H(24B)-C(24)-H(24C)	109.5
O(6)-C(25)-N(7)	124.0(4)
O(6)-C(25)-C(26)	118.6(3)
N(7)-C(25)-C(26)	117.4(3)
N(1)-C(26)-C(25)	113.3(3)
N(1)-C(26)-C(27)	113.1(3)
C(25)-C(26)-C(27)	110.8(3)
N(1)-C(26)-H(26A)	106.3
C(25)-C(26)-H(26A)	106.3
C(27)-C(26)-H(26A)	106.3
C(28)-C(27)-C(26)	114.3(3)
C(28)-C(27)-H(27A)	108.7
C(26)-C(27)-H(27A)	108.7
C(28)-C(27)-H(27B)	108.7
C(26)-C(27)-H(27B)	108.7
H(27A)-C(27)-H(27B)	107.6
C(29)-C(28)-C(33)	119.3(3)
C(29)-C(28)-C(27)	121.7(3)
C(33)-C(28)-C(27)	118.9(3)
C(28)-C(29)-C(30)	120.7(3)
C(28)-C(29)-H(29A)	119.7
C(30)-C(29)-H(29A)	119.7
C(31)-C(30)-C(29)	119.4(3)
C(31)-C(30)-H(30A)	120.3
C(29)-C(30)-H(30A)	120.3
C(32)-C(31)-C(30)	120.4(3)
C(32)-C(31)-H(31A)	119.8
C(30)-C(31)-H(31A)	119.8
C(31)-C(32)-C(33)	120.6(3)
C(31)-C(32)-H(32A)	119.7
C(33)-C(32)-H(32A)	119.7
C(32)-C(33)-C(28)	119.6(3)
C(32)-C(33)-H(33A)	120.2
C(28)-C(33)-H(33A)	120.2
H(1WA)-O(1W)-H(1WB)	122.8

Symmetry transformations used to generate equivalent atoms:

Table 4. Anisotropic displacement parameters ($Å^2$x 10^3) for d13160_sq_d_x. The anisotropic displacement factor exponent takes the form: $-2\pi^2$[h^2 $a^{*2}U^{11}$ + ... + 2 h k a* b* U^{12}]

	U^{11}	U^{22}	U^{33}	U^{23}	U^{13}	U^{12}
O(1)	46(1)	160(4)	74(2)	-74(2)	13(1)	-10(2)
O(2)	88(2)	30(1)	41(1)	1(1)	4(1)	4(1)
O(3)	48(1)	39(1)	68(2)	12(1)	5(1)	-11(1)
O(4)	62(1)	41(1)	33(1)	2(1)	15(1)	2(1)
O(5)	42(1)	39(1)	28(1)	-2(1)	3(1)	7(1)
O(6)	51(2)	105(2)	65(2)	-46(2)	3(1)	-14(2)
N(1)	47(1)	45(2)	34(1)	-13(1)	16(1)	-17(1)
N(2)	45(1)	27(1)	36(1)	-1(1)	12(1)	-1(1)
N(3)	38(1)	25(1)	31(1)	1(1)	7(1)	0(1)
N(4)	56(2)	32(1)	30(1)	1(1)	4(1)	1(1)
N(5)	60(2)	44(2)	23(1)	0(1)	-1(1)	11(1)
N(6)	51(2)	45(2)	30(1)	-1(1)	3(1)	14(1)
N(7)	46(2)	54(2)	25(1)	-2(1)	2(1)	-10(1)
C(1)	45(2)	41(2)	31(1)	-6(1)	8(1)	1(1)
C(2)	45(2)	38(2)	40(2)	-5(1)	15(1)	0(1)
C(3)	33(1)	27(2)	39(2)	-1(1)	6(1)	2(1)
C(4)	41(2)	31(2)	30(1)	2(1)	3(1)	-2(1)
C(5)	45(2)	38(2)	34(2)	-3(1)	0(1)	3(1)
C(6)	65(2)	63(2)	34(2)	-3(2)	9(2)	2(2)
C(7)	45(2)	80(3)	56(2)	-19(2)	-8(2)	-4(2)
C(8)	33(1)	28(1)	33(1)	2(1)	0(1)	1(1)
C(9)	51(2)	29(2)	34(1)	-5(1)	7(1)	-6(1)
C(10)	58(2)	30(2)	32(1)	2(1)	9(1)	-5(1)
C(11)	63(2)	29(2)	50(2)	-4(1)	6(2)	-4(2)
C(12)	62(2)	32(2)	24(1)	5(1)	10(1)	-1(2)
C(13)	62(2)	30(2)	24(1)	0(1)	5(1)	1(1)
C(14)	75(2)	40(2)	23(1)	0(1)	3(1)	-3(2)
C(15)	88(3)	41(2)	27(2)	4(1)	-6(2)	-4(2)
C(16)	80(2)	45(2)	28(2)	-2(1)	-8(2)	17(2)
C(17)	47(2)	57(2)	32(2)	-3(2)	-2(1)	10(2)
C(18)	44(2)	43(2)	24(1)	-3(1)	2(1)	2(1)

C(19)	49(2)	34(2)	24(1)	0(1)	3(1)	9(1)
C(20)	52(2)	42(2)	31(1)	-5(1)	9(1)	2(1)
C(21)	56(2)	50(2)	34(2)	-4(1)	9(1)	8(2)
C(22)	61(2)	40(2)	40(2)	1(1)	11(2)	-2(2)
C(23)	48(2)	73(2)	30(2)	-9(2)	0(1)	1(2)
C(24)	54(2)	109(4)	57(2)	-19(2)	15(2)	-11(2)
C(25)	47(2)	69(2)	38(2)	-18(2)	19(1)	-20(2)
C(26)	49(2)	57(2)	42(2)	-19(2)	18(1)	-24(2)
C(27)	62(2)	49(2)	49(2)	-20(2)	28(2)	-23(2)
C(28)	51(2)	38(2)	40(2)	-4(1)	21(1)	-10(1)
C(29)	44(2)	36(2)	48(2)	4(1)	17(1)	-1(1)
C(30)	56(2)	43(2)	37(2)	4(1)	8(1)	-1(2)
C(31)	73(2)	43(2)	33(2)	6(1)	21(2)	6(2)
C(32)	51(2)	57(2)	50(2)	11(2)	25(2)	8(2)
C(33)	47(2)	59(2)	42(2)	3(2)	17(1)	-7(2)
O(1W)	50(2)	99(3)	176(4)	73(3)	24(2)	4(2)

Table 5. Hydrogen coordinates (x 10^4) and isotropic displacement parameters ($Å^2$x 10^3) for d13160_sq_d_x.

	x	y	z	U(eq)
H(1N)	6858	3446	7289	61
H(2N)	8679	5483	7125	53
H(3N)	7894	6921	7928	48
H(4N)	6772	9035	7092	60
H(6N)	3499	8416	7806	65
H(7N)	4960	5137	7066	64
H(2A)	9437	2849	7042	48
H(2B)	9206	3900	6418	48
H(4A)	9164	4616	9292	42
H(4B)	7900	5579	8996	42
H(5A)	9247	7458	9500	49
H(6A)	9103	6975	10661	81
H(6B)	8917	5420	10471	81
H(6C)	7764	6468	10118	81
H(7A)	11297	7091	10322	95
H(7B)	11433	6589	9572	95
H(7C)	11218	5528	10143	95
H(8A)	10414	5875	8591	39
H(10A)	9397	9226	7066	48
H(11A)	8275	10942	7531	73
H(11B)	9667	10528	8060	73
H(11C)	8239	10021	8189	73
H(13A)	5717	6344	6177	48
H(14A)	6619	7711	5217	57
H(14B)	5133	7020	5047	57
H(15A)	5748	9752	5476	66
H(15B)	4585	9258	4819	66
H(16A)	3811	9771	5913	66
H(16B)	3264	8392	5525	66
H(17A)	3262	8154	6793	57

H(20A)	6367	9696	8737	63
H(20B)	6902	8186	8759	63
H(20C)	6632	8867	9448	63
H(21A)	2980	8980	8869	71
H(21B)	3975	10174	8769	71
H(21C)	4203	9417	9500	71
H(22A)	3769	6651	8930	70
H(22B)	5019	6985	9569	70
H(22C)	5294	6300	8882	70
H(23A)	2963	6225	6030	62
H(24A)	1670	5092	6709	110
H(24B)	1439	6675	6723	110
H(24C)	2408	5946	7374	110
H(26A)	5081	2235	6172	58
H(27A)	3818	1325	6896	61
H(27B)	5393	1058	7234	61
H(29A)	6515	2400	8294	50
H(30A)	6215	3386	9327	55
H(31A)	4031	3893	9446	58
H(32A)	2167	3506	8537	61
H(33A)	2454	2587	7494	58
H(1WA)	1702	9152	7566	163
H(1WB)	1670	10200	7035	163

Table 6. Hydrogen bonds for d13160_sq_d_x [Å and °].

D-H...A	d(D-H)	d(H...A)	d(D...A)	<(DHA)
N(1)-H(1N)...N(2)	0.90	2.39	2.788(3)	107.1
N(2)-H(2N)...O(4)	0.90	2.01	2.870(3)	158.6
N(2)-H(2N)...N(3)	0.90	2.32	2.723(3)	106.9
N(3)-H(3N)...O(5)	0.90	2.05	2.945(3)	170.4
N(3)-H(3N)...N(4)	0.90	2.45	2.785(3)	102.2
N(4)-H(4N)...N(5)	0.90	2.30	2.694(4)	106.3
N(6)-H(6N)...O(1W)	0.90	1.98	2.782(4)	147.9
N(7)-H(7N)...O(5)	0.90	2.06	2.790(3)	137.3
O(1W)-H(1WA)...O(3)#1	0.84	1.92	2.760(4)	178.0

Symmetry transformations used to generate equivalent atoms:

#1 x-1,y,z

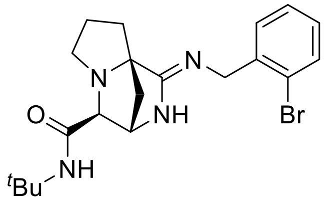

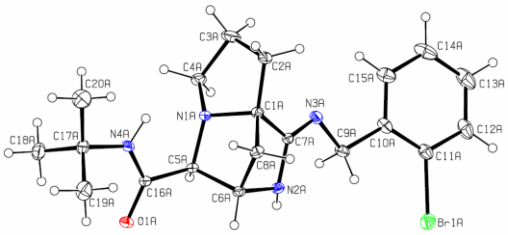

(28)

Table 1. Crystal data and structure refinement for d14137.

Identification code	d14137	
Empirical formula	C20 H27 Br N4 O	
Formula weight	419.36	
Temperature	147(2) K	
Wavelength	1.54178 Å	
Crystal system	Monoclinic	
Space group	$P2_1$	
Unit cell dimensions	a = 8.9698(4) Å	α= 90°.
	b = 18.4284(8) Å	β= 98.310(2)°.
	c = 12.3981(5) Å	γ = 90°.
Volume	2027.88(15) $Å^3$	
Z	4	
Density (calculated)	1.374 Mg/m^3	
Absorption coefficient	2.882 mm^{-1}	
F(000)	872	
Crystal size	0.220 x 0.200 x 0.180 mm^3	
Theta range for data collection	3.603 to 67.191°.	
Index ranges	-10<=h<=10, -21<=k<=21, -14<=l<=14	
Reflections collected	39585	
Independent reflections	7137 [R(int) = 0.0326]	
Completeness to theta = 67.679°	98.0 %	
Absorption correction	Semi-empirical from equivalents	
Max. and min. transmission	0.7529 and 0.6391	
Refinement method	Full-matrix least-squares on F^2	
Data / restraints / parameters	7137 / 1 / 491	
Goodness-of-fit on F^2	0.977	
Final R indices [I>2sigma(I)]	R1 = 0.0226, wR2 = 0.0588	
R indices (all data)	R1 = 0.0228, wR2 = 0.0590	

Absolute structure parameter	0.016(3)
Extinction coefficient	n/a
Largest diff. peak and hole	0.495 and -0.868 e.Å^{-3}

Table 2. Atomic coordinates (x 10^4) and equivalent isotropic displacement parameters ($Å^2$x 10^3) for d14137. U(eq) is defined as one third of the trace of the orthogonalized U^{ij} tensor.

	x	y	z	U(eq)
Br(1A)	7210(1)	2823(1)	11118(1)	32(1)
O(1A)	110(2)	-517(1)	12149(2)	26(1)
N(1A)	641(2)	300(1)	9564(2)	19(1)
N(2A)	2435(3)	1302(1)	10923(2)	19(1)
N(3A)	3372(3)	1514(1)	9224(2)	22(1)
N(4A)	-1407(3)	-421(1)	10510(2)	24(1)
C(1A)	746(3)	1093(1)	9357(2)	18(1)
C(2A)	307(3)	1137(2)	8140(2)	25(1)
C(3A)	911(5)	421(2)	7734(2)	42(1)
C(4A)	1401(4)	-57(2)	8750(2)	28(1)
C(5A)	997(3)	183(1)	10742(2)	18(1)
C(6A)	1023(3)	984(1)	11150(2)	19(1)
C(7A)	2358(3)	1332(1)	9799(2)	18(1)
C(8A)	-90(3)	1367(2)	10267(2)	22(1)
C(9A)	4793(3)	1743(2)	9850(2)	26(1)
C(10A)	5751(3)	2154(2)	9149(2)	25(1)
C(11A)	6845(3)	2654(2)	9587(2)	28(1)
C(12A)	7710(3)	3044(2)	8948(3)	39(1)
C(13A)	7495(4)	2940(2)	7839(3)	50(1)
C(14A)	6423(4)	2446(2)	7380(3)	47(1)
C(15A)	5569(4)	2062(2)	8032(3)	34(1)
C(16A)	-154(3)	-292(2)	11198(2)	20(1)
C(17A)	-2719(3)	-870(2)	10695(2)	28(1)
C(18A)	-2203(4)	-1654(2)	10882(3)	38(1)
C(19A)	-3386(4)	-588(2)	11678(3)	40(1)
C(20A)	-3855(4)	-805(3)	9670(3)	54(1)
Br(1B)	-2337(1)	-2261(1)	3727(1)	27(1)
O(1B)	4938(2)	1038(1)	2602(1)	24(1)
N(1B)	4752(2)	-116(1)	4966(2)	17(1)
N(2B)	1618(2)	-5(1)	4250(2)	17(1)
N(3B)	1652(2)	-982(1)	5552(2)	19(1)

N(4B)	6486(2)	893(1)	4213(2)	20(1)
C(1B)	3581(3)	-7(1)	5683(2)	16(1)
C(2B)	4347(3)	-306(2)	6761(2)	22(1)
C(3B)	5340(3)	-930(2)	6399(2)	29(1)
C(4B)	5298(3)	-856(2)	5155(2)	25(1)
C(5B)	4189(3)	195(1)	3896(2)	17(1)
C(6B)	2720(3)	569(1)	4156(2)	16(1)
C(7B)	2156(3)	-407(1)	5170(2)	17(1)
C(8B)	3147(3)	774(1)	5372(2)	17(1)
C(9B)	302(3)	-1300(2)	4935(2)	22(1)
C(10B)	-613(3)	-1706(1)	5674(2)	18(1)
C(11B)	-1821(3)	-2146(2)	5261(2)	18(1)
C(12B)	-2691(3)	-2508(2)	5927(2)	23(1)
C(13B)	-2346(3)	-2442(2)	7045(2)	25(1)
C(14B)	-1140(3)	-2010(2)	7483(2)	24(1)
C(15B)	-291(3)	-1647(2)	6803(2)	21(1)
C(16B)	5264(3)	751(2)	3506(2)	18(1)
C(17B)	7655(3)	1442(2)	4107(2)	21(1)
C(18B)	8442(3)	1262(2)	3129(2)	25(1)
C(19B)	6930(3)	2195(2)	3987(2)	25(1)
C(20B)	8775(3)	1397(2)	5150(3)	30(1)

Table 3. Bond lengths [Å] and angles [°] for d14137.

Br(1A)-C(11A)	1.904(3)
O(1A)-C(16A)	1.240(3)
N(1A)-C(4A)	1.454(3)
N(1A)-C(5A)	1.465(3)
N(1A)-C(1A)	1.490(3)
N(2A)-C(7A)	1.386(3)
N(2A)-C(6A)	1.460(3)
N(2A)-H(2NA)	0.76(4)
N(3A)-C(7A)	1.279(4)
N(3A)-C(9A)	1.456(4)
N(4A)-C(16A)	1.331(4)
N(4A)-C(17A)	1.483(4)
N(4A)-H(4NA)	0.89(4)
C(1A)-C(2A)	1.505(4)
C(1A)-C(8A)	1.529(4)
C(1A)-C(7A)	1.535(4)
C(2A)-C(3A)	1.539(4)
C(2A)-H(2AA)	0.9900
C(2A)-H(2AB)	0.9900
C(3A)-C(4A)	1.548(4)
C(3A)-H(3AA)	0.9900
C(3A)-H(3AB)	0.9900
C(4A)-H(4AA)	0.9900
C(4A)-H(4AB)	0.9900
C(5A)-C(16A)	1.524(4)
C(5A)-C(6A)	1.558(3)
C(5A)-H(5AA)	1.0000
C(6A)-C(8A)	1.542(4)
C(6A)-H(6AA)	1.0000
C(8A)-H(8AA)	0.9900
C(8A)-H(8AB)	0.9900
C(9A)-C(10A)	1.512(4)
C(9A)-H(9AA)	0.9900
C(9A)-H(9AB)	0.9900

C(10A)-C(15A)	1.381(4)
C(10A)-C(11A)	1.397(4)
C(11A)-C(12A)	1.387(4)
C(12A)-C(13A)	1.374(5)
C(12A)-H(12A)	0.9500
C(13A)-C(14A)	1.386(6)
C(13A)-H(13A)	0.9500
C(14A)-C(15A)	1.385(5)
C(14A)-H(14A)	0.9500
C(15A)-H(15A)	0.9500
C(17A)-C(20A)	1.514(5)
C(17A)-C(18A)	1.524(5)
C(17A)-C(19A)	1.525(4)
C(18A)-H(18A)	0.9800
C(18A)-H(18B)	0.9800
C(18A)-H(18C)	0.9800
C(19A)-H(19A)	0.9800
C(19A)-H(19B)	0.9800
C(19A)-H(19C)	0.9800
C(20A)-H(20A)	0.9800
C(20A)-H(20B)	0.9800
C(20A)-H(20C)	0.9800
Br(1B)-C(11B)	1.904(2)
O(1B)-C(16B)	1.235(3)
N(1B)-C(4B)	1.457(3)
N(1B)-C(5B)	1.466(3)
N(1B)-C(1B)	1.485(3)
N(2B)-C(7B)	1.386(3)
N(2B)-C(6B)	1.464(3)
N(2B)-H(2NB)	0.85(4)
N(3B)-C(7B)	1.271(4)
N(3B)-C(9B)	1.458(3)
N(4B)-C(16B)	1.327(4)
N(4B)-C(17B)	1.477(4)
N(4B)-H(4NB)	0.70(4)
C(1B)-C(2B)	1.515(4)

C(1B)-C(8B)	1.526(3)
C(1B)-C(7B)	1.532(3)
C(2B)-C(3B)	1.560(4)
C(2B)-H(2BA)	0.9900
C(2B)-H(2BB)	0.9900
C(3B)-C(4B)	1.543(4)
C(3B)-H(3BA)	0.9900
C(3B)-H(3BB)	0.9900
C(4B)-H(4BA)	0.9900
C(4B)-H(4BB)	0.9900
C(5B)-C(16B)	1.533(4)
C(5B)-C(6B)	1.561(3)
C(5B)-H(5BA)	1.0000
C(6B)-C(8B)	1.548(3)
C(6B)-H(6BA)	1.0000
C(8B)-H(8BA)	0.9900
C(8B)-H(8BB)	0.9900
C(9B)-C(10B)	1.514(4)
C(9B)-H(9BA)	0.9900
C(9B)-H(9BB)	0.9900
C(10B)-C(11B)	1.391(4)
C(10B)-C(15B)	1.392(4)
C(11B)-C(12B)	1.386(4)
C(12B)-C(13B)	1.381(4)
C(12B)-H(12B)	0.9500
C(13B)-C(14B)	1.388(4)
C(13B)-H(13B)	0.9500
C(14B)-C(15B)	1.386(4)
C(14B)-H(14B)	0.9500
C(15B)-H(15B)	0.9500
C(17B)-C(20B)	1.521(4)
C(17B)-C(18B)	1.524(4)
C(17B)-C(19B)	1.531(4)
C(18B)-H(18D)	0.9800
C(18B)-H(18E)	0.9800
C(18B)-H(18F)	0.9800

C(19B)-H(19D)	0.9800
C(19B)-H(19E)	0.9800
C(19B)-H(19F)	0.9800
C(20B)-H(20D)	0.9800
C(20B)-H(20E)	0.9800
C(20B)-H(20F)	0.9800

C(4A)-N(1A)-C(5A)	124.7(2)
C(4A)-N(1A)-C(1A)	106.02(19)
C(5A)-N(1A)-C(1A)	107.83(19)
C(7A)-N(2A)-C(6A)	106.9(2)
C(7A)-N(2A)-H(2NA)	121(2)
C(6A)-N(2A)-H(2NA)	118(2)
C(7A)-N(3A)-C(9A)	114.6(2)
C(16A)-N(4A)-C(17A)	128.0(2)
C(16A)-N(4A)-H(4NA)	115(2)
C(17A)-N(4A)-H(4NA)	117(2)
N(1A)-C(1A)-C(2A)	102.2(2)
N(1A)-C(1A)-C(8A)	98.4(2)
C(2A)-C(1A)-C(8A)	129.6(2)
N(1A)-C(1A)-C(7A)	107.4(2)
C(2A)-C(1A)-C(7A)	116.0(2)
C(8A)-C(1A)-C(7A)	100.3(2)
C(1A)-C(2A)-C(3A)	103.2(2)
C(1A)-C(2A)-H(2AA)	111.1
C(3A)-C(2A)-H(2AA)	111.1
C(1A)-C(2A)-H(2AB)	111.1
C(3A)-C(2A)-H(2AB)	111.1
H(2AA)-C(2A)-H(2AB)	109.1
C(2A)-C(3A)-C(4A)	107.1(2)
C(2A)-C(3A)-H(3AA)	110.3
C(4A)-C(3A)-H(3AA)	110.3
C(2A)-C(3A)-H(3AB)	110.3
C(4A)-C(3A)-H(3AB)	110.3
H(3AA)-C(3A)-H(3AB)	108.6
N(1A)-C(4A)-C(3A)	101.6(2)

N(1A)-C(4A)-H(4AA)	111.4
C(3A)-C(4A)-H(4AA)	111.4
N(1A)-C(4A)-H(4AB)	111.4
C(3A)-C(4A)-H(4AB)	111.4
H(4AA)-C(4A)-H(4AB)	109.3
N(1A)-C(5A)-C(16A)	113.1(2)
N(1A)-C(5A)-C(6A)	100.10(19)
C(16A)-C(5A)-C(6A)	113.8(2)
N(1A)-C(5A)-H(5AA)	109.8
C(16A)-C(5A)-H(5AA)	109.8
C(6A)-C(5A)-H(5AA)	109.8
N(2A)-C(6A)-C(8A)	99.7(2)
N(2A)-C(6A)-C(5A)	106.90(19)
C(8A)-C(6A)-C(5A)	103.06(19)
N(2A)-C(6A)-H(6AA)	115.1
C(8A)-C(6A)-H(6AA)	115.1
C(5A)-C(6A)-H(6AA)	115.1
N(3A)-C(7A)-N(2A)	129.4(2)
N(3A)-C(7A)-C(1A)	125.8(2)
N(2A)-C(7A)-C(1A)	104.8(2)
C(1A)-C(8A)-C(6A)	92.08(19)
C(1A)-C(8A)-H(8AA)	113.3
C(6A)-C(8A)-H(8AA)	113.3
C(1A)-C(8A)-H(8AB)	113.3
C(6A)-C(8A)-H(8AB)	113.3
H(8AA)-C(8A)-H(8AB)	110.6
N(3A)-C(9A)-C(10A)	111.4(2)
N(3A)-C(9A)-H(9AA)	109.3
C(10A)-C(9A)-H(9AA)	109.3
N(3A)-C(9A)-H(9AB)	109.3
C(10A)-C(9A)-H(9AB)	109.3
H(9AA)-C(9A)-H(9AB)	108.0
C(15A)-C(10A)-C(11A)	116.5(3)
C(15A)-C(10A)-C(9A)	121.4(3)
C(11A)-C(10A)-C(9A)	122.2(3)
C(12A)-C(11A)-C(10A)	122.6(3)

C(12A)-C(11A)-Br(1A)	117.0(2)
C(10A)-C(11A)-Br(1A)	120.3(2)
C(13A)-C(12A)-C(11A)	119.3(3)
C(13A)-C(12A)-H(12A)	120.3
C(11A)-C(12A)-H(12A)	120.3
C(12A)-C(13A)-C(14A)	119.5(3)
C(12A)-C(13A)-H(13A)	120.3
C(14A)-C(13A)-H(13A)	120.3
C(15A)-C(14A)-C(13A)	120.3(3)
C(15A)-C(14A)-H(14A)	119.8
C(13A)-C(14A)-H(14A)	119.8
C(10A)-C(15A)-C(14A)	121.8(3)
C(10A)-C(15A)-H(15A)	119.1
C(14A)-C(15A)-H(15A)	119.1
O(1A)-C(16A)-N(4A)	125.3(3)
O(1A)-C(16A)-C(5A)	119.9(2)
N(4A)-C(16A)-C(5A)	114.8(2)
N(4A)-C(17A)-C(20A)	106.2(3)
N(4A)-C(17A)-C(18A)	108.8(3)
C(20A)-C(17A)-C(18A)	110.9(3)
N(4A)-C(17A)-C(19A)	110.2(3)
C(20A)-C(17A)-C(19A)	110.5(3)
C(18A)-C(17A)-C(19A)	110.3(3)
C(17A)-C(18A)-H(18A)	109.5
C(17A)-C(18A)-H(18B)	109.5
H(18A)-C(18A)-H(18B)	109.5
C(17A)-C(18A)-H(18C)	109.5
H(18A)-C(18A)-H(18C)	109.5
H(18B)-C(18A)-H(18C)	109.5
C(17A)-C(19A)-H(19A)	109.5
C(17A)-C(19A)-H(19B)	109.5
H(19A)-C(19A)-H(19B)	109.5
C(17A)-C(19A)-H(19C)	109.5
H(19A)-C(19A)-H(19C)	109.5
H(19B)-C(19A)-H(19C)	109.5
C(17A)-C(20A)-H(20A)	109.5

C(17A)-C(20A)-H(20B)	109.5
H(20A)-C(20A)-H(20B)	109.5
C(17A)-C(20A)-H(20C)	109.5
H(20A)-C(20A)-H(20C)	109.5
H(20B)-C(20A)-H(20C)	109.5
C(4B)-N(1B)-C(5B)	125.1(2)
C(4B)-N(1B)-C(1B)	106.31(19)
C(5B)-N(1B)-C(1B)	107.98(19)
C(7B)-N(2B)-C(6B)	107.0(2)
C(7B)-N(2B)-H(2NB)	123(2)
C(6B)-N(2B)-H(2NB)	120(2)
C(7B)-N(3B)-C(9B)	116.9(2)
C(16B)-N(4B)-C(17B)	127.3(3)
C(16B)-N(4B)-H(4NB)	119(3)
C(17B)-N(4B)-H(4NB)	114(3)
N(1B)-C(1B)-C(2B)	102.1(2)
N(1B)-C(1B)-C(8B)	98.78(19)
C(2B)-C(1B)-C(8B)	129.3(2)
N(1B)-C(1B)-C(7B)	107.8(2)
C(2B)-C(1B)-C(7B)	115.9(2)
C(8B)-C(1B)-C(7B)	100.45(19)
C(1B)-C(2B)-C(3B)	102.7(2)
C(1B)-C(2B)-H(2BA)	111.2
C(3B)-C(2B)-H(2BA)	111.2
C(1B)-C(2B)-H(2BB)	111.2
C(3B)-C(2B)-H(2BB)	111.2
H(2BA)-C(2B)-H(2BB)	109.1
C(4B)-C(3B)-C(2B)	106.9(2)
C(4B)-C(3B)-H(3BA)	110.4
C(2B)-C(3B)-H(3BA)	110.4
C(4B)-C(3B)-H(3BB)	110.4
C(2B)-C(3B)-H(3BB)	110.4
H(3BA)-C(3B)-H(3BB)	108.6
N(1B)-C(4B)-C(3B)	101.7(2)
N(1B)-C(4B)-H(4BA)	111.4
C(3B)-C(4B)-H(4BA)	111.4

N(1B)-C(4B)-H(4BB)	111.4
C(3B)-C(4B)-H(4BB)	111.4
H(4BA)-C(4B)-H(4BB)	109.3
N(1B)-C(5B)-C(16B)	113.3(2)
N(1B)-C(5B)-C(6B)	100.13(18)
C(16B)-C(5B)-C(6B)	111.0(2)
N(1B)-C(5B)-H(5BA)	110.7
C(16B)-C(5B)-H(5BA)	110.7
C(6B)-C(5B)-H(5BA)	110.7
N(2B)-C(6B)-C(8B)	100.02(18)
N(2B)-C(6B)-C(5B)	107.24(19)
C(8B)-C(6B)-C(5B)	102.23(19)
N(2B)-C(6B)-H(6BA)	115.2
C(8B)-C(6B)-H(6BA)	115.2
C(5B)-C(6B)-H(6BA)	115.2
N(3B)-C(7B)-N(2B)	130.7(2)
N(3B)-C(7B)-C(1B)	124.5(2)
N(2B)-C(7B)-C(1B)	104.8(2)
C(1B)-C(8B)-C(6B)	91.96(18)
C(1B)-C(8B)-H(8BA)	113.3
C(6B)-C(8B)-H(8BA)	113.3
C(1B)-C(8B)-H(8BB)	113.3
C(6B)-C(8B)-H(8BB)	113.3
H(8BA)-C(8B)-H(8BB)	110.6
N(3B)-C(9B)-C(10B)	111.2(2)
N(3B)-C(9B)-H(9BA)	109.4
C(10B)-C(9B)-H(9BA)	109.4
N(3B)-C(9B)-H(9BB)	109.4
C(10B)-C(9B)-H(9BB)	109.4
H(9BA)-C(9B)-H(9BB)	108.0
C(11B)-C(10B)-C(15B)	116.9(2)
C(11B)-C(10B)-C(9B)	121.8(2)
C(15B)-C(10B)-C(9B)	121.3(2)
C(12B)-C(11B)-C(10B)	122.5(2)
C(12B)-C(11B)-Br(1B)	117.7(2)
C(10B)-C(11B)-Br(1B)	119.80(19)

C(13B)-C(12B)-C(11B)	119.5(3)
C(13B)-C(12B)-H(12B)	120.3
C(11B)-C(12B)-H(12B)	120.3
C(12B)-C(13B)-C(14B)	119.4(2)
C(12B)-C(13B)-H(13B)	120.3
C(14B)-C(13B)-H(13B)	120.3
C(15B)-C(14B)-C(13B)	120.3(2)
C(15B)-C(14B)-H(14B)	119.8
C(13B)-C(14B)-H(14B)	119.8
C(14B)-C(15B)-C(10B)	121.4(3)
C(14B)-C(15B)-H(15B)	119.3
C(10B)-C(15B)-H(15B)	119.3
O(1B)-C(16B)-N(4B)	125.4(2)
O(1B)-C(16B)-C(5B)	119.7(2)
N(4B)-C(16B)-C(5B)	114.9(2)
N(4B)-C(17B)-C(20B)	106.1(2)
N(4B)-C(17B)-C(18B)	109.8(2)
C(20B)-C(17B)-C(18B)	109.8(2)
N(4B)-C(17B)-C(19B)	109.4(2)
C(20B)-C(17B)-C(19B)	110.6(2)
C(18B)-C(17B)-C(19B)	111.0(2)
C(17B)-C(18B)-H(18D)	109.5
C(17B)-C(18B)-H(18E)	109.5
H(18D)-C(18B)-H(18E)	109.5
C(17B)-C(18B)-H(18F)	109.5
H(18D)-C(18B)-H(18F)	109.5
H(18E)-C(18B)-H(18F)	109.5
C(17B)-C(19B)-H(19D)	109.5
C(17B)-C(19B)-H(19E)	109.5
H(19D)-C(19B)-H(19E)	109.5
C(17B)-C(19B)-H(19F)	109.5
H(19D)-C(19B)-H(19F)	109.5
H(19E)-C(19B)-H(19F)	109.5
C(17B)-C(20B)-H(20D)	109.5
C(17B)-C(20B)-H(20E)	109.5
H(20D)-C(20B)-H(20E)	109.5

C(17B)-C(20B)-H(20F)	109.5
H(20D)-C(20B)-H(20F)	109.5
H(20E)-C(20B)-H(20F)	109.5

Symmetry transformations used to generate equivalent atoms:

Table 4. Anisotropic displacement parameters ($Å^2$x 10^3) for d14137. The anisotropic displacement factor exponent takes the form: $-2\pi^2$[h^2 $a^{*2}U^{11}$ + ... + 2 h k a* b* U^{12}]

	U^{11}	U^{22}	U^{33}	U^{23}	U^{13}	U^{12}
Br(1A)	28(1)	31(1)	34(1)	1(1)	-4(1)	-4(1)
O(1A)	32(1)	30(1)	15(1)	3(1)	-1(1)	-6(1)
N(1A)	25(1)	18(1)	12(1)	-1(1)	2(1)	-2(1)
N(2A)	17(1)	23(1)	16(1)	-2(1)	-1(1)	-2(1)
N(3A)	22(1)	24(1)	20(1)	3(1)	3(1)	-3(1)
N(4A)	25(1)	30(1)	15(1)	4(1)	-2(1)	-10(1)
C(1A)	18(1)	19(1)	16(1)	1(1)	-1(1)	1(1)
C(2A)	26(1)	28(2)	19(1)	4(1)	-4(1)	-2(1)
C(3A)	68(2)	42(2)	15(1)	0(1)	8(1)	7(2)
C(4A)	41(2)	24(1)	18(1)	-4(1)	7(1)	3(1)
C(5A)	21(1)	21(1)	12(1)	-1(1)	0(1)	-2(1)
C(6A)	21(1)	22(1)	15(1)	-4(1)	4(1)	-3(1)
C(7A)	22(1)	16(1)	15(1)	-1(1)	2(1)	0(1)
C(8A)	21(1)	23(1)	22(1)	-3(1)	2(1)	3(1)
C(9A)	23(1)	35(2)	18(1)	6(1)	-1(1)	-5(1)
C(10A)	18(1)	31(2)	27(1)	9(1)	5(1)	3(1)
C(11A)	22(1)	31(2)	29(1)	10(1)	1(1)	3(1)
C(12A)	26(2)	43(2)	49(2)	18(2)	8(1)	-6(1)
C(13A)	33(2)	69(3)	51(2)	26(2)	16(2)	-4(2)
C(14A)	37(2)	78(3)	28(2)	15(2)	12(1)	6(2)
C(15A)	25(1)	51(2)	27(2)	5(1)	5(1)	2(1)
C(16A)	25(1)	19(1)	14(1)	-1(1)	1(1)	-1(1)
C(17A)	26(2)	31(2)	27(2)	-1(1)	4(1)	-13(1)
C(18A)	49(2)	30(2)	37(2)	-6(1)	16(2)	-13(2)
C(19A)	34(2)	43(2)	48(2)	-5(2)	18(2)	-8(2)
C(20A)	36(2)	73(3)	47(2)	10(2)	-13(2)	-28(2)
Br(1B)	29(1)	28(1)	22(1)	-3(1)	-3(1)	-5(1)
O(1B)	22(1)	34(1)	15(1)	7(1)	0(1)	-3(1)
N(1B)	16(1)	16(1)	19(1)	3(1)	2(1)	3(1)
N(2B)	15(1)	20(1)	16(1)	2(1)	-2(1)	-4(1)
N(3B)	18(1)	20(1)	19(1)	3(1)	0(1)	-2(1)

N(4B)	19(1)	26(1)	15(1)	7(1)	1(1)	-1(1)
C(1B)	16(1)	18(1)	15(1)	2(1)	0(1)	-1(1)
C(2B)	23(1)	24(1)	19(1)	5(1)	-3(1)	1(1)
C(3B)	27(1)	28(2)	32(2)	11(1)	1(1)	7(1)
C(4B)	25(1)	20(1)	32(2)	6(1)	5(1)	6(1)
C(5B)	15(1)	19(1)	16(1)	0(1)	0(1)	0(1)
C(6B)	16(1)	16(1)	16(1)	2(1)	1(1)	0(1)
C(7B)	14(1)	19(1)	16(1)	0(1)	1(1)	2(1)
C(8B)	18(1)	16(1)	17(1)	0(1)	1(1)	-1(1)
C(9B)	23(1)	24(1)	18(1)	3(1)	-1(1)	-8(1)
C(10B)	20(1)	14(1)	20(1)	2(1)	5(1)	2(1)
C(11B)	20(1)	14(1)	20(1)	2(1)	2(1)	2(1)
C(12B)	22(1)	16(1)	33(2)	1(1)	6(1)	-1(1)
C(13B)	25(1)	22(1)	30(1)	10(1)	11(1)	3(1)
C(14B)	27(1)	27(1)	19(1)	5(1)	6(1)	6(1)
C(15B)	21(1)	21(1)	21(1)	1(1)	1(1)	0(1)
C(16B)	17(1)	21(1)	18(1)	-1(1)	5(1)	0(1)
C(17B)	14(1)	23(1)	24(1)	3(1)	2(1)	-3(1)
C(18B)	20(1)	26(1)	31(2)	2(1)	11(1)	2(1)
C(19B)	23(1)	24(1)	29(1)	-2(1)	6(1)	0(1)
C(20B)	21(1)	35(2)	31(2)	3(1)	-6(1)	-6(1)

Table 5. Hydrogen coordinates (x 10^4) and isotropic displacement parameters ($Å^2$x 10^3) for d14137.

	x	y	z	U(eq)
H(2AA)	782	1561	7835	30
H(2AB)	-800	1171	7941	30
H(3AA)	1780	516	7344	50
H(3AB)	116	174	7228	50
H(4AA)	1054	-564	8627	33
H(4AB)	2508	-52	8961	33
H(5AA)	2024	-35	10919	22
H(6AA)	810	1048	11914	23
H(8AA)	-1132	1179	10215	26
H(8AB)	-77	1902	10339	26
H(9AA)	5352	1311	10164	31
H(9AB)	4588	2056	10461	31
H(12A)	8444	3380	9274	47
H(13A)	8077	3205	7391	60
H(14A)	6273	2369	6614	56
H(15A)	4838	1726	7702	41
H(18A)	-1818	-1835	10232	57
H(18B)	-3056	-1954	11024	57
H(18C)	-1403	-1677	11510	57
H(19A)	-3701	-83	11552	61
H(19B)	-2625	-617	12329	61
H(19C)	-4260	-885	11786	61
H(20A)	-4153	-296	9557	81
H(20B)	-4745	-1098	9747	81
H(20C)	-3403	-978	9044	81
H(2BA)	3600	-495	7206	27
H(2BB)	4972	69	7183	27
H(3BA)	6388	-886	6774	35
H(3BB)	4937	-1408	6579	35
H(4BA)	6312	-918	4942	31

H(4BB)	4598	-1211	4754	31
H(5BA)	3943	-198	3341	21
H(6BA)	2347	980	3662	20
H(8BA)	4004	1117	5506	20
H(8BB)	2283	954	5710	20
H(9BA)	597	-1637	4380	27
H(9BB)	-323	-910	4549	27
H(12B)	-3518	-2798	5617	28
H(13B)	-2928	-2690	7511	30
H(14B)	-895	-1963	8251	29
H(15B)	528	-1352	7115	26
H(18D)	8874	774	3216	38
H(18E)	9247	1615	3082	38
H(18F)	7710	1282	2461	38
H(19D)	6454	2303	4632	37
H(19E)	6167	2205	3336	37
H(19F)	7705	2560	3916	37
H(20D)	9209	908	5223	45
H(20E)	8258	1500	5777	45
H(20F)	9581	1752	5123	45
H(2NA)	3160(40)	1194(17)	11280(30)	15(8)
H(2NB)	1130(40)	-192(18)	3670(30)	21(8)
H(4NA)	-1440(40)	-216(19)	9850(30)	25(8)
H(4NB)	6590(40)	707(19)	4710(30)	14(8)

Table 6. Torsion angles [°] for d14137.

C(4A)-N(1A)-C(1A)-C(2A)	46.3(3)
C(5A)-N(1A)-C(1A)-C(2A)	-178.1(2)
C(4A)-N(1A)-C(1A)-C(8A)	-180.0(2)
C(5A)-N(1A)-C(1A)-C(8A)	-44.4(2)
C(4A)-N(1A)-C(1A)-C(7A)	-76.3(2)
C(5A)-N(1A)-C(1A)-C(7A)	59.3(2)
N(1A)-C(1A)-C(2A)-C(3A)	-32.8(3)
C(8A)-C(1A)-C(2A)-C(3A)	-144.8(3)
C(7A)-C(1A)-C(2A)-C(3A)	83.7(3)
C(1A)-C(2A)-C(3A)-C(4A)	10.3(3)
C(5A)-N(1A)-C(4A)-C(3A)	-164.4(3)
C(1A)-N(1A)-C(4A)-C(3A)	-38.5(3)
C(2A)-C(3A)-C(4A)-N(1A)	16.8(3)
C(4A)-N(1A)-C(5A)-C(16A)	-104.1(3)
C(1A)-N(1A)-C(5A)-C(16A)	130.8(2)
C(4A)-N(1A)-C(5A)-C(6A)	134.5(2)
C(1A)-N(1A)-C(5A)-C(6A)	9.3(2)
C(7A)-N(2A)-C(6A)-C(8A)	-40.8(2)
C(7A)-N(2A)-C(6A)-C(5A)	66.2(3)
N(1A)-C(5A)-C(6A)-N(2A)	-75.7(2)
C(16A)-C(5A)-C(6A)-N(2A)	163.4(2)
N(1A)-C(5A)-C(6A)-C(8A)	28.9(2)
C(16A)-C(5A)-C(6A)-C(8A)	-92.0(2)
C(9A)-N(3A)-C(7A)-N(2A)	-2.0(4)
C(9A)-N(3A)-C(7A)-C(1A)	177.9(2)
C(6A)-N(2A)-C(7A)-N(3A)	-173.8(3)
C(6A)-N(2A)-C(7A)-C(1A)	6.3(3)
N(1A)-C(1A)-C(7A)-N(3A)	108.6(3)
C(2A)-C(1A)-C(7A)-N(3A)	-5.0(4)
C(8A)-C(1A)-C(7A)-N(3A)	-149.1(3)
N(1A)-C(1A)-C(7A)-N(2A)	-71.4(2)
C(2A)-C(1A)-C(7A)-N(2A)	174.9(2)
C(8A)-C(1A)-C(7A)-N(2A)	30.8(3)
N(1A)-C(1A)-C(8A)-C(6A)	57.8(2)

C(2A)-C(1A)-C(8A)-C(6A)	171.4(3)
C(7A)-C(1A)-C(8A)-C(6A)	-51.8(2)
N(2A)-C(6A)-C(8A)-C(1A)	56.2(2)
C(5A)-C(6A)-C(8A)-C(1A)	-53.8(2)
C(7A)-N(3A)-C(9A)-C(10A)	-163.1(2)
N(3A)-C(9A)-C(10A)-C(15A)	-23.7(4)
N(3A)-C(9A)-C(10A)-C(11A)	155.2(3)
C(15A)-C(10A)-C(11A)-C(12A)	0.2(4)
C(9A)-C(10A)-C(11A)-C(12A)	-178.8(3)
C(15A)-C(10A)-C(11A)-Br(1A)	-179.5(2)
C(9A)-C(10A)-C(11A)-Br(1A)	1.5(4)
C(10A)-C(11A)-C(12A)-C(13A)	-0.1(5)
Br(1A)-C(11A)-C(12A)-C(13A)	179.7(3)
C(11A)-C(12A)-C(13A)-C(14A)	-0.2(6)
C(12A)-C(13A)-C(14A)-C(15A)	0.3(6)
C(11A)-C(10A)-C(15A)-C(14A)	-0.1(5)
C(9A)-C(10A)-C(15A)-C(14A)	178.9(3)
C(13A)-C(14A)-C(15A)-C(10A)	-0.1(6)
C(17A)-N(4A)-C(16A)-O(1A)	-2.4(5)
C(17A)-N(4A)-C(16A)-C(5A)	178.8(3)
N(1A)-C(5A)-C(16A)-O(1A)	170.2(2)
C(6A)-C(5A)-C(16A)-O(1A)	-76.5(3)
N(1A)-C(5A)-C(16A)-N(4A)	-10.9(3)
C(6A)-C(5A)-C(16A)-N(4A)	102.4(3)
C(16A)-N(4A)-C(17A)-C(20A)	177.5(3)
C(16A)-N(4A)-C(17A)-C(18A)	-63.2(4)
C(16A)-N(4A)-C(17A)-C(19A)	57.9(4)
C(4B)-N(1B)-C(1B)-C(2B)	47.0(3)
C(5B)-N(1B)-C(1B)-C(2B)	-176.6(2)
C(4B)-N(1B)-C(1B)-C(8B)	-179.6(2)
C(5B)-N(1B)-C(1B)-C(8B)	-43.2(2)
C(4B)-N(1B)-C(1B)-C(7B)	-75.5(2)
C(5B)-N(1B)-C(1B)-C(7B)	60.9(2)
N(1B)-C(1B)-C(2B)-C(3B)	-32.9(2)
C(8B)-C(1B)-C(2B)-C(3B)	-145.0(3)
C(7B)-C(1B)-C(2B)-C(3B)	83.9(3)

C(1B)-C(2B)-C(3B)-C(4B)	10.0(3)
C(5B)-N(1B)-C(4B)-C(3B)	-166.2(2)
C(1B)-N(1B)-C(4B)-C(3B)	-39.4(3)
C(2B)-C(3B)-C(4B)-N(1B)	17.2(3)
C(4B)-N(1B)-C(5B)-C(16B)	-108.4(3)
C(1B)-N(1B)-C(5B)-C(16B)	125.6(2)
C(4B)-N(1B)-C(5B)-C(6B)	133.4(2)
C(1B)-N(1B)-C(5B)-C(6B)	7.4(2)
C(7B)-N(2B)-C(6B)-C(8B)	-39.2(2)
C(7B)-N(2B)-C(6B)-C(5B)	67.1(2)
N(1B)-C(5B)-C(6B)-N(2B)	-74.1(2)
C(16B)-C(5B)-C(6B)-N(2B)	165.9(2)
N(1B)-C(5B)-C(6B)-C(8B)	30.6(2)
C(16B)-C(5B)-C(6B)-C(8B)	-89.4(2)
C(9B)-N(3B)-C(7B)-N(2B)	1.4(4)
C(9B)-N(3B)-C(7B)-C(1B)	-178.2(2)
C(6B)-N(2B)-C(7B)-N(3B)	-175.1(3)
C(6B)-N(2B)-C(7B)-C(1B)	4.5(2)
N(1B)-C(1B)-C(7B)-N(3B)	109.2(3)
C(2B)-C(1B)-C(7B)-N(3B)	-4.4(4)
C(8B)-C(1B)-C(7B)-N(3B)	-148.0(2)
N(1B)-C(1B)-C(7B)-N(2B)	-70.5(2)
C(2B)-C(1B)-C(7B)-N(2B)	175.9(2)
C(8B)-C(1B)-C(7B)-N(2B)	32.3(2)
N(1B)-C(1B)-C(8B)-C(6B)	57.89(19)
C(2B)-C(1B)-C(8B)-C(6B)	171.4(2)
C(7B)-C(1B)-C(8B)-C(6B)	-52.2(2)
N(2B)-C(6B)-C(8B)-C(1B)	55.5(2)
C(5B)-C(6B)-C(8B)-C(1B)	-54.8(2)
C(7B)-N(3B)-C(9B)-C(10B)	-151.1(2)
N(3B)-C(9B)-C(10B)-C(11B)	-170.0(2)
N(3B)-C(9B)-C(10B)-C(15B)	10.8(4)
C(15B)-C(10B)-C(11B)-C(12B)	0.8(4)
C(9B)-C(10B)-C(11B)-C(12B)	-178.4(3)
C(15B)-C(10B)-C(11B)-Br(1B)	-179.79(19)
C(9B)-C(10B)-C(11B)-Br(1B)	1.0(4)

C(10B)-C(11B)-C(12B)-C(13B)	-1.0(4)
Br(1B)-C(11B)-C(12B)-C(13B)	179.6(2)
C(11B)-C(12B)-C(13B)-C(14B)	0.5(4)
C(12B)-C(13B)-C(14B)-C(15B)	0.1(4)
C(13B)-C(14B)-C(15B)-C(10B)	-0.3(4)
C(11B)-C(10B)-C(15B)-C(14B)	-0.2(4)
C(9B)-C(10B)-C(15B)-C(14B)	179.0(2)
C(17B)-N(4B)-C(16B)-O(1B)	3.8(4)
C(17B)-N(4B)-C(16B)-C(5B)	-174.7(2)
N(1B)-C(5B)-C(16B)-O(1B)	178.1(2)
C(6B)-C(5B)-C(16B)-O(1B)	-70.2(3)
N(1B)-C(5B)-C(16B)-N(4B)	-3.4(3)
C(6B)-C(5B)-C(16B)-N(4B)	108.4(3)
C(16B)-N(4B)-C(17B)-C(20B)	178.3(3)
C(16B)-N(4B)-C(17B)-C(18B)	-63.0(3)
C(16B)-N(4B)-C(17B)-C(19B)	59.0(3)

Symmetry transformations used to generate equivalent atoms:

Table 7. Hydrogen bonds for d14137 [Å and °].

D-H...A	d(D-H)	d(H...A)	d(D...A)	<(DHA)
N(4A)-H(4NA)...N(1A)	0.89(4)	2.17(3)	2.672(3)	115(3)
N(4B)-H(4NB)...N(1B)	0.70(4)	2.30(3)	2.677(3)	116(3)
N(2B)-H(2NB)...O(1A)#1	0.85(4)	2.07(4)	2.913(3)	171(3)
N(2A)-H(2NA)...O(1B)#2	0.76(4)	2.13(4)	2.874(3)	165(3)

Symmetry transformations used to generate equivalent atoms:

#1 x,y,z-1 #2 x,y,z+1
